# Branched-Chain Amino Acid Degradation Pathway was Inactivated in Colorectal Cancer: Results from a Proteomics Study

**DOI:** 10.7150/jca.95454

**Published:** 2024-05-20

**Authors:** Shixian Lian, Siyuan Liu, Ao Wu, Lin Yin, Lei Li, Liyan Zeng, Mingkun Zhao, Lijun Zhang

**Affiliations:** 1 Shanghai Public Health Clinical Center, Fudan University, Shanghai 201508, China.; 2 Shanghai Ninth People's Hospital, Shanghai Jiaotong University School of Medicine, Shanghai 200011, China.

**Keywords:** colon cancer, AOM/DSS, proteomics, ALDH2, HCDH, branched chain amino acids.

## Abstract

**Background:** Colorectal cancer (CRC) ranks third in terms of cancer incidence and fourth in terms of cancer-related deaths worldwide. Identifying potential biomarkers of CRC is crucial for treatment and drug development.

**Methods:** In this study, we established a C57B/6N mouse model of colon carcinogenesis using azoxymethane-dextran sodium sulfate (AOM-DSS) treatment for 14 weeks to identify proteins associated with colon cancer. An isobaric tag for relative and absolute quantitation (iTRAQ)-based proteomic analysis was conducted on the cell membrane components enriched in the colonic mucosa. Additionally, tumor tissues and adjacent normal colon tissues were collected from patients with colon cancer for comparative protein and metabolite analyses.

**Results:** In total, 74 differentially expressed proteins were identified in the tumor tissue samples from AOM/DSS-treated mice compared to both the adjacent tissue samples from AOM/DSS-treated mice and tissue samples from saline-treated control mice. Bioinformatics analysis revealed eight downregulated proteins enriched in the branched-chain amino acids pathway (valine, leucine, and isoleucine degradation). Moreover, these proteins are already known to be associated with the survival rate of patients with cancer. Targeted metabolomics showed increased levels of valine, leucine, and isoleucine in tumor tissues compared to those in adjacent normal tissues in patients with colon cancer. Furthermore, a real-time PCR experiment demonstrated that Aldehyde dehydrogenase, mitochondrial (short protein name ALDH2, gene name *Aldh2*) and Hydroxyacyl-coenzyme A dehydrogenase, mitochondrial (short protein name HCDH, gene name *Hadh*) (two genes) in the pathway of branched-chain amino acids) were downregulated in patients with colon cancer (colon tumor tissues vs. their adjacent colon tissues). ALDH2 expression was further validated by western blotting in AOM/DSS-treated mouse model and in clinical samples.

**Conclusion:** This study highlighted the inactivation of the branched-chain amino acid degradation

pathway in colon cancer and identified ALDH2 and HCDH as potential biomarkers for diagnosing colon cancer and developing new therapeutic strategies.

## Background

Colorectal cancer (CRC) is the third most prevalent cancer and fourth leading cause of cancer-related deaths worldwide 1. The incidence of CRC is currently on the rise 2. Despite significant progress in early diagnosis and treatment, the 5-year overall survival rate for patients with CRC remains at just 63.5% 3. Improving the survival rate relies on early detection and a comprehensive understanding of the mechanisms underlying CRC development. Therefore, identifying potential biomarkers for predicting CRC progression and gaining new insights into the mechanisms of CRC development will contribute to the advancement of tumor treatment options and potentially prolong the survival of patients with CRC.

CRC is a complex disease influenced by various factors, including genetics, lifestyle, and inflammation. Patients with inflammatory bowel disease (IBD) have an increased risk of developing CRC 4, 5. To investigate the underlying mechanisms and potential biomarkers of colon cancer, researchers have developed animal models that induce tumor growth through inflammation by utilizing substances, such as azoxymethane (AOM) and dextran sulfate sodium (DSS) 6-8. AOM, a well-established carcinogen, creates DNA adducts 9, whereas DSS serves as an inflammatory agent that damages the epithelial lining of the colon 10. The combination of AOM and DSS induces significant inflammation, leading to the development of colon tumors, effectively mimicking the progression of colitis-accelerated colon cancer 6, 7. Despite some research on colitis-accelerated colon cancer, the precise molecular changes, especially at the cellular membrane level, remain unknown. Proteomics technology is ideal for the comparative analysis of proteins within cells or tissues in normal and diseased states, aiding in the identification of proteins involved in the pathogenic process. Recent literature reviews have highlighted the identification of numerous candidate protein biomarkers of CRC through proteomics studies 11-16. Examples include proteins such as β-catenin, decorin, septin-7, S100A10, and drebrin, which have been reported to be associated with CRC development and metastasis 15. Additionally, 14-3-3β has been linked to prognosis, with low levels of 14-3-3β associated with better outcomes 17. A combination of five proteins (fatty acid-binding protein 1, intelectin 1, transitional endoplasmic reticulum ATPase, transgelin, and tropomyosin 2) has provided valuable prognostic insights 18. However, the molecular mechanisms underlying CRC development and reliable biomarkers for CRC diagnosis remain limited.

In this study, we developed a mouse model using AOM and DSS to identify the proteins associated with CRC. An isobaric tag for relative and absolute quantitation (iTRAQ)-based proteomic study was performed using colon tumor tissues and adjacent normal tissues from AOM/DSS-treated mice, as well as normal colon tissues from saline-treated mice. Proteins in the branched-chain amino acid (valine, leucine, and isoleucine) degradation pathway were found to be downregulated in tumor tissues compared to the controls. ALDH2 (Aldehyde dehydrogenase, mitochondrial) and HCDH (hydroxyacyl-coenzyme A dehydrogenase, mitochondrial) were validated in the AOM/DSS-treated mouse model and clinical samples.

## Materials and Methods

### Materials

Colon-specific carcinogens, dextran sodium sulfate (DSS; MW = 36,000-50,000), and azoxymethane (AOM) 19, 20 were purchased from ICN Biomedicals Inc. (Aurora, Ohio) and Sigma-Aldrich Company (Steinheim, Germany), respectively. Na^+^/K^+^-ATPase Mouse Monoclonal Antibody [1G1] (RT1412) was bought from huaBIO company (Zhejiang province, China). Prohibitin antibody (2426) was from Cell Signaling Technology, Inc (Massachusetts (MA), USA). Anti-ALDH2 antibody (ab227021) was sourced from Abcam company (Cambridgeshire, UK), and β-actin antibody (66009-1-Ig) and GAPDH (60004-1-Ig) were obtained from PROTEINTECH company (Chicago, USA). Anti-Rabbit IgG (H+L) Antibody, Peroxidase-Labeled (074-1506), anti-Mouse IgA + IgG + IgM (H+L) Antibody, Human Serum-Adsorbed and Peroxidase-Labeled (074-1807) were provided by KPL Inc. (Massachusetts, USA). The iTRAQ Reagent-8Plex Multiplex Kit and RevertAid Reverse Transcriptase (EP0441) were obtained from Thermo Fisher Scientific (California, USA), and the ultra-high sensitivity ECL Kit was from Millipore company (Now Merck company, Massachusetts, USA). The PerfectStart Green qPCR SuperMix (AQ601-04) was purchased from TransGen Biotech (Beijing, China). Other analytical reagents were obtained from Sinopharm Chemical Reagent Co., Ltd. (Shanghai, China).

### Patients and clinical sample collection

This study received approval from Shanghai Public Health Clinical Center (Approval No. 2019-S035-02), and a written informed consent was obtained from each participating patient. A total of twenty-one patients who underwent surgical resection at Shanghai Public Health Clinical Center were randomly selected from October 2020 to August 2022. The patients were pathologically diagnosed according to the standard of the 8^th^ edition of the American Joint Committee on Cancer/International Union against cancer classification. All patients were Chinese. We enrolled a total of ten males and eleven females with mean age of 62.8±12.1 (range, 42-84) years and differentiation of II to IV. Eight patients with lymph nodes lymph node metastasis and four with distal metastasis were included. Patients with age less than 18, pregnancy, breastfeeding, HIV, and TB infection were excluded. Colon tumor tissues and adjacent normal colon tissues were collected during the surgical resection for pathological examination. Residual tissues from these 21 pairs were utilized for subsequent real-time PCR (n = 14 pairs), western blot (WB) analysis (n = 5 pairs) and metabolomics study (n = 7 pairs). Clinical data, including gender, age, tumor size, and metastasis information, were meticulously documented for each patient (Supplement table 1 (Table S1)). The fresh colon tissues were cut into 1-2 cm sizes, washed with normal saline solution for three times, and stored into -80°C for further use.

### Colon cancer mouse model developed by AOM/DSS treatment

The development of a colon cancer model through AOM/DSS treatment was approved by the Ethics Committee of Shanghai Public Health Clinic Center (2014-A032-02) and followed established procedures 6. Briefly, male C57BL/6N mice, aged 6-8 weeks, were obtained from Shanghai Public Health Clinical Center and divided into two groups: the AOM/DSS-treatment group and the control group, each consisting of 10 mice. After one week of free access to water and food, mice in the AOM/DSS-treatment group were intraperitoneally injected with one dose of AOM (12.5 mg/kg body weight in saline) and were provided 2.5% DSS (in drinking water) for seven days, followed by two weeks of drinking water. This cycle of one week of water containing DSS followed by two weeks of normal drinking water was repeated over a 9-week period. Subsequently, these mice were maintained without receiving any drugs until the 14th week, and are referred to as the “AOM/DSS group.” Control animals, denoted as the “saline group”, were intraperitoneally injected with a single dose of saline (0.2 mL), corresponding to the AOM injection, and had continuous access to drinking water. At the end of 14^th^ week, all mice were anesthetized by intraperitoneal injection of pentobarbital sodium (10 mg/mL in saline) at a dose of 50 mg/kg (50 mg drug per kg mouse body weight), and tumor development was monitored using colon capsule endoscopy. After endoscopy detection, the mice were fed normally for two days, subsequently fasted for 10 h with normal water drinking, and then euthanized by intraperitoneal injection of pentobarbital sodium (50 mg/mL in saline) at the volume of 1.0 mL, which corresponds to a dose of approximately 1850 mg/kg 21. After making sure the mouse death through observing the pain response disappearance, and cardiac and respiratory arrest, the mouse was dissected. Tumor sizes were measured using a vernier caliper. The fresh colon was longitudinally dissected and thoroughly rinsed multiple times with normal saline solution till the intestinal wall clean. A quarter of the colon tissues was fixed in formaldehyde for pathological examination, another quarter was frozen for future use, and the remainder was collected for mucosal separation, membrane enrichment, and western blot (WB) analysis.

### Colon mucosa separation and purity detection

The mucosal layer was carefully peeled under anatomical microscopy guidance. In the AOM/DSS group, tumor tissues with intact mucosa were dissected for mucosal separation (referred to as Part 1). If the mucosal layer was disrupted in the tumor tissues, the tumors were directly extracted (referred to as Part 2). Part 1 and Part 2 were combined for membrane enrichment (referred to as the “tumor” sample). The adjacent normal colon tissues in the AOM/DSS model and the normal colon tissues in the saline-treated group were subjected to mucosal separation and labeled as “adjacent” and “normal”, respectively. Finally, a portion of the separated mucosa was preserved in formalin for subsequent pathological examination using hematoxylin and eosin (HE) staining, and the remainder was designated for cell membrane enrichment. The mucosa of every 3 mice were mixed together, and a total of 3 mixed samples from 10 mice were prepared. The three samples (triple biological repetitions) prepared in this way were used for following experimental processes, including membrane enrichment, protein digestion, iTRAQ labeling, mass spectrometry detection and western blot analysis.

### Cell membrane enrichment

The separated mucosal tissues were homogenized in phosphate buffer saline (PBS) at a ratio of 1:2 (tissue weight to buffer volume). After centrifugation at 700 × g for 10 minutes to remove unbroken cells and nuclei, the supernatant was collected. Subsequently, another centrifugation step was performed for 30 min at 10,000 g to isolate the cell membrane pellet. All these procedures were conducted at a temperature of 4°C to preserve membrane integrity. The effectiveness of membrane enrichment was assessed through WB analysis, utilizing specific markers associated with the plasma membrane and mitochondria.

### iTRAQ-based proteomic study

(1) Protein extraction, quantification, and digestion: The membrane-enriched samples underwent pretreatment with a lysis buffer composed of 5 M Urea, 2 M Thiourea, 4% CHAPS, 1% NP-40, 65 mM dithiothreitol, and 0.5 mM phenylmethylsulfonyl fluoride (PMSF). Protein quantification was determined using the Bradford method (Bio-Rad, Hercules, CA, USA). Subsequently, 100 µg of proteins were subjected to reduction, alkylation, and trypsin (Promega (Madison, USA)) digestion at a protein-to-trypsin ratio of 20:1 at 37°C overnight.

(2) iTRAQ labeling: Peptide mixtures were labeled using the 4-plex iTRAQ labeling kit, following the iTRAQ™ Reagent protocol and our prior descriptions 22, 23, with 117 and 116 for tumor tissues and adjacent colon tissues from AOM/DSS-treated mice, while 114 for the saline-treated group, respectively. After incubated at room temperature for one hour, all iTRAQ-labeled tryptic peptide solutions were mixed.

(3) Peptide separation and mass spectrometry detection: The labeled peptides were fractionated into 14 segments by strong cation-exchange (SCX) chromatography on an UltiMate high-performance liquid chromatography (HPLC) system (LC Packings, The Netherlands) using a polysulfoethyl A column (2.1µm [diameter] x 150 mm [length], 5 µm [particle size] (Agilent Technologies, USA). The elution was carried out through utilizing two mobile phases: (A) 10 mmol/L KH_2_P0_4_ and 25% ACN (pH 2.6), and (B) 10 mmol/L KH_2_P0_4_, 350 mmol/L KCl, and 25% ACN (pH 2.6). A gradient elution started from 0% B, increasing to 80% B, and returning to 0% B over one hour at a flow rate of 0.2 mL/min. Subsequently, the 14 fractions were dried in a vacuum concentrator, resolved in 50 µL water with 0.1% formic acid, and used for Nano LC-MS/MS analysis.

For the Nano LC-MS/MS analysis, each fraction (2 µL) was loaded onto a trap column (Thermo Scientific Acclaim PepMap C18, 100 μm×2 cm) for a 3-minute duration at a flow rate of 10 μL/min. The sample was then separated using the analytical column (Acclaim PepMap C18, 75 μm × 25 cm) with a linear gradient, progressing from 5% to 30% phase B over 95 minutes. Phase A consisted of water with 0.1% formic acid, and phase B was composed of ACN with 0.1% formic acid. The flow rate of the analytical column was maintained at 300 nL/min, and the column temperature was held at 45°C. The Q-Exactive mass spectrometer was operated in a data-dependent mode with full-scan MS spectra from 350-1600 m/z at a resolution of 70 K, followed by fifteen sequential high-energy collisional dissociation (HCD) MS/MS scans with a resolution of 17.5 K. For each case, a single microscan was recorded, with dynamic exclusion set at 30 seconds 24, 25. Three technical replications were conducted for Nano LC-MS/MS analysis.

(4) Database search: The raw mass spectrometry data files were processed using Proteome Discoverer software (Thermo Fisher Scientific, version 1.4.0.288), and the MS/MS results were analyzed using Mascot software (Matrix Science, London, UK; version 2.3). Search parameters were as follows: Mus musculus as the taxonomy in the Uniprot-SwissProt database, trypsin enzyme specificity, a fragment ion tolerance of 0.050 Da, and a parent ion mass tolerance of 10 PPM. Carbamidomethyl modification of cysteine, iTRAQ 8-plex labeling of lysine, and the N-terminus were considered as fixed modifications, while oxidation of methionine and iTRAQ 8-plex labeling of tyrosine were designated as variable modifications. Peptide-level false discovery rates (FDR) were controlled lower than 1% by the percolator algorithm. Protein groups considered for quantification required at least one peptide with 95% confidence. Protein quantification relied on unique peptides as well as shared peptides. The experimental bias was corrected via normalization based on the protein median, with a minimum of 100 proteins required for normalization.

### Quantification of metabolites using mass spectrometry

Overall, seven pairs of colon cancer tissue samples and their corresponding adjacent colon tissues (refer to Table S1) were used. Colon tissues were dissected into 1-2 mm^3^ fragments, washed with PBS to remove blood, and ground under liquid nitrogen. Metabolites were extracted at a tissue weight-to-extract volume ratio of 1:2.5 using acetonitrile: methanol (1:1) containing internal standards (200 ng/mL L-valine-d8 and 200 ng/mL L-isoleucine-d10). After thorough mixing, samples were frozen at -80°C overnight, thawed at room temperature (approximately 25°C), subjected to ultrasonic crushing for 10 minutes at 4°C, and centrifuged for 10 minutes at 12,000 rpm at 4°C. The supernatant was collected and the pellet was re-extracted using an equal volume of extract by repeating the above-mentioned procedure. The newly obtained supernatant was combined with the previous supernatant. Finally, 20 µL of the supernatant was diluted with 380 µL of water. The diluted solution (10 µL) was loaded onto an ultra-high-performance liquid chromatography/tandem mass spectrometry (UHPLC-MS) system (Waters Acquity UPLC system and AB Sciex Triple Quad 5500 mass spectrometer) for metabolite quantification. Compounds were separated using an Agela Venusil ASB C18 2.1*50 mm (Agela Technologies, California, USA) column using the mobile phase of 5 mM ammonium formate (A) and acetonitrile with 0.6% formic acid (B). Mass spectrometry detection was performed using multiple reaction monitoring (MRM) in the positive-ion mode. The analytes were detected three times in MRM mode using the MS conditions described in Table S2.

### Bioinformatics analysis

The set of differentially expressed proteins (n = 74) was analyzed using the STRING software (version 11.5, https://string-db.org) 26. The analysis encompassed the examination of cellular components (CC), molecular functions (MF), biological processes (BP), KEGG pathways, and protein-protein interactions. To enhance the accuracy of the biological analysis, a minimum required interaction score of 0.7 (indicating high confidence) and an FDR lower than 0.05 were employed as selection criteria. In cases where the number of description items for the CC, MF, BP, and KEGG pathways (as exported from the STRING software) exceeded 10, only the top 10 were extracted. The protein network derived from the STRING software was subsequently imported into Cytoscape software (version 3.7.1) 27, and an interaction map was drawn using this software.

Furthermore, the 74 differentially expressed proteins were entered into the Clinical Proteomic Tumor Analysis Consortium (CPTAC) database (cProSite-Cancer Proteogenomic Data Analysis Site) (https://cprosite.ccr.cancer.gov/) 28 to compare the relative protein abundance in tumor and normal tissue from patients with colon cancer. Moreover, proteins that were observed to have an abundance similar to that reported in the CPTAC data, were input to the TCGA database (http://gepia2.cancer-pku.cn/#survival) to analyze the relationship between protein expression and survival rate in the dataset of colon adenocarcinoma (COAD). Finally, the eight proteins (enriched in the leucine, isoleucine, and valine degradation pathway) were used to analyze the relationship between their protein expression and the survival rate of patients in any of the 33 cancer datasets from the TCGA database.

### Western blot analysis

Protein extraction (25 μg) was initially separated by SDS-PAGE, followed by transferring onto a PVDF membrane. Subsequently, the membrane was incubated with primary antibodies: Na^+^/K^+^-ATPase (a plasma membrane biomarker) at a 1:2000 dilutions, prohibit (a mitochondrial apparatus biomarker) at a 1:4000 dilutions, β-actin at a 1:5000 dilutions, or ALDH2 at a 1:500 dilutions. This incubation process occurred overnight at 4°C. Afterwards, the PVDF membrane was washed three times with TBS/Tween 20 and then exposed to a secondary antibody at a 1:5000 dilutions for one hour. The protein bands were visualized using an ultra-high-sensitive ECL Kit. This experimental procedure was performed in triplicate for technical or biological replications and consistency.

### Real-time PCR

Total RNA was extracted from frozen human colon tissues, including 14 pairs of tumor tissues and their adjacent colon tissues (refer to Table S1). The extraction process was performed in accordance with the manufacturer's instructions, including tissue cutting, cryogenic tissue disruption, and RNA extraction using a Trizol Kit (Thermo Fisher Scientific, USA). Subsequently, the RNA was reversely transcribed into cDNA using RevertAid Reverse Transcriptase (EP0442, Thermo Fisher Scientific, USA). Primer pairs for the differentially expressed genes were designed using Primer 5.0 software. The RT-qPCR reactions were conducted on an ABI 7500 real-time fluorescence quantitative PCR system (Thermo, USA) using PerfectStart Green qPCR SuperMix (AQ601-04, TransGen Biotech, Beijing, China), following the manufacturer's protocol. The reaction conditions consisted of an initial denaturation step at 95°C for 3 minutes, followed by 40 cycles of 95°C for 15 seconds, 59°C for 20 seconds, and 72°C for 30 seconds. To confirm primer specificity, a melting curve was generated by incrementally heating the amplicon from 60°C to 95°C. Each qPCR analysis was performed in triplicate. The forward and reverse primers of *Aldh2*, *Hadh* and *GAPDH* were ACAAAAGGGGACGAACACAG and GTGAGCCTGCTGTGAATCAA for* Aldh2*; CACACAGTAGTGTTGGTAGACC and TGCCACTTTCCTAAGGCTTTC for *Hadh*, and CCAGGTGGTCTCCTCTGA and GCTGTAGCCAATTCGTTGT for *GAPDH*, respectively. *GAPDH* was used as a reference gene. Comparative quantification was performed using the 2^-ΔΔCq^ method 29.

### Statistical analysis

For the proteomic study, quantitative data were statistically analyzed using Microsoft Excel 2010 (Microsoft, Washington, USA). The differentially expressed proteins were defined as a fold change of ≥ 1.50 or ≤ 0.666, with *p* < 0.05, for cancer-to-normal (117:114) and cancer-to-adjacent (117:116) comparisons, respectively, while, adjacent-to-normal (116:114) and normal-to-adjacent (114:116) comparisons were between 0.667 and 1.5 in all three replicates. R package software (version 4.0.2) (RStudio Inc., MINNESOTA, USA) was used to analyze the volcano plot, Venn diagram, and clustering data 30. For real-time PCR quantification and western blotting, statistical analyses were performed using GraphPad Prism software (version 8.3.0) (GraphPad Company, California, USA). Student's unpaired t-test was used to compare differences between two groups. Significant differences among multiple groups were determined using one-way analysis of variance (ANOVA) with multiple comparisons and adjusted using Tukey's post-hoc test. Differences were considered statistically significant at *p* < 0.05.

## Results

### Animal model of colon cancer

**Figure 1:**
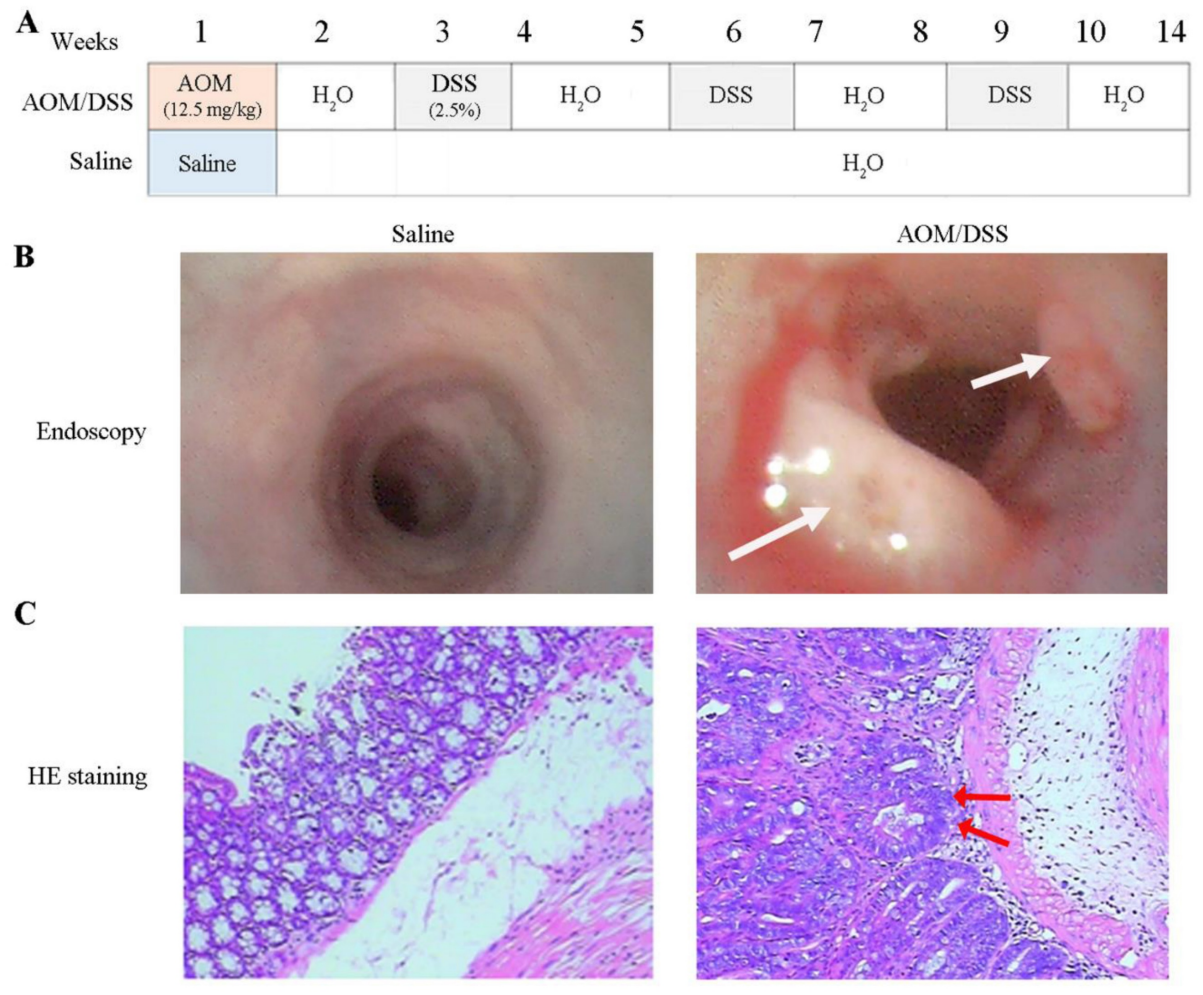
** Development of colon cancer model with AOM/DSS treatment. (A)** The experiment processes of tumor development. Mice were euthanized at the 14^th^ week. AOM and DSS represent azoxymethane, and dextran sodium sulfate, respectively. **(B)** Colonoscopy. Tumors were highlighted by white arrows. **(C)** Pathological examination of colon tissues. The nuclei of colonic mucosal epithelium are large and deep-stained, with focal adenocarcinoma of glandular epithelium and tumor stroma specified by red arrows.

A mouse model was established using AOM/DSS treatment to identify proteins related to colon cancer, as depicted in Figure 1A and as previously described 8. The total duration of the study was 15 weeks and two days, including one week of free feeding, 14 weeks for model development, and two days between capsule endoscopy and euthanasia. No mice died during the study. Endoscopic examination after 14 weeks of AOM/DSS treatment revealed colon abnormalities such as bleeding, edema, erosion, and mucosal protrusions (Figure 1B, right). By contrast, the saline-treated control group exhibited smooth, pink intestinal walls (Figure 1B, left). The 10 AOM/DSS-treated mice developed a total of 35 tumors with a mean tumor size of 8.58±3.97 mm^2^. However, no tumors were detected in any of the mice treated with saline (Table S3 and supplement Figure 1 (Figure S1), and Figure S2). HE staining revealed distinct differences in the colonic mucosal epithelium, with notable adenocarcinoma in the glandular epithelium and tumor stroma (Figure 1C, right) in the AOM/DSS-treated group, whereas no pathological changes were observed in the saline-treated group (Figure 1C, left).

### Mucosa separation and membrane enrichment

**Figure 2:**
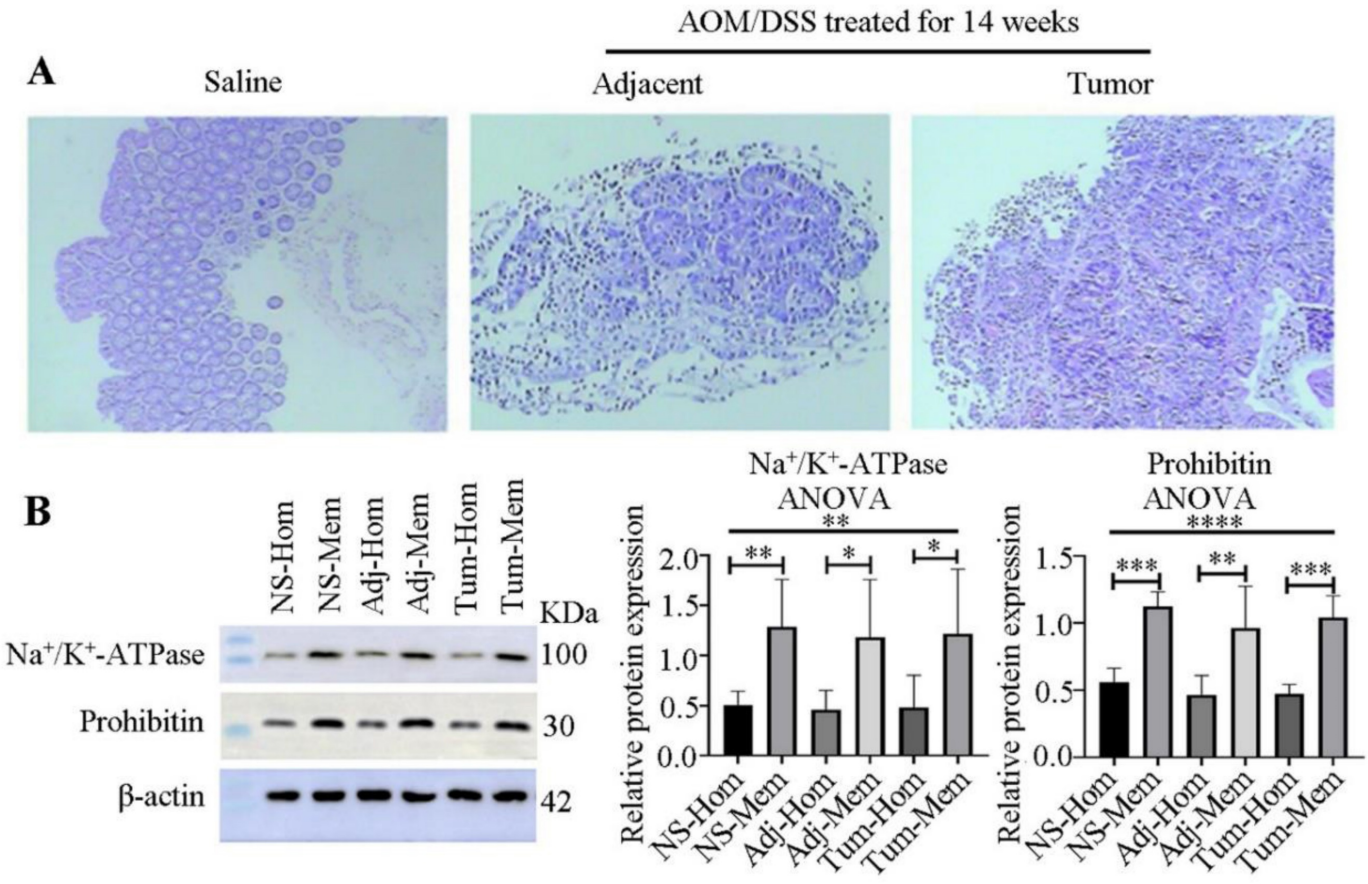
** Verification of mucosa separation and membrane enrichment. (A)** Pathological examination of mucosa separation from colon tissues, stained with HE. From left to right: Mucosae from the control group (saline-treated), adjacent normal colon tissues and tumor tissues treated with AOM/DSS for 14 weeks. **(B)** Western blot verification of membrane enrichment (left) and semi-quantitative analysis of protein bands using ImageJ software (right). Student's t-test analysis was used to compare differences for two groups. Significant differences among multiple groups were determined using a one-way analysis of variance (ANOVA). Significance levels are denoted as *, **, and *** for p-values less than 0.05, 0.01, and 0.005, respectively. Abbreviations: NS, Adj, Tum, Hom, and Mem correspond to saline treatment, adjacent colon tissues from AOM/DSS-treated mice, tumor tissues from AOM/DSS-treated mice, homogenate, and membrane, respectively.

Because colon tumors typically originate from mucosal tissues, we stripped the mucosa and verified its purity through HE staining (Figure 2A), confirming no contamination with muscle or other tissues.

To explore the membrane proteins associated with colon cancer in mucosal tissues, we employed differential centrifugation techniques for membrane enrichment. The results, as presented in Figure 2B, indicated a 2.5-, 2.6- and 2.5-fold enrichment of the plasma membrane in the normal colon tissues from saline-treated groups, and adjacent and colon tumors from AOM/DSS-treated groups, respectively, for 14 weeks. Additionally, mitochondria were enriched 2.0-, 2.1-, and 2.2-fold in the three kinds of samples, respectively.

### Differential proteomics study

**Figure 3:**
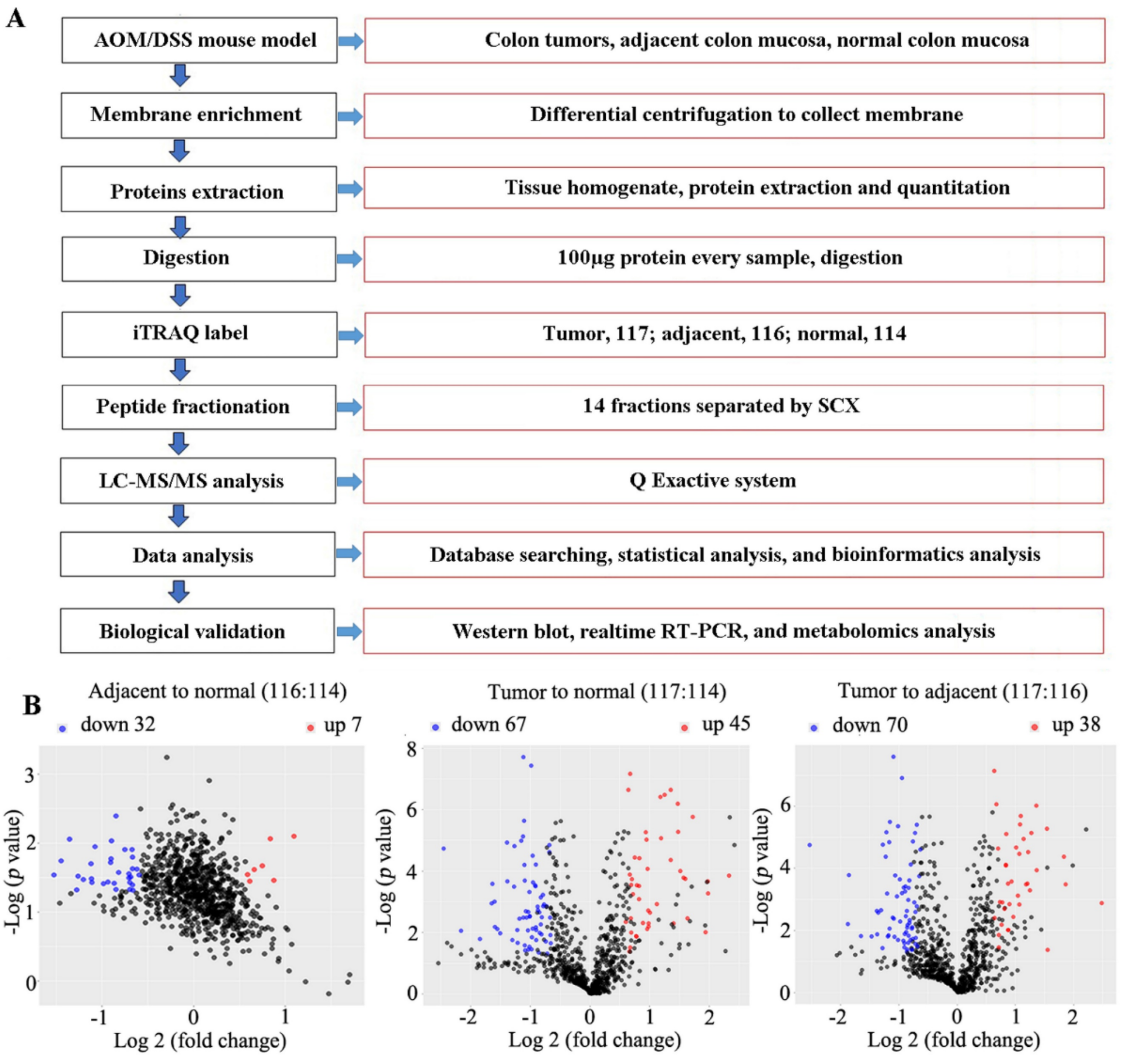
** The flow chart of experiment processes and volcano plot analyzed by R software. (A)** flow chart. **(B)** Volcano plot illustrating the differentially expressed proteins in adjacent compared to normal (left), cancer to normal (middle) or adjacent colon tissues (right). Mice were induced to develop colon cancer via AOM/DSS treatment, and colon tissues were collected after 14 weeks. The x-axis represents the average fold change (Log 2), while the y-axis represents the negative *p*-value (-Log 10). Fold change of ≥1.5, or ≤0.666, and *p* < 0.05 were defined as difference. Blue, red and black dots denote downregulated, upregulated and no differential proteins, respectively.

**Figure 4:**
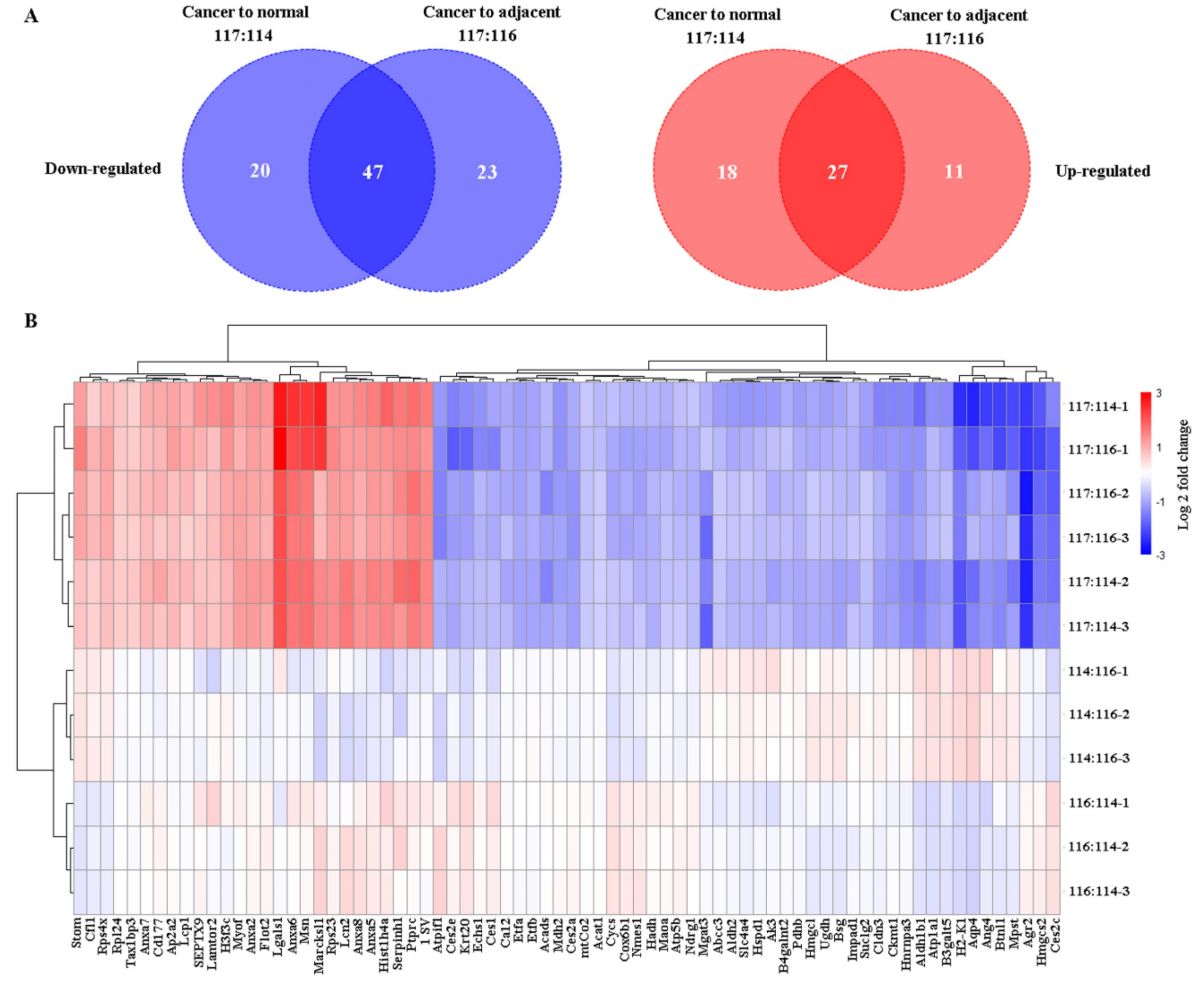
** Evaluation of the differentially-expressed proteins using R software. (A)** Venn diagrams depicting the number of differential proteins in cancer compared to normal (117:114) or adjacent colon (117:116) tissues in comparison to the saline-treated control. Left, the count of downregulated proteins; right, the count of upregulated proteins. **(B)** Clustering of the differential proteins using a heatmap. Downregulated proteins are represented in blue, while upregulated proteins are shown in red. Color intensity indicates differences in protein expression levels, with the color bar representing a Log 2^fold change^. The y-axis displays the three labeling ratios in triple replications (-1, -2, and -3), and the differentially expressed proteins (n = 74) are displayed at the bottom of the image.

Using iTRAQ-based proteomics technology, 1212 (Table S4), 1070 (Table S5), and 1036 (Table S6) proteins were identified with iTRAQ quantification information in replicates 1, 2, and 3, respectively. Of them, 67 and 45 downregulated and upregulated proteins, respectively, (Table S7) were identified in cancer tissue relative to normal tissue, and 70 and 38 downregulated and upregulated proteins, respectively, in the cancer tissue relative to adjacent tissue (fold change of ≥ 1.5 and ≤ 0.666, and *p* < 0.05 for upregulated and downregulated genes, respectively) (Table S8, Figure 3). In total, 74 common differential proteins were identified, including 47 downregulated and 27 upregulated proteins, in cancer tissues compared to both normal and adjacent tissues, as shown in Figure 4, Table S9 and Table S10.

### Bioinformatics analysis

**Figure 5:**
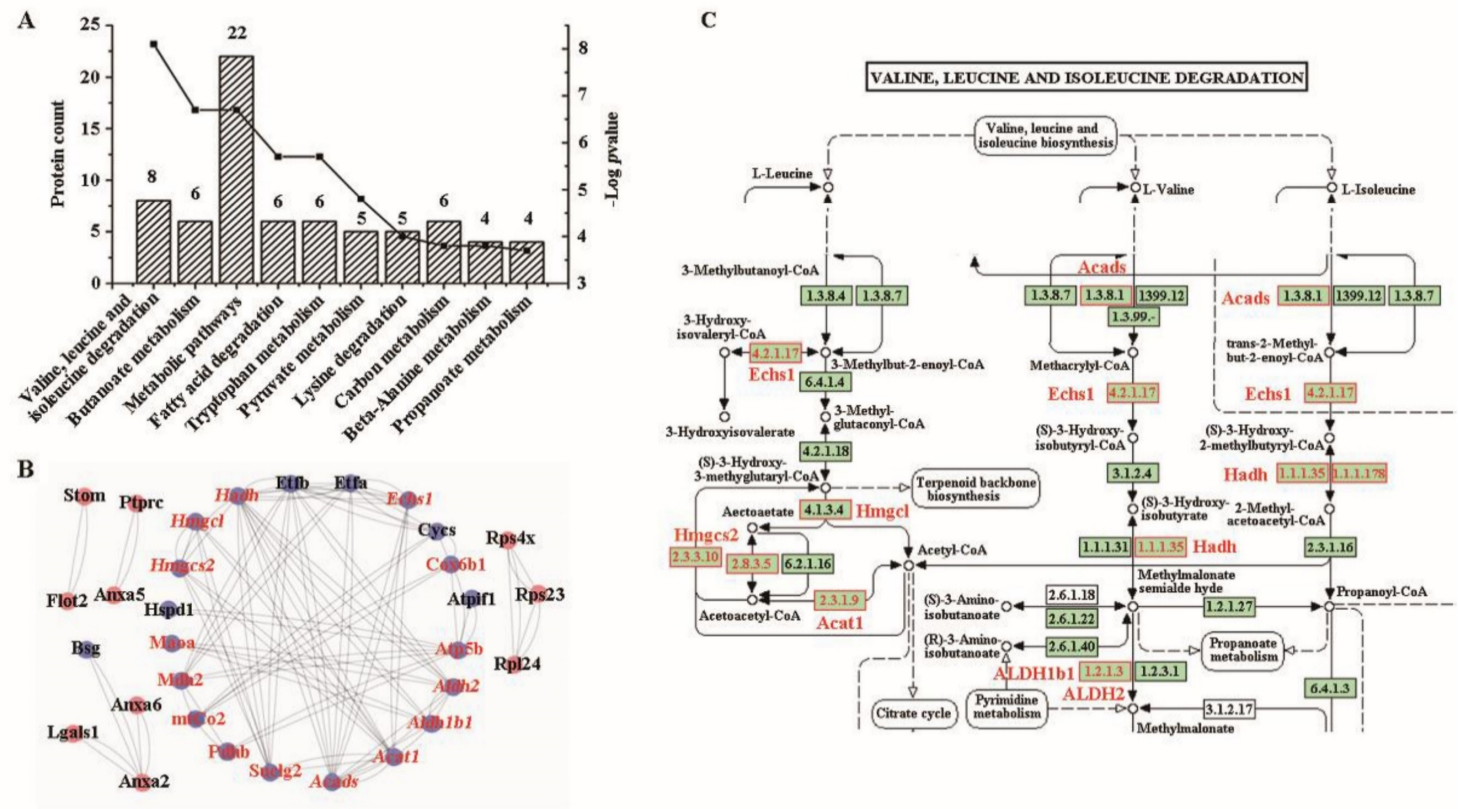
**Bioinformatics analysis of KEGG pathway and protein-protein interaction**. **(A)** KEGG pathway. **(B)** a protein-protein interaction network drawn by Cytoscape software. The downregulated proteins are highlighted by blue background. Proteins involved in metabolic pathways are highlighted in red, and those in valine, leucine and isoleucine degradation are highlighted in italics. **(C)** The pathway of valine, leucine and isoleucine degradation. The differential proteins are denoted with red boxes and printed labels.

According to the annotation of CC in the GO database, approximately 14.8% of the proteins (n = 65) were associated with cellular components related to intracellular organelles, while 13.9% (n = 61) were associated with membrane-bound organelles (Figure S3A)). In the MF, these proteins were predominantly related to binding and enzyme activities (Figure S3B). Regarding biological processes, the top nine items were surprisingly related to metabolic processes, except for the first item which was related to cellular processes (Figure S3C). Notably, all top KEGG pathways (Figure 5A) were involved in metabolism. All genes enriched in metabolism were downregulated in the mucosa of colon cancer tissues compared to adjacent or normal colon tissues (Table S10). The most significantly enriched pathway (highest -log *p-*value) involved valine, leucine, and isoleucine degradation, including genes such as *Echs1, Hadh, Hmgcl, Aldh2, Acads, Acat1, Aldh1b1,* and* Hmgcs2* (Table 1).

When comparing the relative abundance of the 74 differentially expressed proteins with the data from CPTAC, we found 40 proteins with relative protein abundance consistent with information in the COAD dataset, including 33 with statistically significant difference (*p* < 0.05) (27 downregulated and 6 upregulated proteins), and 7 without significant difference (3 downregulated and 4 upregulated). However, 18 proteins had abundance that was different from that reported in the COAD dataset, including 17 proteins with statistically significant difference (*p* < 0.05) (4 downregulated and 13 upregulated proteins) and one downregulated protein (*p* > 0.05). Furthermore, 16 of the 74 differentially expressed proteins were not identified in the CPTAC database (Table S10). For the proteins enriched in the valine, leucine and isoleucine degradation pathway, only HMGCl was not reported in CPTAC, whereas all others had consistent protein abundance (*p* < 0.05) (Table 1).

According to the survival rate analysis from the TCGA database, we found that four genes (from the 33 consistent proteins with *p* < 0.05) were related to the survival rate in patients with COAD, including *B4galnt2*, *Hmgcs2*, *Serpinh1* and *Suclg2*. However, only* Hmgcs2* and *Suclg2* were enriched in the KEGG pathways, with *Hmgcs2* in the valine, leucine, and isoleucine degradation pathway and *Suclg2* in a metabolic pathway (Table S11). Further bioinformatics analysis discovered that all eight genes in the valine, leucine, and isoleucine degradation pathway were related to the survival rate of patients with kidney renal clear cell carcinoma (KIRC), and six of the eight proteins were related to the survival rate of patients with kidney renal papillary cell carcinoma (KIRP) (Table S11).

To elucidate the interplay among these differential proteins, a protein-protein network was constructed using Cytoscape software. Remarkably, 20 proteins downregulated in cancer, including all eight proteins enriched in the valine, leucine, and isoleucine degradation pathway, were found to interact with each other (Figure 5B). By carefully checking the pathway of valine, leucine, and isoleucine degradation, we found that these differentially expressed proteins covered most of the signaling pathways (Figure 5C).

### Verification of HCDH and ALDH2 expression

**Figure 6:**
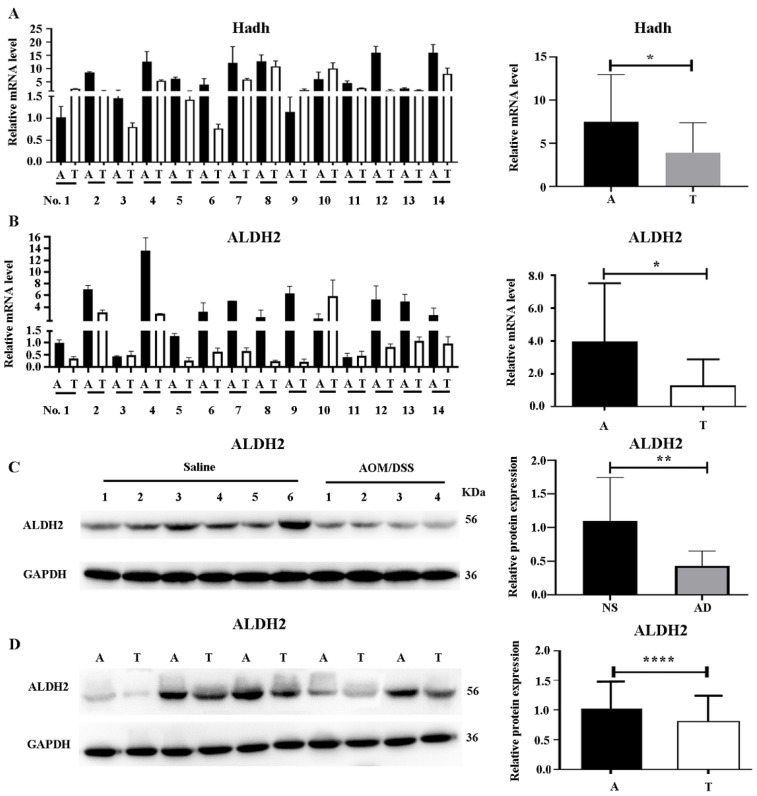
** Verification of ALDH2 and HCDH expression in mouse model and clinical samples. (A)** Verification of *Hadh* expression by real-time PCR in human colon cancer tissues (left) and quantification by Image J software (right). **(B)** Verification of *Aldh2* expression by real-time PCR in human colon cancer tissues (left) and quantification by Image J software (right). **(C)** Verification of ALDH2 by western blot (WB) in mouse model (left), and quantification by Image J software (right). **(D)** Verification of ALDH2 by WB in human colon cancer tissues (left) and quantification by Image J software (right). T and A represent tumor and adjacent colon tissues, respectively. Statistical analysis was performed by using the unpaired student's t-test. *, **, *** and ****, represent p<0.05, 0.01, 0.005 and 0.001, respectively.

Given that most differentially expressed proteins in the valine, leucine, and isoleucine degradation pathway were downregulated, the expression of HCDH and ALDH2, two key proteins in this pathway, was analyzed in 14 pairs of colon cancer and adjacent colon tissues from 14 patients. As shown in Figure 6A and 6B, the mRNA levels of both *Hadh* and *Aldh2* were significantly downregulated in colon cancer tissues compared to their adjacent colon tissues.

ALDH2 (a protein in the trunk node of the valine, leucine, and isoleucine degradation pathway) was selected for further WB experiments in the mouse model as well as the clinical samples. As shown in Figure 6C, ALDH2 exhibited reduced expression in colon cancer tissues obtained from the AOM/DSS-treated mice compared to that in tissue from the saline-treated controls. Further WB validation with five selected pairs of colon cancer tissues and their adjacent colon tissues also confirmed a significant decrease in ALDH2 expression in tumor tissues (Figure 6D).

### Upregulation of valine, leucine, and isoleucine in colon cancer

**Figure 7:**
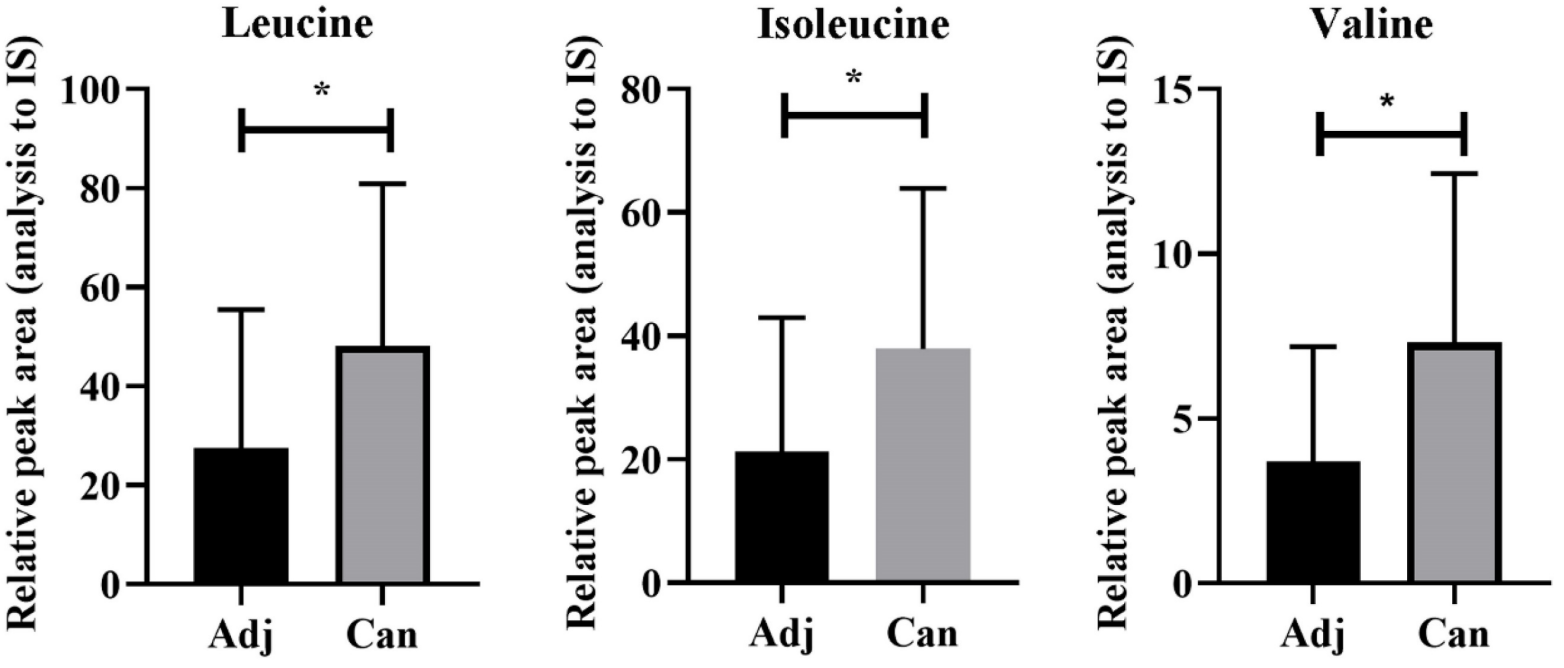
**Upregulation of leucine, isoleucine, and valine in cancer as detected by UHPLC-MS/MS.** Adj and Can correspond to adjacent colon and tumor tissues from colon cancer, respectively. The asterisk (*) indicates *p* < 0.05.

To validate the dysregulation of the valine, leucine, and isoleucine degradation pathway observed in the animal models, we conducted targeted metabolomics on cancer tissues and adjacent tissues from patients with colon cancer, revealing a significant upregulation of valine, leucine, and isoleucine in colon cancer tissues compared to adjacent tissues (Figure 7 and Supplement Table S12).

## Discussion

Colon cancer poses a significant global health challenge, as indicated in previous studies 31. In this study, membrane proteomic analysis was conducted on the intestinal mucosa from an AOM/DSS-induced mouse model of colon cancer. Although previous proteomic studies utilizing the AOM/DSS mouse model have explored proteins related to colon cancer 32-37, membrane proteomic studies are limited, hindering the investigation of lower abundance and insoluble membrane proteins. In this study, membrane proteomic analysis revealed the regulation of metabolic pathways in tumor tissues compared with that in the adjacent normal colon tissues. Most solid cancers, including CRC, exhibit inherent metabolic characteristics, such as aerobic glycolysis and abnormal mitochondrial metabolism 38. Therefore, targeting cancer-specific metabolism is a promising strategy for the treatment of CRC 38, 39. Although metabolic dysregulation is a hallmark of cancer 40, its investigation in the context of colon cancer remains limited. Our study of cancer metabolism may offer new clues for the development of novel targeted therapies 41. In the present study, almost half of the proteins (eight in total) involved in the valine, leucine, and isoleucine degradation pathway were downregulated in colon cancer tissue. Based on these observations, we hypothesized that inactivation of this pathway could lead to reduced valine, leucine, and isoleucine degradation, thereby promoting cancer cell growth and development. Elevated levels of branched-chain amino acids (BCAAs) such as valine, leucine, and isoleucine have been associated with various health issues, including cancer 42. As cancer cells require essential nutrients for growth and proliferation 43, BCAAs play a vital role by being utilized in various biosynthetic pathways 44, 45. Furthermore, cancer cells reprogram their BCAA metabolism to facilitate cancer progression 46. Previous reports have highlighted increased BCAA levels in the plasma/sera and tissue samples of patients with breast cancer 47, and pancreatic cancer 48, whereas, downregulated protein expressions in BCAA pathway in intrahepatic cholangiocarcinoma 49. Similar findings were observed in the present study, with elevated levels of valine, leucine, and isoleucine detected in tumor tissues compared to those in adjacent normal tissues from patients with colon cancer.

To investigate the potential patho-clinical characteristics of proteins in the BCAA pathway, we analyzed the relationship between protein expression and the survival rate of patients with cancer using TCGA database, and found that all eight proteins in the valine, leucine, and isoleucine degradation pathway were related to the survival rate of patients with different cancers such as COVD, KIRC, and KIRP (Table S11). For example, patients with lower HMGCS2 levels exhibit a reduced survival rate in COVD, KIRC, and liver hepatocellular carcinoma (LIHC). Similarly, lower HCDH levels were associated with decreased survival rates in KIRC, KIRP, thymoma (THYM), and uterine corpus endometrial carcinoma (UCEC). Additionally, diminished ALDH2 expression is associated with lower survival rates in skin cutaneous melanoma (SKCM), head and neck squamous cell carcinoma (HNSC), kidney chromophobe (KICH), KIRC, KIRP, brain low-grade glioma (LGG), LIHC, and mesothelioma (MESO). Therefore, we hypothesized that proteins involved in the BCAA pathway play crucial roles in the survival of patients with cancer and may serve as potential targets for anticancer drug development.

To validate the dysregulation of the BCAA pathway, both downregulated proteins (ALDH2 and HCDH), two key node proteins in the valine, leucine, and isoleucine degradation pathway, were selected for further studies. We validated the downregulation of ALDH2 and HCDH in mRNA and protein level in colon tumor tissues compared to adjacent normal tissues. These validation results indicated that proteomic findings were consistent with transcriptomic changes in colon cancer. Previous studies have reported that ALDH2 is expressed at lower levels in liver tumor tissues than in normal tissues and that its expression is negatively correlated with hepatocellular carcinoma progression 50, 51. ALDH2 deficiency accelerates gastric carcinogenesis 52. Additionally, differences in ALDH2 expression have been observed in other human diseases including cardiovascular diseases and diabetes 53. Moreover, decreased HCDH expression is associated with a poor prognosis and immune infiltration in KIRC 54. Therefore, ALDH2 and HCDH play crucial roles in the prognosis of patients with cancer and may provide crucial information for the development of immunotherapies for colon cancer. Our findings suggest that inactivation of proteins in the valine, leucine, and isoleucine degradation pathway reduces the metabolism of these amino acids, thereby promoting cancer cell growth and development. Elevated BCAA levels in tissues are likely to provide an additional source of nutrients to support cancer cell growth. This study provides new information for elucidating the mechanisms underlying colon cancer.

This study has a few limitations. First, only two proteins were validated in 21 samples, in the future, validation of these differential proteins in larger sample sizes are necessary; second, we did not detect the functions of differentially expressed proteins such as ALDH2 and HCDH in cell models or animal models by generating knockouts or performing gene-silencing experiments; third, we did not experimentally verify the relationship between the protein expression of ALDH2 or HCDH and the patho-clinical characteristics of patients (such as tumor staging, and patients survival); forth, we did not investigate the functional effects of altered BCAA pathway activity in colon cancer cells or animal models, and did not study the phenotypic impact of BCAA pathway on colon cancer. In the future, it is important to perform the following studies: 1) further clinical studies in large sample sizes to prove the value of ALDH2 and HCDH in clinical applications; 2) further functional studies in cell or animal models to understand the mechanism of ALDH2 and HCDH involved in colon cancer; 3) deep investigations of the functional effects of altered BCAA pathway activity in colon cancer cells or animal models to study the impact of BCAA pathway on the occurrence and development of colon cancer.

In conclusion, we performed a subcellular proteomic study in an AOM/DSS mouse model and found that proteins involved in the BCAA pathway were downregulated. Eight differentially expressed proteins in the valine, leucine, and isoleucine degradation pathway were related to the survival rate of patients with cancer. Lower ALDH2 or HCDH expression is related to poor prognosis in patients with cancers such as KIRC and KIRP. Our study may offer new clues to accelerate our understanding of tumor biology and provide new targets for immunotherapies for colon cancer.

## Supplementary Material

Supplementary figures and tables.

## Figures and Tables

**Table 1:** The differential proteins involved in the valine, leucine, and isoleucine degradation pathway.

Accession	Gene	Protein name	Cov (95%)	Peptides (matched)	Ratio (Adj/Nor)	Ratio (Can/Nor)	Ratio (Can/Adj)	Protein expression in CPTAC	Overall survival in COAD (*p* value)	Overall survival rate in cancer datasets*
P38060	*Hmgcl*	Hydroxymethylglutaryl-CoA lyase, mitochondrial	5.85	2	1.02	0.5	0.58	NA	0.072	Yes
P47738	*Aldh2*	Aldehyde dehydrogenase, mitochondrial	27.36	17	1.01	0.5	0.56	Down	0.65	Yes
P54869	*Hmgcs2*	Hydroxymethylglutaryl-CoA synthase, mitochondrial	27.17	20	1.01	0.32	0.28	Down	0.016	Yes
Q07417	*Acads*	Short-chain specific acyl-CoA dehydrogenase, mitochondrial	19.42	7	1	0.46	0.45	Down	0.24	Yes
Q61425	*Hadh*	Hydroxyacyl-coenzyme A dehydrogenase, mitochondrial	21.34	7	1.01	0.53	0.51	Down	0.25	Yes
Q8BH95	*Echs1*	Enoyl-CoA hydratase, mitochondrial	35.52	12	1	0.52	0.48	Down	0.99	Yes
Q8QZT1	*Acat1*	Acetyl-CoA acetyltransferase, mitochondrial	17.45	9	1	0.63	0.62	Down	0.91	Yes
Q9CZS1	*Aldh1b1*	Aldehyde dehydrogenase X, mitochondrial	27.17	14	1.04	0.33	0.44	Down	0.23	Yes

* 'Yes' indicates a relationship between protein expression and the overall survival rate of cancer patients in datasets reported from the TCGA database.
