# Supplementary material for: Branched-Chain Amino Acid Degradation Pathway was Inactivated in Colorectal Cancer: Results from a Proteomics Study
Source: J Cancer. 2024 May 20;15(12):3724–37. doi: 10.7150/jca.95454 (PMC11190764; doi:10.7150/jca.95454)
Supplement: Supplementary file 1 — Supplementary figures and tables. [file jcav15p3724s1.pdf]

Supplementary materials for the manuscript:

Branched-Chain Amino Acid Degradation Pathway was Inactivated in Colorectal Cancer: Results from a Proteomics Study

Shixian Lian[1*], Siyuan Liu[2*], Ao Wu[1*], Lin Yin[1], Lei Li[1], Liyan Zeng[1], Mingkun Zhao[1], Lijun Zhang[1#]

1. Shanghai Public Health Clinical Center, Fudan University, Shanghai 201508, China;

2. Shanghai Ninth People's Hospital, Shanghai Jiaotong University School of Medicine, Shanghai 200011, China.

[*] Shixian Lian, Siyuan Liu and Ao Wu equally contributed to this work.

#Correspondence to

Lijun Zhang: zhanglijun@shphc.org.cn

Key words: colon cancer, AOM/DSS, proteomics, ALDH2, branched chain amino acids

Figure S1. The images of each mouse and its colon against a ruler from AOM-DSS treated mice. The mice are numbered by #number.

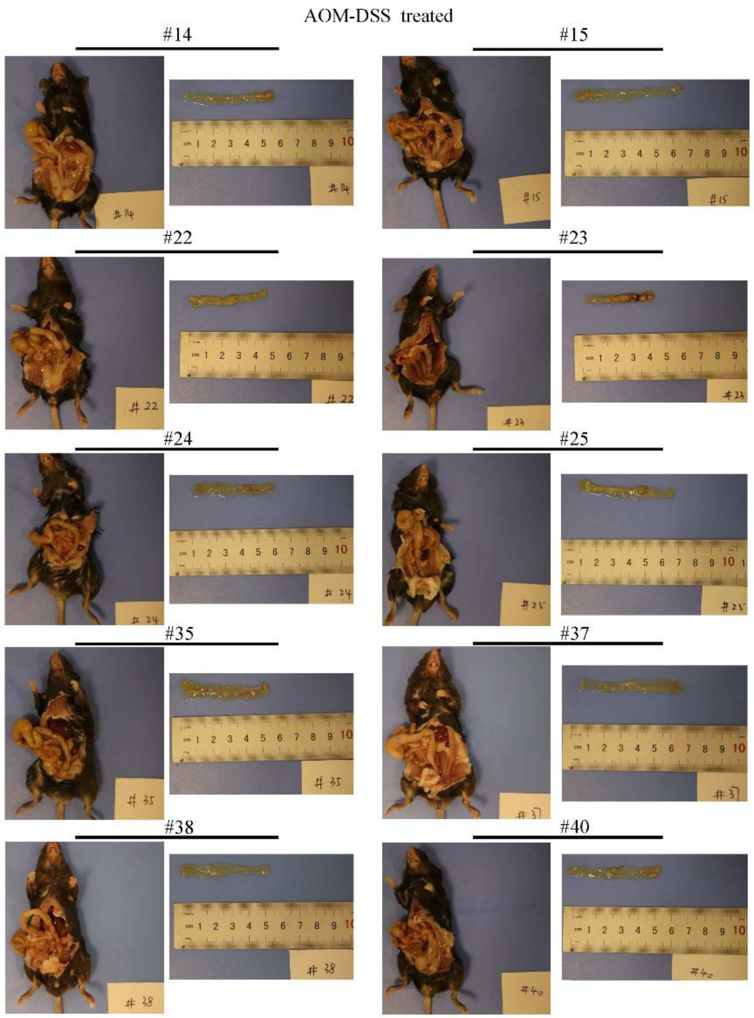

Figure S2. The images of each mouse and its colon against a ruler from saline treated mice. The mice are numbered by #number.

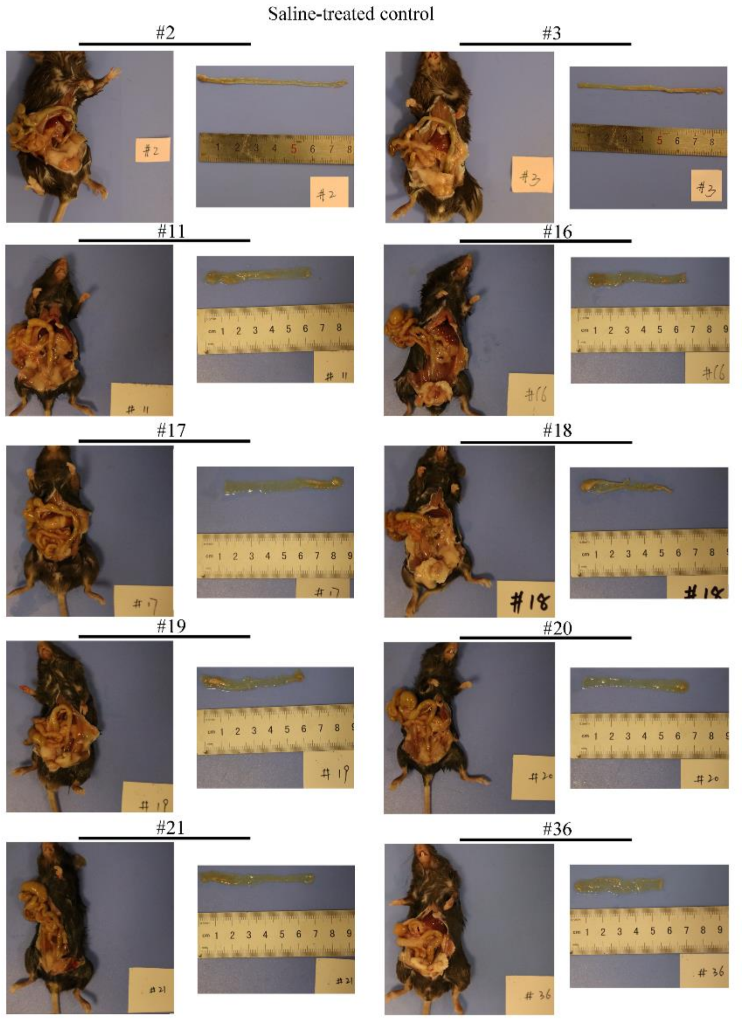

Figure S3. Bioinformatics analysis of the 74 differential proteins by STRING software

A. cellular component (CC), (B) molecular function (MF), and (C) biological process (BP).

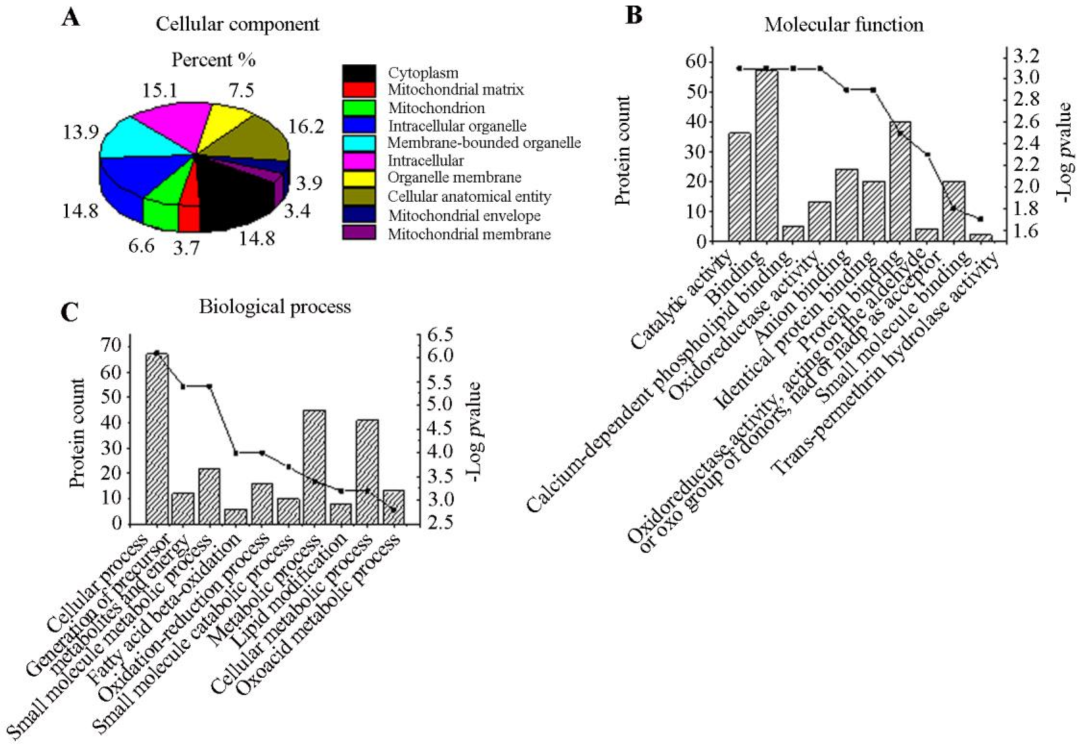

Table S1 The clinical characteristics of 21 patients with ulcerative colon cancer enrolled for RT-qPCR, western blot or metabolite quantification

Number	Sex	Age (years)	Tumor size (length*width (cm))	Differentiation	pTNM	Number of lymph nodes (transferred / harvested)	Metastasis (Yes/No)	Metastasis site	RT-qPCR[a]	WB[b]	Metabonomics[c]
1	F	72	4*4	II	pT3N0M0	0/30	No		Yes		
2	F	71	5*6	IV	pT3N1bM1c	3/39	Yes	Greater omentum	Yes		
3	F	68	4*5	II	pT2N0M0	0/23	No		Yes		
4	M	69	6*6	IV	pT4aN1bM1a	2/45	Yes	Liver	Yes	Yes	
5	F	77	7*8	IV	pT3N1aM1c	1/38	Yes	Mesentery	Yes	Yes	
6	F	45	6*7	II	pT3N1aM0	1/35	No		Yes	Yes	
7	F	44	4*4	II	pT2N0M0	0/30	No		Yes		
8	F	54	2*2	II	pT3N1aM0	1/37	No		Yes		
9	F	77	5*6	II	pT2N0M0	0/29	No		Yes		
10	M	84	6*6	II	pT3N0M0	0/39	No		Yes	Yes	
11	M	63	3*3	II	pT2N0M0	0/28	No		Yes		
12	M	57	5*5	IV	pT4aN1aM1a	1/43	Yes	Liver	Yes		
13	F	59	4*4	II	pT3N0M0	0/30	No		Yes	Yes	
14	M	70	4*4	II	pT2N0M0	0/26	No		Yes		
15	M	64	4*3	II	pT3N0M0	0/41	No				Yes
16	M	68	4*3	II	pT2N1aM0	1/20	No				Yes
17	F	42	8*5	III	pT4bN0M0	0/49	No				Yes
18	M	71	5*4	II	pT3N0M0	0/23	No				Yes
19	F	47	4*4	II	pT3N1aM0	1/42	No				Yes

20	M	68	6*5	II	pT3N0M0	0/30	No			Yes
21	M	48	8*7	II	pT3N0M0	0/36	No			Yes
Statistical analysis	11 F, 10 M	62.8±12.1					4	14	5	7

[a, b, c]: 14, 5 and 7 pairs of tumor and adjacent normal tissues from colon cancer patients were used for RT-qPCR, western blot (WB) detection, and metabolite quantification, respectively. F: female; M: male. pTNM: pathologic tumor node metastasis (T, tumor; N: node; M: metastasis).

Table S2. The optimized mass spectrometry parameters for valine, leucine and isoleucine detection

Name	Q1 Mass (Da)	Q3 Mass (Da)	DP (volts)	EP (volts)	CE (volts)	CXP (volts)
Valine	118.1	72.1	55.0	9.0	15.0	13.0
Leucine	132.1	86.1	39.0	13.0	12.0	10.0
Isoleucine	132.2	86.1	76.0	13.0	14.0	8.0
L-isoleucine-d10	142.0	96.2	66.0	10.0	15.0	13.0
L-Valine-d8	126.1	80.1	56.0	10.0	16.0	13.0

DP: declustering potential; EP: entrance potential; CE: collision energy; CXP: cell exit potential.

Q1 and Q3 represent precursor and fragment ions, respectively.

Table S3. The tumor account and size from AOM/DSS treated mice

Number	Items	Tumor 1	Tumor 2	Tumor 3	Tumor 4	Tumor 5	Tumor 6	Tumor 7	Tumor 8
#14	Length (mm)	2.38	2.22						
	Width (mm)	2.19	1.80						
	Area (mm^2)	5.21	4.00						
#15	Length (mm)	2.56	3.15						
	Width (mm)	2.45	3.14						
	Area (mm^2)	6.27	9.89						
#22	Length (mm)	3.43	3.71	4.56	3.32	2.21	2.56		
	Width (mm)	3.41	2.90	3.06	2.63	2.21	1.64		
	Area (mm^2)	11.70	10.76	13.95	8.73	4.88	4.20		
#23	Length (mm)	2.36	3.55	4.07	3.79	2.70	2.29	4.43	2.70
	Width (mm)	2.02	2.30	3.37	2.38	2.63	1.60	3.28	2.48
	Area (mm^2)	4.77	8.17	13.72	9.02	7.10	3.66	14.53	6.70
#24	Length (mm)	2.88	4.13	2.62	2.68	3.18			
	Width (mm)	2.81	3.35	2.24	2.68	3.18			
	Area (mm^2)	8.09	13.84	5.87	7.18	10.11			
#25	Length (mm)	3.15	5.05	2.31					
	Width (mm)	2.56	4.37	2.29					
	Area (mm^2)	8.06	22.07	5.29					
#35	Length (mm)	3.42	2.56						
	Width (mm)	2.41	2.55						
	Area (mm^2)	8.24	6.53						
#37	Length (mm)	3.22	3.26						
	Width (mm)	2.90	3.18						

	Area (mm²)	9.34	10.37		
	Length (mm)	2.23			
#38	Width (mm)	2.05			
	Area (mm²)	4.57			
	Length (mm)	4.34	2.69	2.35	3.24
#40	Width (mm)	3.54	2.22	1.80	2.43
	Area (mm²)	15.36	5.97	4.23	7.87

Table S4. The proteins identified and quantified in the experiment replicate 1

Accession	Unused	Total	%Cov(95)	Name	Peptides (95%)	116:114	117:114	114:116	117:116
A2A863	30.7	31.38	7.646	Integrin beta-4	13	1.12524	1.54791	0.88830	1.31991
A2AFS3	2.19	4.97	1.883	UPF0577 protein KIAA1324	3	0.86870	0.43885	1.15064	0.51491
A7L9Z8	2.08	3.95	1.801	Calcium-transporting ATPase type 2C member 2	2	0.94881	0.82197	1.05348	0.86747
B1AXP6	3.18	3.21	13.730	Mitochondrial import receptor subunit TOM5 homolog	1	0.98036	1.02866	1.01958	1.04660
B2RX12	26.5	29.12	7.223	Canalicular multispecific organic anion transporter 2	13	0.81237	0.44353	1.23042	0.54998
B2RXS4	31.1	32.49	7.980	Plexin-B2	15	0.98001	0.94012	1.01994	0.96443
D3Z6P0	2.05	4.56	3.226	Protein disulfide-isomerase A2	2	1.96369	0.92202	0.50902	0.50169
D3Z7P3	2.16	3.51	2.374	Glutaminase kidney isoform, mitochondrial	2	0.90745	0.60583	1.10149	0.66937
E9Q557	28.7	36.31	3.677	Desmoplakin	13	1.52715	1.56997	0.65452	1.02279
F7BWT7	3.2	3.32	6.803	Tetraspanin-15	2	1.45711	1.21103	0.68598	0.82575
O08528	8.2	9.37	4.035	Hexokinase-2	4	1.12154	0.90983	0.89123	0.81468
O08547	2.16	2.27	4.651	Vesicle-trafficking protein SEC22b	1	1.19060	1.00487	0.83953	0.85358
O08553	7.3	8.11	7.692	Dihydropyrimidinase-related protein 2	4	0.69082	0.80494	1.44690	1.17418
O08573	3.36	3.36	6.799	Galectin-9	2	1.01757	1.66360	0.98229	1.64094
O08585	3.35	3.47	6.809	Clathrin light chain A	2	1.03694	1.40046	0.96395	1.36439

O08638	26.3	48.42	8.976	Myosin-11	20	1.42866	1.97902	0.69964	1.34226
O08663	2.02	2.35	1.883	Methionine aminopeptidase 2	1	1.17129	1.98598	0.85338	1.69550
O08688	1.78	2.36	1.719	Calpain-5	1	0.64470	0.82435	1.55041	1.28216
O08709	17.5	18.22	37.500	Peroxiredoxin-6	9	0.57799	0.45228	1.72936	0.78520
O08734	2.5	2.55	4.808	Bcl-2 homologous antagonist/killer	1	1.34518	1.12909	0.74306	0.83092
O08749	11.4	11.74	10.810	Dihydrolipoyl dehydrogenase, mitochondrial	6	0.76874	0.50921	1.30024	0.67444
O08756	7.17	7.55	14.940	3-hydroxyacyl-CoA dehydrogenase type-2	5	0.85987	0.55534	1.16245	0.64381
O08788	4.66	7.82	2.654	Dynactin subunit 1	3	0.69424	0.50997	1.43977	0.73648
O08795	6.17	6.22	5.950	Glucosidase 2 subunit beta	4	1.79400	1.46754	0.55717	0.81157
O08807	2	4.96	10.580	Peroxiredoxin-4	3	1.43104	1.57321	0.69848	1.10224
O08832	2.41	3.02	1.903	Polypeptide N-acetylgalactosaminyltransferase 4	1	1.22861	0.67612	0.81356	0.55616
O08917	5.97	6.2	8.645	Flotillin-1	3	1.38481	3.52723	0.72180	2.54907
O08997	2	2	11.760	Copper transport protein ATOX1	1	0.68330	0.73718	1.46284	1.08168
O09044	2.95	3.05	10.000	Synaptosomal-associated protein 23	2	0.98549	1.43865	1.01427	1.46464
O09061	2.22	2.22	3.750	Proteasome subunit beta type-1	1	0.91163	0.90022	1.09644	0.99007
O09111	2	2	6.623	NADH dehydrogenase [ubiquinone] 1 beta subcomplex subunit 11, mitochondrial	1	1.14843	1.47474	0.87036	1.28750
O09117	3.16	3.25	5.747	Synaptophysin-like protein 1	2	1.06756	1.23386	0.93630	1.15139
O09131	6.87	7.31	13.330	Glutathione S-transferase omega-1	4	0.63071	0.48309	1.58480	0.76626
O35054	2	2	5.714	Claudin-4	1	1.38607	2.51854	0.72114	1.82180
O35114	2.97	3.32	4.184	Lysosome membrane protein 2	2	1.16804	1.76029	0.85575	1.51465
O35129	12.2	12.78	21.740	Prohibitin-2	7	1.37566	1.35001	0.72660	0.97780
O35143	2	2.03	7.547	ATPase inhibitor, mitochondrial	1	1.14602	0.42479	0.87220	0.37164
O35350	4.18	4.88	3.226	Calpain-1 catalytic subunit	2	0.93241	1.14294	1.07201	1.23049
O35405	2	2.03	2.049	Phospholipase D3	1	1.01401	1.71054	0.98574	1.69132

O35459	4.61	4.7	6.422	Delta(3,5)-Delta(2,4)-dienoyl-CoA isomerase, mitochondrial	2	0.97123	0.64910	1.02916	0.67186
O35566	3.08	3.36	5.138	CD151 antigen	2	1.15159	1.38431	0.86797	1.20329
O35593	3.71	4.02	7.097	26S proteasome non-ATPase regulatory subunit 14	2	0.86640	0.78251	1.15369	0.90515
O35598	4.22	4.58	2.937	Disintegrin and metalloproteinase domain-containing protein 10	2	1.10520	1.39264	0.90441	1.26676
O35604	2.03	2.51	0.548	Niemann-Pick C1 protein	1	1.18202	4.28901	0.84563	3.63806
O35609	2.02	2.03	4.585	Secretory carrier-associated membrane protein 3	1	0.74250	1.31140	1.34620	1.77084
O35639	16.9	21.31	34.670	Annexin A3	10	0.93025	1.34248	1.07450	1.44672
O35640	6.25	8.36	12.840	Annexin A8	4	1.15988	2.69126	0.86177	2.29849
O35643	6.2	22.44	12.200	AP-1 complex subunit beta-1	13	0.83322	1.15093	1.19963	1.38381
O35683	1.33	1.33	7.143	NADH dehydrogenase [ubiquinone] 1 alpha subcomplex subunit 1	1	1.36123	0.87915	0.73430	0.64832
O35704	1.44	1.61	1.691	Serine palmitoyltransferase 1	1	0.97259	0.95256	1.02772	0.98266
O35841	1.7	2.63	1.587	Apoptosis inhibitor 5	1	0.84296	1.63954	1.18577	1.95008
O35874	2.07	2.84	2.068	Neutral amino acid transporter A	1	1.11017	0.78495	0.90036	0.70908
O35887	2.14	2.28	5.714	Calumenin	2	1.36781	1.24522	0.73077	0.91270
O54724	1.52	4.44	3.827	Polymerase I and transcript release factor	2	1.60472	2.60916	0.62288	1.64774
O54734	11.1	12.89	15.650	Dolichyl-diphosphooligosaccharide--protein glycosyltransferase 48 kDa subunit	8	1.38451	0.93506	0.72195	0.69985
O54950	3.91	4.04	6.061	5'-AMP-activated protein kinase subunit gamma-1	2	0.83467	0.76368	1.19754	0.92086
O54988	2.23	4.75	1.379	STE20-like serine/threonine-protein kinase	3	1.07580	1.18836	0.92912	1.10752
O55013	4.38	4.78	12.780	Trafficking protein particle complex subunit 3	3	1.01657	1.09918	0.98326	1.08603
O55022	7.05	7.62	11.790	Membrane-associated progesterone receptor component 1	4	1.29583	1.24693	0.77136	0.96182
O55023	2	2.98	5.776	Inositol monophosphatase 1	2	0.79696	0.75042	1.25420	0.94406
O55111	11.5	12.59	6.506	Desmoglein-2	7	1.19325	1.08462	0.83767	0.91055

O55125	3.08	3.65	4.225	Protein NipSnap homolog 1	2	1.37804	1.06372	0.72534	0.77456
O55143	9.72	15.08	5.268	Sarcoplasmic/endoplasmic reticulum calcium ATPase 2	6	1.03116	1.10816	0.96934	1.08203
O70200	2.07	2.19	5.442	Allograft inflammatory factor 1	1	1.20667	4.35175	0.82836	3.61589
O70251	6.18	6.33	13.780	Elongation factor 1-beta	4	1.05308	0.84407	0.94917	0.80013
O70318	2.78	4.16	1.518	Band 4.1-like protein 2	2	0.28636	0.58235	3.49060	2.03899
O70400	9	9.63	11.620	PDZ and LIM domain protein 1	4	0.69490	0.83781	1.43840	1.21308
O70435	3.84	4.04	7.451	Proteasome subunit alpha type-3	2	1.16361	1.17205	0.85901	1.01215
O70439	4.82	4.9	12.640	Syntaxin-7	3	1.36789	2.09143	0.73073	1.48427
O70456	2.29	7.31	11.690	14-3-3 protein sigma	6	0.59586	0.78312	1.67750	1.31113
O70475	20.6	21.12	21.500	UDP-glucose 6-dehydrogenase	10	0.86884	0.45355	1.15045	0.51350
O70493	2	2.05	5.455	Sorting nexin-12	1	1.05676	1.75906	0.94586	1.66894
O70503	1.35	1.43	2.885	Estradiol 17-beta-dehydrogenase 12	1	1.56916	1.90837	0.63700	1.22136
O70570	14.7	14.89	9.987	Polymeric immunoglobulin receptor	7	1.33611	0.74870	0.74811	0.56089
O88312	16.2	16.2	33.140	Anterior gradient protein 2 homolog	15	1.10863	0.19845	0.90161	0.19317
O88329	16.7	18.18	6.999	Unconventional myosin-Ia	7	0.49811	0.47368	2.00669	0.95326
O88342	12.7	13.21	10.230	WD repeat-containing protein 1	6	0.72461	1.19985	1.37943	1.66695
O88343	4.06	4.52	1.761	Electrogenic sodium bicarbonate cotransporter 1	2	0.80305	0.41735	1.24469	0.51150
O88398	1.8	2.9	1.221	Advillin	1	0.81282	2.96020	1.22974	3.65146
O88428	1.57	2.1	2.093	Bifunctional 3'-phosphoadenosine 5'-phosphosulfate synthase 2	1	1.11421	0.41788	0.89709	0.37603
O88569	5.26	5.76	6.516	Heterogeneous nuclear ribonucleoproteins A2/B1	3	0.52830	0.37021	1.89203	0.69340
O88587	1.9	2.38	3.396	Catechol O-methyltransferase	1	1.22297	1.66516	0.81731	1.38118
O88593	2.31	2.36	6.593	Peptidoglycan recognition protein 1	1	1.22004	2.45434	0.81928	2.01616
O88685	4.63	8.2	8.824	26S protease regulatory subunit 6A	5	0.97112	0.78973	1.02928	0.81939
O88792	3.97	4.04	7.667	Junctional adhesion molecule A	2	1.06877	1.24099	0.93524	1.18211
O88844	16.7	17.21	20.050	Isocitrate dehydrogenase [NADP] cytoplasmic	8	0.94726	1.28099	1.05520	1.35524

O88952	12.5	13.8	25.380	Protein lin-7 homolog C	7	0.80889	1.07259	1.23571	1.32999
O88983	1.94	3.18	3.390	Syntaxin-8	1	1.06901	2.47479	0.93503	2.32110
O89001	18.6	21.34	4.575	Carboxypeptidase D	8	0.82091	0.58116	1.21761	0.71459
O89053	5.58	5.81	6.941	Coronin-1A	4	1.76682	4.48961	0.56574	2.56413
O89079	8.05	10.14	20.130	Coatomer subunit epsilon	6	0.99541	0.87612	1.00416	0.88105
P00329	8.86	9.01	10.670	Alcohol dehydrogenase 1	4	0.68561	0.77924	1.45791	1.13881
P00405	3.52	4.51	7.489	Cytochrome c oxidase subunit 2	2	1.07299	0.62726	0.93156	0.58603
P00920	2.74	2.85	3.462	Carbonic anhydrase 2	1	0.47918	0.25455	2.08598	0.51080
P01592	2.99	3.04	16.350	Immunoglobulin J chain	2	1.48703	1.25832	0.67218	0.84772
P01831	4.04	4.04	14.810	Thy-1 membrane glycoprotein	2	1.19931	4.81437	0.83344	4.08130
P01837	1.33	2.37	17.920	Ig kappa chain C region	2	1.50095	1.34289	0.66594	0.89545
P01878	4	4.33	5.814	Ig alpha chain C region	2	1.44137	1.21078	0.69348	0.84224
P01899	16.4	16.53	26.240	H-2 class I histocompatibility antigen, D-B alpha chain	8	0.91277	1.07243	1.09508	1.17764
P01901	3.54	12.25	11.380	H-2 class I histocompatibility antigen, K-B alpha chain	5	0.98141	1.10114	1.01848	1.12618
P01902	2.02	4.81	5.435	H-2 class I histocompatibility antigen, K-D alpha chain	2	0.69418	0.18212	1.43991	0.26304
P01942	9.93	9.98	33.800	Hemoglobin subunit alpha	6	0.64590	0.45569	1.54753	0.71725
P02088	14.1	14.05	59.180	Hemoglobin subunit beta-1	23	0.73970	0.44733	1.35129	0.61175
P02301	8.35	9.31	19.120	Histone H3.3C	4	1.12484	2.81918	0.88861	2.44939
P02535	6.04	10	9.649	Keratin, type I cytoskeletal 10	6	0.26063	0.59905	3.83508	2.30028
P03930	3.17	3.17	28.360	ATP synthase protein 8	2	1.00648	0.94785	0.99311	0.95098
P04441	1.82	1.92	5.018	H-2 class II histocompatibility antigen gamma chain	1	0.91416	1.42464	1.09341	1.56251
P04919	2	3.3	2.153	Band 3 anion transport protein	2	1.76495	3.40023	0.56634	1.93159
P05064	23.2	23.86	35.160	Fructose-bisphosphate aldolase A	15	0.90836	0.97154	1.10039	1.07424
P05201	1.57	2.12	3.148	Aspartate aminotransferase, cytoplasmic	1	0.80849	0.96396	1.23632	1.19542
P05202	14.6	14.57	17.210	Aspartate aminotransferase, mitochondrial	7	1.40705	0.87238	0.71039	0.62525
P05366	2.22	2.22	10.660	Serum amyloid A-1 protein	1	0.95622	0.64286	1.04532	0.67476

P05555	8.7	9.8	4.163	Integrin alpha-M	5	1.15931	2.64526	0.86220	2.69584
P05784	30	36.54	42.550	Keratin, type I cytoskeletal 18	28	1.48309	0.85177	0.67397	0.57352
P06151	17.3	18.24	22.590	L-lactate dehydrogenase A chain	9	0.68812	0.71054	1.45258	1.01077
P06745	6.63	6.83	4.839	Glucose-6-phosphate isomerase	3	0.57095	1.35147	1.75069	2.37138
P06800	4.31	4.87	2.169	Receptor-type tyrosine-protein phosphatase C	3	1.25563	3.33350	0.79606	2.68427
P07356	42.5	43.87	52.510	Annexin A2	42	1.08786	2.46310	0.91882	2.25692
P07724	26.2	27.42	18.750	Serum albumin	14	1.17571	1.42885	0.85017	1.21447
P07758	5.02	6.31	7.264	Alpha-1-antitrypsin 1-1	4	1.23187	1.02403	0.81141	0.82666
P07759	2.01	2.33	2.871	Serine protease inhibitor A3K	1	1.00803	0.92524	0.99158	0.92027
P07901	5.32	27.35	16.920	Heat shock protein HSP 90-alpha	16	1.15366	1.56802	0.86642	1.37094
P08003	29.8	33.08	22.100	Protein disulfide-isomerase A4	19	1.16315	1.06441	0.85935	0.91767
P08030	2.04	2.11	5.556	Adenine phosphoribosyltransferase	1	1.17959	1.90426	0.84737	1.61857
P08071	2.9	3.05	1.132	Lactotransferrin	1	1.61614	3.60854	0.61848	2.37428
P08113	33.3	40.01	21.820	Endoplasmin	20	1.73855	1.25227	0.57494	0.72171
P08207	13.8	13.75	34.020	Protein S100-A10	11	0.94443	1.51241	1.05836	1.57952
P08228	5.2	5.2	23.380	Superoxide dismutase [Cu-Zn]	3	0.84266	0.78290	1.18618	0.92862
P08249	33.5	34.01	54.730	Malate dehydrogenase, mitochondrial	23	1.03751	0.41122	0.96342	0.40306
P08752	13.9	16.06	23.380	Guanine nucleotide-binding protein G(i) subunit alpha-2	10	0.95104	1.44088	1.05101	1.51600
P09055	27.3	28.96	16.420	Integrin beta-1	14	0.97255	1.47505	1.02776	1.47832
P09103	45.1	46.97	38.510	Protein disulfide-isomerase	26	1.30194	0.81013	0.76774	0.62370
P09405	17.5	19.59	15.420	Nucleolin	12	1.70563	1.84687	0.58603	1.11190
P09411	14.5	17.22	17.510	Phosphoglycerate kinase 1	8	0.73164	0.89869	1.36618	1.23417
P09671	4.12	4.29	9.910	Superoxide dismutase [Mn], mitochondrial	3	0.89729	0.66839	1.11396	0.74122
P09803	14.3	15.27	8.371	Cadherin-1	6	1.24960	1.46564	0.79990	1.15894
P10107	33.8	36.71	55.200	Annexin A1	22	1.41059	6.21191	0.70861	4.64563
P10126	26.3	26.9	31.600	Elongation factor 1-alpha 1	17	1.18688	1.91104	0.84217	1.60661

P10404	6.88	7.2	5.304	MLV-related proviral Env polyprotein	3	1.23092	2.95109	0.81204	2.40297
P10639	5.13	5.51	29.520	Thioredoxin	5	0.60124	0.43468	1.66249	0.71942
P10649	11.5	11.76	26.610	Glutathione S-transferase Mu 1	6	0.72119	0.61558	1.38597	0.86209
P10852	8.65	9.01	7.224	4F2 cell-surface antigen heavy chain	4	1.01699	1.41363	0.98285	1.39613
P11031	2.02	2.1	8.661	Activated RNA polymerase II transcriptional coactivator p15	1	1.30615	1.70278	0.76527	1.30708
P11352	2	2.17	5.473	Glutathione peroxidase 1	1	0.73251	0.94675	1.36456	1.29587
P11438	5.71	6	7.635	Lysosome-associated membrane glycoprotein 1	4	0.84872	1.41697	1.17771	1.67691
P11499	43.5	44.19	27.210	Heat shock protein HSP 90-beta	25	1.19965	1.74709	0.83320	1.45793
P11672	1.53	1.74	7.000	Neutrophil gelatinase-associated lipocalin	2	1.06262	2.45078	0.94065	2.31242
P11679	72.2	72.57	65.710	Keratin, type II cytoskeletal 8	95	1.77567	0.39984	0.56292	0.22852
P11688	3.34	3.89	2.279	Integrin alpha-5	2	1.40455	1.76875	0.71165	1.28837
P11835	11	11.78	8.690	Integrin beta-2	5	1.76364	5.20952	0.56675	3.11299
P11983	11.5	15.81	16.550	T-complex protein 1 subunit alpha	8	1.12445	1.41598	0.88892	1.26309
P12367	6.32	7	7.980	cAMP-dependent protein kinase type II-alpha regulatory subunit	3	0.87402	0.72175	1.14363	0.82774
P12382	2.16	4.36	2.179	6-phosphofructokinase, liver type	2	0.63292	0.56058	1.57927	0.88775
P12787	11.1	11.12	42.470	Cytochrome c oxidase subunit 5A, mitochondrial	10	1.41752	0.67953	0.70514	0.52274
P12815	2.72	3.15	10.990	Programmed cell death protein 6	2	0.88194	1.03806	1.13336	1.17634
P12970	1.45	1.91	3.008	60S ribosomal protein L7a	1	1.03607	1.24736	0.96476	1.20713
P13020	11.2	13.48	9.744	Gelsolin	6	0.69033	0.75262	1.44794	1.10757
P13634	14.1	14.39	33.720	Carbonic anhydrase 1	15	0.62077	0.17328	1.61017	0.23330
P13707	1.41	3.06	4.298	Glycerol-3-phosphate dehydrogenase [NAD(+)], cytoplasmic	2	1.03843	0.71965	0.96256	0.69483
P13808	7.21	10.71	3.476	Anion exchange protein 2	5	0.85727	0.57401	1.16597	0.67510
P14069	3.44	3.93	24.720	Protein S100-A6	3	0.95142	1.17664	1.05059	1.21387

P14094	15	15.74	27.300	Sodium/potassium-transporting ATPase subunit beta-1	24	0.64909	0.37584	1.53992	0.57723
P14115	2.44	2.75	7.432	60S ribosomal protein L27a	1	0.78638	1.39760	1.27109	1.77495
P14131	6.71	6.9	17.810	40S ribosomal protein S16	3	0.86239	1.65198	1.15905	1.93622
P14148	3.16	3.61	6.296	60S ribosomal protein L7	2	0.75634	1.35050	1.32156	1.82782
P14152	13.4	15.2	21.260	Malate dehydrogenase, cytoplasmic	7	0.67415	0.74736	1.48268	1.10997
P14206	9.72	10.43	20.000	40S ribosomal protein SA	6	1.03957	1.14369	0.96151	1.10529
P14211	21.2	21.63	23.560	Calreticulin	13	1.91746	1.41840	0.52129	0.75377
P14438	2.64	2.64	6.608	H-2 class II histocompatibility antigen, A-U alpha chain (Fragment)	1	0.65781	1.84710	1.51952	2.81289
P14733	9.6	12.46	9.864	Lamin-B1	6	1.36093	2.28177	0.73446	1.67927
P14824	46.8	48.35	30.910	Annexin A6	23	1.25095	5.39976	0.79904	4.38766
P14869	20.8	20.98	37.540	60S acidic ribosomal protein P0	12	1.35669	1.27501	0.78202	0.99421
P15105	1.58	1.64	2.145	Glutamine synthetase	1	1.19184	1.55251	0.83866	1.30482
P15208	2.8	4.72	1.093	Insulin receptor	2	0.86965	0.80493	1.14937	0.92943
P15532	4.24	9.54	26.320	Nucleoside diphosphate kinase A	4	1.03589	1.17388	0.96492	1.13599
P15535	2.06	2.16	2.506	Beta-1,4-galactosyltransferase 1	1	1.74317	1.48963	0.57341	0.85680
P15626	2.23	4.49	12.840	Glutathione S-transferase Mu 2	3	0.64682	0.39794	1.54534	0.61684
P15864	8.25	8.45	18.400	Histone H1.2	4	0.83780	1.46822	1.19307	1.76100
P16045	5.11	5.26	17.780	Galectin-1	3	0.80804	6.67265	1.23701	8.30991
P16110	15.6	15.63	32.580	Galectin-3	20	0.64910	0.67755	1.53990	1.01126
P16332	1.3	1.75	1.070	Methylmalonyl-CoA mutase, mitochondrial	1	0.76327	0.38782	1.30956	0.50944
P16546	90.5	92.28	19.170	Spectrin alpha chain, non-erythrocytic 1	47	1.17356	1.29254	0.85173	1.10196
P16858	27.7	29.93	40.240	Glyceraldehyde-3-phosphate dehydrogenase	17	0.98452	1.81452	1.01527	1.81206
P17047	8.98	9.06	12.050	Lysosome-associated membrane glycoprotein 2	6	1.03658	5.05204	0.96428	4.83860
P17182	24.7	25.29	27.650	Alpha-enolase	14	0.86221	1.00427	1.15930	1.17095
P17427	8.31	9.65	4.264	AP-2 complex subunit alpha-2	4	0.93755	2.14744	1.06614	2.30740

P17439	5.96	7.9	6.408	Glucosylceramidase	4	1.12867	1.84300	0.88560	1.63335
P17563	18.9	19.27	20.970	Selenium-binding protein 1	9	0.49520	0.21031	2.01846	0.40811
P17665	4.01	4.04	28.570	Cytochrome c oxidase subunit 7C, mitochondrial	3	0.91638	0.61450	1.09076	0.66855
P17710	2.74	7.46	2.977	Hexokinase-1	3	1.43801	1.44185	0.69510	1.00761
P17742	15.2	17.99	48.780	Peptidyl-prolyl cis-trans isomerase A	11	0.72214	1.75107	1.38416	2.39226
P17751	14.5	14.63	31.100	Triosephosphate isomerase	8	0.75908	0.81802	1.31679	1.07761
P18242	9.98	10.43	11.220	Cathepsin D	8	0.98857	2.19120	1.01111	2.25050
P18572	14.2	14.55	17.220	Basigin	10	0.88239	0.47814	1.13278	0.53205
P18653	4.5	8.6	3.315	Ribosomal protein S6 kinase alpha-1	3	0.85973	1.38946	1.16263	1.62217
P18760	20.8	21	50.000	Cofilin-1	11	0.80407	1.53667	1.24312	1.90915
P19001	62.8	62.86	73.200	Keratin, type I cytoskeletal 19	70	1.83744	0.47604	0.54399	0.27266
P19096	9.37	13.87	1.917	Fatty acid synthase	6	0.83958	1.23127	1.19054	1.46658
P19157	5.52	5.78	20.480	Glutathione S-transferase P 1	3	0.63451	0.82563	1.57530	1.30527
P19253	4.24	4.31	7.389	60S ribosomal protein L13a	2	0.85787	1.12917	1.16516	1.32460
P19324	4.55	4.83	5.995	Serpin H1	2	1.30468	3.03426	0.76613	2.62709
P19467	10.5	10.91	12.040	Mucin-13	6	0.56426	0.77569	1.77142	1.37395
P19536	9.12	9.12	44.530	Cytochrome c oxidase subunit 5B, mitochondrial	12	1.30097	0.58290	0.76831	0.45220
P19783	14.1	14.5	38.460	Cytochrome c oxidase subunit 4 isoform 1, mitochondrial	9	1.12372	0.69222	0.88950	0.60028
P20029	55.6	56.35	41.980	78 kDa glucose-regulated protein	39	1.51665	0.93060	0.65905	0.62113
P20060	2.76	3.1	1.493	Beta-hexosaminidase subunit beta	1	1.29441	1.51200	0.77221	1.17311
P20108	7.94	8.35	16.340	Thioredoxin-dependent peroxide reductase, mitochondrial	5	0.45067	0.44510	2.21791	0.99370
P20152	15.8	29.54	24.680	Vimentin	19	2.24581	2.81003	0.44507	1.35248
P20352	2.37	2.41	3.061	Tissue factor	1	1.04128	2.14198	0.95993	2.08302
P21107	10.5	11.95	12.630	Tropomyosin alpha-3 chain	6	0.91878	1.06776	1.08791	1.17031
P21278	15.5	17.35	23.680	Guanine nucleotide-binding protein subunit alpha-11	11	0.55071	0.47820	1.81502	0.87024

P21279	4.86	15.57	21.730	Guanine nucleotide-binding protein G(q) subunit alpha	9	0.80981	1.24779	1.23430	1.54953
P21447	49.3	50.06	19.040	Multidrug resistance protein 1A	27	0.49499	0.26560	2.01934	0.53899
P22892	5.25	5.39	2.190	AP-1 complex subunit gamma-1	2	0.99246	1.26496	1.00715	1.27792
P23591	1.83	2.31	3.738	GDP-L-fucose synthase	1	0.70104	0.79511	1.42581	1.13716
P24270	5.52	6.12	3.985	Catalase	3	1.24308	1.11857	0.80409	0.88972
P24369	18.8	18.91	40.280	Peptidyl-prolyl cis-trans isomerase B	12	1.34445	1.28306	0.74346	0.96033
P24452	6.49	6.52	9.659	Macrophage-capping protein	3	1.40156	3.62778	0.71317	2.62991
P24472	2.18	2.31	4.054	Glutathione S-transferase A4	1	0.87254	1.11420	1.14557	1.27361
P24527	1.45	1.85	0.982	Leukotriene A-4 hydrolase	1	0.76525	1.41050	1.30617	1.85183
P24549	3.61	6.16	4.790	Retinal dehydrogenase 1	3	0.77670	0.49898	1.28693	0.64525
P24668	6.67	6.83	14.030	Cation-dependent mannose-6-phosphate receptor	4	1.10360	1.53666	0.90572	1.39993
P25444	8.99	9.29	18.430	40S ribosomal protein S2	6	1.08883	1.82402	0.91801	1.73643
P26039	29.4	33.19	4.998	Talin-1	12	1.12603	1.80366	0.88767	1.62436
P26040	33	33.97	23.210	Ezrin	20	0.53262	0.78922	1.87668	1.47589
P26041	5.95	17.26	13.520	Moesin	10	1.23582	5.17679	0.80882	4.84258
P26150	4.87	5.49	10.460	3 beta-hydroxysteroid dehydrogenase/Delta 5-->4-isomerase type 3	4	1.40794	0.59699	0.70994	0.42513
P26231	66.6	67.54	36.310	Catenin alpha-1	48	1.01242	1.05144	0.98729	1.03827
P26339	2.07	2.14	2.376	Chromogranin-A	1	0.95617	0.59125	1.04537	0.61997
P26369	2.1	2.44	2.105	Splicing factor U2AF 65 kDa subunit	1	0.87669	0.66699	1.14014	0.76279
P26443	13.2	13.63	12.010	Glutamate dehydrogenase 1, mitochondrial	6	1.05453	0.92490	0.94786	0.87479
P27546	1.86	2.34	0.800	Microtubule-associated protein 4	1	0.73487	1.13465	1.36017	1.54805
P27601	9.82	14.01	15.650	Guanine nucleotide-binding protein subunit alpha-13	7	0.66888	0.95590	1.49437	1.40419
P27773	55.1	55.59	49.310	Protein disulfide-isomerase A3	34	1.37276	0.74335	0.72813	0.54416
P28063	2.15	4.13	4.348	Proteasome subunit beta type-8	2	0.87181	1.22702	1.14652	1.42893
P28076	1.68	1.87	4.110	Proteasome subunit beta type-9	1	0.65242	0.65524	1.53206	1.00695

P28667	2.03	2.07	7.500	MARCKS-related protein	1	1.18881	6.14787	0.84080	5.18502
P28825	5.35	7.45	5.355	Meprin A subunit alpha	5	0.57356	0.29018	1.74270	0.50735
P29341	6.01	6.5	6.447	Polyadenylate-binding protein 1	4	1.16748	1.22622	0.85616	1.05219
P29452	3.35	4.72	5.970	Caspase-1	3	0.83609	0.59901	1.19551	0.71274
P29595	2.67	2.91	13.580	NEDD8	1	0.70371	1.21077	1.42040	1.72505
P29758	3.25	4.77	5.011	Ornithine aminotransferase, mitochondrial	3	1.25032	1.08501	0.79944	0.86677
P30115	3.38	3.58	7.240	Glutathione S-transferase A3	2	1.21158	1.61903	0.82500	1.33976
P30275	24.1	26.58	30.860	Creatine kinase U-type, mitochondrial	14	0.87717	0.36152	1.13952	0.42955
P30416	3.22	3.44	4.148	Peptidyl-prolyl cis-trans isomerase FKBP4	2	0.88912	1.49179	1.12420	1.68627
P30999	41	41.46	20.790	Catenin delta-1	22	1.00000	1.26461	0.99955	1.26444
P31001	25	32.32	30.920	Desmin	19	2.13575	3.84557	0.46801	1.87931
P31230	2	2.42	3.871	Aminoacyl tRNA synthase complex-interacting multifunctional protein 1	1	1.05256	1.30921	0.94964	1.24710
P31428	7.52	7.64	9.268	Dipeptidase 1	5	0.41179	0.21584	2.42733	0.52671
P31725	12.9	12.88	39.820	Protein S100-A9	10	2.78792	9.03594	0.35853	3.25854
P31809	7.65	8.27	9.981	Carcinoembryonic antigen-related cell adhesion molecule 1	9	0.48928	0.25251	2.04288	0.52322
P31938	3.75	3.93	5.344	Dual specificity mitogen-activated protein kinase kinase 1	2	0.85906	1.46840	1.16354	1.73003
P32020	6.87	7.34	5.484	Non-specific lipid-transfer protein	6	1.50319	1.00412	0.66496	0.67046
P32883	4.24	5.32	17.460	GTPase KRas	3	0.78162	0.86187	1.27883	1.10792
P32921	1.58	1.73	3.534	Tryptophan--tRNA ligase, cytoplasmic	2	1.07048	1.29545	0.93374	1.23725
P34022	2	2.32	5.419	Ran-specific GTPase-activating protein	1	0.66178	1.01993	1.51041	1.54524
P35276	7.08	9.23	21.920	Ras-related protein Rab-3D	7	1.13041	0.75133	0.88424	0.66687
P35278	4.71	9.14	22.220	Ras-related protein Rab-5C	5	0.88966	0.66301	1.12352	0.74728
P35279	12.3	13.9	35.100	Ras-related protein Rab-6A	8	1.02699	0.67308	0.97328	0.65489

P35282	4.02	5.44	15.770	Ras-related protein Rab-21	3	0.86011	0.96824	1.16212	1.12867
P35293	8.33	10.15	28.640	Ras-related protein Rab-18	6	0.96236	0.79731	1.03865	0.83395
P35486	11.6	11.98	16.920	Pyruvate dehydrogenase E1 component subunit alpha, somatic form, mitochondrial	7	1.01170	0.52115	0.98800	0.51851
P35550	1.85	2.69	3.364	rRNA 2'-O-methyltransferase fibrillarin	1	1.94810	3.11765	0.51309	1.60455
P35564	17.8	18.16	14.040	Calnexin	9	2.21219	1.90866	0.45184	0.82496
P35700	16.8	17.01	36.180	Peroxiredoxin-1	13	0.88058	1.28993	1.13510	1.46874
P35979	8.46	9	29.090	60S ribosomal protein L12	4	1.12448	1.23562	0.88890	1.10589
P35980	2.85	3.4	6.915	60S ribosomal protein L18	1	0.36995	1.44894	2.70184	3.92685
P37040	3.18	4.56	2.065	NADPH--cytochrome P450 reductase	2	1.27906	0.85437	0.78147	0.67582
P37804	17.1	17.93	45.270	Transgelin	12	1.24368	1.75401	0.80371	1.41416
P38060	5.51	5.51	5.846	Hydroxymethylglutaryl-CoA lyase, mitochondrial	2	0.97220	0.47817	1.02813	0.49233
P38647	30.2	32.68	27.840	Stress-70 protein, mitochondrial	16	0.88242	0.57282	1.13273	0.65054
P39054	4.84	7.35	4.253	Dynamin-2	4	1.06916	1.30068	0.93489	1.21746
P40124	16	16.54	17.720	Adenylyl cyclase-associated protein 1	9	1.02100	1.12026	0.97899	1.10907
P40142	22.1	22.64	18.620	Transketolase	11	0.84318	1.28931	1.18545	1.53366
P40237	2.6	2.6	4.511	CD82 antigen	1	0.67900	0.65688	1.47210	0.96958
P40240	2.27	2.27	3.097	CD9 antigen	2	0.69205	1.34043	1.44433	1.92933
P41105	2	2.06	5.109	60S ribosomal protein L28	1	0.66515	1.47057	1.50275	2.21669
P41731	4.12	4.23	7.563	CD63 antigen	3	0.83368	1.23989	1.19897	1.49279
P42125	10.1	10.53	20.760	Enoyl-CoA delta isomerase 1, mitochondrial	5	1.13071	0.57510	0.88400	0.51116
P42932	21.7	25.21	21.530	T-complex protein 1 subunit theta	12	0.90701	1.09529	1.10203	1.21666
P43406	19.9	24.12	9.770	Integrin alpha-V	11	1.01677	1.85784	0.98307	1.83784
P45376	4.55	5.12	9.810	Aldose reductase	3	0.71045	0.74799	1.40693	1.05692
P45878	1.79	2.98	12.860	Peptidyl-prolyl cis-trans isomerase FKBP2	2	1.44643	0.73513	0.69105	0.50957

P45952	12.3	14.02	15.680	Medium-chain specific acyl-CoA dehydrogenase, mitochondrial	6	0.99016	0.65363	1.00949	0.66435
P46460	17.9	19.04	13.440	Vesicle-fusing ATPase	9	1.01892	1.37369	0.98099	1.35860
P46471	3.43	7.83	7.159	26S protease regulatory subunit 7	4	0.82880	0.72897	1.20603	0.88276
P46638	14.9	14.94	29.360	Ras-related protein Rab-11B	7	0.92970	0.70214	1.07514	0.76834
P46735	1.33	2.6	0.903	Unconventional myosin-Ib	1	0.77988	1.28226	1.28168	1.64849
P46935	2.15	3.08	1.015	E3 ubiquitin-protein ligase NEDD4	1	1.24758	2.45145	0.80119	1.97011
P46978	2.72	2.95	2.553	Dolichyl-diphosphooligosaccharide--protein glycosyltransferase subunit STT3A	2	1.37354	1.13486	0.72772	0.82535
P47738	27.6	28.54	27.360	Aldehyde dehydrogenase, mitochondrial	17	0.86067	0.42080	1.16137	0.48690
P47753	4.42	4.55	8.741	F-actin-capping protein subunit alpha-1	5	0.82787	0.87911	1.20738	1.06957
P47754	2.02	4.48	8.741	F-actin-capping protein subunit alpha-2	5	0.87084	0.86326	1.14780	0.99390
P47757	4.47	4.7	6.498	F-actin-capping protein subunit beta	2	0.69976	0.83356	1.42843	1.19140
P47758	4.33	6.5	10.040	Signal recognition particle receptor subunit beta	3	1.92253	1.52563	0.51991	0.79552
P47791	2.93	2.99	4.000	Glutathione reductase, mitochondrial	2	0.79342	0.82637	1.25980	1.04839
P47856	24.4	25.34	17.220	Glutamine--fructose-6-phosphate aminotransferase [isomerizing] 1	11	1.00322	0.65357	0.99635	0.64897
P47955	3.29	3.56	19.300	60S acidic ribosomal protein P1	2	1.11814	0.82651	0.89394	0.74113
P47963	2.97	3.2	7.109	60S ribosomal protein L13	2	0.98351	1.83444	1.01632	1.84256
P48036	25.1	27.2	34.170	Annexin A5	14	1.20897	2.89778	0.82678	2.41876
P48678	21.9	22.63	15.790	Prelamin-A/C	11	1.25144	1.94291	0.79872	1.54586
P48758	8.91	9.1	9.025	Carbonyl reductase [NADPH] 1	3	0.88384	0.46922	1.13092	0.53260
P48771	2.68	2.79	15.660	Cytochrome c oxidase subunit 7A2, mitochondrial	2	1.05083	0.74456	0.95120	0.71040
P49312	4.04	4.72	7.187	Heterogeneous nuclear ribonucleoprotein A1	2	1.03455	0.79029	0.96617	0.75825
P49817	2.31	2.31	3.933	Caveolin-1	1	2.21907	5.02095	0.45044	2.27113
P50171	2.03	2.17	4.633	Estradiol 17-beta-dehydrogenase 8	1	0.98052	0.38212	1.01941	0.39046

P50247	1.73	1.92	2.778	Adenosylhomocysteinase	1	0.69678	0.98544	1.43454	1.41697
P50516	13.7	13.89	11.350	V-type proton ATPase catalytic subunit A	6	0.91145	0.91984	1.09666	1.02316
P50518	3.98	4.31	5.752	V-type proton ATPase subunit E 1	1	0.92408	0.95223	1.08167	1.03554
P50543	2.1	2.61	19.390	Protein S100-A11	2	1.76109	5.49055	0.56758	3.17904
P50544	22.2	23.34	15.850	Very long-chain specific acyl-CoA dehydrogenase, mitochondrial	10	1.25517	0.83032	0.79635	0.65873
P50580	2.53	2.65	2.030	Proliferation-associated protein 2G4	1	0.91045	1.36475	1.09787	1.50997
P51125	3.06	3.37	5.330	Calpastatin	2	0.89591	0.81853	1.11568	0.91377
P51150	16.6	16.85	48.310	Ras-related protein Rab-7a	10	0.87181	1.06086	1.14652	1.22019
P51174	8.5	8.86	10.700	Long-chain specific acyl-CoA dehydrogenase, mitochondrial	4	0.86847	0.72504	1.15094	0.83653
P51432	9.47	9.96	3.728	1-phosphatidylinositol 4,5-bisphosphate phosphodiesterase beta-3	5	0.72362	0.94129	1.38131	1.30917
P51437	3.34	3.83	12.720	Cathelin-related antimicrobial peptide	2	1.58392	4.60076	0.63106	2.96829
P51655	1.89	3.07	1.257	Glypican-4	1	1.14713	1.87718	0.87135	1.64072
P51658	5.54	6.21	8.136	Estradiol 17-beta-dehydrogenase 2	3	4.93287	0.86718	0.20263	0.23413
P51660	8.79	9.13	7.483	Peroxisomal multifunctional enzyme type 2	5	1.18970	0.92542	0.84017	0.77060
P51661	9.63	9.63	14.250	Corticosteroid 11-beta-dehydrogenase isozyme 2	6	1.75964	1.10161	0.56804	0.63281
P51855	2.12	2.38	3.376	Glutathione synthetase	2	0.74782	0.78246	1.33662	1.04609
P51863	6.06	6.12	8.262	V-type proton ATPase subunit d 1	3	0.84089	1.12695	1.18869	1.34544
P51881	21.5	21.47	29.190	ADP/ATP translocase 2	11	1.27170	1.41431	0.78599	1.10858
P51912	4.86	5.34	4.521	Neutral amino acid transporter B(0)	2	1.02851	0.92985	0.97184	0.90260
P52196	13.9	13.91	26.260	Thiosulfate sulfurtransferase	7	0.95828	0.57399	1.04307	0.61322
P52480	30.3	31.23	31.640	Pyruvate kinase PKM	15	0.83887	1.18198	1.19155	1.42117
P52503	6.47	6.47	37.070	NADH dehydrogenase [ubiquinone] iron-sulfur protein 6, mitochondrial	4	1.31622	0.72619	0.75941	0.55451

P52795	4.16	4.95	8.986	Ephrin-B1	2	0.85532	0.65988	1.16864	0.77085
P52825	7.09	8.6	4.863	Carnitine O-palmitoyltransferase 2, mitochondrial	4	1.28109	0.90469	0.78024	0.71341
P53395	2.61	3.28	1.867	Lipoamide acyltransferase component of branched-chain alpha-keto acid dehydrogenase complex, mitochondrial	1	1.15896	0.77638	0.86245	0.68280
P53564	3.07	5.86	1.782	Homeobox protein cut-like 1	3	1.30244	1.03161	0.76745	0.78705
P53986	9.26	9.41	11.360	Monocarboxylate transporter 1	5	0.89661	0.47430	1.16186	0.55241
P53994	13.1	13.91	33.490	Ras-related protein Rab-2A	7	1.08086	0.67469	0.92478	0.62997
P54071	15.3	17.74	17.480	Isocitrate dehydrogenase [NADP], mitochondrial	8	1.04233	0.67225	0.95896	0.64741
P54116	1.59	1.75	4.930	Erythrocyte band 7 integral membrane protein	1	0.79983	2.25488	1.24970	2.87541
P54775	4.7	4.91	3.589	26S protease regulatory subunit 6B	2	0.89739	0.79819	1.11384	0.89002
P54869	29.3	31.13	27.170	Hydroxymethylglutaryl-CoA synthase, mitochondrial	20	1.10639	0.25225	0.90344	0.21736
P55012	24.7	27.18	10.950	Solute carrier family 12 member 2	12	1.09845	1.71585	0.90997	1.56344
P55088	1.89	2.53	3.096	Aquaporin-4	1	0.71876	0.16676	1.39067	0.23263
P55258	2	7.55	17.390	Ras-related protein Rab-8A	6	0.95888	0.89118	1.04241	0.93183
P55264	2	3.88	3.324	Adenosine kinase	2	0.53011	0.34830	1.88555	0.65875
P55292	2.05	2.77	0.887	Desmocollin-2	1	1.45228	1.16797	0.68826	0.80461
P55302	4	5.28	5.833	Alpha-2-macroglobulin receptor-associated protein	3	1.23005	1.39747	0.81261	1.13557
P55772	4.57	4.68	4.118	Ectonucleoside triphosphate diphosphohydrolase 1	2	1.11805	2.91884	0.89402	2.74170
P56379	2	2	13.790	6.8 kDa mitochondrial proteolipid	1	0.75155	0.77054	1.32999	1.02796
P56383	2	2.12	4.795	ATP synthase F(0) complex subunit C2, mitochondrial	1	1.02319	0.91193	0.97690	0.89361
P56391	9.67	9.67	53.490	Cytochrome c oxidase subunit 6B1	7	1.19843	0.54171	0.83405	0.45319
P56393	1.46	1.46	8.750	Cytochrome c oxidase subunit 7B, mitochondrial	1	1.55861	1.16065	0.64131	0.74662
P56394	2	2.07	11.110	Cytochrome c oxidase copper chaperone	1	0.71670	0.64064	1.39465	0.89621
P56395	7.93	8.06	38.810	Cytochrome b5	4	1.93362	1.16281	0.51693	0.58454
P56404	1.44	1.62	2.682	Aquaporin-8	1	0.59310	0.18552	1.68531	0.31363
P56480	46.4	48.45	49.530	ATP synthase subunit beta, mitochondrial	38	1.07281	0.58047	0.93172	0.54284

P56528	8.33	8.76	14.800	ADP-ribosyl cyclase 1	6	0.92659	0.81247	1.07874	0.86032
P56677	12.8	12.76	9.006	Suppressor of tumorigenicity 14 protein homolog	7	0.88003	0.74609	1.13581	0.84887
P57016	3.84	4.45	2.462	Ladinin-1	1	0.99915	0.72443	1.00041	0.72748
P57096	2	2	8.130	Prostate stem cell antigen	1	1.00530	1.49426	0.99428	1.49029
P57716	12.3	13.18	9.322	Nicastrin	7	1.14516	1.22844	0.87285	1.07638
P57746	1.69	1.76	4.049	V-type proton ATPase subunit D	1	0.98544	1.45485	1.01432	1.48022
P57759	7.59	8.5	17.180	Endoplasmic reticulum resident protein 29	4	1.14218	1.10428	0.87513	0.96915
P57776	7.29	7.47	16.370	Elongation factor 1-delta	6	1.26238	0.89038	0.79180	0.70515
P57780	49.5	51.04	29.390	Alpha-actinin-4	30	0.71071	0.74432	1.40641	1.04918
P58021	8.43	9.02	5.438	Transmembrane 9 superfamily member 2	4	1.42413	0.76244	0.70187	0.56819
P58252	28.9	31.92	12.470	Elongation factor 2	14	1.11293	1.65245	0.89813	1.49234
P58466	1.86	3.35	5.747	Carboxy-terminal domain RNA polymerase II polypeptide A small phosphatase 1	2	1.09732	1.78782	0.91090	1.63352
P58771	4.21	11.1	12.680	Tropomyosin alpha-1 chain	6	1.00626	0.90869	0.99334	0.90541
P58774	2.02	5.72	9.155	Tropomyosin beta chain	3	1.11419	2.48439	0.89711	2.23562
P59242	2.45	4.34	1.008	Cingulin	1	1.49942	1.25758	0.66663	0.83820
P59325	2.74	3.1	4.895	Eukaryotic translation initiation factor 5	2	1.29001	1.68463	0.77484	1.31284
P59999	7.34	7.34	17.860	Actin-related protein 2/3 complex subunit 4	4	0.92603	1.12736	1.07940	1.21480
P60122	2	2.17	2.412	RuvB-like 1	1	1.04064	1.18293	0.96051	1.13972
P60335	4.12	8.85	14.610	Poly(rC)-binding protein 1	6	0.98362	1.60195	1.01620	1.63491
P60766	8.27	8.27	35.600	Cell division control protein 42 homolog	6	0.93489	1.16516	1.06916	1.29730
P60843	10.6	12.2	13.550	Eukaryotic initiation factor 4A-I	6	0.94381	1.25976	1.05906	1.31795
P60867	2.05	2.1	10.080	40S ribosomal protein S20	1	0.91012	1.30873	1.09826	1.44156
P61022	9.84	10.07	30.770	Calcineurin B homologous protein 1	5	0.63602	0.50120	1.57158	0.78916
P61027	6.84	13.3	31.500	Ras-related protein Rab-10	9	0.92289	0.65901	1.08307	0.71071
P61082	2.02	2.07	6.011	NEDD8-conjugating enzyme Ubc12	1	1.24062	1.00000	0.80569	0.80816

P61087	2.03	2.42	5.000	Ubiquitin-conjugating enzyme E2 K	1	0.93451	1.07944	1.06960	1.15812
P61089	6.31	6.31	23.680	Ubiquitin-conjugating enzyme E2 N	3	1.16340	1.25168	0.85916	1.08365
P61161	17.6	18.87	26.140	Actin-related protein 2	11	0.97715	1.17015	1.02292	1.20230
P61205	11.4	12.31	35.360	ADP-ribosylation factor 3	11	0.77534	0.85787	1.28917	1.09964
P61211	4	4.04	11.050	ADP-ribosylation factor-like protein 1	2	1.12538	0.76435	0.88819	0.68496
P61620	2.54	2.95	1.891	Protein transport protein Sec61 subunit alpha isoform 1	1	1.76736	1.52843	0.56556	0.83740
P61750	3.72	8.09	25.000	ADP-ribosylation factor 4	6	1.03612	0.85872	0.96470	0.82825
P61804	4.94	4.94	28.320	Dolichyl-diphosphooligosaccharide--protein glycosyltransferase subunit DAD1	4	1.67818	0.99669	0.59562	0.59328
P61924	2.16	2.26	6.215	Coatomer subunit zeta-1	1	1.07762	1.07482	0.92756	1.00000
P61979	22.8	23.13	25.920	Heterogeneous nuclear ribonucleoprotein K	12	1.35457	0.93918	0.73791	0.69872
P61982	8	13.04	24.700	14-3-3 protein gamma	9	0.90349	1.54265	1.10632	1.71559
P62071	4.75	4.77	13.240	Ras-related protein R-Ras2	2	0.85955	1.13834	1.16287	1.32834
P62073	1.92	1.93	7.778	Mitochondrial import inner membrane translocase subunit Tim10	1	1.34325	1.31476	0.74413	0.98136
P62075	3.05	3.25	26.320	Mitochondrial import inner membrane translocase subunit Tim13	2	1.04999	0.71113	0.95196	0.67563
P62077	1.96	2	13.250	Mitochondrial import inner membrane translocase subunit Tim8 B	1	0.76610	0.49377	1.30472	0.64621
P62137	4.76	4.89	6.364	Serine/threonine-protein phosphatase PP1-alpha catalytic subunit	2	1.06601	1.26763	0.93766	1.18995
P62141	2	2.77	3.058	Serine/threonine-protein phosphatase PP1-beta catalytic subunit	1	1.09646	1.21232	0.91162	1.10857
P62192	2.04	6.34	5.909	26S protease regulatory subunit 4	3	0.96136	0.87653	1.03973	0.91416
P62196	7.84	8.27	8.128	26S protease regulatory subunit 8	4	1.07535	1.07585	0.92952	1.00288
P62204	18	18.71	38.260	Calmodulin	12	0.64185	0.59633	1.55729	0.92802

P62245	4.61	4.69	18.460	40S ribosomal protein S15a	3	0.95209	1.20272	1.04985	1.26445
P62259	8.6	12.93	22.750	14-3-3 protein epsilon	9	0.85542	1.05237	1.16850	1.23460
P62264	3.73	3.73	15.230	40S ribosomal protein S14	2	1.04065	1.17972	0.96051	1.14038
P62267	3.93	3.95	13.290	40S ribosomal protein S23	2	0.92758	2.49603	1.07760	2.70915
P62270	5.21	5.26	19.740	40S ribosomal protein S18	3	0.86309	1.64704	1.15811	1.92180
P62281	3.68	4.21	11.390	40S ribosomal protein S11	2	0.89352	1.36907	1.11867	1.53600
P62305	2	2.06	11.960	Small nuclear ribonucleoprotein E	1	1.48090	1.48075	0.67496	1.00253
P62317	3.07	3.18	7.627	Small nuclear ribonucleoprotein Sm D2	1	1.65773	2.25270	0.60297	1.34289
P62320	2.52	2.79	7.143	Small nuclear ribonucleoprotein Sm D3	1	2.13917	2.43618	0.46726	1.13976
P62331	6.06	8.2	21.710	ADP-ribosylation factor 6	4	0.65079	0.95063	1.53591	1.46379
P62702	3.76	3.81	6.844	40S ribosomal protein S4, X isoform	2	0.83916	1.82881	1.19113	2.18260
P62717	2	2.03	5.682	60S ribosomal protein L18a	1	1.36163	3.16445	0.73409	2.33012
P62746	2.09	4.14	14.290	Rho-related GTP-binding protein RhoB	2	0.80169	1.20216	1.24680	1.51170
P62748	6.8	6.93	17.620	Hippocalcin-like protein 1	3	0.87638	0.61976	1.14055	0.71052
P62751	2.26	2.56	8.333	60S ribosomal protein L23a	1	1.10839	1.85488	0.90181	1.67977
P62754	3.95	4.21	8.032	40S ribosomal protein S6	2	0.92446	1.55567	1.08122	1.69397
P62806	10	10.09	50.490	Histone H4	6	1.47663	3.66166	0.67691	2.48915
P62814	9.47	10.28	9.393	V-type proton ATPase subunit B, brain isoform	4	0.99905	0.97670	1.00050	0.97997
P62821	14.1	15.75	30.240	Ras-related protein Rab-1A	9	0.77614	0.69963	1.28785	0.90518
P62827	4.31	4.9	9.722	GTP-binding nuclear protein Ran	2	0.92378	2.06226	1.08203	2.24118
P62830	2.46	2.52	14.290	60S ribosomal protein L23	2	0.97098	1.51226	1.02943	1.56521
P62852	1.86	3.25	7.200	40S ribosomal protein S25	2	0.74286	1.11506	1.34555	1.50285
P62855	2	2.06	7.826	40S ribosomal protein S26	1	0.84273	1.10407	1.18609	1.31354
P62858	2.19	2.19	17.390	40S ribosomal protein S28	1	1.10189	1.22022	0.90713	1.11622
P62862	2	2	16.950	40S ribosomal protein S30	1	0.67395	0.55517	1.48312	0.82590
P62869	2.14	2.31	7.627	Transcription elongation factor B polypeptide 2	1	0.99473	1.00903	1.00485	1.02244

P62874	19	19.62	28.820	Guanine nucleotide-binding protein G(I)/G(S)/G(T) subunit beta-1	13	0.94198	1.13022	1.06112	1.18423
P62880	6.03	18.54	28.820	Guanine nucleotide-binding protein G(I)/G(S)/G(T) subunit beta-2	11	0.95569	1.17246	1.04589	1.22480
P62889	2.39	2.39	10.430	60S ribosomal protein L30	1	1.11876	1.38350	0.89345	1.23988
P62897	5.11	5.11	23.810	Cytochrome c, somatic	4	1.28665	0.59034	0.77687	0.48578
P62908	20.7	21.22	37.040	40S ribosomal protein S3	10	1.06906	1.32139	0.93498	1.24275
P62918	3.54	3.75	7.393	60S ribosomal protein L8	2	1.14511	1.95548	0.87289	1.78287
P62962	15.5	15.62	71.430	Profilin-1	9	0.56956	1.57375	1.75495	2.71899
P62983	8.75	9.38	29.490	Ubiquitin-40S ribosomal protein S27a	6	0.83850	1.65074	1.19207	1.97596
P63001	4.28	6.52	13.540	Ras-related C3 botulinum toxin substrate 1	4	0.79460	1.01749	1.25793	1.29794
P63017	40.2	46.77	37.770	Heat shock cognate 71 kDa protein	37	0.99443	1.16578	1.00515	1.16369
P63024	4.54	4.54	32.040	Vesicle-associated membrane protein 3	4	0.80948	0.83974	1.23481	1.03929
P63038	33.6	34.36	31.760	60 kDa heat shock protein, mitochondrial	21	0.78151	0.42171	1.27900	0.54078
P63073	1.77	1.89	3.226	Eukaryotic translation initiation factor 4E	1	1.14538	1.48628	0.87268	1.30104
P63101	22.9	23.37	44.490	14-3-3 protein zeta/delta	17	0.80705	1.07840	1.23853	1.34401
P63168	2.21	2.31	7.865	Dynein light chain 1, cytoplasmic	1	0.64470	0.71699	1.55041	1.11504
P63242	2.07	2.42	5.195	Eukaryotic translation initiation factor 5A-1	1	0.98407	1.49684	1.01573	1.52527
P63276	2	2.05	8.148	40S ribosomal protein S17	1	0.49104	0.68362	2.03556	1.39583
P63323	4	4.22	13.640	40S ribosomal protein S12	2	1.06533	0.98121	0.93826	0.93093
P63328	1.35	1.68	1.152	Serine/threonine-protein phosphatase 2B catalytic subunit alpha isoform	1	1.02489	1.41955	0.97527	1.40169
P63330	1.33	1.82	2.589	Serine/threonine-protein phosphatase 2A catalytic subunit alpha isoform	1	0.85788	1.10414	1.16514	1.29043
P67778	18.5	18.98	33.090	Prohibitin	10	1.30508	1.02976	0.76590	0.80113
P67984	3.18	3.33	8.594	60S ribosomal protein L22	1	1.26602	1.80367	0.78953	1.43040

P68033	8.38	33.95	44.560	Actin, alpha cardiac muscle 1	44	1.66647	2.30231	0.59980	1.21710
P68037	3.89	4.47	10.390	Ubiquitin-conjugating enzyme E2 L3	2	1.11725	1.28744	0.89465	1.15856
P68040	9.2	9.21	14.510	Guanine nucleotide-binding protein subunit beta-2-like 1	5	1.22816	1.36644	0.81386	1.11016
P68254	4.5	12.22	17.960	14-3-3 protein theta	8	0.76566	1.35999	1.30547	1.79409
P68368	5.29	22.24	26.120	Tubulin alpha-4A chain	14	1.08283	0.93233	0.92309	0.85693
P68372	20.2	20.44	30.790	Tubulin beta-4B chain	18	0.90194	0.79727	1.10822	0.88832
P68373	22.2	22.86	28.510	Tubulin alpha-1C chain	15	1.02805	1.56876	0.97228	1.53232
P68510	8.58	15.15	28.460	14-3-3 protein eta	10	0.80777	1.30736	1.23743	1.61804
P70122	2	5.04	6.000	Ribosome maturation protein SBDS	3	0.77668	1.25223	1.28695	1.61651
P70124	8.81	9.56	13.870	Serpin B5	5	0.88795	1.15406	1.12568	1.29814
P70168	2.19	3.22	0.913	Importin subunit beta-1	1	1.11173	1.20690	0.89910	1.08846
P70195	2.85	3.18	6.498	Proteasome subunit beta type-7	2	0.56769	1.00840	1.76072	1.78096
P70296	4.02	4.02	14.440	Phosphatidylethanolamine-binding protein 1	2	0.76769	0.75816	1.30203	1.00433
P70302	9.55	11.15	5.255	Stromal interaction molecule 1	4	1.60126	0.95517	0.62423	0.60015
P70333	1.46	4.27	3.563	Heterogeneous nuclear ribonucleoprotein H2	2	0.66228	0.62360	1.50926	0.94407
P70349	2.36	3.76	15.080	Histidine triad nucleotide-binding protein 1	2	0.49194	0.49845	2.03187	1.01660
P70404	2.02	3.77	3.562	Isocitrate dehydrogenase [NAD] subunit gamma 1, mitochondrial	2	0.83472	0.75820	1.19747	0.90847
P70424	6.71	10.92	3.264	Receptor tyrosine-protein kinase erbB-2	6	1.04015	0.96464	0.96097	0.92815
P70441	14.2	15.55	23.940	Na(+)/H(+) exchange regulatory cofactor NHE-RF1	8	0.42317	0.40276	2.36206	0.94891
P70452	1.36	1.6	3.691	Syntaxin-4	1	0.71415	0.72977	1.39963	1.03080
P70460	6.26	6.3	8.000	Vasodilator-stimulated phosphoprotein	3	0.94086	1.18749	1.06238	1.26062
P70670	2.08	2.66	0.594	Nascent polypeptide-associated complex subunit alpha, muscle-specific form	1	0.99915	0.73998	1.00040	0.74255
P70695	1.63	1.71	2.950	Fructose-1,6-bisphosphatase isozyme 2	1	0.65481	0.54912	1.52648	0.83713
P80313	5.7	7.96	3.125	T-complex protein 1 subunit eta	2	1.11766	1.47005	0.89432	1.32032

P80314	16.5	17.64	16.260	T-complex protein 1 subunit beta	8	0.91166	1.21753	1.09641	1.31549
P80315	16.5	17.26	16.510	T-complex protein 1 subunit delta	8	1.01512	1.33972	0.98466	1.33016
P80316	13.4	14.49	9.612	T-complex protein 1 subunit epsilon	7	0.95348	1.15227	1.04832	1.21379
P80317	16.2	18.95	15.250	T-complex protein 1 subunit zeta	9	0.86209	1.18249	1.15945	1.37318
P80318	11.7	16.1	11.190	T-complex protein 1 subunit gamma	8	0.80753	1.11508	1.23779	1.36839
P84084	2	8.18	22.220	ADP-ribosylation factor 5	9	1.51738	1.17172	0.65873	0.77423
P84096	3.76	5.86	14.140	Rho-related GTP-binding protein RhoG	3	0.79831	1.47488	1.25209	1.87342
P86048	3.75	4.33	7.944	60S ribosomal protein L10-like	2	0.96009	1.90689	1.04110	1.99387
P97300	5.81	5.96	6.297	Neuroplastin	3	0.75656	0.58315	1.32118	0.77190
P97313	2.4	8.53	0.170	DNA-dependent protein kinase catalytic subunit	1	1.12065	1.45618	0.89194	1.30057
P97315	8.71	8.91	28.500	Cysteine and glycine-rich protein 1	5	1.31480	2.45760	0.76023	1.85005
P97351	1.59	2.79	6.061	40S ribosomal protein S3a	2	0.81807	1.61488	1.22185	1.97792
P97352	1.78	1.78	8.163	Protein S100-A13	1	1.14329	1.13642	0.87428	0.99660
P97370	1.76	1.89	4.676	Sodium/potassium-transporting ATPase subunit beta-3	1	1.06368	2.16764	0.93971	2.05050
P97371	13.5	13.93	28.920	Proteasome activator complex subunit 1	6	0.76965	0.70943	1.29871	0.92381
P97372	6.57	6.88	14.230	Proteasome activator complex subunit 2	4	0.81270	0.80900	1.22991	0.98797
P97384	20.8	25.21	25.250	Annexin A11	17	1.05967	1.18580	0.94326	1.12057
P97429	24.8	29.55	45.770	Annexin A4	20	1.04792	1.69148	0.95385	1.60990
P97430	1.39	1.4	6.107	Antileukoproteinase	1	1.32128	1.71718	0.75650	1.30304
P97447	3.99	4.02	8.214	Four and a half LIM domains protein 1	2	0.95220	1.42867	1.04973	1.50273
P97449	1.31	1.89	0.725	Aminopeptidase N	1	1.15976	1.71478	0.86186	1.50310
P97450	5.34	5.55	32.410	ATP synthase-coupling factor 6, mitochondrial	3	1.23974	0.84230	0.80626	0.67966
P97792	8.13	8.37	13.970	Coxsackievirus and adenovirus receptor homolog	5	1.02488	1.09644	0.97529	1.06900
P97805	10	10.12	23.770	Protein FAM3D	7	1.19780	0.65154	0.83449	0.55065
P97807	15.1	15.43	15.580	Fumarate hydratase, mitochondrial	8	0.68259	0.41666	1.46435	0.61055
P97823	3.64	3.69	8.261	Acyl-protein thioesterase 1	2	0.66636	0.87274	1.50002	1.31098

P97855	2.06	2.11	2.366	Ras GTPase-activating protein-binding protein 1	1	3.03449	2.19590	0.32940	0.72554
P97864	2.02	2.44	3.300	Caspase-7	1	1.19555	0.86000	0.83606	0.71827
P99024	5.89	19.9	28.150	Tubulin beta-5 chain	16	2.22312	4.22984	0.44962	1.93727
P99026	1.98	3.25	5.303	Proteasome subunit beta type-4	2	0.72350	0.98197	1.38155	1.36081
P99027	9.27	9.27	47.830	60S acidic ribosomal protein P2	6	1.01527	0.98285	0.98452	0.96592
P99028	8.25	8.3	41.570	Cytochrome b-c1 complex subunit 6, mitochondrial	4	1.30751	0.76463	0.76447	0.58569
P99029	10.8	11.12	29.050	Peroxiredoxin-5, mitochondrial	6	0.73315	0.97035	1.36337	1.33198
Q00612	2.27	2.5	1.359	Glucose-6-phosphate 1-dehydrogenase X	1	0.97134	1.85717	1.02904	1.91699
Q00623	6.4	6.66	11.360	Apolipoprotein A-I	3	1.58336	1.37026	0.63128	0.87522
Q01339	6.48	9	10.140	Beta-2-glycoprotein 1	4	1.39312	1.62047	0.71749	1.19090
Q01768	9.57	10.03	33.550	Nucleoside diphosphate kinase B	5	1.10539	1.15780	0.90425	1.02164
Q01853	34.4	35.8	22.210	Transitional endoplasmic reticulum ATPase	18	1.23512	1.19335	0.80928	0.98297
Q02053	8.29	9.32	3.214	Ubiquitin-like modifier-activating enzyme 1	4	0.95537	1.01855	1.04624	1.06827
Q02248	36.7	36.87	24.710	Catenin beta-1	25	1.08820	1.19026	0.91853	1.09551
Q02257	20	29.02	20.130	Junction plakoglobin	16	1.17108	1.08889	0.85353	0.92942
Q02819	22.4	24.02	25.270	Nucleobindin-1	11	1.48758	0.74663	0.67193	0.50671
Q03265	42.7	46.79	39.420	ATP synthase subunit alpha, mitochondrial	35	1.39654	0.77156	0.71573	0.58658
Q04447	2.03	6.01	8.399	Creatine kinase B-type	3	0.96574	1.16817	1.03501	1.21224
Q05144	1.86	5.45	13.540	Ras-related C3 botulinum toxin substrate 2	4	2.09937	5.09039	0.47612	2.54760
Q06138	4.96	5.74	4.692	Calcium-binding protein 39	2	0.55885	0.97526	1.78858	1.76635
Q06185	5.74	6.55	50.700	ATP synthase subunit e, mitochondrial	5	1.08302	0.88788	0.92293	0.81997
Q07076	18.2	18.32	19.220	Annexin A7	11	1.16834	1.97517	0.85553	1.76625
Q07113	3.82	4.94	0.604	Cation-independent mannose-6-phosphate receptor	2	0.98682	1.07188	1.01290	1.08535
Q07417	13.2	13.19	19.420	Short-chain specific acyl-CoA dehydrogenase, mitochondrial	7	1.06761	0.55105	0.93625	0.52586
Q08091	2.22	2.6	3.704	Calponin-1	1	1.46257	1.15814	0.68342	0.78967

Q08189	1.77	1.94	1.010	Protein-glutamine gamma-glutamyltransferase E	1	0.50797	0.22795	1.96775	0.44880
Q08509	11.2	11.89	5.481	Epidermal growth factor receptor kinase substrate 8	4	0.51226	0.75691	1.95127	1.47887
Q09199	15.5	15.83	15.880	Beta-1,4 N-acetylgalactosaminyltransferase 2	8	0.89537	0.49715	1.11636	0.60068
Q09200	4.63	7.27	4.503	Beta-1,4 N-acetylgalactosaminyltransferase 1	3	0.97586	0.56343	1.02427	0.61473
Q10470	2.02	2.2	1.859	Beta-1,4-mannosyl-glycoprotein 4-beta-N-acetylglucosaminyltransferase	1	0.85701	0.52113	1.16633	0.61631
Q148W0	6.24	7.67	2.398	Probable phospholipid-transporting ATPase IC	3	0.89621	0.70767	1.11530	0.79290
Q2KHK6	7.3	9.26	7.500	Gasdermin-C2	5	0.95887	0.58598	1.04242	0.57570
Q2TBE6	2.06	2.5	2.296	Phosphatidylinositol 4-kinase type 2-alpha	1	1.09918	1.87032	0.90936	1.70602
Q2TPA8	2.02	2.18	1.633	Hydroxysteroid dehydrogenase-like protein 2	1	0.91593	0.88941	1.09130	0.97359
Q3TFQ1	1.32	1.4	5.102	SPRY domain-containing protein 7	1	0.94925	0.51325	1.05299	0.54211
Q3THE2	12.2	12.34	33.140	Myosin regulatory light chain 12B	8	1.00579	1.25826	0.99380	1.25313
Q3TMQ6	2.06	2.06	6.944	Angiogenin-4	1	0.68340	0.20319	1.46263	0.29894
Q3TTY5	2	8.13	4.526	Keratin, type II cytoskeletal 2 epidermal	5	0.30701	0.51538	3.25571	1.68309
Q3TXS7	2.12	5.53	2.204	26S proteasome non-ATPase regulatory subunit 1	3	0.61182	0.75218	1.63373	1.23144
Q3U0V1	1.49	1.54	2.540	Far upstream element-binding protein 2	2	1.29920	1.04421	0.76936	0.80584
Q3U1J4	2.33	4.55	2.193	DNA damage-binding protein 1	3	1.11901	1.04445	0.89324	0.93581
Q3U7R1	4.43	6.97	2.381	Extended synaptotagmin-1	3	1.22369	1.82506	0.81684	1.54744
Q3ULJ0	3.19	4.33	3.989	Glycerol-3-phosphate dehydrogenase 1-like protein	2	0.98552	0.71143	1.01424	0.72490
Q3UMR5	2.23	2.76	5.429	Calcium uniporter protein, mitochondrial	2	1.04702	1.11839	0.95466	1.07097
Q3UNX5	2	2.67	1.724	Acyl-coenzyme A synthetase ACSM3, mitochondrial	1	0.82058	0.43130	1.21810	0.52698
Q3UPL0	4.54	7.59	1.707	Protein transport protein Sec31A	3	1.00738	0.84264	0.99223	0.83614
Q3UQ44	17	20.35	3.810	Ras GTPase-activating-like protein IQGAP2	6	0.88270	0.77373	1.13238	0.87708
Q3URD3	4.02	4.85	2.249	Sarcolemmal membrane-associated protein	2	1.57883	1.82609	0.63310	1.16442
Q3USF0	4.42	4.5	5.371	UDP-GlcNAc:betaGal beta-1,3-N-acetylglucosaminyltransferase 6	2	0.93583	0.47514	1.06809	0.47667

Q3UV17	2.12	9.81	6.734	Keratin, type II cytoskeletal 2 oral	11	0.32403	0.56342	3.08470	1.74332
Q3UW53	4.39	7.85	1.080	Protein Niban	2	0.92522	0.79985	1.08034	0.86546
Q3V0K9	24.3	27.85	18.410	Plastin-1	13	0.57738	0.50569	1.73119	0.87419
Q3V3R1	2.68	6.61	3.275	Monofunctional C1-tetrahydrofolate synthase, mitochondrial	4	1.60462	1.23847	0.62292	0.78500
Q501J6	4.06	16.52	9.692	Probable ATP-dependent RNA helicase DDX17	7	1.16147	1.37844	0.86060	1.18967
Q52KP5	1.5	3.09	2.578	NXPE family member 4	2	1.01656	0.99384	0.98326	0.98021
Q5FWK3	5.7	6.13	6.834	Rho GTPase-activating protein 1	3	1.25344	1.24537	0.79745	0.99362
Q5JCS9	3.71	3.78	1.882	UDP-GlcNAc:betaGal beta-1,3-N-acetylglucosaminyltransferase 3	1	1.10264	0.91088	0.90651	0.83364
Q5JCT0	2.99	5.81	6.636	Beta-1,3-galactosyl-O-glycosyl-glycoprotein beta-1,6-N-acetylglucosaminyltransferase 3	3	0.86349	0.69975	1.15757	0.81248
Q5SWT3	3.68	3.68	5.667	Solute carrier family 25 member 35	2	1.54084	1.18554	0.64871	0.77073
Q5SYD0	6.15	7.53	2.783	Unconventional myosin-Id	4	0.46726	0.63749	2.13919	1.36942
Q5XJY5	11	11.36	9.980	Coatomer subunit delta	5	0.97418	0.91512	1.02605	0.93709
Q60597	4.54	5.23	2.248	2-oxoglutarate dehydrogenase, mitochondrial	2	1.03798	0.53513	0.96298	0.52382
Q60598	14.9	14.92	13.190	Src substrate cortactin	7	1.04047	0.97696	0.96067	0.93547
Q60604	12.3	13.92	8.392	Adseverin	5	0.51433	0.41956	1.94342	0.81198
Q60605	11.9	12.18	40.400	Myosin light polypeptide 6	11	1.00197	1.09829	0.99759	1.09440
Q60634	13.3	13.86	17.990	Flotillin-2	7	1.13996	2.39042	0.87683	2.11084
Q60668	4.06	4.25	6.197	Heterogeneous nuclear ribonucleoprotein D0	2	0.56430	0.28829	1.77132	0.50759
Q60766	6.21	6.43	8.802	Immunity-related GTPase family M protein 1	3	1.17328	1.25916	0.85193	1.07169
Q60770	2.6	3.13	1.689	Syntaxin-binding protein 3	1	0.84797	1.03533	1.17876	1.22598
Q60854	9.13	11.58	14.810	Serpin B6	7	0.66211	0.51535	1.50965	0.78188
Q60864	6.09	6.62	5.157	Stress-induced-phosphoprotein 1	3	0.86436	0.98949	1.15640	1.15336
Q60865	2.55	3.8	3.678	Caprin-1	3	0.85412	0.74503	1.17027	0.87457

Q60870	5.84	5.97	14.590	Receptor expression-enhancing protein 5	3	1.26993	1.13329	0.78709	0.89759
Q60930	18.4	19.04	30.850	Voltage-dependent anion-selective channel protein 2	13	1.76814	1.28828	0.56531	0.72601
Q60931	8.76	11.83	18.730	Voltage-dependent anion-selective channel protein 3	7	1.57970	1.01993	0.63275	0.64264
Q60932	19.4	19.79	39.530	Voltage-dependent anion-selective channel protein 1	17	1.54424	1.09034	0.64728	0.70714
Q60973	2.12	2.12	3.059	Histone-binding protein RBBP7	1	1.09714	1.04683	0.91105	0.95927
Q60997	2	2.37	0.480	Deleted in malignant brain tumors 1 protein	1	1.56226	1.09082	0.63981	0.70007
Q61029	3.85	3.96	4.646	Lamina-associated polypeptide 2, isoforms beta/delta/epsilon/gamma	2	3.15024	8.19725	0.31729	2.23380
Q61081	2	2.23	2.902	Hsp90 co-chaperone Cdc37	1	0.93986	0.91939	1.06352	0.98080
Q61112	2.73	3.31	2.493	45 kDa calcium-binding protein	1	1.18979	0.89492	0.84011	0.77100
Q61133	1.89	1.99	2.869	Glutathione S-transferase theta-2	1	0.77192	0.68965	1.29489	0.89577
Q61165	1.43	1.64	0.854	Sodium/hydrogen exchanger 1	1	0.80092	0.48306	1.24800	0.59952
Q61171	8.5	9.13	21.210	Peroxiredoxin-2	4	0.70401	1.38052	1.41980	1.96106
Q61187	3.6	3.65	4.092	Tumor susceptibility gene 101 protein	2	0.98725	1.21740	1.01246	1.25526
Q61190	2	2.23	3.725	Interleukin-10 receptor subunit beta	1	0.96945	0.46781	1.03105	0.48382
Q61205	2.1	3.56	3.879	Platelet-activating factor acetylhydrolase IB subunit gamma	3	0.69157	1.10206	1.44534	1.59694
Q61207	11.6	12.43	11.670	Sulfated glycoprotein 1	8	0.99983	1.49645	0.99972	1.50696
Q61233	6.89	8.72	5.582	Plastin-2	3	0.98481	1.96889	1.01497	2.01015
Q61335	12.5	12.92	20.410	B-cell receptor-associated protein 31	6	1.49949	1.06709	0.66659	0.71172
Q61420	2.06	2.64	3.571	CMP-sialic acid transporter	1	1.30014	0.50487	0.76881	0.40292
Q61425	14.5	15	21.340	Hydroxyacyl-coenzyme A dehydrogenase, mitochondrial	7	1.18099	0.53983	0.84637	0.47491
Q61490	4.4	4.6	3.602	CD166 antigen	2	1.12258	1.76347	0.89040	1.56914
Q61503	6.5	9.02	5.903	5'-nucleotidase	4	0.48477	0.94999	2.06192	1.96430
Q61543	31.7	32.98	10.130	Golgi apparatus protein 1	13	1.25888	1.16059	0.79400	0.92556
Q61598	16	16.5	16.400	Rab GDP dissociation inhibitor beta	7	0.73414	0.90894	1.36153	1.23854

Q61656	23.2	23.7	14.500	Probable ATP-dependent RNA helicase DDX5	10	1.50489	1.63619	0.66420	1.08056
Q61696	1.49	13.09	10.760	Heat shock 70 kDa protein 1A	11	0.60251	0.62125	1.65897	1.03354
Q61735	5.86	7.62	12.870	Leukocyte surface antigen CD47	4	1.21559	2.57839	0.82227	2.13058
Q61739	24.8	28.39	9.441	Integrin alpha-6	10	1.11431	1.76434	0.89702	1.58806
Q61753	5.76	5.88	6.379	D-3-phosphoglycerate dehydrogenase	3	0.91198	1.35656	1.09603	1.49656
Q61765	2.02	16.23	19.470	Keratin, type I cuticular Ha1	9	4.36866	2.38036	0.22880	0.54630
Q61792	4.91	5.77	10.270	LIM and SH3 domain protein 1	3	0.82686	1.00866	1.20886	1.22521
Q61847	4.67	4.84	4.261	Meprin A subunit beta	3	0.55238	0.36772	1.80955	0.67077
Q61937	3.58	4.06	7.534	Nucleophosmin	2	1.65291	1.85741	0.60472	1.09369
Q61941	18.1	18.98	8.748	NAD(P) transhydrogenase, mitochondrial	9	1.08805	1.23867	0.91867	1.14377
Q61990	9	9.3	14.360	Poly(rC)-binding protein 2	5	0.91831	1.10526	1.08847	1.20488
Q62086	2.37	4.12	7.345	Serum paraoxonase/arylesterase 2	2	1.78118	1.07990	0.56117	0.60787
Q62087	5.59	5.98	9.322	Serum paraoxonase/lactonase 3	3	1.33537	0.70053	0.74852	0.52708
Q62167	8.4	11.46	8.157	ATP-dependent RNA helicase DDX3X	5	1.19403	1.28107	0.83712	1.07630
Q62186	4.76	4.84	13.370	Translocon-associated protein subunit delta	3	2.06300	1.09686	0.48451	0.54240
Q62261	71.7	74.43	13.080	Spectrin beta chain, non-erythrocytic 1	33	1.18192	1.17657	0.84570	0.98693
Q62273	11.6	12.8	6.631	Sulfate transporter	6	0.74631	0.47967	1.33933	0.65947
Q62313	6.54	6.62	6.516	Trans-Golgi network integral membrane protein 1	3	0.94487	0.59466	1.05787	0.62752
Q62318	2.58	2.77	1.799	Transcription intermediary factor 1-beta	1	0.94815	1.12686	1.05421	1.19100
Q62351	5.79	7.21	3.801	Transferrin receptor protein 1	3	1.15184	1.34055	0.86778	1.16333
Q62393	6.18	6.37	17.860	Tumor protein D52	3	1.03235	0.69037	0.96823	0.67019
Q62425	5.99	5.99	35.370	NADH dehydrogenase [ubiquinone] 1 alpha subcomplex subunit 4	4	1.42818	1.22722	0.69988	0.86156
Q62426	2.1	2.17	11.220	Cystatin-B	1	0.80251	1.24364	1.24553	1.55389
Q62433	7.9	8.53	12.940	Protein NDRG1	4	1.16495	0.60555	0.85802	0.51979
Q62446	2	2.03	4.911	Peptidyl-prolyl cis-trans isomerase FKBP3	1	1.22350	2.88039	0.81696	2.36039

Q62465	13.9	13.92	21.430	Synaptic vesicle membrane protein VAT-1 homolog	7	1.14212	1.04500	0.87517	0.91908
Q62468	55.7	55.93	33.980	Villin-1	30	0.41943	0.61124	2.38315	1.45969
Q62469	22.8	24.18	11.460	Integrin alpha-2	13	0.97949	0.95709	1.02049	0.97644
Q62470	7.84	9.36	4.748	Integrin alpha-3	6	0.89743	1.07270	1.11379	1.20091
Q63739	1.34	1.7	7.514	Protein tyrosine phosphatase type IVA 1	1	0.62313	0.52945	1.60409	0.85193
Q63844	9.16	10	15.530	Mitogen-activated protein kinase 3	5	0.89942	0.89764	1.11132	0.99820
Q64133	16.5	17.42	13.120	Amine oxidase [flavin-containing] A	8	1.15750	0.51689	0.86355	0.46966
Q64310	2.55	2.69	6.691	Surfeit locus protein 4	2	1.21059	0.94263	0.82567	0.80693
Q64331	2.04	3.27	0.711	Unconventional myosin-VI	1	0.62418	1.07546	1.60139	1.72753
Q64433	10.2	10.25	60.780	10 kDa heat shock protein, mitochondrial	6	0.64283	0.75621	1.55491	1.19115
Q64435	1.64	2.79	4.896	UDP-glucuronosyltransferase 1-6	3	2.50915	0.24435	0.39836	0.09764
Q64442	2	3.92	3.922	Sorbitol dehydrogenase	2	0.69854	0.87261	1.43093	1.25247
Q64444	10.5	10.55	17.700	Carbonic anhydrase 4	8	0.37593	0.13503	2.65889	0.35536
Q64514	1.64	2.07	0.792	Tripeptidyl-peptidase 2	1	0.90958	1.68378	1.09891	1.85602
Q64518	18.8	21.54	10.310	Sarcoplasmic/endoplasmic reticulum calcium ATPase 3	10	1.32149	0.72748	0.75638	0.55680
Q64521	35	35.17	26.690	Glycerol-3-phosphate dehydrogenase, mitochondrial	17	1.47739	1.42121	0.67657	0.95823
Q64522	6.26	6.43	26.920	Histone H2A type 2-B	5	1.52162	3.97934	0.65690	2.69847
Q64704	4.27	4.31	8.997	Syntaxin-3	3	0.62491	0.69883	1.59950	1.12421
Q64727	31.8	32.46	14.540	Vinculin	15	0.95768	1.24820	1.04372	1.29773
Q64735	3.47	3.63	3.313	Complement component receptor 1-like protein	2	0.92611	1.08928	1.07931	1.19073
Q68FD5	51.7	54.07	15.760	Clathrin heavy chain 1	27	0.96256	0.95962	1.03843	1.00205
Q68FL4	7.5	7.87	6.852	Putative adenosylhomocysteinase 3	4	0.90209	0.69566	1.10804	0.79573
Q69ZF7	27.8	28.47	17.120	Metal transporter CNNM4	17	0.89019	0.41236	1.12286	0.46345
Q69ZN7	3.13	8.85	0.586	Myoferlin	2	1.17355	2.20644	0.85174	1.95200
Q6A068	1.57	4.28	1.372	Cell division cycle 5-like protein	2	1.16576	0.51714	0.85742	0.44477
Q6GQT9	13.2	15.71	4.860	Nodal modulator 1	6	1.61427	1.04933	0.61920	0.64892

Q6IFZ6	3.67	11.59	8.217	Keratin, type II cytoskeletal 1b	7	0.34314	0.71616	2.91292	2.09252
Q6IRU2	6.78	9	16.940	Tropomyosin alpha-4 chain	5	0.94687	1.83379	1.05563	1.90239
Q6IRU5	5.71	5.71	11.350	Clathrin light chain B	3	0.77384	0.75656	1.29168	0.98029
Q6P069	6.19	6.19	20.710	Sorcin	4	0.76151	1.03209	1.31260	1.35813
Q6P5E4	23.4	25.18	7.092	UDP-glucose:glycoprotein glucosyltransferase 1	11	1.17808	0.88561	0.84846	0.75517
Q6PB66	6.81	13.25	2.227	Leucine-rich PPR motif-containing protein, mitochondrial	5	1.15012	0.74739	0.86909	0.64995
Q6PD26	3.16	3.79	4.505	GPI transamidase component PIG-S	2	1.43635	1.36503	0.69590	0.95516
Q6PDM2	1.88	1.91	3.226	Serine/arginine-rich splicing factor 1	1	1.49776	1.79023	0.66736	1.22228
Q6PDN3	1.64	4.94	0.824	Myosin light chain kinase, smooth muscle	2	1.94672	2.17237	0.51345	1.11884
Q6PHN9	2	6.22	15.420	Ras-related protein Rab-35	4	0.82494	1.03637	1.21166	1.25958
Q6PHU5	5.4	8.11	2.061	Sortilin	3	1.07889	1.14110	0.92646	1.05834
Q6PHZ2	2.29	2.88	4.609	Calcium/calmodulin-dependent protein kinase type II subunit delta	2	1.06434	0.97349	0.93913	0.92462
Q6Q473	22.1	22.54	10.820	Calcium-activated chloride channel regulator 4	12	0.69125	0.51038	1.44600	0.74562
Q6R0H7	2.09	5.3	1.853	Guanine nucleotide-binding protein G(s) subunit alpha isoforms XLas	2	0.81671	1.49565	1.22388	1.84055
Q6URW6	48	67.67	14.400	Myosin-14	29	1.42788	0.93851	0.70003	0.66218
Q6X893	8.06	8.08	6.891	Choline transporter-like protein 1	4	0.95323	0.73184	1.11931	0.81909
Q6ZQ38	4.27	5.92	2.602	Cullin-associated NEDD8-dissociated protein 1	3	0.95889	1.19480	1.04241	1.24071
Q6ZQI3	9.41	9.54	12.370	Malectin	4	1.32267	1.04911	0.75571	0.77880
Q6ZQM8	9.83	10.31	11.490	UDP-glucuronosyltransferase 1-7C	6	2.49953	0.94858	0.45592	0.44009
Q6ZWX6	3.55	4.52	8.254	Eukaryotic translation initiation factor 2 subunit 1	3	0.86002	0.92195	1.16224	1.08079
Q6ZWY3	2	2	15.480	40S ribosomal protein S27-like	1	1.21612	1.64173	0.82192	1.35352
Q76MZ3	8.24	8.94	6.621	Serine/threonine-protein phosphatase 2A 65 kDa regulatory subunit A alpha isoform	4	0.86490	1.00354	1.15569	1.16576

Q78IK4	1.89	2.11	2.642	Apolipoprotein O-like	1	1.37855	1.60818	0.72507	1.16963
Q78PY7	4.74	8.63	3.187	Staphylococcal nuclease domain-containing protein 1	4	1.41440	2.24030	0.70670	1.59186
Q78S06	1.62	1.64	5.769	Protein SYS1 homolog	1	0.84556	0.53152	1.18212	0.63025
Q7M6Y3	2.01	2.35	1.212	Phosphatidylinositol-binding clathrin assembly protein	1	0.78976	1.22198	1.26564	1.55134
Q7TMF3	3.16	4.45	16.550	NADH dehydrogenase [ubiquinone] 1 alpha subcomplex subunit 12	3	1.14090	0.85398	0.87611	0.74918
Q7TMK9	3.32	4.65	3.531	Heterogeneous nuclear ribonucleoprotein Q	3	0.96187	1.17446	1.03918	1.22422
Q7TN37	4.71	5.69	1.566	Transient receptor potential cation channel subfamily M member 4	2	1.00345	0.55049	0.99612	0.53678
Q7TNS2	1.72	1.72	10.530	Mitochondrial inner membrane organizing system protein 1	1	1.04368	1.08505	0.95771	1.04236
Q7TPR4	16	39.09	22.530	Alpha-actinin-1	21	0.87872	1.50763	1.13751	1.72621
Q7TST0	2	2.4	2.161	Butyrophilin-like protein 1	1	0.93462	0.20874	1.06948	0.22393
Q7TT45	2.02	2.49	2.004	Ras-related GTP-binding protein D	1	0.42691	1.37082	2.34136	3.21946
Q80SZ7	2.87	2.87	13.240	Guanine nucleotide-binding protein G(I)/G(S)/G(O) subunit gamma-5	1	0.67424	1.24031	1.48250	1.83569
Q80TH2	9.38	9.86	2.924	Protein LAP2	4	0.82810	0.67757	1.20704	0.83952
Q80U72	3.5	4.06	0.869	Protein scribble homolog	1	1.27985	1.44903	0.78099	1.13330
Q80UG5	7.91	8.44	4.631	Septin-9	4	1.29918	2.46216	0.76937	1.93365
Q80UU9	1.82	6.77	10.600	Membrane-associated progesterone receptor component 2	3	1.58234	1.23773	0.63169	0.78507
Q80V26	9.22	10.04	11.520	Inositol monophosphatase 3	4	1.01462	0.53656	0.98515	0.51956
Q80VA0	7.33	8.61	5.632	N-acetylgalactosaminyltransferase 7	4	1.17543	0.65054	0.85037	0.55680
Q80VQ1	4.04	5.92	5.725	Leucine-rich repeat-containing protein 1	3	1.21009	0.83490	0.82602	0.69309
Q80W54	3.6	4.25	3.368	CAAX prenyl protease 1 homolog	2	1.09458	1.07481	0.91319	0.98326
Q80WJ7	2.01	2.27	2.073	Protein LYRIC	1	1.57077	1.66108	0.63635	1.06027
Q80X90	123	123.3	25.900	Filamin-B	56	1.11186	1.20302	0.89899	1.07976

Q80XN0	14.4	14.63	18.080	D-beta-hydroxybutyrate dehydrogenase, mitochondrial	6	1.35112	0.70688	0.73979	0.53060
Q80Y14	1.35	1.47	3.947	Glutaredoxin-related protein 5, mitochondrial	1	0.36937	0.50069	2.70611	1.35907
Q80YW5	1.56	4.74	2.960	B box and SPRY domain-containing protein	2	1.19893	1.36542	0.83370	1.15245
Q80Z19	61.2	62.04	8.993	Mucin-2 (Fragments)	29	0.57050	0.23734	1.75208	0.41247
Q810Q5	3.59	3.7	18.070	Normal mucosa of esophagus-specific gene 1 protein	2	1.18867	0.53873	0.84090	0.46168
Q811D0	7.8	8.28	4.088	Disks large homolog 1	4	0.88948	0.83284	1.12375	0.93809
Q812C9	1.62	2.18	1.189	Retina-specific copper amine oxidase	1	1.15626	2.34588	0.86447	2.03418
Q8BFR5	31.4	32.81	35.620	Elongation factor Tu, mitochondrial	15	0.98220	0.51227	1.01767	0.52544
Q8BFZ9	13.5	13.64	19.120	Erlin-2	7	1.40340	0.90169	0.71223	0.64260
Q8BG05	10.8	11.59	16.890	Heterogeneous nuclear ribonucleoprotein A3	6	0.88485	0.36806	1.12963	0.41244
Q8BG32	6.43	7.12	7.583	26S proteasome non-ATPase regulatory subunit 11	4	0.88996	0.84900	1.12314	0.95576
Q8BGC4	6.12	8.63	10.340	Zinc-binding alcohol dehydrogenase domain-containing protein 2	4	1.50590	1.11603	0.66376	0.72987
Q8BGD8	2	2	13.920	Cytochrome c oxidase assembly factor 6 homolog	1	1.26949	0.95361	0.78736	0.75314
Q8BGH2	3.27	3.5	2.132	Sorting and assembly machinery component 50 homolog	1	1.36887	1.27831	0.73020	0.93182
Q8BGT9	4.83	5.13	2.951	Polypeptide N-acetylgalactosaminyltransferase 12	2	0.97349	0.45304	1.02678	0.46720
Q8BGZ7	7.1	21.24	14.160	Keratin, type II cytoskeletal 75	19	0.19650	0.49544	5.08670	2.52788
Q8BH24	6.87	7.44	6.376	Transmembrane 9 superfamily member 4	4	1.25971	0.89283	0.79348	0.71124
Q8BH43	7.39	7.46	7.847	Wiskott-Aldrich syndrome protein family member 2	4	1.00771	1.40000	0.99190	1.38958
Q8BH95	19	19.62	35.520	Enoyl-CoA hydratase, mitochondrial	12	1.13300	0.41149	0.88222	0.36394
Q8BHG3	1.37	1.66	3.683	Cell cycle control protein 50B	1	0.86514	0.80048	1.15537	0.92688
Q8BHL4	1.88	2.13	3.652	Retinoic acid-induced protein 3	1	0.43359	0.24839	2.30528	0.57830
Q8BHN3	3.01	3.29	2.331	Neutral alpha-glucosidase AB	2	1.32985	1.13538	0.75163	0.85820
Q8BHY3	2.07	2.82	0.938	Anoctamin-1	1	0.70612	1.32599	1.41556	1.88279
Q8BI08	2	2	6.286	Protein MAL2	1	1.15781	0.88960	0.86331	0.77036
Q8BI84	4.78	6.15	1.088	Melanoma inhibitory activity protein 3	2	1.25897	0.88612	0.79394	0.70561

Q8BIQ5	2	2.31	1.552	Cleavage stimulation factor subunit 2	1	0.99956	1.08591	1.00000	1.08924
Q8BJ64	7.93	7.97	4.866	Choline dehydrogenase, mitochondrial	3	1.12903	0.94500	0.88532	0.84411
Q8BJF9	1.64	1.89	3.286	Charged multivesicular body protein 2b	1	1.23158	1.36683	0.81160	1.10600
Q8BK30	2.19	2.51	10.580	NADH dehydrogenase [ubiquinone] flavoprotein 3, mitochondrial	1	1.17632	1.01856	0.84973	0.92326
Q8BK48	3.59	6.14	4.651	Pyrethroid hydrolase Ces2e	3	1.35092	0.35552	0.73990	0.26012
Q8BK64	3.01	3.34	5.621	Activator of 90 kDa heat shock protein ATPase homolog 1	2	0.93092	1.19749	1.07372	1.29434
Q8BK72	1.49	1.66	1.928	28S ribosomal protein S27, mitochondrial	1	0.89489	0.80215	1.11695	0.89872
Q8BKX1	2.13	2.55	1.869	Brain-specific angiogenesis inhibitor 1-associated protein 2	1	0.65429	1.56872	1.52768	2.40386
Q8BLF1	2.13	3.39	3.431	Neutral cholesterol ester hydrolase 1	2	1.34749	0.83696	0.74179	0.65421
Q8BLN5	1.54	2.06	1.228	Lanosterol synthase	1	1.66649	0.95032	0.59980	0.57175
Q8BLX4	3.05	3.94	6.061	GDP-fucose transporter 1	2	0.99690	0.68558	1.00266	0.68891
Q8BM96	2.94	3.31	2.038	Probable G-protein coupled receptor 128	2	0.61767	0.48501	1.61825	0.78729
Q8BMD8	13.4	15.3	15.370	Calcium-binding mitochondrial carrier protein SCaMC-1	9	1.41538	0.96089	0.70621	0.67397
Q8BMF4	17.5	18.24	16.670	Dihydrolipoyllysine-residue acetyltransferase component of pyruvate dehydrogenase complex, mitochondrial	10	0.81755	0.44806	1.22262	0.54495
Q8BMJ3	2.26	4.22	10.420	Eukaryotic translation initiation factor 1A, X-chromosomal	2	0.90405	1.11875	1.10564	1.23906
Q8BMK4	23.4	24.6	19.830	Cytoskeleton-associated protein 4	11	1.74923	1.74764	0.57142	1.00520
Q8BMP6	4	4.03	2.857	Golgi resident protein GCP60	2	0.94404	0.66601	1.05880	0.71272
Q8BMS1	29.3	31.66	17.690	Trifunctional enzyme subunit alpha, mitochondrial	15	1.70922	1.28247	0.58480	0.74142
Q8BP67	4.07	4.14	14.650	60S ribosomal protein L24	2	1.03007	1.65218	0.97037	1.60813
Q8BR63	4.74	4.74	10.140	Protein FAM177A1	2	0.92028	0.76460	1.08614	0.83289
Q8BRF7	6.06	7.98	5.164	Sec1 family domain-containing protein 1	4	0.85397	0.68457	1.17047	0.80454

Q8BT60	12	12.57	11.630	Copine-3	7	0.99774	1.44266	1.00181	1.45178
Q8BTM8	30.1	37.56	6.536	Filamin-A	14	1.51905	3.76507	0.65801	2.60996
Q8BTY2	7.41	8.73	3.482	Sodium bicarbonate cotransporter 3	4	0.77643	0.68935	1.28736	0.88513
Q8BTZ7	1.49	1.62	3.056	Mannose-1-phosphate guanyltransferase beta	1	0.75469	1.05349	1.32446	1.39959
Q8BU31	4.71	7.49	20.220	Ras-related protein Rap-2c	4	0.77401	0.80801	1.29139	1.05414
Q8BUV8	2	2.35	1.633	Protein GPR107	1	0.94398	0.68958	1.05887	0.73242
Q8BVW3	2.08	2.49	2.500	Tripartite motif-containing protein 14	1	0.83006	1.07367	1.20420	1.29789
Q8BWT1	13.4	13.59	16.880	3-ketoacyl-CoA thiolase, mitochondrial	6	0.90747	0.34424	1.10147	0.38261
Q8BXK9	2	4.09	6.773	Chloride intracellular channel protein 5	2	0.38914	0.39233	2.56863	1.01084
Q8BXN9	5.67	8.77	5.225	Transmembrane protein 87A	3	1.35779	0.89847	0.73616	0.66072
Q8BXZ1	5.28	6.61	7.237	Protein disulfide-isomerase TMX3	4	1.28323	1.50587	0.77894	1.17644
Q8C0Z1	7.73	7.77	5.225	Protein ITFG3	3	0.90020	0.44620	1.11037	0.49901
Q8C102	2	3.09	1.183	Polypeptide N-acetylgalactosaminyltransferase 5	1	1.12705	0.29745	0.88688	0.26461
Q8C129	2.18	2.72	1.268	Leucyl-cystinyl aminopeptidase	1	1.14434	1.23798	0.87348	1.08561
Q8C1B7	4.29	4.68	4.640	Septin-11	2	1.06622	2.64280	0.93747	2.52756
Q8C407	2.02	3.27	6.504	Protein YIPF4	2	1.01655	0.73366	0.98328	0.72362
Q8CAQ8	20.5	22.91	10.170	Mitochondrial inner membrane protein	7	1.25401	0.99659	0.79708	0.79180
Q8CBW3	4.91	5.49	3.742	Abl interactor 1	2	0.81509	1.09140	1.22631	1.34095
Q8CC88	9.76	11.68	2.257	von Willebrand factor A domain-containing protein 8	4	1.27378	0.66390	0.78472	0.52856
Q8CCS6	2.75	3	2.318	Polyadenylate-binding protein 2	1	0.84456	0.55693	1.18352	0.66419
Q8CGF7	1.73	2.85	0.818	Transcription elongation regulator 1	1	1.37299	2.71020	0.72801	1.97912
Q8CGK3	10.5	13.44	5.479	Lon protease homolog, mitochondrial	5	1.07341	0.67417	0.93119	0.63701
Q8CGP2	11.7	11.7	39.680	Histone H2B type 1-P	8	1.52591	5.16379	0.65505	3.43547
Q8CHG3	2.43	7.46	1.072	GRIP and coiled-coil domain-containing protein 2	2	1.14236	0.77737	0.87498	0.68269
Q8CI51	2.54	2.6	1.523	PDZ and LIM domain protein 5	1	0.73001	0.88499	1.36923	1.20631
Q8CI85	2	2.05	2.542	Carbonic anhydrase 12	1	0.98655	0.48794	1.01318	0.49589

Q8CIN4	2.09	2.63	1.718	Serine/threonine-protein kinase PAK 2	1	1.38744	1.93663	0.72043	1.39949
Q8JZN5	3.88	4.54	4.160	Acyl-CoA dehydrogenase family member 9, mitochondrial	3	0.85009	0.81772	1.17582	0.96639
Q8JZQ2	12.5	15.46	7.357	AFG3-like protein 2	6	1.30250	1.08325	0.76741	0.82383
Q8K021	3.76	4.04	5.621	Secretory carrier-associated membrane protein 1	2	1.09213	0.96677	0.91523	0.89255
Q8K0C5	14.8	14.83	40.720	Zymogen granule membrane protein 16	14	0.58103	0.18908	1.72031	0.34019
Q8K0C9	4.04	6.19	8.871	GDP-mannose 4,6 dehydratase	4	0.94650	0.96925	1.05605	1.03105
Q8K0J2	11.7	11.94	14.360	UDP-GlcNAc:betaGal beta-1,3-N-acetylglucosaminyltransferase 7	6	0.84262	0.88619	1.18624	1.05368
Q8K0Z7	1.43	1.64	3.061	Translational activator of cytochrome c oxidase 1	1	0.88793	0.64599	1.12572	0.72944
Q8K1Z0	2	2.06	2.875	Ubiquinone biosynthesis protein COQ9, mitochondrial	1	1.00097	0.60447	0.99858	0.60547
Q8K2B3	23.9	24.14	19.430	Succinate dehydrogenase [ubiquinone] flavoprotein subunit, mitochondrial	14	1.29725	0.73438	0.77052	0.56802
Q8K353	2	2	9.615	Cysteine-rich and transmembrane domain-containing protein 1	2	0.75625	0.71485	1.32173	0.94774
Q8K354	2	5.89	7.220	Carbonyl reductase [NADPH] 3	2	0.91904	0.45827	1.08760	0.49995
Q8K3J1	6.83	6.87	16.980	NADH dehydrogenase [ubiquinone] iron-sulfur protein 8, mitochondrial	4	1.31478	0.76790	0.76024	0.58838
Q8K419	34.3	34.94	44.790	Galectin-4	40	1.06957	1.60715	0.93454	1.44006
Q8K4L4	4.43	4.94	4.259	Protein POF1B	2	1.20769	0.85873	0.82766	0.69008
Q8K4Z5	4.66	7.3	2.781	Splicing factor 3A subunit 1	3	1.54627	1.25389	0.64643	0.81774
Q8QZR3	1.3	5.07	4.839	Pyrethroid hydrolase Ces2a	3	1.06688	0.49650	0.93689	0.46659
Q8QZT1	13.5	14.65	17.450	Acetyl-CoA acetyltransferase, mitochondrial	9	0.97539	0.54856	1.02477	0.56972
Q8R059	5.32	5.49	7.781	UDP-glucose 4-epimerase	3	0.84563	1.02141	1.18202	1.21186
Q8R081	5.26	5.35	4.437	Heterogeneous nuclear ribonucleoprotein L	3	1.30425	0.59356	0.76638	0.49540
Q8R088	2.16	2.31	4.561	Golgi phosphoprotein 3-like	1	1.13291	0.72143	0.88229	0.64420

Q8R0W0	4.87	11.65	2.902	Epiplakin	5	1.32692	0.85802	0.75329	0.64921
Q8R143	1.64	1.64	5.172	Pituitary tumor-transforming gene 1 protein-interacting protein	1	0.81314	0.90088	1.22925	1.11081
Q8R1F1	5.92	6.68	3.738	Niban-like protein 1	3	0.86822	1.38662	1.15126	1.59837
Q8R1F6	6.03	7.22	3.807	Protein HID1	3	0.93714	0.94755	1.06660	1.01802
Q8R1Q8	3.09	5.27	4.780	Cytoplasmic dynein 1 light intermediate chain 1	3	1.08359	1.09418	0.92244	1.02574
Q8R1S0	1.6	2.23	2.101	Ubiquinone biosynthesis monooxygenase COQ6	1	1.22412	0.38989	0.81655	0.31935
Q8R1V4	2.31	6.36	12.780	Transmembrane emp24 domain-containing protein 4	4	1.88602	0.85505	0.52998	0.45537
Q8R2S8	18.2	18.43	11.510	CD177 antigen	9	1.12833	2.06040	0.88587	1.85510
Q8R311	1.47	2.8	0.770	cTAGE family member 5	2	1.38727	0.85465	0.72052	0.61871
Q8R3G9	5.59	5.69	11.490	Tetraspanin-8	7	0.70106	0.50243	1.42576	0.72302
Q8R4G6	2.16	3.76	1.081	Alpha-1,6-mannosylglycoprotein 6-beta-N-acetylglucosaminyltransferase A	1	1.29767	1.36036	0.77027	1.05137
Q8VC28	2.32	2.58	3.096	Aldo-keto reductase family 1 member C13	1	0.65560	0.57615	1.52464	0.88112
Q8VCA5	4.06	4.36	4.138	Transmembrane protease serine 4	2	0.79737	0.70878	1.25356	0.89586
Q8VCC2	5.94	6.22	3.540	Liver carboxylesterase 1	2	1.39583	0.47304	0.71610	0.35266
Q8VCF1	12.1	12.69	19.850	Soluble calcium-activated nucleotidase 1	6	1.26027	0.75065	0.79313	0.60712
Q8VCN5	2	2.09	3.266	Cystathionine gamma-lyase	1	0.84812	0.13017	1.17856	0.15389
Q8VCR7	1.73	1.73	5.714	Alpha/beta hydrolase domain-containing protein 14B	1	0.59762	0.51856	1.67255	0.87004
Q8VCW8	19.5	21.12	14.800	Acyl-CoA synthetase family member 2, mitochondrial	9	0.76764	0.30685	1.30211	0.39873
Q8VCZ2	1.66	1.78	3.237	Transmembrane protein 45B	1	0.96277	0.86614	1.03820	0.90199
Q8VDD5	84.9	86.1	19.690	Myosin-9	41	1.46298	2.39197	0.68323	1.65234
Q8VDM4	4.48	6.15	3.304	26S proteasome non-ATPase regulatory subunit 2	4	0.84497	0.83222	1.18294	0.98538
Q8VDN2	87.1	88.01	40.570	Sodium/potassium-transporting ATPase subunit alpha-1	89	0.70414	0.39231	1.41954	0.55468
Q8VE70	3.87	5.32	12.740	Programmed cell death protein 10	3	0.74755	0.97253	1.33710	1.30792
Q8VED5	2.02	11.34	7.533	Keratin, type II cytoskeletal 79	9	0.70109	0.80067	1.42572	1.17480

Q8VEH8	1.89	3.11	2.692	Endoplasmic reticulum lectin 1	2	2.00009	1.12667	0.49975	0.56479
Q8VEJ9	5.44	7.22	7.323	Vacuolar protein sorting-associated protein 4A	4	0.88395	1.39721	1.13078	1.58445
Q8VEK3	9.73	10.56	4.875	Heterogeneous nuclear ribonucleoprotein U	4	1.08030	1.18658	0.92525	1.11809
Q8VEM8	8.63	8.88	7.563	Phosphate carrier protein, mitochondrial	3	1.43955	1.50109	0.69435	1.04740
Q8VHF2	10.3	11.78	5.535	Cadherin-related family member 5	4	0.45842	0.35170	2.18043	0.76009
Q8VIB3	3.96	5.64	8.511	Type 2 lactosamine alpha-2,3-sialyltransferase	3	0.95305	0.43882	1.04879	0.46341
Q8VIJ6	5.51	5.72	2.718	Splicing factor, proline- and glutamine-rich	2	1.18976	1.37417	0.84013	1.16185
Q91V12	2	2.21	2.887	Cytosolic acyl coenzyme A thioester hydrolase	1	0.89508	1.02093	1.11672	1.14360
Q91V14	2.25	2.86	0.967	Solute carrier family 12 member 5	1	1.07064	1.42081	0.93360	1.33054
Q91V41	17.1	17.09	37.670	Ras-related protein Rab-14	9	0.95013	0.69492	1.05202	0.73342
Q91V61	2.04	2.08	4.050	Sideroflexin-3	1	1.85518	3.02025	0.53879	1.63228
Q91V76	2.25	2.44	3.810	Ester hydrolase C11orf54 homolog	1	0.55078	0.24209	1.81478	0.44685
Q91V92	2.05	3.13	0.733	ATP-citrate synthase	1	0.90575	0.78746	1.10356	0.87168
Q91VA1	5.43	7.74	4.950	Choline transporter-like protein 4	4	0.56810	0.43631	1.75946	0.76729
Q91VD9	32.5	34.77	21.730	NADH-ubiquinone oxidoreductase 75 kDa subunit, mitochondrial	16	1.24876	0.74290	0.80043	0.59777
Q91VM5	4.89	4.92	9.021	RNA binding motif protein, X-linked-like-1	3	1.55646	2.01001	0.64220	1.27989
Q91VM9	5.62	5.69	11.820	Inorganic pyrophosphatase 2, mitochondrial	3	0.68641	0.29072	1.45620	0.41922
Q91VR2	13.8	16.04	24.160	ATP synthase subunit gamma, mitochondrial	7	1.38179	1.34781	0.72338	0.94834
Q91VR8	1.79	1.81	14.670	Protein BRICK1	1	0.99846	2.67218	1.00109	2.70260
Q91VS7	1.9	1.94	8.387	Microsomal glutathione S-transferase 1	1	1.69028	0.81023	0.59135	0.48061
Q91VT4	2.48	2.62	2.119	Carbonyl reductase family member 4	1	0.79681	0.90687	1.25444	1.14111
Q91VW3	2.86	3.5	16.130	SH3 domain-binding glutamic acid-rich-like protein 3	1	0.78717	0.89475	1.26981	1.14822
Q91W53	1.52	1.52	8.029	Golgin subfamily A member 7	1	0.72851	0.86390	1.37205	1.18895
Q91W90	12.5	14.86	17.750	Thioredoxin domain-containing protein 5	6	0.85157	0.51502	1.17377	0.61120

Q91WD5	9.26	9.61	9.503	NADH dehydrogenase [ubiquinone] iron-sulfur protein 2, mitochondrial	4	1.05479	0.87542	0.94763	0.83194
Q91WG0	10.3	10.56	9.091	Acylcarnitine hydrolase	5	1.42815	0.36719	0.69990	0.26048
Q91WG8	6.92	7.15	5.263	Bifunctional UDP-N-acetylglucosamine 2-epimerase/N-acetylmannosamine kinase	3	0.98877	0.89364	1.01090	0.90483
Q91WK2	2.05	2.16	3.409	Eukaryotic translation initiation factor 3 subunit H	1	0.85526	0.81179	1.16871	0.95164
Q91WL0	5.79	6.85	2.667	Epidermal growth factor receptor kinase substrate 8-like protein 3	2	0.49486	0.84265	2.01986	1.71302
Q91WQ9	1.63	1.71	5.882	Calmodulin-like protein 4	1	0.55513	0.54236	1.80056	0.98300
Q91WS0	4.07	4.42	20.370	CDGSH iron-sulfur domain-containing protein 1	2	1.32966	0.74638	0.75174	0.56311
Q91X72	1.96	2.5	1.957	Hemopexin	1	1.39476	1.75782	0.71665	1.26369
Q91X97	2	2.39	6.736	Neurocalcin-delta	1	1.10549	1.25514	0.90417	1.13835
Q91XA2	25	25.24	31.040	Golgi membrane protein 1	19	1.04498	0.65761	0.95652	0.62433
Q91Y74	9.99	10.79	15.020	CMP-N-acetylneuraminate-beta-galactosamide-alpha-2,3-sialyltransferase 4	6	0.93565	0.59985	1.06830	0.64462
Q91Y97	5.14	5.64	7.967	Fructose-bisphosphate aldolase B	3	0.89158	0.59093	1.12110	0.66369
Q91YH5	1.4	1.83	2.218	Atlastin-3	1	1.24797	1.78971	0.80094	1.43369
Q91YQ5	26.9	27.63	22.200	Dolichyl-diphosphooligosaccharide--protein glycosyltransferase subunit 1	13	1.82021	1.23717	0.54914	0.66200
Q91YR1	3.02	4.51	6.571	Twinfilin-1	3	0.77956	0.90083	1.28220	1.15697
Q91YT0	15.9	20.1	19.400	NADH dehydrogenase [ubiquinone] flavoprotein 1, mitochondrial	11	1.25027	1.17954	0.79947	0.94151
Q91YT8	2	2.5	1.493	Transmembrane protein 63A	1	0.96025	0.52530	1.04093	0.54848
Q91YW3	3.24	6.58	3.175	DnaJ homolog subfamily C member 3	3	1.31928	0.93244	0.75765	0.73055
Q91ZA3	3.43	3.83	2.901	Propionyl-CoA carboxylase alpha chain, mitochondrial	2	1.03987	0.73693	0.96123	0.71801
Q91ZJ5	9.94	10.78	7.480	UTP--glucose-1-phosphate uridylyltransferase	5	0.92136	0.63350	1.08487	0.69189

Q91ZR1	2	3.24	10.330	Ras-related protein Rab-4B	3	0.94936	1.18299	1.05287	1.24936
Q91ZX7	3.05	4.72	0.286	Prolow-density lipoprotein receptor-related protein 1	1	1.15033	1.41324	0.86893	1.24264
Q921E2	2	2	5.670	Ras-related protein Rab-31	1	1.02977	1.28534	0.97065	1.25145
Q921F2	4.1	4.32	5.797	TAR DNA-binding protein 43	2	0.91642	0.79079	1.09071	0.86681
Q921G7	10.2	12.58	10.390	Electron transfer flavoprotein-ubiquinone oxidoreductase, mitochondrial	7	1.22365	0.97023	0.81686	0.79636
Q921H8	3.66	4.78	6.840	3-ketoacyl-CoA thiolase A, peroxisomal	4	1.23089	0.91095	0.81206	0.73723
Q921I1	14.7	15.43	10.330	Serotransferrin	7	1.21254	1.65951	0.82434	1.36210
Q921W0	1.66	1.7	3.571	Charged multivesicular body protein 1a	1	0.99582	1.05147	1.00375	1.05955
Q921X9	11	11.26	8.897	Protein disulfide-isomerase A5	5	1.38367	0.85772	0.72239	0.60800
Q922B2	2.25	2.52	1.996	Aspartate--tRNA ligase, cytoplasmic	1	1.15607	1.52859	0.86462	1.32284
Q922D8	3.46	4.12	2.032	C-1-tetrahydrofolate synthase, cytoplasmic	2	0.93807	1.03066	1.06554	1.10238
Q922Q1	4.3	4.5	7.101	MOSC domain-containing protein 2, mitochondrial	2	1.02461	1.04243	0.97555	1.01817
Q922Q4	3.86	3.86	7.500	Pyrroline-5-carboxylate reductase 2	2	1.13831	0.82138	0.87810	0.72463
Q922Q8	7.03	8.56	9.446	Leucine-rich repeat-containing protein 59	4	1.64661	1.53471	0.60704	0.93480
Q922R8	17.9	18.66	21.590	Protein disulfide-isomerase A6	9	1.34216	0.65684	0.74473	0.48771
Q93092	5.07	5.64	9.199	Transaldolase	3	0.84197	1.07434	1.18717	1.27820
Q99020	2.04	2.33	4.912	Heterogeneous nuclear ribonucleoprotein A/B	1	0.47601	0.41040	2.09983	0.86441
Q99J99	8.38	8.64	14.480	3-mercaptopyruvate sulfurtransferase	4	0.84748	0.22525	1.17944	0.27195
Q99JB2	5.72	6.12	8.782	Stomatin-like protein 2, mitochondrial	3	1.32009	1.13538	0.75718	0.86273
Q99JG3	5.39	7.83	11.040	Annexin A13	4	0.48000	0.29781	2.08240	0.61610
Q99JI4	2.19	3.22	2.314	26S proteasome non-ATPase regulatory subunit 6	1	0.75699	0.75839	1.32043	1.00828
Q99JI6	7.78	8.5	23.370	Ras-related protein Rap-1b	6	0.93747	1.55446	1.06622	1.64855
Q99JR1	1.85	2.05	4.037	Sideroflexin-1	1	0.60213	0.88467	1.66004	1.46061
Q99JT2	1.83	2.87	1.683	Serine/threonine-protein kinase MST4	1	0.78019	1.89626	1.28116	2.43687
Q99JW5	21.4	22.91	37.140	Epithelial cell adhesion molecule	15	1.03249	0.93571	0.96810	0.84799

Q99JX3	8.03	8.14	9.534	Golgi reassembly-stacking protein 2	4	0.94016	0.85329	1.06317	0.92055
Q99JY0	19.4	19.81	18.530	Trifunctional enzyme subunit beta, mitochondrial	10	1.44200	1.18932	0.69317	0.83194
Q99JY9	9.55	10.4	13.400	Actin-related protein 3	5	0.96746	1.03530	1.03318	1.07735
Q99K01	14.2	15.14	12.960	Pyridoxal-dependent decarboxylase domain-containing protein 1	8	1.11187	0.58988	0.89898	0.53603
Q99K30	4.01	4.78	2.743	Epidermal growth factor receptor kinase substrate 8-like protein 2	2	0.77899	0.96746	1.28313	1.23909
Q99K48	3.74	4.35	7.188	Non-POU domain-containing octamer-binding protein	3	1.38269	1.14593	0.72290	0.83558
Q99KE1	2.87	3.33	3.565	NAD-dependent malic enzyme, mitochondrial	2	0.88764	0.54937	1.12608	0.62054
Q99KF1	9.31	9.41	17.870	Transmembrane emp24 domain-containing protein 9	7	1.45342	0.86381	0.68772	0.59563
Q99KI0	43.6	46.42	28.850	Aconitate hydratase, mitochondrial	23	0.97766	0.58908	1.02239	0.60994
Q99KJ8	5.74	5.86	8.209	Dynactin subunit 2	3	0.80395	0.74304	1.24331	0.92964
Q99KK2	1.37	1.58	2.778	N-acylneuraminate cytidylyltransferase	1	1.14811	0.86252	0.87060	0.75653
Q99KN9	5.37	5.45	4.596	Clathrin interactor 1	3	1.27899	0.83891	0.78151	0.66145
Q99KQ4	1.5	2.11	2.037	Nicotinamide phosphoribosyltransferase	1	0.78914	1.11813	1.26664	1.41972
Q99KR7	4.2	5.96	10.190	Peptidyl-prolyl cis-trans isomerase F, mitochondrial	3	1.02432	0.41882	0.97582	0.40915
Q99KV1	5.61	5.64	6.704	DnaJ homolog subfamily B member 11	2	1.42843	0.82502	0.69975	0.57736
Q99L04	3.57	3.57	2.556	Dehydrogenase/reductase SDR family member 1	1	1.29351	1.46874	0.77274	1.14603
Q99L45	2.01	3.02	4.834	Eukaryotic translation initiation factor 2 subunit 2	2	1.08446	1.28996	0.92170	1.19261
Q99L47	2.2	2.27	2.695	Hsc70-interacting protein	1	0.66507	1.30311	1.50294	1.97133
Q99LB2	11.9	12.3	17.560	Dehydrogenase/reductase SDR family member 4	5	1.48848	1.22114	0.67153	0.82114
Q99LC3	6.75	6.75	9.859	NADH dehydrogenase [ubiquinone] 1 alpha subcomplex subunit 10, mitochondrial	3	1.29290	1.13184	0.77311	0.88067
Q99LC5	21.8	23.11	36.040	Electron transfer flavoprotein subunit alpha, mitochondrial	15	1.04390	0.44632	0.95751	0.45148
Q99LF4	10.2	10.44	10.890	tRNA-splicing ligase RtcB homolog	5	1.01621	0.86486	1.02157	0.89132

Q99LJ1	2.05	2.22	1.549	Tissue alpha-L-fucosidase	1	0.86750	0.69374	1.15222	0.80180
Q99LM2	3.92	4.37	4.175	CDK5 regulatory subunit-associated protein 3	3	1.49091	0.91131	0.67043	0.61158
Q99LP6	2.7	4.17	5.069	GrpE protein homolog 1, mitochondrial	2	0.78179	0.66266	1.27854	0.87284
Q99LX0	5.21	5.32	15.870	Protein DJ-1	3	0.82388	1.01727	1.21323	1.26338
Q99P58	2.67	4.77	14.220	Ras-related protein Rab-27B	4	0.79703	0.70382	1.25409	0.96459
Q99P72	6.57	7.32	2.496	Reticulon-4	3	1.76289	3.11579	0.56700	1.67285
Q99PL5	30.2	35.62	9.283	Ribosome-binding protein 1	18	1.36315	1.10324	0.73326	0.81565
Q99PT1	4.33	4.4	15.200	Rho GDP-dissociation inhibitor 1	2	0.81983	0.87845	1.21922	1.08477
Q9CPP6	7.65	7.95	32.760	NADH dehydrogenase [ubiquinone] 1 alpha subcomplex subunit 5	4	1.23640	0.66649	0.80844	0.54247
Q9CPQ1	4.94	5.07	26.320	Cytochrome c oxidase subunit 6C	3	1.17097	0.70237	0.85361	0.60261
Q9CPQ8	3.55	3.88	31.070	ATP synthase subunit g, mitochondrial	2	1.04755	1.31521	0.95418	1.25880
Q9CPT4	2.49	2.58	9.036	UPF0556 protein C19orf10 homolog	2	1.37832	0.91426	0.72519	0.66865
Q9CPU0	1.3	1.58	4.891	Lactoylglutathione lyase	1	0.64830	0.70036	1.54181	1.07497
Q9CPW4	6.74	6.84	37.750	Actin-related protein 2/3 complex subunit 5	5	1.05463	1.01242	0.94777	0.95708
Q9CPY7	3.24	6.69	4.432	Cytosol aminopeptidase	4	0.81590	0.66934	1.22509	0.82553
Q9CQ10	3.67	3.78	7.589	Charged multivesicular body protein 3	2	0.97198	1.34465	1.02837	1.38799
Q9CQ19	2.03	10.33	26.740	Myosin regulatory light polypeptide 9	6	2.82354	4.56453	0.35401	1.63672
Q9CQ22	2.41	2.52	8.075	Ragulator complex protein LAMTOR1	1	1.21473	1.69882	0.82286	1.40219
Q9CQ54	2.79	3.12	18.330	NADH dehydrogenase [ubiquinone] 1 subunit C2	2	1.47582	1.04304	0.67729	0.80246
Q9CQ62	5.72	5.96	8.657	2,4-dienoyl-CoA reductase, mitochondrial	3	1.07401	0.49105	0.93067	0.46146
Q9CQ69	4.89	4.89	25.610	Cytochrome b-c1 complex subunit 8	2	1.45171	1.16732	0.68853	0.78166
Q9CQ75	5.32	5.49	22.220	NADH dehydrogenase [ubiquinone] 1 alpha subcomplex subunit 2	3	1.04643	0.79322	0.95521	0.76037
Q9CQ92	2.17	2.22	7.237	Mitochondrial fission 1 protein	1	2.21914	2.09908	0.45042	0.98703

Q9CQA3	13.5	14.17	22.700	Succinate dehydrogenase [ubiquinone] iron-sulfur subunit, mitochondrial	7	1.03424	0.70907	0.96646	0.67900
Q9CQB5	1.59	1.79	5.185	CDGSH iron-sulfur domain-containing protein 2	1	1.37401	0.91026	0.72747	0.66434
Q9CQC6	3.19	6.3	5.489	Basic leucine zipper and W2 domain-containing protein 1	3	1.21628	1.47216	0.82181	1.21640
Q9CQC7	2.72	2.82	16.280	NADH dehydrogenase [ubiquinone] 1 beta subcomplex subunit 4	2	1.17803	1.23638	0.84849	1.03953
Q9CQC9	4.15	4.48	12.630	GTP-binding protein SAR1b	3	1.13802	1.08663	0.87832	0.95060
Q9CQD1	9.2	9.32	22.330	Ras-related protein Rab-5A	5	1.35391	1.25413	0.73827	0.98207
Q9CQE8	2.43	2.43	3.689	UPF0568 protein C14orf166 homolog	1	0.88945	0.66717	1.12378	0.75213
Q9CQH3	6.08	6.19	14.290	NADH dehydrogenase [ubiquinone] 1 beta subcomplex subunit 5, mitochondrial	3	1.37178	1.21338	0.72865	0.88965
Q9CQM5	1.96	2.21	7.317	Thioredoxin domain-containing protein 17	1	0.57497	0.77646	1.73845	1.35398
Q9CQN1	6.3	10.16	5.807	Heat shock protein 75 kDa, mitochondrial	5	1.22934	0.77386	0.81308	0.62605
Q9CQQ7	9.68	10.43	17.580	ATP synthase F(0) complex subunit B1, mitochondrial	6	1.03061	0.91171	0.96986	0.88632
Q9CQR2	4.25	4.74	20.480	40S ribosomal protein S21	2	1.25165	1.06519	0.79859	0.85800
Q9CQR4	3.29	3.29	15.710	Acyl-coenzyme A thioesterase 13	2	0.93750	0.84669	1.06619	0.92322
Q9CQS8	2	2	10.420	Protein transport protein Sec61 subunit beta	1	1.84207	1.34629	0.54262	0.73278
Q9CQU0	2	2.09	5.294	Thioredoxin domain-containing protein 12	1	1.49648	0.83021	0.66794	0.55623
Q9CQV8	4.01	17.16	30.490	14-3-3 protein beta/alpha	11	0.66727	1.03845	1.49797	1.56097
Q9CQW1	2	2.21	3.535	Synaptobrevin homolog YKT6	1	1.02088	1.32660	0.97911	1.30288
Q9CQW2	3.65	3.86	12.370	ADP-ribosylation factor-like protein 8B	3	1.30601	2.06044	0.76535	1.63723
Q9CQX2	5.96	6.39	21.230	Cytochrome b5 type B	3	1.40693	0.76202	0.76375	0.58832
Q9CQZ5	2.1	3.04	12.210	NADH dehydrogenase [ubiquinone] 1 alpha subcomplex subunit 6	2	1.01848	0.77129	0.98142	0.75928
Q9CR09	2	2.04	5.389	Ubiquitin-fold modifier-conjugating enzyme 1	1	0.88358	1.36921	1.13125	1.55368
Q9CR21	4.9	5.09	12.820	Acyl carrier protein, mitochondrial	2	1.13906	0.93893	0.87752	0.83068

Q9CR26	1.3	1.39	2.589	Vacuolar protein sorting-associated protein VTA1 homolog	1	0.92730	1.46841	1.07791	1.58767
Q9CR51	2	2	9.322	V-type proton ATPase subunit G 1	1	0.58638	0.63413	1.70461	1.08426
Q9CR57	2.17	2.45	5.991	60S ribosomal protein L14	1	0.74353	1.68402	1.34433	2.28737
Q9CR61	5.64	5.75	23.360	NADH dehydrogenase [ubiquinone] 1 beta subcomplex subunit 7	3	1.02422	1.19592	0.97591	1.16766
Q9CR67	1.45	1.52	4.858	Transmembrane protein 33	1	1.50281	2.01775	0.66512	1.34617
Q9CR68	10.6	10.74	13.870	Cytochrome b-c1 complex subunit Rieske, mitochondrial	6	1.14256	0.64700	0.87484	0.56502
Q9CR98	2.27	2.43	5.797	Protein FAM136A	1	1.16073	0.92338	0.86114	0.79886
Q9CRA5	3.01	3.5	9.396	Golgi phosphoprotein 3	2	0.96020	0.88738	1.04098	0.92709
Q9CRB9	8.61	10.97	17.620	Coiled-coil-helix-coiled-coil-helix domain-containing protein 3, mitochondrial	6	1.25293	0.82952	0.79777	0.66243
Q9CRY7	2.5	3.34	3.822	Glycerophosphodiester phosphodiesterase domain-containing protein 1	1	1.09280	1.02105	0.91467	0.94693
Q9CVB6	17.8	20.09	29.000	Actin-related protein 2/3 complex subunit 2	11	0.98881	1.25933	1.01086	1.27476
Q9CWJ9	2.83	3.25	1.689	Bifunctional purine biosynthesis protein PURH	1	1.31598	1.79762	0.75955	1.36957
Q9CWS0	5.14	6.07	10.180	N(G),N(G)-dimethylarginine dimethylaminohydrolase 1	3	0.78221	0.89809	1.27786	1.14442
Q9CWZ3	2.37	2.37	4.598	RNA-binding protein 8A	1	1.51305	1.18688	0.66062	0.78687
Q9CWZ7	9.89	10.55	15.060	Gamma-soluble NSF attachment protein	5	0.83535	1.01897	1.19657	1.22262
Q9CX00	8.63	9.09	9.669	IST1 homolog	5	0.96246	1.27657	1.03854	1.32490
Q9CXD6	2	2.42	2.059	Mitochondrial calcium uniporter regulator 1	1	1.12048	0.92772	0.89207	0.83014
Q9CXI0	1.57	1.96	3.058	2-methoxy-6-polyprenyl-1,4-benzoquinol methylase, mitochondrial	1	1.04589	0.34631	0.95570	0.33198
Q9CXI5	10.1	10.39	29.610	Mesencephalic astrocyte-derived neurotrophic factor	5	0.95471	0.57038	1.04697	0.58886
Q9CXR1	3.97	4.06	5.325	Dehydrogenase/reductase SDR family member 7	2	1.36138	1.33745	0.73422	0.98911
Q9CXW3	1.62	2.32	3.057	Calcyclin-binding protein	1	1.35056	1.54326	0.74010	1.14568

Q9CXW4	4.41	5.57	15.730	60S ribosomal protein L11	3	1.10529	1.52272	0.90433	1.38210
Q9CXZ1	8.22	8.36	25.140	NADH dehydrogenase [ubiquinone] iron-sulfur protein 4, mitochondrial	5	1.43948	0.87730	0.69439	0.68227
Q9CY50	3.75	3.8	6.643	Translocon-associated protein subunit alpha	3	1.48532	1.06404	0.67295	0.72098
Q9CY58	2.03	2.32	1.474	Plasminogen activator inhibitor 1 RNA-binding protein	1	0.68195	1.10929	1.46572	1.63091
Q9CYN2	2.54	2.98	2.655	Signal peptidase complex subunit 2	1	1.12471	0.80710	0.88872	0.71433
Q9CYR0	2.18	2.24	5.263	Single-stranded DNA-binding protein, mitochondrial	1	1.08413	1.57515	0.92199	1.45673
Q9CYW4	3.34	3.66	6.773	Haloacid dehalogenase-like hydrolase domain-containing protein 3	2	0.45535	0.21246	2.19512	0.48204
Q9CYZ2	2.94	3.03	13.640	Tumor protein D54	2	1.24336	1.16878	0.80391	0.93726
Q9CZ13	20.2	21.55	25.210	Cytochrome b-c1 complex subunit 1, mitochondrial	11	1.25163	0.68096	0.79860	0.53846
Q9CZB0	2	2	7.692	Succinate dehydrogenase cytochrome b560 subunit, mitochondrial	1	2.03430	1.24883	0.49135	0.61550
Q9CZD3	5.64	5.7	3.567	Glycine--tRNA ligase	3	0.74612	0.87404	1.33966	1.17814
Q9CZS1	23.3	28.15	27.170	Aldehyde dehydrogenase X, mitochondrial	14	0.72594	0.29555	1.37690	0.40961
Q9CZU6	19.3	20.19	17.670	Citrate synthase, mitochondrial	10	0.95156	0.63821	1.05044	0.67248
Q9CZX8	10.7	12.09	33.100	40S ribosomal protein S19	7	0.73166	1.11456	1.36614	1.53275
Q9CZY3	4.46	4.49	11.560	Ubiquitin-conjugating enzyme E2 variant 1	2	0.74032	1.16119	1.35015	1.57124
Q9D023	1.55	1.64	5.512	Mitochondrial pyruvate carrier 2	1	0.97766	0.82583	1.02239	0.84692
Q9D051	11.8	12.27	19.220	Pyruvate dehydrogenase E1 component subunit beta, mitochondrial	6	0.86659	0.44260	1.15343	0.51231
Q9D0E1	12.7	14.52	11.660	Heterogeneous nuclear ribonucleoprotein M	8	1.04765	0.85555	0.95409	0.81663
Q9D0F3	9.13	10.89	13.150	Protein ERGIC-53	6	1.34526	0.91107	0.74302	0.68947
Q9D0F9	1.59	2.1	1.423	Phosphoglucomutase-1	1	0.66604	0.75954	1.50074	1.14337
Q9D0I4	2.01	2.48	2.990	Syntaxin-17	1	0.85363	0.59656	1.17095	0.70069

Q9D0K2	11.8	12.45	12.120	Succinyl-CoA:3-ketoacid coenzyme A transferase 1, mitochondrial	6	1.06551	0.59326	0.93810	0.56468
Q9D0M3	6.6	6.71	10.460	Cytochrome c1, heme protein, mitochondrial	5	1.17948	0.83736	0.84745	0.75402
Q9D0M5	2	2.28	7.865	Dynein light chain 2, cytoplasmic	1	0.83370	0.62974	1.19894	0.75733
Q9D154	16	16.11	24.540	Leukocyte elastase inhibitor A	9	0.77634	0.65044	1.28752	0.83906
Q9D1A2	3.07	3.2	4.842	Cytosolic non-specific dipeptidase	2	0.62495	0.61772	1.59941	1.00004
Q9D1D4	9.6	13.56	24.660	Transmembrane emp24 domain-containing protein 10	9	1.63589	0.88531	0.61101	0.54182
Q9D1G1	4.32	12.7	30.850	Ras-related protein Rab-1B	8	1.26282	0.93565	0.79153	0.75127
Q9D1M7	2.15	2.49	10.450	Peptidyl-prolyl cis-trans isomerase FKBP11	2	1.65285	0.87058	0.60474	0.53446
Q9D1Q6	9.21	9.95	9.852	Endoplasmic reticulum resident protein 44	4	1.11348	0.74140	0.89768	0.67133
Q9D279	20.1	21.26	13.890	Mitotic interactor and substrate of PLK1	9	0.66164	0.57364	1.51072	0.85907
Q9D2G2	5.74	6.1	5.947	Dihydrolipoyllysine-residue succinyltransferase component of 2-oxoglutarate dehydrogenase complex, mitochondrial	3	1.00348	0.55587	0.99609	0.55847
Q9D2Q8	12.6	12.96	65.380	Protein S100-A14	9	1.28540	2.57531	0.77762	2.01834
Q9D2R6	2.01	2.01	9.259	Cytochrome c oxidase assembly protein 3 homolog, mitochondrial	1	1.07102	0.98229	0.93327	0.91955
Q9D306	6.58	6.87	6.067	Alpha-1,3-mannosyl-glycoprotein 4-beta-N-acetylglucosaminyltransferase C	3	1.37522	0.63240	0.72683	0.48362
Q9D309	9.59	9.59	18.720	Protein FAM3B	5	1.34228	0.67488	0.74467	0.50798
Q9D311	2	3.14	3.750	Dual oxidase maturation factor 2	2	0.92105	1.59259	1.08523	1.73363
Q9D312	45.9	49.39	55.680	Keratin, type I cytoskeletal 20	45	1.42420	0.38206	0.70183	0.28003
Q9D3D9	9.29	9.7	26.790	ATP synthase subunit delta, mitochondrial	8	1.07733	0.74669	0.92781	0.69573
Q9D3P8	2.32	2.48	6.803	Plasminogen receptor (KT)	1	1.35506	0.99903	0.73764	0.73511
Q9D4P0	2	2.25	6.145	ADP-ribosylation factor-like protein 5B	1	1.30759	0.98812	0.76442	0.75767
Q9D554	4.06	4.46	4.192	Splicing factor 3A subunit 3	2	1.30882	1.61623	0.76370	1.24684

Q9D5T0	2.01	4.25	4.709	ATPase family AAA domain-containing protein 1	2	1.68729	1.68337	0.59240	1.00030
Q9D646	15.6	15.92	20.920	Keratin, type I cuticular Ha4	8	3.21031	0.62207	0.31136	0.19476
Q9D6J6	10.2	11.1	19.350	NADH dehydrogenase [ubiquinone] flavoprotein 2, mitochondrial	6	1.01066	0.71543	0.98901	0.70509
Q9D6R2	18.5	19.11	19.950	Isocitrate dehydrogenase [NAD] subunit alpha, mitochondrial	9	0.88097	0.50371	1.13461	0.59706
Q9D6Y7	4	4.06	8.584	Mitochondrial peptide methionine sulfoxide reductase	2	0.65906	0.71075	1.51663	1.08005
Q9D7N9	5.51	5.51	7.711	Adipocyte plasma membrane-associated protein	3	1.39758	0.96871	0.71520	0.69654
Q9D7P6	3.01	3.32	10.120	Iron-sulfur cluster assembly enzyme ISCU, mitochondrial	2	0.84084	0.54175	1.18875	0.64834
Q9D7T1	4.79	5.07	12.170	Rab15 effector protein	4	0.65990	0.49096	1.51470	0.74616
Q9D7Z6	37.2	37.71	24.420	Calcium-activated chloride channel regulator 1	18	0.46777	0.19448	2.13686	0.40723
Q9D816	28.3	29.09	31.020	Cytochrome P450 2C55	18	1.98770	0.39825	0.50287	0.24568
Q9D819	8.87	9.77	16.260	Inorganic pyrophosphatase	4	0.61074	0.51691	1.63663	0.85925
Q9D855	13	13.16	54.050	Cytochrome b-c1 complex subunit 7	9	1.20925	0.63620	0.82658	0.49869
Q9D868	2	5.32	11.700	Peptidyl-prolyl cis-trans isomerase H	3	1.88656	2.55712	0.52983	1.35900
Q9D869	1.36	1.61	10.200	Calcineurin B homologous protein 2	1	0.49578	0.16304	2.01612	0.34300
Q9D880	2.06	2.12	1.983	Mitochondrial import inner membrane translocase subunit TIM50	1	1.22254	1.33625	0.81760	1.09623
Q9D898	3.96	4.09	16.340	Actin-related protein 2/3 complex subunit 5-like protein	2	1.08217	0.86770	0.92366	0.80616
Q9D8B3	6.83	8.29	16.520	Charged multivesicular body protein 4b	4	1.24919	1.59306	0.80016	1.28556
Q9D8C4	2.53	2.87	2.448	Interferon-induced 35 kDa protein homolog	1	0.96688	1.10075	1.03379	1.13717
Q9D8E6	3.41	3.75	4.296	60S ribosomal protein L4	2	1.12084	1.35965	0.89179	1.21019
Q9D8N0	6.07	6.21	5.263	Elongation factor 1-gamma	5	1.15907	1.00690	0.86237	0.87292
Q9D8T0	2.19	4.25	9.130	Protein FAM3A	2	1.10633	0.91614	0.90348	0.83184
Q9D8T2	2.67	2.84	2.053	Gasdermin-D	1	0.96892	0.74261	1.03162	0.77826

Q9D8U8	2.35	2.54	3.713	Sorting nexin-5	1	2.83729	3.94470	0.35229	1.39395
Q9D8V0	2.08	3.58	2.646	Minor histocompatibility antigen H13	2	1.44984	1.75685	0.68942	1.21216
Q9D8V7	3.35	3.44	8.854	Signal peptidase complex catalytic subunit SEC11C	2	1.21979	0.75521	0.81945	0.62075
Q9D8W5	2.22	2.54	1.535	26S proteasome non-ATPase regulatory subunit 12	1	1.10115	1.25138	0.90773	1.13941
Q9D8W7	4.85	4.94	8.442	OCIA domain-containing protein 2	2	1.30039	0.74580	0.76866	0.54934
Q9D8Y0	2.89	3.14	9.167	EF-hand domain-containing protein D2	2	0.65747	0.86641	1.52031	1.31404
Q9DAS9	6.37	6.37	56.940	Guanine nucleotide-binding protein G(I)/G(S)/G(O) subunit gamma-12	3	0.75556	0.69170	1.32292	0.95797
Q9DAX2	3.72	3.72	6.159	Lipid phosphate phosphohydrolase 2	2	0.75373	0.59600	1.32614	0.79027
Q9DB05	12.4	12.52	20.680	Alpha-soluble NSF attachment protein	6	1.18374	1.13415	0.84440	0.95788
Q9DB15	2.38	2.65	4.478	39S ribosomal protein L12, mitochondrial	1	1.15781	0.97674	0.86331	0.84582
Q9DB20	12.3	12.48	28.170	ATP synthase subunit O, mitochondrial	7	1.39011	1.38782	0.71905	0.98902
Q9DB77	25.9	26	35.100	Cytochrome b-c1 complex subunit 2, mitochondrial	19	1.47232	0.96874	0.67890	0.65117
Q9DBG3	24	25.75	13.870	AP-2 complex subunit beta	15	0.93772	1.91094	1.06593	2.03921
Q9DBG6	14.2	15.69	13.000	Dolichyl-diphosphooligosaccharide--protein glycosyltransferase subunit 2	10	1.97432	1.16405	0.50628	0.59878
Q9DBG7	2.03	4.03	2.201	Signal recognition particle receptor subunit alpha	2	1.38304	1.78089	0.72272	1.29104
Q9DBG9	4.45	4.45	17.740	Tax1-binding protein 3	3	0.96811	1.51315	1.03248	1.57038
Q9DBH5	9.13	9.46	13.130	Vesicular integral-membrane protein VIP36	5	1.38151	1.11265	0.72352	0.80548
Q9DBJ1	4.36	4.62	10.630	Phosphoglycerate mutase 1	3	0.71037	0.88642	1.40709	1.25159
Q9DBJ3	7.28	7.76	6.420	Brain-specific angiogenesis inhibitor 1-associated protein 2-like protein 1	4	0.65192	0.80305	1.53325	1.23587
Q9DBP5	4.52	6.11	12.240	UMP-CMP kinase	3	0.69014	0.44099	1.44833	0.63744
Q9DBS2	2	2.13	3.008	Tumor protein p63-regulated gene 1-like protein	1	0.85824	0.94350	1.16465	1.10222
Q9DBS5	3.7	3.87	2.908	Kinesin light chain 4	2	1.04193	0.75022	0.95933	0.71275
Q9DBX6	2.02	2.48	1.796	Cytochrome P450 2S1	1	2.14260	0.64507	0.46651	0.30186

Q9DC16	5.85	6.1	11.380	Endoplasmic reticulum-Golgi intermediate compartment protein 1	3	1.36767	1.12589	0.73084	0.81252
Q9DC29	4.08	6.94	3.444	ATP-binding cassette sub-family B member 6, mitochondrial	3	1.06763	0.81703	0.93623	0.76662
Q9DC51	1.79	9.78	14.690	Guanine nucleotide-binding protein G(k) subunit alpha	6	0.96815	1.02316	1.03244	1.04053
Q9DCC4	2.04	2.06	4.015	Pyrroline-5-carboxylate reductase 3	1	0.74083	0.75908	1.34923	1.02732
Q9DCD0	4.62	5.12	3.313	6-phosphogluconate dehydrogenase, decarboxylating	2	0.67800	1.22210	1.47426	1.80967
Q9DCH4	3.97	4.14	6.371	Eukaryotic translation initiation factor 3 subunit F	2	0.97103	0.63863	1.02937	0.65933
Q9DCI3	2	2.49	4.255	MLN64 N-terminal domain homolog	1	1.11617	1.88552	0.89552	1.69371
Q9DCJ5	4.02	4.24	12.210	NADH dehydrogenase [ubiquinone] 1 alpha subcomplex subunit 8	2	1.06146	0.84896	0.94168	0.80048
Q9DCM0	3.82	4.66	11.020	Persulfide dioxygenase ETHE1, mitochondrial	2	0.57803	0.19822	1.72924	0.32591
Q9DCM2	7.19	7.83	18.140	Glutathione S-transferase kappa 1	4	1.77576	0.81439	0.63696	0.52346
Q9DCS3	4.23	4.43	6.166	Trans-2-enoyl-CoA reductase, mitochondrial	2	0.71807	0.45967	1.39200	0.64247
Q9DCS9	4.75	5.73	23.860	NADH dehydrogenase [ubiquinone] 1 beta subcomplex subunit 10	3	1.40292	0.82086	0.71248	0.58488
Q9DCT2	10.6	10.7	21.670	NADH dehydrogenase [ubiquinone] iron-sulfur protein 3, mitochondrial	6	1.16838	0.78344	0.85550	0.67529
Q9DCV7	35.8	48.53	49.670	Keratin, type II cytoskeletal 7	39	1.73484	1.54454	0.57616	0.89449
Q9DCW4	12.3	12.54	19.610	Electron transfer flavoprotein subunit beta	6	1.02673	0.46697	0.97353	0.46469
Q9DCX2	3.71	3.73	7.453	ATP synthase subunit d, mitochondrial	2	1.06758	0.84914	0.93628	0.79837
Q9DD20	4.84	4.94	11.890	Methyltransferase-like protein 7B	4	2.05367	0.55000	0.48671	0.29313
Q9EP69	3.28	3.9	3.237	Phosphatidylinositide phosphatase SAC1	2	1.45897	1.39620	0.68511	0.95797
Q9EPB4	2.42	2.48	7.254	Apoptosis-associated speck-like protein containing a CARD	1	0.65769	0.65600	1.51979	1.00004
Q9EPK2	5.59	5.7	5.187	Protein XRP2	2	0.79244	1.06924	1.26136	1.35842

Q9EQ06	6.88	7.03	11.740	Estradiol 17-beta-dehydrogenase 11	4	1.24855	0.95321	0.80057	0.76689
Q9EQ20	5.94	6.37	6.355	Methylmalonate-semialdehyde dehydrogenase [acylating], mitochondrial	3	0.94604	0.39453	1.05656	0.41165
Q9EQH2	5.38	6.11	2.366	Endoplasmic reticulum aminopeptidase 1	2	0.86595	0.78888	1.15428	0.91480
Q9EQK5	21.6	25.34	13.360	Major vault protein	11	1.05424	0.83569	0.94812	0.79641
Q9ER00	2.65	2.94	5.474	Syntaxin-12	1	1.24796	0.83614	0.80095	0.67359
Q9ERE2	2.26	15.41	10.810	Keratin, type II cuticular Hb1	9	4.39360	0.60787	0.30942	0.19166
Q9ERG0	19.6	20.33	12.350	LIM domain and actin-binding protein 1	10	0.95441	1.12515	1.04730	1.17735
Q9ERI2	2.06	4.13	9.955	Ras-related protein Rab-27A	3	0.78591	0.71528	1.27183	0.91482
Q9ERL7	1.84	2.08	6.338	Glia maturation factor gamma	1	0.88692	0.99191	1.12700	1.12631
Q9ERS2	6.09	6.13	21.530	NADH dehydrogenase [ubiquinone] 1 alpha subcomplex subunit 13	4	1.32540	0.96450	0.75415	0.72733
Q9ES64	2.05	2.72	1.538	Harmonin	1	0.52977	0.28819	1.88675	0.54542
Q9ES97	5.13	6.01	2.801	Reticulon-3	3	1.26702	1.62387	0.78890	1.27489
Q9ESP1	3.24	3.4	9.502	Stromal cell-derived factor 2-like protein 1	2	1.31713	0.84280	0.84465	0.71389
Q9ET30	6.64	8.26	3.918	Transmembrane 9 superfamily member 3	3	1.41296	1.08248	0.70742	0.75540
Q9JHC0	5.85	5.85	16.840	Glutathione peroxidase 2	3	0.67427	0.97724	1.48242	1.45165
Q9JHJ0	7.22	8.2	8.239	Tropomodulin-3	4	1.13653	1.18987	0.87948	1.05068
Q9JHS3	1.82	1.83	7.200	Ragulator complex protein LAMTOR2	1	1.46567	2.54873	0.68197	1.74351
Q9JI67	6.22	9.12	10.390	Beta-1,3-galactosyltransferase 5	5	0.80200	0.38293	1.24632	0.47613
Q9JIF7	11.9	13.75	6.401	Coatomer subunit beta	5	0.98783	0.82769	1.01187	0.84172
Q9JII6	2.43	2.69	7.077	Alcohol dehydrogenase [NADP(+)]	2	0.71974	0.92036	1.38877	1.28319
Q9JIP7	2.43	2.63	0.987	Solute carrier family 15 member 1	1	0.40368	0.32828	2.47607	0.79606
Q9JIW9	5.48	5.6	13.590	Ras-related protein Ral-B	3	1.09988	1.19937	0.90879	1.09488
Q9JIY5	4.09	4.38	4.803	Serine protease HTRA2, mitochondrial	2	1.19944	0.48747	0.83335	0.40645
Q9JJ00	2.18	2.35	3.049	Phospholipid scramblase 1	1	0.70855	0.84598	1.41070	1.19875

Q9JJ06	2.86	3.4	6.612	Glycoprotein-N-acetylgalactosamine 3-beta-galactosyltransferase 1	2	1.10270	0.84396	0.90646	0.76800
Q9JJI8	3	3.14	27.140	60S ribosomal protein L38	2	1.29622	1.68582	0.77113	1.30320
Q9JJU8	2	2	11.400	SH3 domain-binding glutamic acid-rich-like protein	1	0.64853	0.79232	1.54126	1.22492
Q9JJX6	6.57	7.25	9.278	P2X purinoceptor 4	3	0.56004	0.70920	1.78477	1.26264
Q9JJY3	3.7	4.25	3.053	Sphingomyelin phosphodiesterase 3	2	0.89886	1.07874	1.11202	1.20829
Q9JJZ4	2	2.26	2.201	Ubiquitin-conjugating enzyme E2 J1	1	1.83244	1.25941	0.54548	0.68909
Q9JKA5	14.6	14.91	25.710	Cell surface A33 antigen	12	0.80424	0.66140	1.24285	0.82784
Q9JKF1	7.19	18.97	3.440	Ras GTPase-activating-like protein IQGAP1	6	0.64714	1.28198	1.54458	1.96871
Q9JKR6	19.6	22.76	8.108	Hypoxia up-regulated protein 1	9	0.97792	0.69088	1.02212	0.71191
Q9JL27	3.43	3.72	3.458	Galactoside 2-alpha-L-fucosyltransferase 2	1	1.55013	0.82404	0.64482	0.55890
Q9JL62	2	2.23	4.785	Glycolipid transfer protein	1	0.80769	0.94517	1.23754	1.17328
Q9JLB0	3.34	3.6	3.978	MAGUK p55 subfamily member 6	2	0.83420	0.91059	1.19821	1.09416
Q9JLJ0	2	2	4.348	Lipopolysaccharide-induced tumor necrosis factor-alpha factor homolog	1	0.83601	1.06144	1.19562	1.27298
Q9JLJ2	1.86	2.13	1.417	4-trimethylaminobutyraldehyde dehydrogenase	1	0.71581	0.66394	1.39639	0.92978
Q9JLQ0	4.9	5.38	3.454	CD2-associated protein	2	0.88457	0.78441	1.12999	0.88614
Q9JLZ6	2	2.06	1.616	Hypermethylated in cancer 2 protein	2	1.11152	3.90282	0.89927	3.52046
Q9JM76	6.36	6.8	20.790	Actin-related protein 2/3 complex subunit 3	4	0.90666	1.08129	1.10245	1.20089
Q9JM95	3.46	3.47	9.610	Alpha-N-acetylgalactosaminide alpha-2,6-sialyltransferase 6	3	1.54734	1.11997	0.64598	0.71306
Q9JMA9	5.7	6.45	3.762	Sodium- and chloride-dependent neutral and basic amino acid transporter B(0+)	3	0.37164	0.18670	2.68959	0.50965
Q9JMG2	7.49	7.76	7.595	C1GALT1-specific chaperone 1	3	1.19860	0.64901	0.83393	0.55014
Q9JMH6	2.02	2.43	1.631	Thioredoxin reductase 1, cytoplasmic	1	0.74160	0.95626	1.34783	1.29284
Q9JMH9	4.15	6.99	1.366	Unconventional myosin-XVIIIa	3	1.51577	1.24745	0.65944	0.82735

Q9NYQ2	14.4	15.74	22.100	Hydroxyacid oxidase 2	7	1.21634	0.70512	0.82177	0.63389
Q9QUI0	7.22	7.75	23.830	Transforming protein RhoA	4	0.61006	0.80319	1.63845	1.32570
Q9QUM9	7.01	7.32	17.480	Proteasome subunit alpha type-6	4	0.99326	0.97061	1.00634	0.97810
Q9QWG7	2.43	2.88	5.686	Sulfotransferase family cytosolic 1B member 1	2	0.57255	0.37846	1.74580	0.65486
Q9QXK7	3.29	3.57	2.339	Cleavage and polyadenylation specificity factor subunit 3	2	0.73674	1.51287	1.35673	2.05887
Q9QXS1	118	117.7	9.422	Plectin	48	1.38964	1.12082	0.71929	0.80611
Q9QXT0	1.44	1.44	8.791	Protein canopy homolog 2	1	1.58455	0.70660	0.63081	0.44710
Q9QXX4	5.14	6.41	5.473	Calcium-binding mitochondrial carrier protein Aralar2	3	1.25190	1.04511	0.79843	0.84187
Q9QY23	2.26	2.39	1.882	Plakophilin-3	2	1.03189	1.70220	0.96866	1.65614
Q9QY76	8.17	8.23	18.930	Vesicle-associated membrane protein-associated protein B	5	1.31861	1.14839	0.75803	0.86310
Q9QYA2	2.38	2.76	3.047	Mitochondrial import receptor subunit TOM40 homolog	1	1.25325	1.33995	0.79757	1.07199
Q9QYE6	3.07	4.33	1.783	Golgin subfamily A member 5	1	1.76163	0.98339	0.56740	0.56110
Q9QYJ0	8.72	9.11	10.920	DnaJ homolog subfamily A member 2	5	1.03350	1.02488	0.96715	0.99383
Q9QZ85	1.66	2.4	2.179	Interferon-inducible GTPase 1	1	0.82340	1.99474	1.21393	2.42891
Q9QZ88	2.13	2.24	4.945	Vacuolar protein sorting-associated protein 29	1	0.76341	1.24282	1.30933	1.63255
Q9QZD9	2	2.2	3.077	Eukaryotic translation initiation factor 3 subunit I	1	1.31571	0.77506	0.75971	0.59063
Q9QZE5	2.09	2.92	1.030	Coatomer subunit gamma-1	1	1.23056	0.83731	0.81227	0.68308
Q9QZQ1	2.16	3.1	0.714	Afadin	1	0.97125	1.10064	1.02914	1.14560
Q9QZS3	1.37	1.58	1.685	Protein numb homolog	1	0.98970	1.18814	1.00996	1.20366
Q9R092	2.04	2.28	2.839	17-beta-hydroxysteroid dehydrogenase type 6	1	1.48134	3.08088	0.67476	2.08525
Q9R0A0	2.03	2.26	3.191	Peroxisomal membrane protein PEX14	1	1.47403	1.27058	0.67811	0.86181
Q9R0A1	2.08	2.44	0.881	Chloride channel protein 2	1	1.28117	0.58548	0.78019	0.46063
Q9R0H0	9.59	9.96	7.262	Peroxisomal acyl-coenzyme A oxidase 1	5	1.91449	0.85405	0.52210	0.45739
Q9R0K7	7.81	8.53	5.426	Plasma membrane calcium-transporting ATPase 2	5	0.72343	0.57614	1.38168	0.80104
Q9R0M6	4.03	4.03	9.950	Ras-related protein Rab-9A	2	0.84992	0.78297	1.17605	0.92339

Q9R0P5	17.8	20.15	53.330	Destrin	11	0.62139	0.61616	1.60857	1.00259
Q9R0P6	1.73	1.77	5.028	Signal peptidase complex catalytic subunit SEC11A	1	1.29451	1.36021	0.77215	1.04441
Q9R0Q3	1.32	1.32	8.955	Transmembrane emp24 domain-containing protein 2	2	1.35382	1.00949	0.73832	0.74950
Q9R0Q7	2	2.04	5.000	Prostaglandin E synthase 3	1	0.99819	1.63561	1.00136	1.64287
Q9R0Q9	2.64	4.13	6.478	Mannose-P-dolichol utilization defect 1 protein	2	1.58347	1.21243	0.82338	1.00136
Q9R100	25.5	26.03	16.080	Cadherin-17	13	0.87645	0.57158	1.14045	0.65423
Q9R112	15.2	15.91	16.440	Sulfide:quinone oxidoreductase, mitochondrial	8	1.84945	0.80613	0.54046	0.43862
Q9R1J0	2.74	3.41	3.591	Sterol-4-alpha-carboxylate 3-dehydrogenase, decarboxylating	1	3.76787	1.45682	0.26528	0.38766
Q9R1P1	2.03	2.13	4.390	Proteasome subunit beta type-3	1	0.92359	1.12174	1.08224	1.21939
Q9R1P3	5.16	5.29	11.940	Proteasome subunit beta type-2	3	0.93264	0.99436	1.07175	1.06706
Q9R1P4	4.5	4.8	6.464	Proteasome subunit alpha type-1	2	0.86835	1.26896	1.15110	1.47921
Q9R1Q7	2.16	2.16	7.895	Proteolipid protein 2	1	1.01494	0.96900	0.98484	0.95724
Q9R233	4.84	5.72	4.516	Tapasin	2	1.90967	1.84970	0.52342	0.98039
Q9WTL2	4.02	4.2	9.859	Ras-related protein Rab-25	2	1.33357	0.88942	0.74953	0.66870
Q9WTL7	2.68	4.04	9.091	Acyl-protein thioesterase 2	3	0.88314	1.03293	1.13181	1.17228
Q9WTM5	2.68	2.74	2.592	RuvB-like 2	1	0.97807	0.96279	1.02197	0.98715
Q9WTP6	4.7	6.35	17.990	Adenylate kinase 2, mitochondrial	4	1.03337	0.84667	0.96727	0.82602
Q9WTP7	19.3	19.6	48.900	GTP:AMP phosphotransferase AK3, mitochondrial	11	0.74633	0.43726	1.33930	0.58607
Q9WTS2	2.07	2.36	1.391	Alpha-(1,6)-fucosyltransferase	1	1.53497	1.08355	0.65119	0.70776
Q9WTX5	2.01	2.32	4.908	S-phase kinase-associated protein 1	1	0.78478	1.24967	1.27367	1.59655
Q9WU01	1.6	2.31	1.719	KH domain-containing, RNA-binding, signal transduction-associated protein 2	1	0.92020	1.26061	1.08623	1.37671
Q9WU78	35.1	40.52	17.840	Programmed cell death 6-interacting protein	17	0.95188	1.13080	1.05008	1.18322
Q9WUA3	1.64	3.21	2.679	6-phosphofructokinase type C	3	0.95914	1.03892	1.04213	1.08602
Q9WUD1	3.24	3.31	6.250	STIP1 homology and U box-containing protein 1	2	0.96138	1.17085	1.03971	1.21855

Q9WUK2	1.74	2.12	8.065	Eukaryotic translation initiation factor 4H	2	0.76506	0.79150	1.30651	1.03427
Q9WUM3	2.57	2.75	1.653	Coronin-1B	1	0.81850	1.06618	1.22119	1.30149
Q9WUM4	3.01	6.17	3.165	Coronin-1C	3	0.72750	1.57990	1.37395	2.18879
Q9WUM5	2.62	3.99	4.046	Succinyl-CoA ligase [ADP/GDP-forming] subunit alpha, mitochondrial	2	1.24746	0.70515	0.80127	0.56682
Q9WUR2	2	2.22	2.302	Enoyl-CoA delta isomerase 2, mitochondrial	1	1.30847	0.92644	0.76391	0.70990
Q9WUZ9	3.81	5.24	3.747	Ectonucleoside triphosphate diphosphohydrolase 5	2	1.52031	0.35267	0.65746	0.23597
Q9WV32	4.72	5.28	6.989	Actin-related protein 2/3 complex subunit 1B	2	1.01524	1.45284	0.98454	1.44631
Q9WV54	10.7	10.93	14.970	Acid ceramidase	6	1.07714	1.31185	0.92797	1.21939
Q9WV55	3.64	5.82	10.840	Vesicle-associated membrane protein-associated protein A	4	1.39234	1.08068	0.71789	0.77732
Q9WV80	6.28	8.03	8.812	Sorting nexin-1	4	1.08396	1.25531	0.92213	1.16386
Q9WV91	4.29	4.98	1.934	Prostaglandin F2 receptor negative regulator	2	0.93731	1.31400	1.06641	1.40624
Q9WV92	5.07	5.66	2.799	Band 4.1-like protein 3	3	0.75543	0.55480	1.32315	0.76303
Q9WVA4	15.4	15.49	47.740	Transgelin-2	9	0.63707	1.33561	1.56899	2.10672
Q9WVC8	14.7	15.03	10.570	Chloride anion exchanger	7	0.35554	0.24658	2.81136	0.67938
Q9WVE8	3.34	3.53	4.321	Protein kinase C and casein kinase substrate in neurons protein 2	2	0.77922	0.79170	1.28276	1.01995
Q9WVJ2	8.79	9.62	9.309	26S proteasome non-ATPase regulatory subunit 13	4	0.99184	0.96062	1.00778	0.97102
Q9WVL3	2.12	2.32	0.739	Solute carrier family 12 member 7	1	0.82544	0.78606	1.21093	0.95343
Q9Z0F4	3.07	3.11	8.901	Calcium and integrin-binding protein 1	2	0.79405	0.63035	1.25880	0.78100
Q9Z0F8	2.23	2.5	0.967	Disintegrin and metalloproteinase domain-containing protein 17	1	1.11753	1.73969	0.89443	1.56097
Q9Z0G9	2.27	2.27	5.479	Claudin-3	2	0.89328	0.35650	1.11897	0.41273
Q9Z0S9	2.04	2.21	8.108	Prenylated Rab acceptor protein 1	1	1.16804	0.76827	0.85575	0.65874
Q9Z0T9	4	5.53	3.177	Integrin beta-6	2	1.22295	1.36343	0.81733	1.11888

Q9Z0U1	14	15.2	4.970	Tight junction protein ZO-2	6	1.07626	1.41159	0.92873	1.31563
Q9Z0X1	6.43	6.82	4.575	Apoptosis-inducing factor 1, mitochondrial	3	1.16836	1.10019	0.85551	0.94883
Q9Z0Y1	2.87	3.94	10.220	Dynactin subunit 3	2	0.82181	0.82154	1.21629	1.00373
Q9Z199	2.08	2.08	6.838	Transcription elongation factor SPT4-B	1	1.87298	2.41018	0.53367	1.27507
Q9Z1G4	1.55	1.81	1.073	V-type proton ATPase 116 kDa subunit a isoform 1	1	1.11547	1.12461	0.89609	1.01989
Q9Z1N5	7.67	10.15	9.579	Spliceosome RNA helicase Ddx39b	5	1.38439	1.29796	0.72202	0.94008
Q9Z1P6	3.86	5.97	24.780	NADH dehydrogenase [ubiquinone] 1 alpha subcomplex subunit 7	3	1.32981	0.99448	0.75165	0.75451
Q9Z1Q5	6.51	7.03	16.180	Chloride intracellular channel protein 1	4	0.78902	1.18628	1.26683	1.50922
Q9Z1W8	19.8	35.97	14.590	Potassium-transporting ATPase alpha chain 2	19	0.31922	0.39276	3.13122	1.24094
Q9Z1Z0	2.23	3.41	0.938	General vesicular transport factor p115	1	1.00263	0.87957	0.99693	0.87202
Q9Z204	6.24	6.44	10.540	Heterogeneous nuclear ribonucleoproteins C1/C2	3	1.21389	1.12817	0.82343	0.92941
Q9Z261	2	2	7.583	Claudin-7	2	1.01079	0.60013	0.98888	0.59528
Q9Z280	1.99	6.56	1.862	Phospholipase D1	3	0.76106	0.43236	1.31336	0.57559
Q9Z2G9	2.03	3.62	7.025	Oxidoreductase HTATIP2	2	1.76283	1.09026	0.56702	0.62788
Q9Z2I0	22.5	25.05	16.940	LETM1 and EF-hand domain-containing protein 1, mitochondrial	13	1.06905	0.69239	0.93499	0.64771
Q9Z2I8	20	20.45	23.790	Succinyl-CoA ligase [GDP-forming] subunit beta, mitochondrial	11	1.01895	0.45029	0.98096	0.43797
Q9Z2I9	15.8	16.25	16.850	Succinyl-CoA ligase [ADP-forming] subunit beta, mitochondrial	9	0.99424	0.53823	1.00534	0.54442
Q9Z2M7	13	13.45	28.100	Phosphomannomutase 2	7	0.72041	0.62366	1.38749	0.86501
Q9Z2Q6	7.15	7.24	9.756	Septin-5	4	1.08527	1.64505	0.92102	1.52335
Q9Z2T6	13	17.99	13.410	Keratin, type II cuticular Hb5	11	2.43613	0.44135	0.41030	0.18164
Q9Z2U0	5.04	7.08	20.970	Proteasome subunit alpha type-7	5	0.83289	0.93092	1.20010	1.11915
Q9Z2U1	2.58	4.83	6.224	Proteasome subunit alpha type-5	3	0.64305	0.74130	1.55439	1.15437

Q9Z2W0	1.36	3.48	2.114	Aspartyl aminopeptidase	2	0.95057	1.33641	1.05152	1.40959
Q9Z2X1	5.04	6.42	6.747	Heterogeneous nuclear ribonucleoprotein F	3	0.66162	0.71570	1.51076	1.08458
Q9Z2Z6	1.66	1.89	0.000	Mitochondrial carnitine/acylcarnitine carrier protein	0	1.18718	0.82320	0.84196	0.70479
RRRRRP0C 6F1	2.15	10.53	0.359	REVERSED Dynein heavy chain 2, axonemal	3	1.35126	1.70303	0.73972	1.26363
RRRRRQ80 X19	2.03	2.37	0.334	REVERSED Collagen alpha-1(XIV) chain	1	1.55932	2.42574	0.64102	1.55972
RRRRRQ91 ZU9	1.51	2.7	0.499	REVERSED Glutamate receptor ionotropic, NMDA 3B	1	0.63078	1.50919	1.58464	2.39887

Table S5. The proteins identified and quantified in the experiment replicate 2

Accession	Unused	Total	%Cov(95)	Name	Peptides(95%)	116:114	117:114	114:116	117:116
A2A863	21	21.88	6.711	Integrin beta-4	11	1.17706	1.61278	0.84907	1.37026
A7L9Z8	2.07	3.2	1.271	Calcium-transporting ATPase type 2C member 2	1	0.81855	0.76020	1.22095	0.93379
B2RX12	17.2	17.86	3.677	Canalicular multispecific organic anion transporter 2	6	0.95164	0.60382	1.05020	0.64213
B2RXS4	37.8	39.18	9.935	Plexin-B2	20	0.90114	0.82495	1.10904	0.91145
D3Z6P0	2.72	3.79	2.087	Protein disulfide-isomerase A2	1	1.57303	0.80481	0.63534	0.51633
E9Q557	12.8	22.44	1.769	Desmoplakin	6	1.25003	1.24822	0.79951	0.99499
F7BWT7	2.25	2.34	6.803	Tetraspanin-15	2	0.90691	0.98352	1.10199	1.09176
O08528	9.32	10.28	4.689	Hexokinase-2	5	1.08960	1.06221	0.91722	0.98098
O08553	3.16	4.16	4.545	Dihydropyrimidinase-related protein 2	2	0.91180	1.15988	1.09608	1.27943
O08585	2.71	3.05	2.979	Clathrin light chain A	1	0.96280	1.08316	1.03803	1.12520
O08638	19.3	36.76	6.592	Myosin-11	15	1.24626	1.57912	0.80193	1.22570
O08663	2.73	2.94	1.883	Methionine aminopeptidase 2	1	1.12528	1.18502	0.88814	1.05789
O08709	15.2	17.99	27.230	Peroxiredoxin-6	8	0.64783	0.49585	1.54271	0.77520

O08749	6.29	6.6	10.020	Dihydrolipoyl dehydrogenase, mitochondrial	6	0.71355	0.43869	1.40061	0.62329
O08756	6.23	6.23	11.490	3-hydroxyacyl-CoA dehydrogenase type-2	4	0.85590	0.46588	1.16768	0.54598
O08788	3.23	6.82	1.717	Dynactin subunit 1	2	0.75108	0.55409	1.33063	0.74468
O08795	4.12	4.22	4.415	Glucosidase 2 subunit beta	2	1.83898	1.06691	0.54346	0.57711
O08832	2.03	2.19	1.903	Polypeptide N-acetylgalactosaminyltransferase 4	1	1.52183	0.56251	0.65672	0.37165
O08917	6.15	6.57	8.645	Flotillin-1	3	1.33913	2.69506	0.74631	2.06553
O08997	2	2.02	11.760	Copper transport protein ATOX1	1	1.07564	1.30811	0.92913	1.22278
O09061	2.27	2.27	3.750	Proteasome subunit beta type-1	1	0.91700	0.86421	1.08987	0.94759
O09111	2	2	6.623	NADH dehydrogenase [ubiquinone] 1 beta subcomplex subunit 11, mitochondrial	1	1.49371	1.41180	0.66908	0.95033
O09117	3.96	4.33	5.747	Synaptophysin-like protein 1	3	1.27209	1.37849	0.78564	1.08484
O09131	2.62	3.08	7.500	Glutathione S-transferase omega-1	2	0.71875	0.59986	1.39048	0.83779
O09159	1.39	2.51	0.790	Lysosomal alpha-mannosidase	1	1.08554	1.02838	0.92066	0.95253
O35054	1.89	1.89	5.714	Claudin-4	2	1.08270	1.20010	0.92307	1.11450
O35114	4.47	4.79	4.184	Lysosome membrane protein 2	2	0.95358	1.38369	1.04806	1.45765
O35129	13.2	13.35	21.400	Prohibitin-2	8	1.31882	1.38270	0.75781	1.05209
O35143	2.05	2.06	7.547	ATPase inhibitor, mitochondrial	1	1.46236	0.52554	0.68342	0.36134
O35379	1.4	6.19	1.243	Multidrug resistance-associated protein 1	3	1.06025	1.20467	0.94262	1.14242
O35381	2.02	2.14	3.239	Acidic leucine-rich nuclear phosphoprotein 32 family member A	1	0.72636	1.45676	1.37591	1.99886
O35459	2.44	2.81	4.281	Delta(3,5)-Delta(2,4)-dienoyl-CoA isomerase, mitochondrial	1	1.14951	0.60118	0.86942	0.52427
O35566	1.75	2.16	1.976	CD151 antigen	1	1.06023	1.23421	0.94263	1.17326
O35598	4.13	4.56	2.937	Disintegrin and metalloproteinase domain-containing protein 10	2	1.14294	1.63400	0.87442	1.43691
O35609	2.07	2.11	4.585	Secretory carrier-associated membrane protein 3	1	0.73592	1.09645	1.35803	1.50029

O35639	14.9	17.96	31.890	Annexin A3	10	1.06652	1.36840	0.93707	1.28844
O35640	6.25	6.64	15.600	Annexin A8	6	1.30455	2.51489	0.76609	1.99616
O35643	21.2	24	12.200	AP-1 complex subunit beta-1	12	0.83161	0.99726	1.20178	1.20670
O35744	3.24	4.16	4.774	Chitinase-3-like protein 3	3	0.50766	1.36686	1.96868	2.70721
O35857	2.02	2.4	1.770	Mitochondrial import inner membrane translocase subunit TIM44	1	0.76370	0.68977	1.30863	0.90813
O35874	1.48	2.25	2.068	Neutral amino acid transporter A	1	1.21799	0.79298	0.82054	0.65462
O35887	2.87	3.32	5.714	Calumenin	3	1.19974	1.13554	0.83302	0.94664
O35900	1.62	1.93	7.368	U6 snRNA-associated Sm-like protein LSm2	1	0.65139	0.70196	1.53426	1.08352
O54734	9.31	9.47	11.110	Dolichyl-diphosphooligosaccharide--protein glycosyltransferase 48 kDa subunit	6	1.27190	0.90948	0.78576	0.72246
O55013	1.77	1.79	4.444	Trafficking protein particle complex subunit 3	1	1.09998	1.18389	0.90857	1.08217
O55022	6.94	7.01	11.790	Membrane-associated progesterone receptor component 1	3	1.26078	0.73714	0.79269	0.58903
O55023	1.92	2.19	3.971	Inositol monophosphatase 1	1	0.80165	0.72040	1.24669	0.90355
O55111	10.7	11.79	4.545	Desmoglein-2	5	1.17498	1.11472	0.85058	0.95097
O55125	3.92	4.17	4.225	Protein NipSnap homolog 1	2	1.36145	1.00275	0.73408	0.73780
O55131	2.81	3.23	5.275	Septin-7	3	1.12724	1.75244	0.88660	1.55912
O55143	8.17	15.65	6.513	Sarcoplasmic/endoplasmic reticulum calcium ATPase 2	7	1.05219	1.33227	0.94984	1.26932
O55222	2.02	2.48	2.212	Integrin-linked protein kinase	1	2.62731	5.84683	0.38039	2.23757
O70251	11.1	11.29	22.670	Elongation factor 1-beta	9	1.07607	0.87293	0.92876	0.81270
O70274	2.35	2.82	4.790	Protein tyrosine phosphatase type IVA 2	1	0.89727	0.90222	1.11383	1.01466
O70400	12	12.72	14.070	PDZ and LIM domain protein 1	5	0.69723	0.85232	1.43339	1.23088
O70435	6.15	6.35	10.980	Proteasome subunit alpha type-3	3	1.25586	1.17852	0.79580	0.94026
O70439	6.84	6.85	14.940	Syntaxin-7	3	1.17165	1.61806	0.85299	1.37311
O70456	2.33	8.89	14.920	14-3-3 protein sigma	7	0.89537	0.78776	1.11619	0.88743

O70475	18.9	19.48	22.520	UDP-glucose 6-dehydrogenase	12	0.82630	0.48713	1.20950	0.59823
O70492	3.84	4.27	14.200	Sorting nexin-3	2	1.29519	1.51027	0.77163	1.19959
O70493	2.1	2.14	5.455	Sorting nexin-12	1	0.97487	1.42049	1.02517	1.46506
O70570	10.8	11.05	8.301	Polymeric immunoglobulin receptor	6	1.12351	0.89160	0.88954	0.79767
O70589	2.03	3.34	1.188	Peripheral plasma membrane protein CASK	1	0.84230	1.63404	1.18653	1.95152
O88312	15.6	15.66	32.570	Anterior gradient protein 2 homolog	18	1.10051	0.16473	0.90813	0.14881
O88329	15.4	17.25	8.725	Unconventional myosin-Ia	9	0.59342	0.55736	1.68415	0.94317
O88342	5.35	5.75	4.785	WD repeat-containing protein 1	3	0.65664	1.07540	1.52200	1.64940
O88343	2.08	2.69	0.649	Electrogenic sodium bicarbonate cotransporter 1	1	0.86305	0.54147	1.15799	0.63082
O88456	2.14	2.19	7.063	Calpain small subunit 1	2	1.03189	1.20116	0.96852	1.17192
O88487	2	2.2	1.144	Cytoplasmic dynein 1 intermediate chain 2	1	0.78127	0.84844	1.27920	1.09190
O88569	6.87	7.56	13.310	Heterogeneous nuclear ribonucleoproteins A2/B1	5	0.43109	0.27145	2.31835	0.63544
O88685	4.07	4.52	3.846	26S protease regulatory subunit 6A	2	0.99858	0.73464	1.00083	0.75602
O88844	13.5	13.64	16.430	Isocitrate dehydrogenase [NADP] cytoplasmic	7	0.99382	1.29582	1.00563	1.31645
O88952	7.72	9.1	20.300	Protein lin-7 homolog C	5	0.99324	1.26934	1.00621	1.29516
O88983	1.95	2.83	3.390	Syntaxin-8	1	0.85612	1.41406	1.16738	1.66074
O89001	13	14.22	4.575	Carboxypeptidase D	7	0.85356	0.66177	1.17088	0.77929
O89053	2.53	2.74	2.169	Coronin-1A	1	1.19241	2.31821	0.83814	1.95888
O89079	6.18	8.3	16.230	Coatomer subunit epsilon	4	0.93738	0.76457	1.06618	0.82126
P00329	5	5.36	7.467	Alcohol dehydrogenase 1	4	0.73652	0.64035	1.35693	0.87637
P00405	4.09	6.51	10.570	Cytochrome c oxidase subunit 2	3	1.04473	0.62340	0.95662	0.59833
P00920	2.33	3.23	3.462	Carbonic anhydrase 2	1	0.36765	0.23378	2.71834	0.63936
P01592	2.95	2.95	16.350	Immunoglobulin J chain	2	1.49578	1.20798	0.66815	0.81135
P01831	2.78	2.78	8.642	Thy-1 membrane glycoprotein	1	1.22688	3.46184	0.81459	2.91677
P01837	5.35	5.45	25.470	Ig kappa chain C region	3	1.38276	1.20151	0.72276	0.87465
P01878	2.23	2.47	4.360	Ig alpha chain C region	1	1.23213	1.09100	0.81113	0.88721

P01899	12.9	12.95	22.100	H-2 class I histocompatibility antigen, D-B alpha chain	9	0.79176	0.90099	1.26227	1.14311
P01901	5.65	13.73	15.450	H-2 class I histocompatibility antigen, K-B alpha chain	9	0.94378	1.01490	1.05895	1.08053
P01902	2.04	6.67	9.511	H-2 class I histocompatibility antigen, K-D alpha chain	4	0.70877	0.24630	1.41006	0.34940
P01942	10.9	11.14	33.800	Hemoglobin subunit alpha	9	0.70530	0.48682	1.41699	0.71270
P02088	16.1	16.07	59.180	Hemoglobin subunit beta-1	26	0.75933	0.40968	1.31618	0.54438
P02301	2.32	2.76	10.290	Histone H3.3C	2	0.89199	1.85991	1.12042	2.09779
P02535	4.67	8.64	8.070	Keratin, type I cytoskeletal 10	6	0.39227	0.70734	2.54773	1.81801
P03930	3.07	3.08	28.360	ATP synthase protein 8	3	1.11844	1.13677	0.89357	1.02228
P04441	2.9	2.9	8.602	H-2 class II histocompatibility antigen gamma chain	2	1.23004	1.43686	0.81250	1.17192
P04919	2	5.19	1.615	Band 3 anion transport protein	3	1.04700	1.75897	0.95454	1.68919
P05064	25	25.57	37.090	Fructose-bisphosphate aldolase A	18	0.91517	0.91999	1.09205	1.01862
P05202	12	12.12	16.980	Aspartate aminotransferase, mitochondrial	6	1.28309	0.92312	0.77891	0.72546
P05784	27.8	35.15	36.410	Keratin, type I cytoskeletal 18	36	1.38527	0.89747	0.72145	0.65451
P06151	15.7	17.68	23.490	L-lactate dehydrogenase A chain	15	0.70289	0.75649	1.42186	1.05696
P06745	2.39	4.51	4.122	Glucose-6-phosphate isomerase	3	0.61650	1.22790	1.62110	2.00323
P06800	3.07	3.87	0.852	Receptor-type tyrosine-protein phosphatase C	1	1.12152	3.62129	0.89112	3.26302
P07309	3.26	3.5	10.880	Transthyretin	2	0.92657	1.20249	1.07862	1.30400
P07356	43.9	45.23	49.850	Annexin A2	43	1.11794	2.38561	0.89397	2.11668
P07724	27.1	27.84	18.590	Serum albumin	14	1.07418	1.27291	0.93040	1.16964
P07758	7.13	8.52	8.717	Alpha-1-antitrypsin 1-1	5	1.20041	1.13987	0.83256	0.94640
P07901	3.08	16.13	10.910	Heat shock protein HSP 90-alpha	9	1.12897	1.32264	0.88524	1.18348
P08003	20.9	21.79	18.030	Protein disulfide-isomerase A4	13	1.27102	1.08952	0.78630	0.86450
P08030	2.08	2.08	5.556	Adenine phosphoribosyltransferase	1	1.18653	1.82808	0.84229	1.54911
P08071	5.01	5.33	4.526	Lactotransferrin	3	1.55878	3.11771	0.64115	2.22221
P08113	35.3	38.45	22.320	Endoplasmin	20	1.55536	1.11126	0.64256	0.71568
P08207	12.8	12.84	34.020	Protein S100-A10	13	0.94669	1.35448	1.05569	1.43344

P08228	4.03	4.22	14.940	Superoxide dismutase [Cu-Zn]	2	0.63977	0.54137	1.56215	0.81286
P08249	30	30.73	54.730	Malate dehydrogenase, mitochondrial	24	1.05667	0.44212	0.94581	0.42407
P08752	14	15.85	23.380	Guanine nucleotide-binding protein G(i) subunit alpha-2	10	0.87284	0.99301	1.14501	1.14503
P09055	25.7	27.27	15.410	Integrin beta-1	14	1.00854	1.33389	0.99095	1.32384
P09103	37	37.47	34.180	Protein disulfide-isomerase	23	1.25712	0.87328	0.79500	0.70334
P09405	14.5	15.3	11.880	Nucleolin	9	1.26550	1.26865	0.78974	1.02344
P09411	12.7	15.4	13.190	Phosphoglycerate kinase 1	7	0.82319	0.90226	1.21406	1.10544
P09671	5.98	6.26	13.510	Superoxide dismutase [Mn], mitochondrial	4	0.89367	0.65210	1.11832	0.73576
P09803	9.56	10.88	6.561	Cadherin-1	5	0.90696	1.23590	1.10194	1.37126
P10107	32.6	34.96	48.840	Annexin A1	23	1.52068	5.26510	0.65721	3.88090
P10126	21.4	23.77	27.060	Elongation factor 1-alpha 1	15	1.13200	1.66894	0.88287	1.47153
P10404	6	6.78	5.304	MLV-related proviral Env polyprotein	3	1.04411	2.78348	0.95719	2.68276
P10637	1.41	2.07	1.091	Microtubule-associated protein tau	1	1.05591	0.80072	0.94649	0.75982
P10639	6.15	6.59	29.520	Thioredoxin	7	0.73538	0.62791	1.35905	0.85833
P10649	9.94	10.06	22.480	Glutathione S-transferase Mu 1	5	0.90320	0.63441	1.10652	0.70738
P10852	4.8	5.02	3.802	4F2 cell-surface antigen heavy chain	2	1.07878	1.50423	0.92643	1.39437
P11031	2	2.04	8.661	Activated RNA polymerase II transcriptional coactivator p15	1	1.31383	1.63466	0.76068	1.25100
P11352	4.44	4.62	13.430	Glutathione peroxidase 1	3	0.84897	0.98686	1.17719	1.16475
P11438	4.36	4.36	5.665	Lysosome-associated membrane glycoprotein 1	4	0.78099	1.17425	1.27966	1.49068
P11499	27.6	32.07	18.920	Heat shock protein HSP 90-beta	19	1.15701	1.62731	0.86379	1.42075
P11672	1.82	2.3	3.500	Neutrophil gelatinase-associated lipocalin	1	1.40237	3.06889	0.71266	2.27098
P11679	74.3	74.36	64.900	Keratin, type II cytoskeletal 8	111	1.79716	0.43542	0.55610	0.24587
P11688	2.61	2.94	1.330	Integrin alpha-5	1	1.24701	1.81347	0.80145	1.46291
P11835	4.27	4.92	2.983	Integrin beta-2	2	1.22036	1.88157	0.81895	1.74285
P11983	14.8	17.23	16.910	T-complex protein 1 subunit alpha	8	0.96836	1.23432	1.03206	1.28168

P12367	4.11	4.38	4.239	cAMP-dependent protein kinase type II-alpha regulatory subunit	2	0.90855	0.72953	1.10001	0.80731
P12787	14.7	15.02	53.420	Cytochrome c oxidase subunit 5A, mitochondrial	11	1.36907	0.83847	0.72999	0.61331
P12815	3.32	3.56	10.990	Programmed cell death protein 6	3	1.06557	1.47205	0.93791	1.38778
P13020	4.84	7.83	7.051	Gelsolin	5	0.74398	0.93634	1.34332	1.26910
P13634	15.6	15.91	33.330	Carbonic anhydrase 1	16	0.61747	0.20368	1.61856	0.32910
P13808	11.6	13.47	3.880	Anion exchange protein 2	5	0.90208	0.57121	1.10790	0.63721
P14069	5.55	5.61	16.850	Protein S100-A6	3	0.91867	1.14074	1.08789	1.21760
P14094	13.3	14.6	24.340	Sodium/potassium-transporting ATPase subunit beta-1	21	0.66896	0.41401	1.49397	0.61914
P14115	2	2.25	7.432	60S ribosomal protein L27a	1	1.08978	1.93391	0.91708	1.78429
P14131	4.21	4.63	12.330	40S ribosomal protein S16	2	0.68265	1.46075	1.46402	2.17281
P14152	12.1	12.56	17.070	Malate dehydrogenase, cytoplasmic	6	0.68621	0.72849	1.45643	1.06492
P14206	11.5	11.63	22.030	40S ribosomal protein SA	7	1.00274	1.03520	0.99668	1.03755
P14211	16.7	17.23	18.750	Calreticulin	12	1.57100	1.29738	0.63616	0.82964
P14434	4	4.05	8.984	H-2 class II histocompatibility antigen, A-B alpha chain	2	0.65748	1.66458	1.52006	2.54456
P14733	3.03	5.66	3.231	Lamin-B1	2	1.08557	1.40173	0.92063	1.29035
P14824	42.1	43.68	24.670	Annexin A6	22	1.06568	3.36311	0.93782	3.31812
P14869	22.7	22.92	39.430	60S acidic ribosomal protein P0	14	1.29751	1.21141	0.80838	0.98466
P15105	1.56	1.57	2.145	Glutamine synthetase	1	1.06577	1.61491	0.93773	1.52353
P15208	2.44	4.63	0.656	Insulin receptor	1	0.82416	0.81446	1.21265	0.99029
P15532	4.04	7.52	19.080	Nucleoside diphosphate kinase A	4	0.95612	1.30906	1.04527	1.37614
P15535	2	2.08	2.506	Beta-1,4-galactosyltransferase 1	1	1.29377	1.34247	0.77248	1.04331
P16045	3.85	3.95	11.850	Galectin-1	2	1.02248	4.22228	0.97744	4.18016
P16110	16.2	16.25	32.580	Galectin-3	21	0.71350	0.73365	1.40071	0.95347
P16546	74.9	76.04	16.140	Spectrin alpha chain, non-erythrocytic 1	44	1.11712	1.19399	0.89463	1.07097
P16858	16.9	18.65	24.620	Glyceraldehyde-3-phosphate dehydrogenase	11	0.92042	1.46957	1.08582	1.59455

P17047	4.82	4.87	4.096	Lysosome-associated membrane glycoprotein 2	2	1.45646	5.52090	0.68619	5.04976
P17182	22.2	23.9	27.190	Alpha-enolase	15	0.91467	0.97079	1.09264	1.07251
P17427	5.94	6.93	2.985	AP-2 complex subunit alpha-2	3	1.05601	1.81104	0.94640	1.72288
P17563	21.5	21.62	22.460	Selenium-binding protein 1	13	0.61287	0.48483	1.63070	0.72397
P17665	4	4	28.570	Cytochrome c oxidase subunit 7C, mitochondrial	3	1.49225	0.77771	0.66973	0.51223
P17742	13.8	16.95	45.120	Peptidyl-prolyl cis-trans isomerase A	10	0.77608	1.63053	1.28777	2.06948
P17751	13	13.49	26.760	Triosephosphate isomerase	7	0.81001	0.81335	1.23382	1.00988
P18242	7.71	8.25	8.780	Cathepsin D	4	1.03569	1.59571	0.96497	1.56362
P18572	12.4	13.17	16.970	Basigin	9	0.81023	0.45439	1.23349	0.56133
P18760	14.1	16.73	45.180	Cofilin-1	11	0.86746	1.51654	1.15211	1.77028
P19001	60.6	60.61	70.720	Keratin, type I cytoskeletal 19	71	1.73197	0.49812	0.57704	0.30055
P19096	4.88	9.52	1.198	Fatty acid synthase	4	0.84916	1.30527	1.17694	1.54059
P19157	5.8	6.34	21.430	Glutathione S-transferase P 1	4	0.74915	0.74451	1.33405	0.99637
P19253	2.66	2.76	3.448	60S ribosomal protein L13a	1	0.97779	1.29518	1.02211	1.33862
P19324	4.8	4.94	5.516	Serpin H1	2	1.49558	3.41659	0.66824	2.73105
P19467	9.81	10.06	12.910	Mucin-13	10	0.66067	0.86498	1.51272	1.31103
P19536	12.7	12.93	44.530	Cytochrome c oxidase subunit 5B, mitochondrial	14	1.31452	0.75316	0.76029	0.57693
P19639	1.52	3.69	9.633	Glutathione S-transferase Mu 3	2	0.70138	0.44818	1.42491	0.64249
P19783	9.42	9.57	28.400	Cytochrome c oxidase subunit 4 isoform 1, mitochondrial	8	1.25321	0.75219	0.79748	0.60385
P20029	42.3	44.66	34.660	78 kDa glucose-regulated protein	38	1.41118	0.93150	0.70821	0.66090
P20060	4.11	4.17	3.545	Beta-hexosaminidase subunit beta	2	1.67200	1.43723	0.59773	0.86434
P20065	3.17	3.17	26.000	Thymosin beta-4	2	0.84843	1.46838	1.17796	1.75027
P20108	5.07	5.53	11.670	Thioredoxin-dependent peroxide reductase, mitochondrial	3	0.53081	0.55599	1.88282	1.05316
P20152	6.05	19.17	12.660	Vimentin	14	1.94611	1.90970	0.51354	1.11754

P20352	1.75	1.75	3.061	Tissue factor	1	0.98089	2.67036	1.01888	2.73727
P21107	10.6	11.19	15.090	Tropomyosin alpha-3 chain	7	0.87519	1.18741	1.14193	1.36929
P21278	16.5	17.04	23.120	Guanine nucleotide-binding protein subunit alpha-11	11	0.53044	0.44173	1.88412	0.83766
P21279	3.93	16.18	22.840	Guanine nucleotide-binding protein G(q) subunit alpha	10	0.73838	1.05971	1.35352	1.45173
P21447	46.1	46.52	18.890	Multidrug resistance protein 1A	25	0.58018	0.42712	1.72258	0.72455
P22892	4.47	4.69	2.068	AP-1 complex subunit gamma-1	2	0.83190	1.02127	1.20136	1.23699
P23116	1.34	1.69	0.819	Eukaryotic translation initiation factor 3 subunit A	1	0.59680	0.95007	1.67462	1.60065
P23506	2	2.3	4.846	Protein-L-isoaspartate(D-aspartate) O-methyltransferase	1	1.17545	1.39505	0.85024	1.19331
P23591	1.8	2.54	3.738	GDP-L-fucose synthase	1	0.70517	0.76331	1.41727	1.08837
P24270	4.06	4.1	3.036	Catalase	2	1.16770	0.96218	0.85588	0.82858
P24369	23.4	23.64	43.060	Peptidyl-prolyl cis-trans isomerase B	15	1.23895	1.19927	0.80666	0.97312
P24452	4.03	4.03	7.670	Macrophage-capping protein	2	1.36005	3.03635	0.73483	2.32072
P24527	3.34	3.85	2.619	Leukotriene A-4 hydrolase	2	0.50088	0.73371	1.99532	1.45445
P24668	1.67	1.8	2.878	Cation-dependent mannose-6-phosphate receptor	1	0.96560	1.23091	1.03502	1.27955
P25444	3.59	3.67	8.191	40S ribosomal protein S2	3	0.88876	1.18711	1.12449	1.34907
P26039	13	15.81	2.873	Talin-1	7	0.97535	1.30840	1.02467	1.38438
P26040	28	30.84	18.260	Ezrin	20	0.60988	0.85958	1.63870	1.40976
P26041	4.28	14.62	7.279	Moesin	8	1.09306	3.31843	0.91432	3.06678
P26043	2	11.85	5.832	Radixin	7	0.76743	1.56360	1.30228	2.04859
P26150	2.47	2.8	5.630	3 beta-hydroxysteroid dehydrogenase/Delta 5-->4-isomerase type 3	2	1.54560	0.90035	0.64662	0.58724
P26231	59.2	60.05	34.000	Catenin alpha-1	49	1.05659	1.07773	0.94588	1.02121
P26369	2.27	2.39	2.105	Splicing factor U2AF 65 kDa subunit	1	0.77180	0.74619	1.29491	0.97210
P26443	15.7	16.36	11.470	Glutamate dehydrogenase 1, mitochondrial	7	1.06831	1.07222	0.93551	1.00813
P27546	2.54	2.77	0.800	Microtubule-associated protein 4	1	1.01102	1.04063	0.98852	1.03346
P27601	6.86	10.16	11.410	Guanine nucleotide-binding protein subunit alpha-13	5	0.64946	0.84916	1.53883	1.29515

P27659	1.35	1.76	1.737	60S ribosomal protein L3	1	0.88830	1.11773	1.12508	1.26323
P27773	48.7	49.07	48.320	Protein disulfide-isomerase A3	31	1.29153	0.75682	0.77382	0.59108
P28063	2.05	4.11	4.348	Proteasome subunit beta type-8	2	0.87430	1.16231	1.14310	1.34441
P28667	2.06	2.1	7.500	MARCKS-related protein	1	1.48026	2.68763	0.67516	1.84344
P28825	3.75	4.97	3.213	Meprin A subunit alpha	3	0.68532	0.51048	1.45832	0.74890
P28867	1.68	4.13	1.335	Protein kinase C delta type	1	0.68843	0.80901	1.45171	1.18450
P29452	2.1	3.42	3.980	Caspase-1	2	0.80864	0.65858	1.23591	0.82478
P29595	1.54	1.67	13.580	NEDD8	1	0.86208	1.05990	1.15930	1.23337
P30275	26.8	27.07	31.340	Creatine kinase U-type, mitochondrial	14	0.96130	0.42249	1.03964	0.44343
P30416	2	2.19	2.183	Peptidyl-prolyl cis-trans isomerase FKBP4	1	0.80228	1.33666	1.24571	1.67518
P30681	1.88	1.88	6.190	High mobility group protein B2	1	1.24177	4.57946	0.80483	3.71681
P30999	26.7	27.31	14.290	Catenin delta-1	16	0.91290	1.07935	1.09476	1.17929
P31001	17.9	21.88	24.310	Desmin	15	1.95530	2.76853	0.51113	1.57241
P31428	7.33	7.81	9.268	Dipeptidase 1	5	0.40846	0.27814	2.44676	0.67073
P31809	6.58	7.17	9.981	Carcinoembryonic antigen-related cell adhesion molecule 1	9	0.56821	0.24249	1.75888	0.43055
P32020	8.49	8.73	7.861	Non-specific lipid-transfer protein	6	1.21901	0.96777	0.81985	0.81216
P32883	3.63	4.69	17.460	GTPase KRas	3	0.80746	0.97539	1.23773	1.21094
P34022	1.8	2.02	5.419	Ran-specific GTPase-activating protein	1	0.71877	1.02961	1.39045	1.44031
P35276	4.49	7.02	19.180	Ras-related protein Rab-3D	7	1.02940	0.64525	0.97087	0.62960
P35278	9.92	9.97	22.220	Ras-related protein Rab-5C	6	0.93443	0.76280	1.06954	0.82201
P35279	13.6	15.99	37.500	Ras-related protein Rab-6A	12	0.96946	0.73741	1.03090	0.76359
P35282	4.36	4.45	10.810	Ras-related protein Rab-21	2	1.10458	0.98261	0.90479	0.89092
P35285	2.03	2.04	5.670	Ras-related protein Rab-22A	1	1.02593	1.61820	0.97415	1.58593
P35293	2.38	5.38	14.080	Ras-related protein Rab-18	3	1.07061	0.81851	0.93350	0.76913

P35486	11.7	12.11	15.130	Pyruvate dehydrogenase E1 component subunit alpha, somatic form, mitochondrial	6	1.00227	0.66378	0.99715	0.67716
P35550	1.84	2.21	3.364	rRNA 2'-O-methyltransferase fibrillarin	1	1.95957	2.99294	0.51002	1.53570
P35564	14.3	14.57	11.340	Calnexin	7	1.68492	1.37941	0.59315	0.79767
P35700	18.6	20.4	45.230	Peroxiredoxin-1	17	0.87867	1.32267	1.13741	1.50917
P35979	4.45	4.89	18.790	60S ribosomal protein L12	3	1.12461	1.18177	0.88867	1.05886
P37804	14	14.27	40.800	Transgelin	14	1.18748	1.52756	0.84162	1.29222
P38060	4.58	4.58	5.846	Hydroxymethylglutaryl-CoA lyase, mitochondrial	2	0.80356	0.51016	1.24372	0.63489
P38647	24.2	27.11	21.500	Stress-70 protein, mitochondrial	16	0.89687	0.57389	1.11433	0.65130
P39054	8.95	10.25	4.598	Dynamin-2	4	0.92108	1.07226	1.08504	1.16692
P39447	2.04	2.62	0.516	Tight junction protein ZO-1	1	1.10950	1.37552	0.90078	1.24655
P40124	2	2.22	5.907	Adenylyl cyclase-associated protein 1	3	1.26994	1.10343	0.78698	0.87703
P40142	16.6	17.02	13.000	Transketolase	10	0.94463	1.28547	1.05799	1.36481
P40240	2.35	2.35	3.097	CD9 antigen	1	0.88059	1.40468	1.13493	1.59914
P41105	2.7	2.7	5.109	60S ribosomal protein L28	1	0.70142	0.91226	1.42483	1.30664
P41216	2.33	3.46	1.288	Long-chain-fatty-acid--CoA ligase 1	1	1.06012	0.76223	0.94273	0.72916
P42125	4.05	4.37	9.689	Enoyl-CoA delta isomerase 1, mitochondrial	2	1.08089	0.97359	0.92462	0.90847
P42669	2	2.04	2.804	Transcriptional activator protein Pur-alpha	1	1.01726	1.11701	0.98245	1.10406
P42932	14.9	16.58	14.600	T-complex protein 1 subunit theta	7	0.77707	0.98259	1.28612	1.27283
P43274	3.27	3.43	5.023	Histone H1.4	1	0.89479	1.17206	1.11692	1.32065
P43276	2	2.21	4.933	Histone H1.5	1	0.87051	0.96643	1.14807	1.11626
P43406	11.3	12.61	4.981	Integrin alpha-V	5	1.09826	1.64311	0.90999	1.50724
P45376	1.64	1.79	4.430	Aldose reductase	1	0.72948	0.66004	1.37003	0.91427
P45952	11.9	14.1	15.910	Medium-chain specific acyl-CoA dehydrogenase, mitochondrial	6	1.00000	0.72963	0.99941	0.73740
P46460	12.3	13.45	8.602	Vesicle-fusing ATPase	6	0.94893	1.14341	1.05320	1.21367

P46467	7.41	7.8	8.108	Vacuolar protein sorting-associated protein 4B	4	0.86849	1.06632	1.15074	1.22910
P46471	7.7	10.96	9.007	26S protease regulatory subunit 7	5	0.91390	0.77037	1.09357	0.84952
P46978	2.77	2.82	1.560	Dolichyl-diphosphooligosaccharide--protein glycosyltransferase subunit STT3A	1	1.20550	1.39447	0.82904	1.18042
P47738	22.1	22.56	25.630	Aldehyde dehydrogenase, mitochondrial	15	0.89347	0.52128	1.11857	0.58642
P47753	4.08	4.26	8.741	F-actin-capping protein subunit alpha-1	4	0.97361	0.95767	1.02650	0.98676
P47757	2.84	3.27	3.610	F-actin-capping protein subunit beta	2	0.85756	1.00703	1.16541	1.18379
P47856	17.3	18.97	14.920	Glutamine--fructose-6-phosphate aminotransferase [isomerizing] 1	9	0.97791	0.69662	1.02199	0.71650
P47964	1.32	1.32	8.571	60S ribosomal protein L36	1	0.89421	1.16938	1.11765	1.31488
P48036	23.2	23.67	31.350	Annexin A5	13	1.23245	2.85013	0.81091	2.36009
P48428	1.6	1.61	6.481	Tubulin-specific chaperone A	1	0.91415	1.20865	1.09327	1.32939
P48678	12.8	13.52	10.230	Prelamin-A/C	7	1.09763	1.47388	0.91052	1.35053
P48758	3.63	3.7	9.025	Carbonyl reductase [NADPH] 1	2	0.78131	0.57141	1.27915	0.74091
P48771	4.04	4.15	27.710	Cytochrome c oxidase subunit 7A2, mitochondrial	4	1.31199	0.78452	0.76175	0.60123
P48774	2.68	2.9	3.125	Glutathione S-transferase Mu 5	1	0.78518	0.95841	1.27284	1.22776
P49312	2.03	4.37	7.500	Heterogeneous nuclear ribonucleoprotein A1	2	1.33435	1.09303	0.74898	0.82363
P49817	2.25	2.26	3.933	Caveolin-1	1	1.78961	2.95095	0.55845	1.67315
P50516	10.5	10.72	10.370	V-type proton ATPase catalytic subunit A	6	0.85625	0.89527	1.16720	1.05027
P50518	4.3	4.48	11.060	V-type proton ATPase subunit E 1	2	0.91370	0.81828	1.09381	0.90091
P50544	14.9	15.35	14.630	Very long-chain specific acyl-CoA dehydrogenase, mitochondrial	8	1.27865	0.92740	0.78161	0.72874
P51125	3.06	3.17	5.330	Calpastatin	2	0.90118	0.78578	1.10900	0.87456
P51150	22	21.98	60.870	Ras-related protein Rab-7a	14	0.88452	0.96482	1.12989	1.11139
P51174	2	3.3	3.023	Long-chain specific acyl-CoA dehydrogenase, mitochondrial	1	0.86076	0.53793	1.16107	0.62836

P51410	2.07	2.41	5.208	60S ribosomal protein L9	1	1.05261	1.24272	0.94946	1.18706
P51432	4.31	5.56	2.512	1-phosphatidylinositol 4,5-bisphosphate phosphodiesterase beta-3	3	0.76021	1.02058	1.31466	1.35415
P51437	5.48	5.73	17.920	Cathelin-related antimicrobial peptide	3	1.39619	4.21976	0.71581	3.09758
P51658	2.3	2.34	1.837	Estradiol 17-beta-dehydrogenase 2	1	1.75585	0.59165	0.56919	0.34110
P51660	9.36	9.7	7.075	Peroxisomal multifunctional enzyme type 2	4	1.29095	0.95119	0.77416	0.74323
P51661	7.29	7.67	10.360	Corticosteroid 11-beta-dehydrogenase isozyme 2	4	1.32285	0.94601	0.75550	0.72526
P51855	4.11	5.5	4.219	Glutathione synthetase	3	0.80673	0.88642	1.23884	1.10145
P51859	1.9	1.92	3.797	Hepatoma-derived growth factor	1	0.86750	1.10441	1.15206	1.28415
P51863	3.48	3.66	5.128	V-type proton ATPase subunit d 1	2	1.04528	1.27405	0.95611	1.23924
P51881	19.6	19.71	31.880	ADP/ATP translocase 2	14	1.21617	1.24758	0.82177	1.03165
P51912	2	2.28	2.351	Neutral amino acid transporter B(0)	1	1.13354	0.99405	0.88167	0.87811
P52196	7.2	7.35	17.850	Thiosulfate sulfurtransferase	5	0.86643	0.74395	1.15348	0.87325
P52480	25	26.61	28.440	Pyruvate kinase PKM	14	0.88696	1.28622	1.12679	1.47010
P52503	4.01	4.01	22.410	NADH dehydrogenase [ubiquinone] iron-sulfur protein 6, mitochondrial	3	1.29480	0.58123	0.77186	0.45665
P52795	4.81	5.75	8.986	Ephrin-B1	2	1.00257	0.84556	0.99685	0.84504
P52825	2.83	3.25	1.216	Carnitine O-palmitoyltransferase 2, mitochondrial	1	1.11635	0.94904	0.89525	0.85817
P52840	3.32	3.45	6.186	Sulfotransferase 1A1	2	0.79103	0.28601	1.26342	0.36242
P53395	5.17	5.64	3.112	Lipoamide acyltransferase component of branched-chain alpha-keto acid dehydrogenase complex, mitochondrial	2	1.05481	0.74855	0.94747	0.71537
P53657	1.88	5.15	5.575	Pyruvate kinase PKLR	3	0.85816	0.85405	1.16460	1.00085
P53986	7.09	7.23	8.519	Monocarboxylate transporter 1	5	0.83383	0.57132	1.24459	0.71627
P53994	13.7	14.13	27.830	Ras-related protein Rab-2A	7	1.06679	0.65012	0.93684	0.61931
P54071	10.5	12.87	15.270	Isocitrate dehydrogenase [NADP], mitochondrial	7	1.06211	0.78267	0.94097	0.74383
P54116	1.71	2.14	4.930	Erythrocyte band 7 integral membrane protein	1	0.77911	1.66060	1.28276	2.15977

P54227	2.22	2.3	8.054	Stathmin	1	0.71000	6.40242	1.40761	9.06677
P54763	1.37	2.47	0.704	Ephrin type-B receptor 2	1	0.97740	1.58375	1.02252	1.62923
P54775	2	2.11	2.153	26S protease regulatory subunit 6B	1	0.86737	0.70251	1.15223	0.81436
P54869	26.6	27.72	21.650	Hydroxymethylglutaryl-CoA synthase, mitochondrial	20	1.17655	0.33471	0.84944	0.28672
P55012	25.1	25.88	12.610	Solute carrier family 12 member 2	17	0.90397	1.30219	1.10558	1.47582
P55050	1.5	1.57	6.818	Fatty acid-binding protein, intestinal	1	0.60379	0.20963	1.65523	0.34243
P55088	4.06	5.94	7.121	Aquaporin-4	3	0.68204	0.30215	1.46533	0.45150
P55264	1.68	2.15	2.216	Adenosine kinase	1	0.58482	0.45814	1.70891	0.78766
P55292	1.38	2.48	0.887	Desmocollin-2	1	1.03785	1.29072	0.96297	1.25045
P55302	4	4.44	4.444	Alpha-2-macroglobulin receptor-associated protein	2	1.28276	1.25357	0.77911	0.98306
P56382	1.88	1.92	13.460	ATP synthase subunit epsilon, mitochondrial	2	0.98429	0.79328	1.01536	0.80566
P56383	2	2.28	4.795	ATP synthase F(0) complex subunit C2, mitochondrial	1	1.04892	0.89511	0.95280	0.85804
P56391	7.03	7.04	40.700	Cytochrome c oxidase subunit 6B1	6	1.22926	0.60241	0.81301	0.49397
P56393	2	2	8.750	Cytochrome c oxidase subunit 7B, mitochondrial	1	1.89504	1.58789	0.52738	0.84250
P56480	39.8	41.57	44.800	ATP synthase subunit beta, mitochondrial	36	1.14866	0.59361	0.87007	0.52041
P56528	6.57	6.75	10.530	ADP-ribosyl cyclase 1	6	0.97386	0.90549	1.02623	0.93659
P56546	1.98	2.29	2.247	C-terminal-binding protein 2	1	1.10383	0.88146	0.90540	0.80292
P56677	5.73	5.8	3.275	Suppressor of tumorigenicity 14 protein homolog	3	0.86710	0.91016	1.15259	1.05620
P57716	9.66	10.11	7.203	Nicastrin	6	1.01491	1.22722	0.98473	1.21885
P57759	5.55	5.78	14.120	Endoplasmic reticulum resident protein 29	4	1.09723	1.04702	0.91085	0.95683
P57776	4.35	4.47	8.541	Elongation factor 1-delta	6	1.21551	0.85473	0.82222	0.71533
P57780	37.2	37.75	21.050	Alpha-actinin-4	23	0.75819	0.77365	1.31815	1.02667
P58021	8.52	8.72	5.438	Transmembrane 9 superfamily member 2	5	1.42470	0.80431	0.70149	0.56332
P58252	26.8	27.91	16.320	Elongation factor 2	17	1.12689	1.44721	0.88687	1.30310
P58281	8.22	11.07	5.104	Dynamin-like 120 kDa protein, mitochondrial	5	1.20971	1.13453	0.82616	0.94345
P58771	2.04	7.45	12.680	Tropomyosin alpha-1 chain	6	1.01218	0.87234	0.98738	0.86656

P58774	2.01	6.36	9.507	Tropomyosin beta chain	4	1.12075	2.38501	0.89174	2.13969
P59242	2.16	4.95	2.267	Cingulin	3	1.28489	1.00630	0.77782	0.78111
P59999	6.86	6.86	17.860	Actin-related protein 2/3 complex subunit 4	4	0.98512	1.15269	1.01451	1.17706
P60335	7.01	7.32	16.010	Poly(rC)-binding protein 1	7	1.02369	1.44179	0.97628	1.42151
P60710	52.6	52.99	57.870	Actin, cytoplasmic 1	63	0.65025	0.73654	1.56236	1.15953
P60766	6.15	6.15	18.850	Cell division control protein 42 homolog	3	0.89195	1.02168	1.12048	1.15307
P60843	5.86	8.25	6.158	Eukaryotic initiation factor 4A-I	5	1.34853	1.91706	0.74111	1.38876
P60867	2.02	2.02	10.080	40S ribosomal protein S20	1	1.01648	1.24021	0.98321	1.23417
P61022	7.75	8.01	25.130	Calcineurin B homologous protein 1	4	0.71626	0.54140	1.39532	0.76134
P61082	1.77	1.79	6.011	NEDD8-conjugating enzyme Ubc12	1	0.92760	0.98310	1.07741	1.06563
P61089	7.41	7.67	28.290	Ubiquitin-conjugating enzyme E2 N	5	1.04382	1.27661	0.95745	1.24096
P61161	8.84	9.61	17.010	Actin-related protein 2	6	1.00912	0.96294	0.99037	0.95694
P61211	2.69	2.69	6.077	ADP-ribosylation factor-like protein 1	1	1.17487	1.18790	0.85065	1.01798
P61620	2.12	2.71	1.891	Protein transport protein Sec61 subunit alpha isoform 1	1	1.20795	1.24743	0.82736	1.03266
P61804	3.62	3.62	19.470	Dolichyl-diphosphooligosaccharide--protein glycosyltransferase subunit DAD1	3	1.87962	1.09259	0.53171	0.58426
P61924	2	2.08	6.215	Coatomer subunit zeta-1	1	0.89920	1.03620	1.11144	1.15866
P61961	2	2.06	17.650	Ubiquitin-fold modifier 1	1	0.98519	1.17142	1.01443	1.19553
P61979	19.7	19.98	23.540	Heterogeneous nuclear ribonucleoprotein K	10	1.27621	0.95533	0.78311	0.75683
P61982	9.71	14.41	27.940	14-3-3 protein gamma	10	0.97808	1.32206	1.02180	1.36430
P62071	6.77	6.84	17.650	Ras-related protein R-Ras2	3	0.93642	1.07496	1.06727	1.15668
P62075	3.16	3.18	26.320	Mitochondrial import inner membrane translocase subunit Tim13	2	0.92352	0.64042	1.08218	0.69032
P62077	2	2	13.250	Mitochondrial import inner membrane translocase subunit Tim8 B	1	0.77061	0.47402	1.29691	0.61848

P62137	7.78	7.87	11.820	Serine/threonine-protein phosphatase PP1-alpha catalytic subunit	4	1.16011	1.53653	0.86148	1.33020
P62141	2.01	5.49	7.339	Serine/threonine-protein phosphatase PP1-beta catalytic subunit	3	1.10291	1.16383	0.90616	1.06100
P62196	4.39	6.42	5.172	26S protease regulatory subunit 8	3	1.07892	0.89484	0.92630	0.83485
P62204	19.7	19.91	38.260	Calmodulin	13	0.70485	0.62373	1.41791	0.82900
P62242	1.55	1.65	4.327	40S ribosomal protein S8	1	1.16073	1.62311	0.86102	1.40600
P62245	3.11	3.26	12.310	40S ribosomal protein S15a	2	1.00687	1.16256	0.99259	1.15214
P62259	6.82	11.21	19.610	14-3-3 protein epsilon	9	0.91425	1.15798	1.09314	1.27128
P62264	4	4.08	15.230	40S ribosomal protein S14	2	1.01089	1.53421	0.98864	1.52479
P62267	2.06	2.11	7.692	40S ribosomal protein S23	1	1.13730	2.60579	0.87876	2.30373
P62281	5.4	5.71	16.460	40S ribosomal protein S11	3	0.78523	1.15402	1.27277	1.47513
P62305	2	2.01	11.960	Small nuclear ribonucleoprotein E	1	1.48961	1.42152	0.67092	0.95951
P62317	2.49	2.54	7.627	Small nuclear ribonucleoprotein Sm D2	1	1.42483	1.62976	0.70142	1.13044
P62320	1.51	1.57	7.143	Small nuclear ribonucleoprotein Sm D3	1	2.12144	2.63895	0.47110	1.25074
P62331	5.77	7.71	23.430	ADP-ribosylation factor 6	6	0.68599	1.00398	1.45689	1.46878
P62492	13	13.04	27.780	Ras-related protein Rab-11A	7	0.94415	0.78901	1.05853	0.84033
P62702	2.35	2.39	3.422	40S ribosomal protein S4, X isoform	1	0.83972	1.74840	1.19017	2.07409
P62717	2.65	2.81	5.682	60S ribosomal protein L18a	1	1.30497	3.40003	0.76585	2.68433
P62746	2.09	4.16	14.290	Rho-related GTP-binding protein RhoB	2	0.67025	0.90653	1.49110	1.35992
P62748	4.67	5.01	10.880	Hippocalcin-like protein 1	3	0.92249	0.78282	1.08339	0.85564
P62754	2.31	2.45	3.213	40S ribosomal protein S6	1	0.94657	1.33893	1.05583	1.42224
P62806	8.21	8.29	38.830	Histone H4	6	1.19010	2.70077	0.83977	2.26041
P62814	5.51	6.16	5.675	V-type proton ATPase subunit B, brain isoform	2	0.93316	0.88391	1.07099	0.95595
P62821	17.7	17.92	38.050	Ras-related protein Rab-1A	10	0.80396	0.72152	1.24311	0.90061
P62830	3.72	3.72	16.430	60S ribosomal protein L23	2	1.15396	1.45187	0.86607	1.27931

P62838	2	2.01	7.483	Ubiquitin-conjugating enzyme E2 D2	1	0.99236	1.37044	1.00710	1.38854
P62855	2	2	7.826	40S ribosomal protein S26	1	0.84769	1.05990	1.17898	1.25718
P62858	2.19	2.19	17.390	40S ribosomal protein S28	1	1.19613	1.13021	0.83554	0.94979
P62862	2	2	16.950	40S ribosomal protein S30	1	0.75739	0.91463	1.31954	1.21421
P62869	2.73	3.18	13.560	Transcription elongation factor B polypeptide 2	2	0.84737	0.86127	1.17942	1.02479
P62874	16.8	17.63	29.410	Guanine nucleotide-binding protein G(I)/G(S)/G(T) subunit beta-1	12	0.85895	1.03853	1.16352	1.20504
P62880	6.74	16	29.410	Guanine nucleotide-binding protein G(I)/G(S)/G(T) subunit beta-2	10	1.09694	1.07958	0.91109	0.98334
P62889	3.68	3.76	15.650	60S ribosomal protein L30	2	1.15545	1.34726	0.86495	1.17334
P62897	10.5	10.48	44.760	Cytochrome c, somatic	7	1.33841	0.61611	0.74672	0.45867
P62908	12.1	12.22	22.220	40S ribosomal protein S3	6	1.04076	1.27733	0.96027	1.23393
P62962	14.3	14.37	60.000	Profilin-1	13	0.68996	1.61054	1.44850	2.32957
P62984	8.51	8.67	31.250	Ubiquitin-60S ribosomal protein L40	7	0.87495	1.28902	1.14225	1.48144
P63017	28.3	34.98	32.200	Heat shock cognate 71 kDa protein	30	0.97546	1.07810	1.02455	1.10645
P63024	4.82	4.88	32.040	Vesicle-associated membrane protein 3	5	0.75922	0.84683	1.31637	1.12013
P63028	2.17	2.18	7.558	Translationally-controlled tumor protein	1	0.82755	1.03847	1.20766	1.26581
P63038	29.8	31.68	24.780	60 kDa heat shock protein, mitochondrial	21	0.90837	0.53495	1.10023	0.59259
P63073	2	2.04	3.226	Eukaryotic translation initiation factor 4E	1	0.86651	0.87660	1.15337	1.01717
P63101	15.5	15.86	26.120	14-3-3 protein zeta/delta	12	0.84315	1.04816	1.18533	1.26558
P63168	2.06	2.15	7.865	Dynein light chain 1, cytoplasmic	1	0.87083	1.15561	1.14765	1.33427
P63242	4.33	4.35	14.940	Eukaryotic translation initiation factor 5A-1	3	0.87436	1.15965	1.14302	1.33703
P63276	2	2.16	8.148	40S ribosomal protein S17	1	0.79217	0.91643	1.26161	1.16319
P63323	4	4.02	13.640	40S ribosomal protein S12	2	1.06145	1.00000	0.94155	0.95228
P67778	19.5	19.56	36.400	Prohibitin	12	1.21726	0.89181	0.82103	0.73166
P67984	2.7	2.82	8.594	60S ribosomal protein L22	1	1.26204	1.52679	0.79190	1.22502

P68033	6.87	39.24	48.010	Actin, alpha cardiac muscle 1	45	2.50494	2.61111	0.39898	1.14397
P68037	1.68	2.18	4.545	Ubiquitin-conjugating enzyme E2 L3	1	0.86729	1.02936	1.15234	1.18737
P68040	7.89	8.34	11.670	Guanine nucleotide-binding protein subunit beta-2-like 1	4	1.25402	1.16505	0.79696	0.94130
P68254	3.72	11.08	18.370	14-3-3 protein theta	8	0.74800	1.47960	1.33611	2.00079
P68368	5.31	21.94	26.120	Tubulin alpha-4A chain	14	1.04005	0.90289	0.96093	0.86270
P68372	5.63	21.23	24.490	Tubulin beta-4B chain	20	0.99109	0.81538	1.00839	0.83384
P68373	22.6	22.84	28.510	Tubulin alpha-1C chain	15	0.94142	1.31515	1.06159	1.41630
P68510	4.01	11.28	23.170	14-3-3 protein eta	9	0.73460	1.23317	1.36048	1.68862
P70124	4.06	5.07	10.130	Serpin B5	4	0.81025	1.03604	1.23346	1.28560
P70168	2.03	2.32	0.913	Importin subunit beta-1	1	0.97427	1.11833	1.02581	1.15163
P70296	4.03	4.28	14.440	Phosphatidylethanolamine-binding protein 1	2	0.82712	0.82176	1.20830	1.01223
P70302	4.71	5.41	3.066	Stromal interaction molecule 1	2	1.69376	1.00133	0.59005	0.58826
P70333	1.51	4.01	3.563	Heterogeneous nuclear ribonucleoprotein H2	2	0.61020	0.67482	1.63783	1.10756
P70349	2	2.19	11.110	Histidine triad nucleotide-binding protein 1	2	0.69434	0.75312	1.43936	1.09058
P70404	2.05	4.15	3.562	Isocitrate dehydrogenase [NAD] subunit gamma 1, mitochondrial	2	0.71326	0.57962	1.40118	0.81708
P70441	14.5	15.64	24.510	Na(+)/H(+) exchange regulatory cofactor NHE-RF1	8	0.45379	0.40392	2.20234	0.88833
P70460	6.15	6.46	8.000	Vasodilator-stimulated phosphoprotein	3	1.04515	1.16186	0.95624	1.12246
P70670	2.42	3.57	0.594	Nascent polypeptide-associated complex subunit alpha, muscle-specific form	1	0.89691	0.87341	1.11428	0.97069
P70695	2.02	2.15	3.540	Fructose-1,6-bisphosphatase isozyme 2	1	0.81937	0.59255	1.21974	0.72707
P80313	2.51	3.57	3.125	T-complex protein 1 subunit eta	2	1.02551	1.34224	0.97455	1.31575
P80314	10.9	11.82	13.640	T-complex protein 1 subunit beta	6	0.92610	1.01791	1.07916	1.09279
P80315	6.33	9.26	9.833	T-complex protein 1 subunit delta	4	0.94055	1.29552	1.06257	1.38082
P80316	12.3	13.78	9.797	T-complex protein 1 subunit epsilon	7	0.92909	1.14210	1.07568	1.23515
P80317	6.79	8.18	7.156	T-complex protein 1 subunit zeta	5	0.94757	1.10422	1.05471	1.16643

P80318	11.7	15.55	10.280	T-complex protein 1 subunit gamma	7	1.01173	1.26489	0.98782	1.25933
P83870	2	2.04	7.273	PHD finger-like domain-containing protein 5A	1	0.99239	1.18217	1.00707	1.19775
P83940	2	2.06	10.710	Transcription elongation factor B polypeptide 1	1	1.21842	1.12680	0.82025	0.92986
P84078	10.8	12.99	35.360	ADP-ribosylation factor 1	13	0.78677	0.88538	1.27026	1.13199
P84096	1.41	3.59	10.470	Rho-related GTP-binding protein RhoG	2	0.81984	1.17439	1.21904	1.46437
P97300	5.86	5.86	6.297	Neuroplastin	3	0.78386	0.63391	1.27499	0.81536
P97315	4.06	4.6	11.920	Cysteine and glycine-rich protein 1	3	1.02666	1.42857	0.97346	1.40622
P97370	1.45	1.57	7.914	Sodium/potassium-transporting ATPase subunit beta-3	3	1.07550	1.38871	0.92925	1.32001
P97371	13.2	13.82	27.310	Proteasome activator complex subunit 1	8	0.84554	0.86651	1.18198	1.03186
P97372	6.91	7.1	10.460	Proteasome activator complex subunit 2	3	0.77854	0.68544	1.28369	0.88559
P97384	19.1	24.85	24.850	Annexin A11	16	0.99654	1.07482	1.00288	1.08466
P97429	24.5	29.47	46.710	Annexin A4	20	1.02918	1.51356	0.97107	1.47330
P97447	2.81	2.83	8.214	Four and a half LIM domains protein 1	4	0.96600	0.90421	1.03458	0.94115
P97450	6.97	7.13	41.670	ATP synthase-coupling factor 6, mitochondrial	5	1.24424	0.83497	0.80323	0.68135
P97792	8.48	8.48	11.510	Coxsackievirus and adenovirus receptor homolog	5	0.91840	0.96690	1.08821	1.06462
P97805	8.69	8.85	20.180	Protein FAM3D	7	1.04461	0.64177	0.95673	0.61830
P97807	15.5	16.32	15.580	Fumarate hydratase, mitochondrial	10	0.77005	0.53918	1.29785	0.70824
P97823	2.07	2.13	4.783	Acyl-protein thioesterase 1	1	0.78280	0.85907	1.27671	1.10369
P97855	2.46	2.7	2.366	Ras GTPase-activating protein-binding protein 1	1	1.76847	1.69030	0.56513	0.94350
P97864	2	2.52	3.300	Caspase-7	1	1.42867	1.18576	0.69954	0.83451
P99024	21	21.32	24.550	Tubulin beta-5 chain	20	1.68355	2.47701	0.59363	1.58627
P99027	8.15	8.15	47.830	60S acidic ribosomal protein P2	6	0.83855	0.88805	1.19183	1.04983
P99028	7.44	7.44	41.570	Cytochrome b-c1 complex subunit 6, mitochondrial	5	1.27306	0.66406	0.78504	0.52469
P99029	7.77	7.86	30.480	Peroxiredoxin-5, mitochondrial	5	0.76837	0.95601	1.30068	1.25277
Q00612	3.2	3.88	1.359	Glucose-6-phosphate 1-dehydrogenase X	1	0.87449	1.10995	1.14285	1.27605
Q00623	4.11	5.1	6.818	Apolipoprotein A-I	2	1.51221	1.36562	0.66089	0.90103

Q01279	1.55	2.13	0.992	Epidermal growth factor receptor	1	1.46158	1.36523	0.68379	0.94486
Q01339	1.75	1.86	3.188	Beta-2-glycoprotein 1	1	1.34283	1.51536	0.74426	1.12781
Q01768	7.87	8.08	23.680	Nucleoside diphosphate kinase B	4	1.00690	1.19435	0.99256	1.19287
Q01853	29	29.86	14.890	Transitional endoplasmic reticulum ATPase	15	1.07989	1.02619	0.92547	0.95921
Q02053	3.42	5.03	1.512	Ubiquitin-like modifier-activating enzyme 1	2	1.00000	1.04789	0.99941	1.05571
Q02248	35.4	35.38	25.740	Catenin beta-1	25	1.09988	1.15715	0.90866	1.05859
Q02257	15.7	22.53	15.300	Junction plakoglobin	14	1.10392	1.07346	0.90533	0.97756
Q02819	14.4	15.14	17.650	Nucleobindin-1	7	1.14756	0.68823	0.87090	0.60515
Q03265	43.4	47.45	39.060	ATP synthase subunit alpha, mitochondrial	38	1.39264	0.81269	0.71764	0.62284
Q04447	2.5	8.12	8.399	Creatine kinase B-type	3	0.90499	1.09827	1.10433	1.22022
Q05144	2.22	4.53	13.020	Ras-related C3 botulinum toxin substrate 2	2	0.90713	1.29797	1.10173	1.44113
Q06138	3.53	5.61	2.933	Calcium-binding protein 39	2	0.59785	0.82672	1.67167	1.40827
Q06185	4.52	4.75	50.700	ATP synthase subunit e, mitochondrial	4	1.09178	0.97504	0.91539	0.89648
Q07076	17.1	17.97	19.220	Annexin A7	11	1.07119	1.96440	0.93299	1.85678
Q07113	2.33	3.77	0.282	Cation-independent mannose-6-phosphate receptor	1	0.95543	0.88934	1.04603	0.93619
Q07417	10.2	10.26	13.350	Short-chain specific acyl-CoA dehydrogenase, mitochondrial	5	1.02244	0.35938	0.97747	0.36607
Q08091	1.59	1.63	3.704	Calponin-1	1	1.17227	1.07145	0.85254	0.91818
Q08189	5.19	5.36	3.175	Protein-glutamine gamma-glutamyltransferase E	4	0.54115	0.44737	1.84682	0.82094
Q08509	7.77	8.46	4.385	Epidermal growth factor receptor kinase substrate 8	3	0.60867	0.71947	1.64196	1.18915
Q09199	7.54	8.05	9.608	Beta-1,4 N-acetylgalactosaminyltransferase 2	4	0.94422	0.49845	1.05845	0.54675
Q09200	2.55	2.87	2.064	Beta-1,4 N-acetylgalactosaminyltransferase 1	1	0.94289	0.45040	1.05995	0.50266
Q10470	2	2.65	1.859	Beta-1,4-mannosyl-glycoprotein 4-beta-N-acetylglucosaminyltransferase	1	0.89720	0.35884	1.11392	0.40214
Q148W0	6.29	7.21	2.638	Probable phospholipid-transporting ATPase IC	3	0.89517	0.82385	1.11644	0.93145
Q14AX6	3.32	4.93	1.213	Cyclin-dependent kinase 12	3	0.46839	0.37613	2.13369	0.80740

Q2KHK6	6.1	8.88	7.500	Gasdermin-C2	6	0.88809	0.80966	1.12534	0.86736
Q3THE2	12.2	12.37	33.140	Myosin regulatory light chain 12B	8	1.02711	1.25670	0.97303	1.22646
Q3THS6	1.36	1.6	1.772	S-adenosylmethionine synthase isoform type-2	1	0.70572	1.12042	1.41615	1.59630
Q3TMQ6	2.04	2.04	6.944	Angiogenin-4	1	0.80610	0.40868	1.23980	0.51482
Q3TTY5	2.02	8.44	3.395	Keratin, type II cytoskeletal 2 epidermal	7	0.36729	0.65897	2.72104	1.80394
Q3U0V1	1.79	2.83	1.738	Far upstream element-binding protein 2	1	0.99655	0.97967	1.00287	0.98670
Q3U1J4	4.65	6.63	1.754	DNA damage-binding protein 1	2	1.13795	1.09546	0.87826	0.96918
Q3U7R1	6	8.33	3.205	Extended synaptotagmin-1	4	1.35044	1.91028	0.74006	1.47852
Q3UDR8	2.5	2.5	5.476	Protein YIPF3	2	1.18289	0.67576	0.84489	0.56408
Q3ULJ0	2.03	2.46	2.279	Glycerol-3-phosphate dehydrogenase 1-like protein	1	0.91305	0.72260	1.09458	0.79606
Q3UMR5	1.5	2.35	3.143	Calcium uniporter protein, mitochondrial	2	1.24555	0.81736	0.80239	0.67777
Q3UQ44	12.1	15.32	3.492	Ras GTPase-activating-like protein IQGAP2	6	0.86927	0.81605	1.14971	0.94230
Q3URD3	2.1	2.71	1.183	Sarcolemmal membrane-associated protein	1	1.38142	1.87648	0.72347	1.36580
Q3USF0	2.06	2.11	3.581	UDP-GlcNAc:betaGal beta-1,3-N-acetylglucosaminyltransferase 6	1	0.99858	0.72660	1.00083	0.73090
Q3UTJ2	2.39	5.33	1.356	Sorbin and SH3 domain-containing protein 2	2	1.18956	1.87848	0.84015	1.61178
Q3UW53	5.12	8.87	4.428	Protein Niban	6	1.06089	1.00328	0.94205	0.95350
Q3UZZ6	2.16	3.4	4.746	Sulfotransferase 1 family member D1	2	0.69074	0.65210	1.44686	0.95743
Q3V0K9	11.9	14.85	10.630	Plastin-1	8	0.59808	0.52875	1.67102	0.88676
Q3V3R1	3.33	5.65	3.992	Monofunctional C1-tetrahydrofolate synthase, mitochondrial	4	1.24402	0.81546	0.80337	0.66097
Q3V3R4	1.58	2.7	0.509	Integrin alpha-1	1	0.94031	1.59391	1.06285	1.74066
Q45VN2	2.56	2.56	11.580	Alpha-defensin 20	1	2.53674	7.09883	0.39397	3.25101
Q501J6	2.82	13.42	10.000	Probable ATP-dependent RNA helicase DDX17	7	1.23512	1.31422	0.80916	1.06913
Q5FWI3	1.47	3.66	0.868	Transmembrane protein 2	1	0.97224	1.20172	1.02795	1.23784
Q5FWK3	3.01	3.18	4.328	Rho GTPase-activating protein 1	2	1.06592	1.12382	0.93760	1.06470

Q5JCS9	3.94	4.07	4.570	UDP-GlcNAc:betaGal beta-1,3-N-acetylglucosaminyltransferase 3	2	1.20128	0.89443	0.83195	0.74781
Q5JCT0	1.38	4.14	6.178	Beta-1,3-galactosyl-O-glycosyl-glycoprotein beta-1,6-N-acetylglucosaminyltransferase 3	3	1.04944	0.69661	0.95233	0.66742
Q5SRX1	2.03	3.39	1.775	TOM1-like protein 2	2	0.73251	0.85335	1.36435	1.17133
Q5SUC9	1.42	1.49	2.817	Protein SCO1 homolog, mitochondrial	1	1.62614	1.20894	0.61459	0.74751
Q5XJY5	5.08	5.4	5.479	Coatomer subunit delta	3	1.07919	0.94467	0.92607	0.88000
Q60597	4.19	4.87	1.857	2-oxoglutarate dehydrogenase, mitochondrial	2	1.03839	0.71354	0.96246	0.69894
Q60598	7.99	8.06	9.707	Src substrate cortactin	6	1.11619	0.88543	0.89537	0.79326
Q60604	10.7	12.87	8.252	Adseverin	6	0.53732	0.44862	1.85997	0.82717
Q60605	12.7	12.82	40.400	Myosin light polypeptide 6	12	1.03613	1.06549	0.96456	1.03124
Q60634	7.69	8.02	10.980	Flotillin-2	4	1.16630	2.38748	0.85691	2.07741
Q60668	4.31	4.54	6.197	Heterogeneous nuclear ribonucleoprotein D0	2	0.48584	0.31413	2.05707	0.63998
Q60766	2.12	2.39	2.445	Immunity-related GTPase family M protein 1	1	1.04594	1.15730	0.95552	1.11082
Q60770	2.27	3	1.689	Syntaxin-binding protein 3	1	0.75171	1.14781	1.32951	1.53851
Q60854	10.2	10.54	17.200	Serpin B6	6	0.74337	0.75679	1.34443	1.02694
Q60864	4.76	5.08	4.052	Stress-induced-phosphoprotein 1	2	0.89883	0.97751	1.11190	1.09632
Q60870	5.93	6.1	14.590	Receptor expression-enhancing protein 5	4	1.39987	1.21445	0.71393	0.86041
Q60902	2.15	2.83	2.315	Epidermal growth factor receptor substrate 15-like 1	2	0.86428	1.25583	1.15635	1.46194
Q60930	17.8	18.24	33.560	Voltage-dependent anion-selective channel protein 2	10	1.50605	1.17590	0.66359	0.76665
Q60931	5.58	8.21	12.010	Voltage-dependent anion-selective channel protein 3	5	1.31345	1.12545	0.76090	0.86057
Q60932	18.9	19.63	39.530	Voltage-dependent anion-selective channel protein 1	14	1.47980	1.01428	0.67537	0.68977
Q61029	2.92	3.77	2.876	Lamina-associated polypeptide 2, isoforms beta/delta/epsilon/gamma	1	2.44106	5.12634	0.40942	1.92658
Q61081	1.69	1.96	2.902	Hsp90 co-chaperone Cdc37	1	1.16686	1.22616	0.85650	1.05657
Q61112	1.92	2.23	1.939	45 kDa calcium-binding protein	1	1.50697	0.75815	0.66319	0.50584

Q61165	1.76	2.3	0.854	Sodium/hydrogen exchanger 1	1	0.91912	0.79287	1.08736	0.86921
Q61171	9.41	9.66	26.260	Peroxiredoxin-2	6	0.71552	1.28756	1.39675	1.79842
Q61190	2	2.08	3.725	Interleukin-10 receptor subunit beta	1	0.97515	0.44909	1.02488	0.46306
Q61205	2.65	3.87	3.879	Platelet-activating factor acetylhydrolase IB subunit gamma	2	0.78293	1.00759	1.27649	1.29892
Q61206	2	3.24	3.930	Platelet-activating factor acetylhydrolase IB subunit beta	2	0.62694	0.69428	1.59410	1.11346
Q61207	7.83	8.42	8.079	Sulfated glycoprotein 1	6	0.92804	1.11168	1.07690	1.21992
Q61233	7	10.4	5.742	Plastin-2	4	1.00800	1.82886	0.99148	1.84205
Q61335	7.11	7.39	13.470	B-cell receptor-associated protein 31	5	1.37829	0.99178	0.72511	0.71750
Q61418	1.52	4.58	1.606	H(+)/Cl(-) exchange transporter 4	2	1.01194	1.22087	0.98762	1.22073
Q61420	2	2.3	3.571	CMP-sialic acid transporter	1	1.30752	0.71352	0.76435	0.54869
Q61425	12.9	13.84	21.020	Hydroxyacyl-coenzyme A dehydrogenase, mitochondrial	7	1.05329	0.54161	0.94885	0.53694
Q61503	2.69	3.37	1.563	5'-nucleotidase	1	0.57726	0.87731	1.73130	1.52810
Q61526	2	4.59	0.747	Receptor tyrosine-protein kinase erbB-3	2	0.77900	0.57652	1.28294	0.74412
Q61543	26.5	28.32	10.300	Golgi apparatus protein 1	14	1.19784	1.12305	0.83434	0.94400
Q61598	7.54	7.8	12.130	Rab GDP dissociation inhibitor beta	6	0.68076	0.80895	1.46808	1.19764
Q61599	1.41	2.89	5.500	Rho GDP-dissociation inhibitor 2	2	0.70905	1.50294	1.40950	2.13124
Q61656	18	18.25	11.890	Probable ATP-dependent RNA helicase DDX5	8	1.42977	1.53458	0.69900	1.06570
Q61696	3.82	13.07	11.540	Heat shock 70 kDa protein 1A	11	0.71070	0.81933	1.40624	1.15764
Q61735	5.67	7.74	12.870	Leukocyte surface antigen CD47	4	1.14971	1.73626	0.86927	1.52369
Q61739	22.1	24.71	11.270	Integrin alpha-6	12	1.03344	1.49275	0.96707	1.45016
Q61753	2	2.21	2.064	D-3-phosphoglycerate dehydrogenase	1	0.86110	0.98514	1.16063	1.15031
Q61765	2.09	13.57	17.550	Keratin, type I cuticular Ha1	8	1.13851	0.96068	0.87782	0.77917
Q61792	3.33	4.57	7.605	LIM and SH3 domain protein 1	2	0.75547	0.92588	1.32290	1.23521
Q61847	2.97	3.19	2.983	Meprin A subunit beta	2	0.79358	0.64882	1.25936	0.82126
Q61937	4.03	4.65	5.479	Nucleophosmin	2	1.69096	1.44611	0.59103	0.85925

Q61941	8.11	9.95	3.959	NAD(P) transhydrogenase, mitochondrial	4	1.08140	1.34711	0.92418	1.25546
Q61990	3.23	6.51	15.750	Poly(rC)-binding protein 2	5	0.98871	1.21246	1.01082	1.23617
Q62086	2.1	2.57	5.932	Serum paraoxonase/arylesterase 2	3	1.36891	0.83616	0.73008	0.61400
Q62087	5.9	6.03	10.170	Serum paraoxonase/lactonase 3	4	1.49198	0.85377	0.66985	0.57304
Q62167	4.19	6.7	4.834	ATP-dependent RNA helicase DDX3X	3	1.03576	1.11243	0.96491	1.08013
Q62186	4.69	4.69	13.370	Translocon-associated protein subunit delta	3	1.94149	1.14426	0.51476	0.56563
Q62189	1.5	1.57	2.787	U1 small nuclear ribonucleoprotein A	1	0.96731	1.19484	1.03318	1.24197
Q62261	46.5	50.4	9.268	Spectrin beta chain, non-erythrocytic 1	21	1.13722	1.13153	0.87882	0.99651
Q62273	7.81	8.64	4.871	Sulfate transporter	4	0.77805	0.45623	1.28450	0.59650
Q62313	6.1	6.2	7.365	Trans-Golgi network integral membrane protein 1	3	0.86257	0.62527	1.15865	0.72891
Q62351	4.13	4.91	2.752	Transferrin receptor protein 1	2	1.27075	1.31702	0.78647	1.04370
Q62393	5.05	6.59	13.840	Tumor protein D52	3	0.82847	0.69182	1.20632	0.83485
Q62425	7.68	7.68	45.120	NADH dehydrogenase [ubiquinone] 1 alpha subcomplex subunit 4	5	1.22791	1.11271	0.81391	0.89971
Q62433	2.65	2.94	4.061	Protein NDRG1	1	1.16033	0.57353	0.86131	0.49670
Q62465	8.75	8.75	10.840	Synaptic vesicle membrane protein VAT-1 homolog	4	1.00099	1.06011	0.99842	1.06594
Q62468	52	52.44	30.830	Villin-1	32	0.46761	0.64040	2.13728	1.37172
Q62469	14.4	17.23	5.857	Integrin alpha-2	9	0.92141	0.87632	1.08466	0.95334
Q62470	9.1	10.07	4.368	Integrin alpha-3	5	0.86219	0.98014	1.15915	1.14293
Q63844	5.81	5.94	12.630	Mitogen-activated protein kinase 3	4	1.00865	1.14113	0.99084	1.13986
Q64133	9.8	11.05	9.316	Amine oxidase [flavin-containing] A	6	1.07076	0.58986	0.93336	0.56321
Q64310	2.08	2.16	2.974	Surfeit locus protein 4	1	1.49237	1.29528	0.66968	0.87268
Q64433	9.72	9.85	52.940	10 kDa heat shock protein, mitochondrial	6	0.70271	0.82056	1.42221	1.18010
Q64435	2.03	5.29	4.896	UDP-glucuronosyltransferase 1-6	4	1.84155	0.88012	0.54270	0.48054
Q64444	7.35	7.35	17.700	Carbonic anhydrase 4	10	0.44759	0.18322	2.23285	0.41324
Q64471	2	2.4	4.167	Glutathione S-transferase theta-1	1	0.98957	0.84928	1.00994	0.86292

Q64478	8.8	8.88	27.780	Histone H2B type 1-H	7	1.78097	6.07568	0.56116	3.84553
Q64518	24.5	26.3	13.390	Sarcoplasmic/endoplasmic reticulum calcium ATPase 3	12	1.22110	0.76063	0.81845	0.62896
Q64521	24.1	24.62	17.470	Glycerol-3-phosphate dehydrogenase, mitochondrial	11	1.31733	1.21825	0.75866	0.92317
Q64704	2.04	2.13	2.768	Syntaxin-3	1	0.55558	0.71661	1.79885	1.29690
Q64727	28.2	29.03	12.950	Vinculin	15	1.01249	1.19859	0.98708	1.18912
Q64735	3.79	3.9	3.934	Complement component receptor 1-like protein	3	1.11390	1.30939	0.89722	1.19393
Q66JS6	2.03	2.1	3.042	Eukaryotic translation initiation factor 3 subunit J-B	1	0.66341	0.68281	1.50648	1.03487
Q68FD5	40.4	44.79	13.430	Clathrin heavy chain 1	25	0.94885	0.98979	1.05328	1.04748
Q69ZF7	26.1	26.81	15.180	Metal transporter CNNM4	15	0.84970	0.54503	1.17619	0.65393
Q69ZN7	2.3	6.96	0.781	Myoferlin	2	1.04767	2.31123	0.95394	2.21781
Q6GQT9	10.1	11.76	4.036	Nodal modulator 1	4	1.41682	1.01157	0.70539	0.71629
Q6GSS7	7.25	7.35	26.920	Histone H2A type 2-A	6	1.30226	2.68210	0.76744	2.12851
Q6IFZ6	1.78	9.61	8.217	Keratin, type II cytoskeletal 1b	11	1.42787	0.99424	0.69993	0.70012
Q6IRU2	6.45	8.67	14.110	Tropomyosin alpha-4 chain	5	1.06784	1.62912	0.93592	1.38970
Q6IRU5	3.97	4.05	8.297	Clathrin light chain B	3	0.88907	0.97782	1.12410	1.10583
Q6NZJ6	1.75	2.95	0.687	Eukaryotic translation initiation factor 4 gamma 1	1	1.15324	1.40880	0.86661	1.23401
Q6P069	2.48	2.48	9.596	Sorcin	2	0.74324	0.95599	1.34467	1.28626
Q6P5E4	9.04	10.85	2.515	UDP-glucose:glycoprotein glucosyltransferase 1	4	1.02360	0.92456	0.97637	0.91083
Q6PB66	7.41	12.78	2.945	Leucine-rich PPR motif-containing protein, mitochondrial	6	0.96361	0.84531	1.03715	0.88320
Q6PD26	2.91	3.42	4.505	GPI transamidase component PIG-S	2	1.59041	1.25189	0.62840	0.79206
Q6PDN3	1.89	6.25	0.824	Myosin light chain kinase, smooth muscle	2	1.58502	1.76502	0.63053	1.11552
Q6PDQ2	1.5	2.48	0.470	Chromodomain-helicase-DNA-binding protein 4	1	1.09687	1.19235	0.91115	1.09299
Q6PHU5	2.98	4.88	1.455	Sortilin	2	0.97039	1.04408	1.02990	1.07457
Q6PHZ2	1.33	1.55	2.204	Calcium/calmodulin-dependent protein kinase type II subunit delta	1	1.15993	0.98015	0.86161	0.85253

Q6Q473	14.6	15.66	7.251	Calcium-activated chloride channel regulator 4	7	0.76729	0.58974	1.30251	0.75332
Q6R0H7	2.07	5.59	2.295	Guanine nucleotide-binding protein G(s) subunit alpha isoforms XLas	3	0.81571	1.34113	1.22520	1.66274
Q6URW6	39.3	55.53	13.750	Myosin-14	29	1.35069	0.97825	0.73993	0.73214
Q6X893	6.15	6.19	4.747	Choline transporter-like protein 1	3	0.94640	0.75563	1.05601	0.80180
Q6ZQ38	6.6	11.55	3.577	Cullin-associated NEDD8-dissociated protein 1	4	1.12923	1.27946	0.88504	1.14291
Q6ZQI3	2.5	2.57	5.155	Malectin	2	1.18542	1.00255	0.84308	0.84546
Q6ZQM8	5.43	5.48	4.520	UDP-glucuronosyltransferase 1-7C	4	1.99414	0.81637	0.50117	0.41551
Q76MZ3	7.32	7.61	4.584	Serine/threonine-protein phosphatase 2A 65 kDa regulatory subunit A alpha isoform	3	0.89966	0.95508	1.11087	1.06477
Q78KK3	4.16	5.46	6.404	Solute carrier family 22 member 18	4	1.41959	0.77110	0.70401	0.54749
Q78PY7	5.88	9.25	3.516	Staphylococcal nuclease domain-containing protein 1	4	1.17148	1.48382	0.85312	1.27747
Q7TMK9	2.08	3.64	2.247	Heterogeneous nuclear ribonucleoprotein Q	2	1.38018	1.67213	0.72412	1.21843
Q7TN37	4.26	5.3	1.566	Transient receptor potential cation channel subfamily M member 4	2	0.98068	0.55223	1.01910	0.57132
Q7TNS2	1.55	1.55	10.530	Mitochondrial inner membrane organizing system protein 1	1	0.92794	1.01383	1.07702	1.09853
Q7TPR4	10.7	28.13	14.910	Alpha-actinin-1	16	0.95739	1.49911	1.04389	1.58340
Q7TST0	2.02	2.48	2.161	Butyrophilin-like protein 1	1	0.79143	0.37774	1.26279	0.47990
Q7TSV6	2	2.36	1.733	Putative aspartate aminotransferase, cytoplasmic 2	2	0.62369	0.54782	1.60241	0.88315
Q7TT45	2.03	4.54	3.118	Ras-related GTP-binding protein D	3	0.61601	0.91117	1.62239	1.48723
Q80SW1	6.1	6.28	6.415	Putative adenosylhomocysteinase 2	4	0.95094	0.99525	1.05097	1.05872
Q80TH2	7.01	7.5	1.854	Protein LAP2	3	0.90885	0.80980	1.09965	0.89762
Q80U72	5.68	6.21	2.233	Protein scribble homolog	3	1.11372	1.34513	0.89736	1.20819
Q80UG5	5.47	6.09	3.259	Septin-9	3	1.05294	1.66013	0.94916	1.58965
Q80V26	9.05	10.31	11.520	Inositol monophosphatase 3	5	0.84174	0.46763	1.18731	0.54913

Q80VA0	4.54	5.22	3.196	N-acetylgalactosaminyltransferase 7	2	1.40649	0.87265	0.71057	0.63005
Q80VG1	2.12	2.39	1.716	Sex comb on midleg-like protein 4	1	0.86618	0.96830	1.15381	1.12400
Q80VQ1	2	3.22	3.626	Leucine-rich repeat-containing protein 1	2	1.22753	0.71925	0.81416	0.58914
Q80W54	2.73	2.86	1.684	CAAX prenyl protease 1 homolog	1	1.06268	1.11396	0.94046	1.05378
Q80X90	101	100.9	23.330	Filamin-B	56	1.09189	1.15438	0.91531	1.06239
Q80XN0	12.5	12.64	16.330	D-beta-hydroxybutyrate dehydrogenase, mitochondrial	6	1.18807	0.61326	0.84120	0.51312
Q80Y14	1.48	1.63	3.947	Glutaredoxin-related protein 5, mitochondrial	1	0.57465	0.48886	1.73916	0.87883
Q80YN3	2.12	2.44	1.106	Breast carcinoma-amplified sequence 1 homolog	1	0.85778	0.72173	1.16511	0.84834
Q80Z19	55	56.37	8.284	Mucin-2 (Fragments)	27	0.61995	0.31061	1.61207	0.49941
Q810Q5	3.43	3.44	18.070	Normal mucosa of esophagus-specific gene 1 protein	3	1.16816	0.52291	0.85554	0.45022
Q811D0	5.24	5.91	3.425	Disks large homolog 1	3	0.92709	0.85821	1.07801	0.93410
Q8BFR5	28.4	30.03	33.410	Elongation factor Tu, mitochondrial	15	0.96071	0.56431	1.04028	0.59180
Q8BFW7	2.35	2.49	1.468	Lipoma-preferred partner homolog	1	0.97935	1.41797	1.02049	1.45606
Q8BFZ9	7.43	7.63	17.350	Erlin-2	8	1.33568	1.10131	0.74824	0.82511
Q8BG05	9.78	9.86	14.250	Heterogeneous nuclear ribonucleoprotein A3	5	0.97261	0.38084	1.02755	0.39398
Q8BGC4	5.41	7.24	9.019	Zinc-binding alcohol dehydrogenase domain-containing protein 2	3	1.62352	0.73852	0.61558	0.44046
Q8BGD8	2	2	13.920	Cytochrome c oxidase assembly factor 6 homolog	1	1.27696	0.91546	0.78265	0.72082
Q8BGH2	4.11	4.9	4.691	Sorting and assembly machinery component 50 homolog	2	1.49721	1.45431	0.66752	0.96727
Q8BH24	4.52	5.19	6.221	Transmembrane 9 superfamily member 4	5	1.22438	1.00413	0.81626	0.82535
Q8BH43	4.2	4.22	4.628	Wiskott-Aldrich syndrome protein family member 2	2	0.99645	1.24750	1.00297	1.26045
Q8BH79	2.03	4.11	2.428	Anoctamin-10	2	1.30664	1.32954	0.76487	1.02308
Q8BH95	16.5	17.73	33.100	Enoyl-CoA hydratase, mitochondrial	13	1.05458	0.56931	0.94768	0.54648
Q8BHG3	2.04	2.1	3.683	Cell cycle control protein 50B	1	0.98068	0.86964	1.01910	0.89512
Q8BHL4	2.37	2.5	3.652	Retinoic acid-induced protein 3	1	0.46666	0.26259	2.14160	0.56813
Q8BHN3	3.46	3.57	2.331	Neutral alpha-glucosidase AB	2	1.27537	1.20510	0.78362	0.94608

Q8BHY3	1.45	2.75	0.938	Anoctamin-1	1	1.06088	1.57267	0.94206	1.49054
Q8BI08	1.92	1.92	6.286	Protein MAL2	1	1.16462	0.85401	0.85814	0.73731
Q8BI84	2.7	4.84	0.725	Melanoma inhibitory activity protein 3	2	1.71627	1.32538	0.58232	0.77647
Q8BIJ6	1.42	2.45	1.482	Isoleucine--tRNA ligase, mitochondrial	2	0.89448	0.66653	1.11730	0.74923
Q8BJ64	6.58	7.37	6.040	Choline dehydrogenase, mitochondrial	4	1.11175	1.09068	0.89895	0.98468
Q8BK30	2.32	2.38	16.350	NADH dehydrogenase [ubiquinone] flavoprotein 3, mitochondrial	2	0.87364	1.60958	1.14396	1.96368
Q8BK48	1.45	5.49	5.903	Pyrethroid hydrolase Ces2e	3	1.09615	0.49474	0.91174	0.45673
Q8BK64	2.11	2.56	3.550	Activator of 90 kDa heat shock protein ATPase homolog 1	1	0.91562	1.08293	1.09152	1.20032
Q8BKX1	2.82	3.12	2.804	Brain-specific angiogenesis inhibitor 1-associated protein 2	2	0.79205	1.33640	1.26181	1.69700
Q8BLF1	3.3	3.38	4.412	Neutral cholesterol ester hydrolase 1	2	1.28901	1.02601	0.77533	0.80294
Q8BM96	1.82	3.31	2.038	Probable G-protein coupled receptor 128	2	0.71448	0.59332	1.39879	0.83496
Q8BMD8	15.6	17.01	16.000	Calcium-binding mitochondrial carrier protein SCaMC-1	10	1.26662	0.96879	0.78904	0.76171
Q8BMF4	11.4	12.28	11.530	Dihydrolipoyllysine-residue acetyltransferase component of pyruvate dehydrogenase complex, mitochondrial	6	0.83350	0.56453	1.19905	0.68583
Q8BMJ3	2.3	3.81	10.420	Eukaryotic translation initiation factor 1A, X-chromosomal	2	0.99378	1.12461	1.00566	1.13855
Q8BMK4	22.2	24.4	20.520	Cytoskeleton-associated protein 4	14	1.29169	1.23359	0.77372	0.95546
Q8BMP6	4.1	4.16	2.857	Golgi resident protein GCP60	2	0.96600	0.47940	1.03458	0.49939
Q8BMS1	23.5	24.51	12.980	Trifunctional enzyme subunit alpha, mitochondrial	14	1.35482	1.29695	0.73767	0.93197
Q8BN82	2	2.13	1.616	Sialin	1	1.20168	1.40022	0.83168	1.17159
Q8BP67	2.02	2.03	6.369	60S ribosomal protein L24	1	1.02187	1.57510	0.97802	1.54982
Q8BR63	6.21	6.21	17.870	Protein FAM177A1	4	1.06884	0.96932	0.93504	0.91892
Q8BR70	2	2	3.390	Protein YIPF6	1	0.94858	0.59817	1.05358	0.63404

Q8BRF7	2.9	4.33	3.756	Sec1 family domain-containing protein 1	3	0.84861	0.78385	1.17770	0.93201
Q8BT60	7.06	7.74	7.692	Copine-3	5	0.94471	1.23440	1.05790	1.31134
Q8BTM8	28	33.08	6.347	Filamin-A	14	1.26419	2.25817	0.79055	1.88658
Q8BTY2	3.12	3.66	1.934	Sodium bicarbonate cotransporter 3	2	0.81992	0.73063	1.21891	0.89596
Q8BU31	6.17	7.95	22.950	Ras-related protein Rap-2c	5	0.86487	1.05438	1.15557	1.23092
Q8BWM0	2	2.09	2.344	Prostaglandin E synthase 2	1	1.15726	0.96494	0.86360	0.83838
Q8BWT1	8.12	8.95	12.590	3-ketoacyl-CoA thiolase, mitochondrial	4	0.94506	0.93733	1.05751	1.07415
Q8BXK9	4.03	4.48	9.562	Chloride intracellular channel protein 5	2	0.56193	0.52141	1.77854	0.93281
Q8BXN9	5.3	8.26	6.486	Transmembrane protein 87A	4	1.01350	0.93296	0.98609	0.92602
Q8C0Z1	5.41	5.43	3.243	Protein ITFG3	3	0.78647	0.53720	1.27075	0.68368
Q8C102	4.77	6.35	3.118	Polypeptide N-acetylgalactosaminyltransferase 5	3	1.16849	0.73725	0.85530	0.63573
Q8C129	1.57	2.38	1.171	Leucyl-cystinyl aminopeptidase	1	1.07461	1.19970	0.93002	1.12867
Q8C196	2.25	3.42	0.733	Carbamoyl-phosphate synthase [ammonia], mitochondrial	1	1.49138	1.10214	0.67012	0.74577
Q8C407	2	2.06	4.472	Protein YIPF4	1	1.10946	1.22480	0.90081	1.11000
Q8C8R3	3.85	7.85	0.462	Ankyrin-2	3	0.85254	1.02586	1.17227	1.21600
Q8CAQ8	20.2	22.15	13.470	Mitochondrial inner membrane protein	10	1.10314	0.93446	0.90597	0.84954
Q8CBW3	2.37	2.63	1.871	Abl interactor 1	1	0.77145	0.86899	1.29549	1.12220
Q8CC88	7.09	8.22	1.942	von Willebrand factor A domain-containing protein 8	4	1.39390	0.65821	0.71699	0.48429
Q8CCS6	2.78	4.43	2.318	Polyadenylate-binding protein 2	1	0.81959	0.71594	1.21940	0.87829
Q8CGK3	10	13.01	5.374	Lon protease homolog, mitochondrial	7	0.90863	0.64532	1.09991	0.71384
Q8CI51	1.92	1.94	1.523	PDZ and LIM domain protein 5	1	0.71448	1.16154	1.39879	1.63459
Q8CI85	2.3	2.3	2.542	Carbonic anhydrase 12	1	1.02276	0.47617	0.97716	0.48110
Q8CJ19	1.41	6.37	0.803	Protein-methionine sulfoxide oxidase MICAL3	2	1.02048	1.02597	0.97935	1.01088
Q8JZN7	1.37	2.77	2.258	Mitochondrial Rho GTPase 2	2	1.01061	0.72612	0.98892	0.71852
Q8JZQ2	5.33	6.21	2.494	AFG3-like protein 2	2	1.06886	1.14141	0.93503	1.06769

Q8K0C5	15	14.97	40.720	Zymogen granule membrane protein 16	11	0.58493	0.24714	1.70858	0.44091
Q8K0C9	2.21	2.48	1.613	GDP-mannose 4,6 dehydratase	1	0.93042	0.82377	1.07415	0.89740
Q8K0J2	10.3	10.49	12.850	UDP-GlcNAc:betaGal beta-1,3-N-acetylglucosaminyltransferase 7	6	0.86778	0.92841	1.15168	1.07532
Q8K135	3.9	4.94	1.622	Dyslexia-associated protein KIAA0319-like protein	2	0.89823	0.89867	1.11264	1.00705
Q8K2B3	19.7	19.82	15.510	Succinate dehydrogenase [ubiquinone] flavoprotein subunit, mitochondrial	10	1.21745	0.77000	0.82090	0.63334
Q8K353	2	2	9.615	Cysteine-rich and transmembrane domain-containing protein 1	2	0.76070	0.68625	1.31381	0.90707
Q8K3J1	4.89	4.89	12.740	NADH dehydrogenase [ubiquinone] iron-sulfur protein 8, mitochondrial	4	1.14666	0.83342	0.87158	0.73083
Q8K419	38	38.71	42.940	Galectin-4	47	1.13233	1.63418	0.88261	1.46827
Q8K4L4	2.35	3.12	4.259	Protein POF1B	2	1.08959	0.95448	0.91724	0.87877
Q8K4Z5	1.89	3.04	1.138	Splicing factor 3A subunit 1	1	1.20253	0.76732	0.83109	0.64390
Q8QZR3	5.32	5.48	3.763	Pyrethroid hydrolase Ces2a	3	1.10918	0.45614	0.90103	0.41349
Q8QZT1	8.1	8.6	8.962	Acetyl-CoA acetyltransferase, mitochondrial	4	1.05522	0.66669	0.94711	0.63753
Q8R059	4.34	4.47	5.476	UDP-glucose 4-epimerase	2	1.14293	1.07476	0.87443	0.94650
Q8R081	6.12	6.28	4.778	Heterogeneous nuclear ribonucleoprotein L	3	1.12939	0.74547	0.95168	0.71389
Q8R088	3.81	3.96	9.825	Golgi phosphoprotein 3-like	2	0.83674	0.66444	1.19440	0.78401
Q8R0W0	6.39	10.69	4.337	Epiplakin	6	1.07436	0.88432	0.93024	0.83705
Q8R143	2.01	2.26	5.172	Pituitary tumor-transforming gene 1 protein-interacting protein	1	0.94498	1.16595	1.05759	1.24058
Q8R1F1	1.73	4.37	2.136	Niban-like protein 1	2	0.87611	1.39676	1.14074	1.60235
Q8R1S0	2	3.95	3.151	Ubiquinone biosynthesis monooxygenase COQ6	2	1.20299	0.51885	0.83077	0.43365
Q8R2S8	13.7	13.8	10.530	CD177 antigen	9	1.13685	2.12896	0.87911	1.91399

Q8R3B1	2.04	2.51	2.116	1-phosphatidylinositol 4,5-bisphosphate phosphodiesterase delta-1	1	0.75270	0.63214	1.32777	0.84442
Q8R3G9	10	10.11	19.570	Tetraspanin-8	8	0.74910	0.57975	1.33414	0.77591
Q8R4G6	1.54	3.87	1.081	Alpha-1,6-mannosylglycoprotein 6-beta-N-acetylglucosaminyltransferase A	1	0.84351	0.93775	1.18482	1.11990
Q8R4N0	2.95	3.1	3.550	Citrate lyase subunit beta-like protein, mitochondrial	1	0.82840	0.46883	1.20644	0.56679
Q8VBT0	1.77	1.77	3.237	Thioredoxin-related transmembrane protein 1	1	0.99988	1.43015	0.99952	1.43814
Q8VCA5	2.08	4.47	3.908	Transmembrane protease serine 4	2	1.15272	1.76865	0.86700	1.54272
Q8VCC2	6.89	7.17	5.841	Liver carboxylesterase 1	4	1.18190	0.57080	0.84559	0.50125
Q8VCF1	10.1	10.13	16.380	Soluble calcium-activated nucleotidase 1	5	1.23088	0.86616	0.81195	0.71927
Q8VCS3	1.67	1.96	1.467	Glycosaminoglycan xylosylkinase	1	0.91961	0.96263	1.08677	1.03853
Q8VCT4	2.02	4.91	4.602	Carboxylesterase 1D	3	0.91036	0.66326	1.09781	0.73108
Q8VCW8	10.9	12.99	10.240	Acyl-CoA synthetase family member 2, mitochondrial	6	0.83879	0.57666	1.19149	0.68626
Q8VDD5	64.4	66.81	16.730	Myosin-9	35	1.33771	1.90676	0.74710	1.45775
Q8VDJ3	2.17	3.52	0.710	Vigilin	1	1.08402	1.27089	0.92194	1.18375
Q8VDM4	4.63	5.07	1.872	26S proteasome non-ATPase regulatory subunit 2	2	0.87447	0.81186	1.14287	0.93144
Q8VDN2	88.7	88.72	41.350	Sodium/potassium-transporting ATPase subunit alpha-1	94	0.73463	0.39859	1.36042	0.53739
Q8VED5	2	12.45	7.533	Keratin, type II cytoskeletal 79	12	0.46440	0.77555	2.15206	1.67915
Q8VEK3	4.63	4.79	2.375	Heterogeneous nuclear ribonucleoprotein U	2	1.02917	1.35223	0.97109	1.32739
Q8VEM8	4.77	5.03	7.003	Phosphate carrier protein, mitochondrial	3	1.24665	1.54241	0.80168	1.24873
Q8VHF2	6.05	6.5	5.535	Cadherin-related family member 5	5	0.47853	0.36196	2.08851	0.75978
Q8VIJ6	3.01	3.6	1.144	Splicing factor, proline- and glutamine-rich	1	1.13860	1.33638	0.87775	1.18128
Q91V12	2	2.04	2.887	Cytosolic acyl coenzyme A thioester hydrolase	1	0.90035	0.98009	1.11003	1.09452
Q91V41	10.4	13.41	31.160	Ras-related protein Rab-14	7	0.84188	0.70322	1.18712	0.83382
Q91V61	2.04	2.13	4.050	Sideroflexin-3	1	1.03793	1.33801	0.96289	1.28871
Q91V76	2.01	2.12	3.810	Ester hydrolase C11orf54 homolog	1	0.55648	0.21748	1.79594	0.39295

Q91V92	2.06	2.93	0.733	ATP-citrate synthase	1	0.78865	1.15003	1.26725	1.46714
Q91VA1	2.75	4.65	3.536	Choline transporter-like protein 4	3	0.47822	0.33141	2.08986	0.68962
Q91VD9	27.1	28.77	18.980	NADH-ubiquinone oxidoreductase 75 kDa subunit, mitochondrial	14	1.27551	0.77763	0.78354	0.61052
Q91VM9	5.34	6.61	14.850	Inorganic pyrophosphatase 2, mitochondrial	4	0.66607	0.64060	1.50046	0.96697
Q91VR2	14.6	15.16	24.160	ATP synthase subunit gamma, mitochondrial	9	1.26872	1.29060	0.78773	0.99935
Q91VR8	2	2.02	14.670	Protein BRICK1	1	1.17159	1.64426	0.85304	1.41112
Q91VS7	2.02	2.06	8.387	Microsomal glutathione S-transferase 1	1	1.38080	1.11107	0.72379	0.80905
Q91VW3	2.24	2.34	16.130	SH3 domain-binding glutamic acid-rich-like protein 3	1	0.58902	0.73094	1.69674	1.24891
Q91W90	10.2	10.59	18.470	Thioredoxin domain-containing protein 5	7	0.85171	0.56993	1.17342	0.67720
Q91WD5	5.93	6.65	6.695	NADH dehydrogenase [ubiquinone] iron-sulfur protein 2, mitochondrial	3	1.15487	1.10178	0.86539	0.96378
Q91WG0	5.63	5.84	7.665	Acylcarnitine hydrolase	4	1.29048	0.31507	0.77445	0.26161
Q91WG8	4.17	4.41	3.047	Bifunctional UDP-N-acetylglucosamine 2-epimerase/N-acetylmannosamine kinase	2	1.10521	0.95220	0.90427	0.86796
Q91WL0	2.56	3.13	3.000	Epidermal growth factor receptor kinase substrate 8-like protein 3	2	0.61049	0.88558	1.63706	1.45595
Q91WQ3	2.86	4.01	3.409	Tyrosine--tRNA ligase, cytoplasmic	2	0.63676	0.68452	1.56953	1.08041
Q91WS0	2.37	2.69	12.040	CDGSH iron-sulfur domain-containing protein 1	1	1.39119	0.68188	0.71838	0.49282
Q91X52	2.05	2.22	3.279	L-xylulose reductase	1	0.91964	1.01813	1.08675	1.09745
Q91XA2	14.8	15.62	21.880	Golgi membrane protein 1	15	0.98496	0.65118	1.01467	0.66274
Q91Y74	2.66	3.31	4.204	CMP-N-acetylneuraminate-beta-galactosamide-alpha-2,3-sialyltransferase 4	2	0.88447	0.82421	1.12995	0.97024
Q91Y97	4.03	6.85	7.418	Fructose-bisphosphate aldolase B	3	0.87244	0.65078	1.14554	0.75021
Q91YD6	1.78	2.12	1.164	Villin-like protein	1	0.78928	1.54513	1.26622	1.96834

Q91YQ5	24.8	25.23	22.530	Dolichyl-diphosphooligosaccharide--protein glycosyltransferase subunit 1	13	1.55452	1.05073	0.64291	0.66009
Q91YT0	7.11	11.57	12.280	NADH dehydrogenase [ubiquinone] flavoprotein 1, mitochondrial	6	1.12295	1.02707	0.88999	0.91887
Q91YT8	2	2.58	1.493	Transmembrane protein 63A	1	0.96590	0.50429	1.03469	0.52495
Q91YW3	6.92	8.91	4.960	DnaJ homolog subfamily C member 3	3	1.24195	0.78810	0.80471	0.63393
Q91ZJ5	2.91	3.22	3.346	UTP--glucose-1-phosphate uridylyltransferase	2	0.69868	0.60738	1.43043	0.87527
Q91ZN5	2.64	3.18	4.408	Adenosine 3'-phospho 5'-phosphosulfate transporter 1	2	1.07326	0.78802	0.93119	0.73824
Q921F2	2	2.4	2.657	TAR DNA-binding protein 43	1	1.15194	1.09308	0.86759	0.95410
Q921G7	4.4	4.61	7.143	Electron transfer flavoprotein-ubiquinone oxidoreductase, mitochondrial	4	1.40399	0.93335	0.71184	0.67014
Q921H8	4.6	4.68	5.660	3-ketoacyl-CoA thiolase A, peroxisomal	3	1.16477	0.77137	0.85803	0.66978
Q921I1	6.13	7.08	4.161	Serotransferrin	3	0.94719	1.30997	1.05513	1.33080
Q921X9	10.6	10.65	8.897	Protein disulfide-isomerase A5	6	1.15972	0.97773	0.86177	0.82766
Q922B2	4	4.44	4.192	Aspartate--tRNA ligase, cytoplasmic	2	1.15827	1.29737	0.86284	1.12298
Q922D8	1.34	1.73	0.749	C-1-tetrahydrofolate synthase, cytoplasmic	1	0.93099	1.00390	1.07349	1.09359
Q922Q1	5.1	5.37	9.172	MOSC domain-containing protein 2, mitochondrial	3	1.17986	1.27302	0.84706	1.08551
Q922Q8	2.92	2.97	3.909	Leucine-rich repeat-containing protein 59	2	1.23705	1.29869	0.80790	1.05687
Q922R8	17.9	18.36	20.450	Protein disulfide-isomerase A6	9	1.32658	0.80085	0.75337	0.61078
Q922U2	2.9	13.9	8.621	Keratin, type II cytoskeletal 5	16	0.37287	0.55810	2.68033	1.50495
Q922W5	1.6	1.6	4.207	Pyrroline-5-carboxylate reductase 1, mitochondrial	1	1.25244	0.65048	0.79797	0.52221
Q924N4	2.23	5.29	1.826	Solute carrier family 12 member 6	3	1.07694	1.36397	0.92801	1.27345
Q93092	3.89	4.64	6.231	Transaldolase	2	0.86125	0.97993	1.16042	1.14636
Q99J99	6.37	6.49	11.450	3-mercaptopyruvate sulfurtransferase	4	0.83356	0.31901	1.19897	0.39216
Q99JI4	2.01	3.1	2.314	26S proteasome non-ATPase regulatory subunit 6	1	1.04720	0.90989	0.95436	0.87362
Q99JI6	6.79	7.17	14.130	Ras-related protein Rap-1b	3	0.79030	1.19352	1.26460	1.53873

Q99JR1	1.91	2.14	4.037	Sideroflexin-1	1	0.78424	1.12909	1.27436	1.43906
Q99JW5	17.4	18.45	29.210	Epithelial cell adhesion molecule	12	1.01769	0.92133	0.98203	0.86257
Q99JX3	3.52	3.78	4.213	Golgi reassembly-stacking protein 2	2	1.09758	0.88158	0.91056	0.80803
Q99JY0	19.3	19.72	18.320	Trifunctional enzyme subunit beta, mitochondrial	10	1.28705	1.01493	0.77651	0.78688
Q99JY9	6.92	7.24	12.680	Actin-related protein 3	6	1.00372	0.94719	0.99571	0.94822
Q99K01	8.05	10.81	6.734	Pyridoxal-dependent decarboxylase domain-containing protein 1	5	1.18248	0.55924	0.84518	0.47824
Q99K23	2.06	2.36	1.735	Ufm1-specific protease 2	1	1.27792	1.31651	0.78206	1.06762
Q99K30	3.8	4.52	2.743	Epidermal growth factor receptor kinase substrate 8-like protein 2	2	0.90203	1.11738	1.10796	1.24122
Q99K48	4.73	5.38	4.440	Non-POU domain-containing octamer-binding protein	2	1.74678	1.09488	0.57214	0.64255
Q99KE1	2	2.15	2.547	NAD-dependent malic enzyme, mitochondrial	1	0.89286	0.52739	1.11934	0.59391
Q99KF1	4.13	4.93	15.740	Transmembrane emp24 domain-containing protein 9	7	1.29017	0.87397	0.77464	0.68320
Q99KI0	35.7	38.59	22.950	Aconitate hydratase, mitochondrial	22	0.98958	0.68420	1.00993	0.70644
Q99KJ8	4.23	4.23	6.219	Dynactin subunit 2	2	0.90045	0.88844	1.10990	0.99659
Q99KN9	4.5	4.61	3.487	Clathrin interactor 1	2	1.07538	0.83565	0.92936	0.78500
Q99KQ4	1.48	2.13	1.426	Nicotinamide phosphoribosyltransferase	1	0.85463	1.34353	1.16941	1.58066
Q99KR7	1.37	2.99	2.913	Peptidyl-prolyl cis-trans isomerase F, mitochondrial	1	1.23090	0.84633	0.81193	0.70564
Q99KV1	3.4	3.4	6.145	DnaJ homolog subfamily B member 11	3	1.28687	1.03728	0.77662	0.81022
Q99L04	1.75	1.8	2.556	Dehydrogenase/reductase SDR family member 1	1	1.16295	1.28814	0.85937	1.10804
Q99L47	3.18	3.23	8.086	Hsc70-interacting protein	3	0.66775	0.99942	1.49669	1.50640
Q99LB2	11.5	12.16	18.280	Dehydrogenase/reductase SDR family member 4	6	1.26670	1.20093	0.78899	0.94877
Q99LC3	2.4	2.5	7.324	NADH dehydrogenase [ubiquinone] 1 alpha subcomplex subunit 10, mitochondrial	2	1.25047	1.35261	0.79923	1.08281
Q99LC5	14.8	15.73	32.430	Electron transfer flavoprotein subunit alpha, mitochondrial	11	1.01486	0.46379	0.98477	0.46644

Q99LF4	3.03	3.13	1.188	tRNA-splicing ligase RtcB homolog	1	0.99941	0.94833	1.00000	0.95386
Q99LM2	1.66	2.31	2.783	CDK5 regulatory subunit-associated protein 3	1	1.26764	0.77298	0.78840	0.61311
Q99LP6	3.5	4.92	9.677	GrpE protein homolog 1, mitochondrial	3	0.84711	0.73735	1.17979	0.87869
Q99LR1	1.82	1.91	2.010	Monoacylglycerol lipase ABHD12	1	1.56504	2.29384	0.63858	1.47369
Q99LX0	5.28	5.44	15.870	Protein DJ-1	3	0.78398	0.92676	1.27480	1.20493
Q99M74	2	3.85	4.651	Keratin, type II cuticular Hb2	5	2.93742	0.89507	0.34023	0.30638
Q99P72	2.88	4.22	1.807	Reticulon-4	2	4.19863	6.31349	0.23803	1.43466
Q99PL5	18	24.6	5.545	Ribosome-binding protein 1	11	1.29889	1.11672	0.76943	0.86114
Q99PT1	4.28	5.58	17.160	Rho GDP-dissociation inhibitor 1	3	0.78295	0.75294	1.27647	0.97249
Q9CPP6	4.41	5.18	27.590	NADH dehydrogenase [ubiquinone] 1 alpha subcomplex subunit 5	3	1.28446	0.72381	0.77808	0.58039
Q9CPQ1	5.65	5.71	27.630	Cytochrome c oxidase subunit 6C	4	1.07404	0.72524	0.93051	0.68680
Q9CPQ8	3.54	3.69	31.070	ATP synthase subunit g, mitochondrial	2	1.05371	1.26259	0.94846	1.20479
Q9CPT4	3.19	3.25	10.840	UPF0556 protein C19orf10 homolog	2	1.16386	0.89283	0.85870	0.77782
Q9CPW4	2.97	3.1	9.934	Actin-related protein 2/3 complex subunit 5	2	1.09235	1.34741	0.91492	1.25907
Q9CPY7	2.14	5	2.312	Cytosol aminopeptidase	3	0.88814	0.70147	1.12528	0.80012
Q9CQ10	2.01	2.61	3.571	Charged multivesicular body protein 3	1	0.91550	1.08527	1.09166	1.19192
Q9CQ22	2	2.07	8.075	Ragulator complex protein LAMTOR1	2	0.87981	1.06273	1.13594	1.21451
Q9CQ54	2.1	2.1	10.830	NADH dehydrogenase [ubiquinone] 1 subunit C2	1	1.03396	1.04990	0.96658	1.02371
Q9CQ62	7.13	7.53	8.657	2,4-dienoyl-CoA reductase, mitochondrial	5	1.11840	0.84542	0.89360	0.76114
Q9CQ69	2.25	2.25	15.850	Cytochrome b-c1 complex subunit 8	2	1.12097	0.83043	0.89156	0.72944
Q9CQ75	3.22	3.66	16.160	NADH dehydrogenase [ubiquinone] 1 alpha subcomplex subunit 2	2	0.95955	0.71668	1.04154	0.74859
Q9CQA3	15.4	17.05	24.820	Succinate dehydrogenase [ubiquinone] iron-sulfur subunit, mitochondrial	9	1.05830	0.80229	0.94436	0.75420

Q9CQC6	1.59	5.13	3.580	Basic leucine zipper and W2 domain-containing protein 1	2	1.27042	1.37536	0.78667	1.08273
Q9CQC9	4	4.18	9.596	GTP-binding protein SAR1b	2	1.29211	1.15169	0.77347	0.89491
Q9CQE8	2.32	2.42	3.279	UPF0568 protein C14orf166 homolog	1	1.05877	0.77326	0.94394	0.73171
Q9CQH3	5.91	5.93	16.930	NADH dehydrogenase [ubiquinone] 1 beta subcomplex subunit 5, mitochondrial	3	1.33169	1.10999	0.75048	0.82602
Q9CQI3	2.8	2.82	4.930	Glia maturation factor beta	1	0.92276	1.01256	1.08307	1.10360
Q9CQJ8	2.19	2.47	12.850	NADH dehydrogenase [ubiquinone] 1 beta subcomplex subunit 9	2	1.33600	1.00143	0.74806	0.75436
Q9CQM5	3.32	3.32	15.450	Thioredoxin domain-containing protein 17	2	0.71200	0.70157	1.40367	0.98616
Q9CQN1	4.75	7.57	4.816	Heat shock protein 75 kDa, mitochondrial	4	1.22919	0.83904	0.81306	0.69343
Q9CQQ7	7.95	8.4	16.800	ATP synthase F(0) complex subunit B1, mitochondrial	6	0.99177	0.96806	1.00770	0.98878
Q9CQR2	4.03	4.88	20.480	40S ribosomal protein S21	2	1.18902	1.39076	0.84053	1.20719
Q9CQS8	1.7	1.74	10.420	Protein transport protein Sec61 subunit beta	1	1.41183	1.11227	0.70788	0.79213
Q9CQU0	2.04	2.04	5.294	Thioredoxin domain-containing protein 12	1	1.13945	0.88964	0.87710	0.78503
Q9CQV8	2.3	14.46	24.390	14-3-3 protein beta/alpha	10	0.75800	1.00411	1.31848	1.33400
Q9CQW2	3.89	4.04	12.370	ADP-ribosylation factor-like protein 8B	3	1.04342	1.54694	0.95782	1.49863
Q9CQX2	3.82	4.83	12.330	Cytochrome b5 type B	2	1.58338	0.69326	0.63119	0.44273
Q9CQX5	1.49	1.49	3.557	Claudin domain-containing protein 1	1	0.88675	0.85936	1.12705	0.97442
Q9CQZ5	2.02	3.09	9.924	NADH dehydrogenase [ubiquinone] 1 alpha subcomplex subunit 6	2	1.06235	0.91187	0.94075	0.86452
Q9CR21	2.61	2.66	11.540	Acyl carrier protein, mitochondrial	3	0.92522	0.87568	1.08019	0.95163
Q9CR51	2	2	9.322	V-type proton ATPase subunit G 1	1	0.58983	0.60876	1.69440	1.03774
Q9CR57	2.61	2.7	5.991	60S ribosomal protein L14	1	0.77678	1.26244	1.28661	1.62992
Q9CR61	4	4.05	13.870	NADH dehydrogenase [ubiquinone] 1 beta subcomplex subunit 7	2	1.20207	1.09913	0.83141	0.91912

Q9CR67	2	2.07	4.858	Transmembrane protein 33	1	1.45875	2.32945	0.68511	1.60561
Q9CR68	10.9	11.1	17.520	Cytochrome b-c1 complex subunit Rieske, mitochondrial	7	1.09166	0.69915	0.91549	0.64609
Q9CRB9	6.87	6.98	14.100	Coiled-coil-helix-coiled-coil-helix domain-containing protein 3, mitochondrial	4	1.16514	0.91679	0.85776	0.81062
Q9CVB6	20.6	21.72	29.000	Actin-related protein 2/3 complex subunit 2	11	0.97019	1.18717	1.03012	1.23433
Q9CWJ9	2.14	2.52	1.689	Bifunctional purine biosynthesis protein PURH	1	0.59882	0.70236	1.66897	1.16282
Q9CWS0	3.17	3.34	6.667	N(G),N(G)-dimethylarginine dimethylaminohydrolase 1	2	0.85948	1.05174	1.16281	1.23008
Q9CWZ3	2.6	2.6	6.322	RNA-binding protein 8A	1	1.22325	1.23765	0.81701	1.01833
Q9CWZ7	1.78	2.32	3.205	Gamma-soluble NSF attachment protein	2	1.00469	1.06337	0.99474	1.05585
Q9CX00	4.83	5.23	4.696	IST1 homolog	2	0.88018	1.18670	1.13545	1.35663
Q9CXI5	7.49	8.11	24.020	Mesencephalic astrocyte-derived neurotrophic factor	5	1.18350	1.05404	0.84445	0.89912
Q9CXW3	1.62	1.7	3.057	Calcyclin-binding protein	1	1.12367	1.32517	0.88941	1.18577
Q9CXW4	3.39	4.73	15.730	60S ribosomal protein L11	3	1.05152	1.48025	0.95044	1.42009
Q9CXZ1	8.41	8.52	25.140	NADH dehydrogenase [ubiquinone] iron-sulfur protein 4, mitochondrial	4	1.21086	0.71564	0.82537	0.59640
Q9CY50	4.01	4.01	6.643	Translocon-associated protein subunit alpha	4	1.74126	1.14204	0.57396	0.66718
Q9CY58	2	2.02	1.474	Plasminogen activator inhibitor 1 RNA-binding protein	1	0.86044	1.24424	1.16151	1.45396
Q9CYN2	3.07	3.27	6.195	Signal peptidase complex subunit 2	2	1.31727	1.33351	0.75869	1.01765
Q9CYR0	2.06	2.06	5.263	Single-stranded DNA-binding protein, mitochondrial	1	1.21184	1.38295	0.82470	1.14743
Q9CYZ2	5.97	6.14	21.820	Tumor protein D54	4	1.19303	1.08716	0.83771	0.91359
Q9CZ13	17.4	19.86	17.080	Cytochrome b-c1 complex subunit 1, mitochondrial	10	1.29229	0.65040	0.77336	0.50375
Q9CZ30	3.58	3.98	5.051	Obg-like ATPase 1	2	1.12672	1.10274	0.88701	0.98958
Q9CZ42	2.11	2.11	2.624	ATP-dependent (S)-NAD(P)H-hydrate dehydratase	1	0.84164	0.80242	1.18746	0.96053
Q9CZB0	2.02	2.06	7.692	Succinate dehydrogenase cytochrome b560 subunit, mitochondrial	1	1.25928	0.97836	0.79364	0.78117
Q9CZD3	2	2.14	1.509	Glycine--tRNA ligase	1	1.04017	1.61439	0.96081	1.56052

Q9CZS1	18.6	22.21	18.690	Aldehyde dehydrogenase X, mitochondrial	11	0.77906	0.33873	1.28284	0.44606
Q9CZU6	18.2	19.15	17.670	Citrate synthase, mitochondrial	11	1.07934	0.86702	0.92594	0.80137
Q9CZW5	1.64	3.4	1.146	Mitochondrial import receptor subunit TOM70	3	0.99527	1.09527	1.00416	1.08726
Q9CZX8	8.02	9.14	28.280	40S ribosomal protein S19	4	0.77979	1.03224	1.28165	1.33379
Q9CZY3	4.05	4.26	11.560	Ubiquitin-conjugating enzyme E2 variant 1	2	0.79103	1.17532	1.26343	1.49734
Q9D051	7.55	10.06	13.370	Pyruvate dehydrogenase E1 component subunit beta, mitochondrial	5	0.89908	0.48588	1.11159	0.54563
Q9D0E1	5.75	6.29	5.213	Heterogeneous nuclear ribonucleoprotein M	5	1.01711	0.79751	0.98260	0.78276
Q9D0F3	3.02	3.55	3.482	Protein ERGIC-53	2	1.36969	1.07047	0.72966	0.78835
Q9D0I4	2.05	2.43	2.990	Syntaxin-17	1	0.85865	0.57270	1.16393	0.67062
Q9D0I9	2.88	3.97	2.424	Arginine--tRNA ligase, cytoplasmic	2	1.05397	0.98889	0.94823	0.94334
Q9D0J8	2	2	10.890	Parathymosin	1	1.13754	1.70991	0.87857	1.51138
Q9D0K2	9.6	9.81	7.692	Succinyl-CoA:3-ketoacid coenzyme A transferase 1, mitochondrial	4	0.95522	0.66655	1.04626	0.70802
Q9D0M3	7.07	7.17	13.540	Cytochrome c1, heme protein, mitochondrial	5	1.97972	1.48657	0.50482	0.65333
Q9D154	7.15	9.58	16.360	Leukocyte elastase inhibitor A	7	0.81515	0.57143	1.22604	0.69674
Q9D1D4	10	13.41	24.660	Transmembrane emp24 domain-containing protein 10	10	1.43155	0.86879	0.69813	0.60562
Q9D1G1	4.12	14.21	35.820	Ras-related protein Rab-1B	8	1.08893	0.72255	0.91779	0.67798
Q9D1G5	2	2.61	4.603	Leucine-rich repeat-containing protein 57	1	0.73365	1.04996	1.36225	1.43897
Q9D1Q6	8.41	9.05	9.113	Endoplasmic reticulum resident protein 44	4	1.06245	0.74172	0.94066	0.70275
Q9D1R9	2	2.04	5.983	60S ribosomal protein L34	1	1.11937	1.02447	0.89283	0.92022
Q9D279	18.4	18.73	14.350	Mitotic interactor and substrate of PLK1	10	0.69222	0.59341	1.44377	0.86426
Q9D2G2	5.99	7.92	5.507	Dihydrolipoyllysine-residue succinyltransferase component of 2-oxoglutarate dehydrogenase complex, mitochondrial	3	0.98757	0.69397	1.01199	0.70944
Q9D2Q8	10.2	10.16	54.810	Protein S100-A14	9	1.29296	2.48496	0.77296	1.92045

Q9D306	6.65	6.95	6.485	Alpha-1,3-mannosyl-glycoprotein 4-beta-N-acetylglucosaminyltransferase C	5	1.16105	0.67085	0.86078	0.58540
Q9D309	8.94	8.94	19.150	Protein FAM3B	5	1.35252	0.75138	0.73893	0.56152
Q9D312	44.3	47.24	54.060	Keratin, type I cytoskeletal 20	47	1.32463	0.55274	0.75448	0.43742
Q9D3D9	8.35	8.36	26.790	ATP synthase subunit delta, mitochondrial	7	1.19653	0.88041	0.83525	0.74182
Q9D3P8	2	2.15	6.803	Plasminogen receptor (KT)	1	0.99349	0.72928	1.00595	0.73807
Q9D646	12.1	13.33	18.880	Keratin, type I cuticular Ha4	7	3.06996	0.96695	0.32554	0.33123
Q9D6J6	6.14	6.53	12.900	NADH dehydrogenase [ubiquinone] flavoprotein 2, mitochondrial	4	1.05692	0.82911	0.94558	0.78871
Q9D6R2	21.1	21.47	21.580	Isocitrate dehydrogenase [NAD] subunit alpha, mitochondrial	10	0.88597	0.55615	1.12804	0.63299
Q9D6Y7	4.05	4.11	8.584	Mitochondrial peptide methionine sulfoxide reductase	2	0.63002	0.78424	1.58632	1.24949
Q9D7A8	2	2.1	4.255	Armadillo repeat-containing protein 1	1	0.94657	1.18164	1.05583	1.25517
Q9D7L8	1.67	1.74	3.065	Transmembrane and immunoglobulin domain-containing protein 1	1	0.65883	0.52322	1.51694	0.79851
Q9D7N9	6.08	6.58	9.880	Adipocyte plasma membrane-associated protein	4	1.16633	0.90636	0.85688	0.78298
Q9D7T1	2.34	2.52	4.348	Rab15 effector protein	2	0.90175	0.91803	1.10829	1.01632
Q9D7Z6	32.2	33.64	21.030	Calcium-activated chloride channel regulator 1	19	0.56108	0.33416	1.78124	0.58093
Q9D816	24.4	25.29	23.470	Cytochrome P450 2C55	15	1.81001	0.35614	0.55216	0.21944
Q9D819	8.68	9.09	19.720	Inorganic pyrophosphatase	5	0.82719	0.68874	1.20819	0.85551
Q9D855	10.8	10.81	46.850	Cytochrome b-c1 complex subunit 7	8	1.09835	0.62008	0.90992	0.56080
Q9D869	3.27	3.32	14.290	Calcineurin B homologous protein 2	2	0.56286	0.36289	1.77558	0.64121
Q9D898	4.02	4.08	16.340	Actin-related protein 2/3 complex subunit 5-like protein	2	0.98180	0.95093	1.01794	0.97688
Q9D8B3	8.24	9.6	14.290	Charged multivesicular body protein 4b	6	1.21971	1.37515	0.81938	1.13757
Q9D8E6	2.26	2.68	5.012	60S ribosomal protein L4	3	1.09634	1.44047	0.91159	1.32108
Q9D8N0	6.19	6.33	6.636	Elongation factor 1-gamma	5	1.14735	1.00062	0.87106	0.87731

Q9D8T2	2.75	3.03	2.053	Gasdermin-D	1	1.03185	0.80862	0.96856	0.79667
Q9D8U8	2.04	2.33	3.713	Sorting nexin-5	1	2.85399	3.78690	0.35018	1.33414
Q9D8V0	3.51	5.21	4.497	Minor histocompatibility antigen H13	4	1.36096	1.57276	0.73434	1.16180
Q9D8Y0	4.62	4.95	10.420	EF-hand domain-containing protein D2	3	0.74984	1.08767	1.33284	1.46181
Q9DAS9	5.38	5.38	41.670	Guanine nucleotide-binding protein G(I)/G(S)/G(O) subunit gamma-12	3	0.77338	0.71937	1.29226	0.93327
Q9DAX2	2.02	2.02	3.623	Lipid phosphate phosphohydrolase 2	1	1.08194	0.91146	0.92372	0.84703
Q9DB05	11.9	11.89	22.370	Alpha-soluble NSF attachment protein	8	1.19496	1.11659	0.83635	0.93884
Q9DB15	4.04	4.33	11.440	39S ribosomal protein L12, mitochondrial	2	1.61305	1.20745	0.61958	0.75265
Q9DB20	11.2	11.35	24.880	ATP synthase subunit O, mitochondrial	8	1.23226	1.23153	0.81103	1.00932
Q9DB34	2.01	2.65	4.054	Charged multivesicular body protein 2a	1	1.10281	1.06774	0.90624	0.97350
Q9DB77	25.3	25.25	32.890	Cytochrome b-c1 complex subunit 2, mitochondrial	21	1.61645	1.05926	0.61827	0.65029
Q9DBG3	6.57	23.22	10.670	AP-2 complex subunit beta	11	1.02253	1.47586	0.97739	1.46830
Q9DBG5	1.47	1.52	1.831	Perilipin-3	1	1.05255	1.00033	0.94951	0.95376
Q9DBG6	12.9	12.98	12.040	Dolichyl-diphosphooligosaccharide--protein glycosyltransferase subunit 2	10	1.70281	1.16913	0.58692	0.69642
Q9DBG7	2.04	2.87	1.415	Signal recognition particle receptor subunit alpha	1	1.54834	3.00189	0.64547	1.94937
Q9DBG9	4.9	5.9	21.770	Tax1-binding protein 3	3	0.97103	1.62904	1.02923	1.69080
Q9DBH5	4.39	4.59	5.866	Vesicular integral-membrane protein VIP36	2	1.13196	0.91684	0.88290	0.81096
Q9DBJ1	6.17	7.28	16.540	Phosphoglycerate mutase 1	4	0.83716	1.08523	1.19382	1.30506
Q9DBJ3	1.62	2.06	2.140	Brain-specific angiogenesis inhibitor 1-associated protein 2-like protein 1	1	0.51788	0.59683	1.92980	1.15873
Q9DBS1	2	2.27	2.500	Transmembrane protein 43	1	1.32422	1.64629	0.75472	1.25002
Q9DBU0	2.8	3.49	2.310	Transmembrane 9 superfamily member 1	1	1.61110	0.48162	0.62033	0.32777
Q9DC16	2.54	2.64	4.828	Endoplasmic reticulum-Golgi intermediate compartment protein 1	1	0.97766	0.92128	1.02224	0.94470

Q9DC23	4.33	4.67	2.648	DnaJ homolog subfamily C member 10	3	1.33314	1.20962	0.74967	0.91294
Q9DC29	4.01	6.89	3.444	ATP-binding cassette sub-family B member 6, mitochondrial	3	0.97200	0.87219	1.02820	0.89853
Q9DC51	1.44	11.64	16.380	Guanine nucleotide-binding protein G(k) subunit alpha	7	0.89995	0.86255	1.11051	0.96268
Q9DCD0	3.2	3.5	2.692	6-phosphogluconate dehydrogenase, decarboxylating	2	0.72812	1.15555	1.37258	1.59977
Q9DCH4	7.35	7.47	11.910	Eukaryotic translation initiation factor 3 subunit F	4	0.88746	0.67022	1.12614	0.76025
Q9DCI3	1.96	2.08	4.255	MLN64 N-terminal domain homolog	1	1.12274	1.81009	0.89016	1.62103
Q9DCM0	3.38	3.71	11.420	Persulfide dioxygenase ETHE1, mitochondrial	3	0.61719	0.20763	1.61928	0.33208
Q9DCS3	4.42	4.86	6.166	Trans-2-enoyl-CoA reductase, mitochondrial	2	0.72885	0.48672	1.37122	0.67571
Q9DCS9	5.88	6.31	27.840	NADH dehydrogenase [ubiquinone] 1 beta subcomplex subunit 10	4	1.02695	0.68565	0.97319	0.66439
Q9DCT2	9.84	9.84	19.390	NADH dehydrogenase [ubiquinone] iron-sulfur protein 3, mitochondrial	6	1.19445	0.68726	0.83671	0.57797
Q9DCV7	34.8	46	48.800	Keratin, type II cytoskeletal 7	41	1.60260	1.45216	0.62362	0.90380
Q9DCW4	11	11.2	18.430	Electron transfer flavoprotein subunit beta	6	0.95177	0.46357	1.05005	0.50877
Q9DCX2	4.87	4.93	11.180	ATP synthase subunit d, mitochondrial	3	0.99902	0.72258	1.00039	0.72079
Q9DD20	4.51	4.56	7.787	Methyltransferase-like protein 7B	4	1.67034	0.71539	0.59832	0.43905
Q9EPB4	1.68	1.69	7.254	Apoptosis-associated speck-like protein containing a CARD	2	1.05884	0.78963	0.94388	0.74983
Q9EPK2	6.5	6.58	7.493	Protein XRP2	3	0.77448	0.90951	1.29042	1.17821
Q9EPS2	2.01	2.01	9.184	Peptide YY	1	0.85306	0.72348	1.17156	0.85274
Q9EQ06	3.97	4.39	5.369	Estradiol 17-beta-dehydrogenase 11	2	1.04816	0.55803	0.95349	0.54124
Q9EQ20	3.04	3.29	2.056	Methylmalonate-semialdehyde dehydrogenase [acylating], mitochondrial	1	0.95920	0.43778	1.04192	0.45775
Q9EQH2	2.16	2.92	1.398	Endoplasmic reticulum aminopeptidase 1	1	0.94485	0.71975	1.05774	0.76603
Q9EQI8	1.77	2.13	7.067	39S ribosomal protein L46, mitochondrial	2	1.05529	0.92939	0.94705	0.88551

Q9EQK5	11.8	15.23	9.756	Major vault protein	8	1.06679	0.91751	0.93684	0.86407
Q9ER00	2	2.33	5.474	Syntaxin-12	1	1.25897	0.66613	0.79383	0.53200
Q9ERE2	7.07	10.39	12.890	Keratin, type II cuticular Hb1	11	2.77484	1.10175	0.36017	0.39012
Q9ERG0	14.4	14.91	9.296	LIM domain and actin-binding protein 1	7	0.92935	1.03980	1.07539	1.11509
Q9ERI2	2.24	4.75	13.570	Ras-related protein Rab-27A	4	0.83463	0.62797	1.19743	0.75701
Q9ERS2	6	6.08	21.530	NADH dehydrogenase [ubiquinone] 1 alpha subcomplex subunit 13	4	1.15678	0.82093	0.86396	0.71184
Q9ES64	2.01	2.58	1.538	Harmonin	1	0.53289	0.27666	1.87545	0.52201
Q9ES97	2.69	3.57	1.141	Reticulon-3	1	2.53819	3.02582	0.39375	1.19863
Q9ESP1	3.96	4.45	9.502	Stromal cell-derived factor 2-like protein 1	2	1.28549	1.11210	0.77746	0.87213
Q9ET30	2.07	4.44	2.385	Transmembrane 9 superfamily member 3	3	1.29487	0.92592	0.77182	0.71996
Q9JHC0	4.78	4.84	16.320	Glutathione peroxidase 2	3	0.87407	1.03641	1.14339	1.19055
Q9JHJ0	7.7	8.66	13.070	Tropomodulin-3	5	1.00353	1.04800	0.99590	1.05205
Q9JHK0	1.7	2.26	2.632	Prolactin-2A1	1	1.15180	1.95003	0.86769	1.70228
Q9JHS3	1.6	1.62	7.200	Ragulator complex protein LAMTOR2	1	0.92987	1.65294	1.07478	1.78733
Q9JHU4	3.47	13.6	0.732	Cytoplasmic dynein 1 heavy chain 1	4	0.88242	1.13087	1.13258	1.28652
Q9JI67	6.74	10.24	10.390	Beta-1,3-galactosyltransferase 5	5	0.76580	0.44101	1.30505	0.58653
Q9JIF7	9.9	11.57	5.352	Coatomer subunit beta	5	1.03380	0.83431	0.96673	0.81554
Q9JII6	5.29	5.68	8.308	Alcohol dehydrogenase [NADP(+)]	3	0.78042	0.85257	1.28060	1.09889
Q9JIP7	1.57	1.94	0.987	Solute carrier family 15 member 1	1	0.38355	0.31431	2.60566	0.82394
Q9JIW9	2.08	2.14	5.340	Ras-related protein Ral-B	1	1.11505	1.32449	0.89629	1.19606
Q9JIY5	4	4.07	4.803	Serine protease HTRA2, mitochondrial	2	1.29725	0.52811	0.77041	0.39993
Q9JJ00	2.68	3.13	3.049	Phospholipid scramblase 1	1	0.76329	0.77514	1.30935	1.01852
Q9JJ06	2.88	3.18	6.612	Glycoprotein-N-acetylgalactosamine 3-beta-galactosyltransferase 1	3	1.01951	0.79051	0.98028	0.77204
Q9JJI8	2.15	2.24	28.570	60S ribosomal protein L38	2	0.98220	1.37394	1.01752	1.40583

Q9JJX6	1.92	3.42	3.351	P2X purinoceptor 4	2	0.78754	0.84603	1.26902	1.07848
Q9JJY3	2.64	3.37	2.901	Sphingomyelin phosphodiesterase 3	2	1.03176	1.06718	0.96865	1.03802
Q9JJZ4	2	2.04	2.201	Ubiquitin-conjugating enzyme E2 J1	1	1.49217	1.17057	0.66977	0.78876
Q9JKA5	12	12.52	22.880	Cell surface A33 antigen	11	0.91575	0.75579	1.09136	0.83118
Q9JKF1	8.14	15.75	4.768	Ras GTPase-activating-like protein IQGAP1	8	0.83989	1.34731	1.18992	1.61296
Q9JKR6	18.4	20.51	6.406	Hypoxia up-regulated protein 1	8	0.96947	0.74006	1.03088	0.76519
Q9JL27	1.97	2.32	2.305	Galactoside 2-alpha-L-fucosyltransferase 2	2	1.24256	0.68473	0.80432	0.55489
Q9JLB0	1.5	2.63	2.170	MAGUK p55 subfamily member 6	1	0.88436	0.87135	1.13009	0.99068
Q9JLJ0	1.4	1.4	4.348	Lipopolysaccharide-induced tumor necrosis factor-alpha factor homolog	1	0.89729	1.05774	1.11381	1.18526
Q9JLQ0	6.05	6.49	4.867	CD2-associated protein	3	0.86935	0.95936	1.14960	1.10801
Q9JLZ6	2	2.42	1.616	Hypermethylated in cancer 2 protein	2	1.11806	3.74669	0.89388	3.36940
Q9JM76	6.02	6.42	13.480	Actin-related protein 2/3 complex subunit 3	2	0.86067	1.01722	1.16120	1.19080
Q9JM95	4.81	4.83	8.709	Alpha-N-acetylgalactosaminide alpha-2,6-sialyltransferase 6	3	1.39528	1.01674	0.71628	0.73013
Q9JMA9	2.14	2.64	1.097	Sodium- and chloride-dependent neutral and basic amino acid transporter B(0+)	1	0.49235	0.37744	2.02989	0.76687
Q9JMG2	2.74	3.26	3.165	C1GALT1-specific chaperone 1	1	1.43312	1.14280	0.69737	0.80241
Q9JMH9	4.33	7.17	1.366	Unconventional myosin-XVIIIa	3	1.46450	1.19483	0.68242	0.82172
Q9NYQ2	15.1	16.96	24.930	Hydroxyacid oxidase 2	9	1.30381	0.60060	0.76653	0.50294
Q9QUI0	6.1	6.22	18.650	Transforming protein RhoA	3	0.63835	0.94893	1.56561	1.49242
Q9QWG7	2.02	2.67	5.017	Sulfotransferase family cytosolic 1B member 1	2	0.79886	0.39994	1.25105	0.50291
Q9QXH4	1.55	2.05	0.684	Integrin alpha-X	2	1.39444	4.16388	0.71671	3.00238
Q9QXS1	83.7	83.78	7.248	Plectin	35	1.28268	1.09342	0.77916	0.85305
Q9QXT0	1.41	1.43	8.791	Protein canopy homolog 2	1	1.59387	0.67833	0.62703	0.42791
Q9QXX4	4.46	6.26	5.621	Calcium-binding mitochondrial carrier protein Aralar2	4	1.10602	0.95330	0.90361	0.86262

Q9QY23	2.08	2.17	1.004	Plakophilin-3	1	1.24393	1.40895	0.80343	1.14501
Q9QY76	2.03	2.13	3.292	Vesicle-associated membrane protein-associated protein B	1	1.08466	1.04974	0.92140	0.95743
Q9QYA2	2	2.04	3.047	Mitochondrial import receptor subunit TOM40 homolog	1	1.07660	1.08555	0.92830	1.01382
Q9QYB1	2	2.86	4.348	Chloride intracellular channel protein 4	1	0.71091	1.39446	1.40582	1.97225
Q9QYJ0	2.17	3.53	4.612	DnaJ homolog subfamily A member 2	2	1.04890	1.11461	0.95282	1.06845
Q9QZD9	2.07	2.23	3.077	Eukaryotic translation initiation factor 3 subunit I	1	0.90301	0.79474	1.10676	0.88492
Q9QZE5	2.06	2.57	1.030	Coatomer subunit gamma-1	1	1.12929	1.02237	0.88499	0.91096
Q9QZQ1	2.11	4.11	0.934	Afadin	2	0.98695	1.01455	1.01263	1.04291
Q9R092	2	2.12	2.839	17-beta-hydroxysteroid dehydrogenase type 6	1	1.55129	2.54708	0.64424	1.65089
Q9R0A1	3.77	4.08	2.753	Chloride channel protein 2	2	1.07624	0.55857	0.92861	0.52064
Q9R0H0	4.26	4.32	4.841	Peroxisomal acyl-coenzyme A oxidase 1	3	1.59490	0.95757	0.62663	0.68247
Q9R0K7	2.12	3.65	3.005	Plasma membrane calcium-transporting ATPase 2	3	1.79899	0.66995	0.55554	0.37444
Q9R0P3	2.02	2.45	4.255	S-formylglutathione hydrolase	1	1.08358	1.15026	0.92232	1.06734
Q9R0P5	18.1	18.3	48.480	Destrin	11	0.65571	0.65092	1.52416	0.99311
Q9R0Q3	1.47	1.47	4.478	Transmembrane emp24 domain-containing protein 2	1	1.37712	0.76175	0.72572	0.55409
Q9R100	27.6	28.57	18.620	Cadherin-17	17	0.89986	0.64865	1.11063	0.72278
Q9R112	10.1	10.72	12.670	Sulfide:quinone oxidoreductase, mitochondrial	6	1.34812	0.88052	0.74134	0.65481
Q9R1J0	2.27	2.43	3.591	Sterol-4-alpha-carboxylate 3-dehydrogenase, decarboxylating	1	1.78670	1.21006	0.55936	0.67506
Q9R1P1	2.04	2.3	4.390	Proteasome subunit beta type-3	1	0.83647	0.94003	1.19480	1.13002
Q9R1P4	2.33	2.46	3.422	Proteasome subunit alpha type-1	1	0.96082	1.23943	1.04016	1.29510
Q9R1Q7	2.53	2.53	7.895	Proteolipid protein 2	1	1.01218	0.93196	0.98738	0.92668
Q9R233	4.14	4.96	4.516	Tapasin	2	1.62174	1.70445	0.61626	1.05558
Q9WTL2	6	6.23	14.550	Ras-related protein Rab-25	3	1.13348	0.88046	0.88172	0.77871
Q9WTL7	1.34	2.52	5.628	Acyl-protein thioesterase 2	2	1.20015	1.22388	0.83274	1.02535

Q9WTM5	2.41	2.52	2.592	RuvB-like 2	1	0.89339	0.97843	1.11867	1.10168
Q9WTP6	5.26	6.64	11.300	Adenylate kinase 2, mitochondrial	3	0.87746	0.68105	1.13898	0.78157
Q9WTP7	10.6	10.91	36.120	GTP:AMP phosphotransferase AK3, mitochondrial	8	0.89779	0.44857	1.11319	0.50682
Q9WTS2	2.04	2.33	1.391	Alpha-(1,6)-fucosyltransferase	1	1.54400	1.04021	0.64729	0.67739
Q9WTX5	1.92	2.24	4.908	S-phase kinase-associated protein 1	1	0.81090	1.02387	1.23247	1.26954
Q9WU01	2.15	2.33	4.298	KH domain-containing, RNA-binding, signal transduction-associated protein 2	2	0.92050	1.04673	1.08573	1.14940
Q9WU78	14	17.43	9.321	Programmed cell death 6-interacting protein	10	0.93547	1.04926	1.06834	1.12240
Q9WUA3	4.42	5.24	2.551	6-phosphofructokinase type C	2	1.01051	0.94520	0.98902	0.94048
Q9WUM4	2.05	5.02	3.165	Coronin-1C	2	0.64114	1.07018	1.55881	1.67832
Q9WUM5	2.16	3.21	3.179	Succinyl-CoA ligase [ADP/GDP-forming] subunit alpha, mitochondrial	1	0.87302	0.57052	1.14477	0.65707
Q9WUR2	4.05	4.31	5.371	Enoyl-CoA delta isomerase 2, mitochondrial	2	0.83215	0.59424	1.20100	0.71303
Q9WUZ9	4.68	5.31	4.215	Ectonucleoside triphosphate diphosphohydrolase 5	2	1.04943	0.69134	0.95233	0.69658
Q9WV02	4.22	4.23	6.905	RNA-binding motif protein, X chromosome	2	1.10556	1.21439	0.90398	1.10040
Q9WV54	7.05	7.18	9.645	Acid ceramidase	4	1.04375	1.11789	0.95752	1.07935
Q9WV55	2.14	2.14	3.213	Vesicle-associated membrane protein-associated protein A	1	1.61430	1.01729	0.61910	0.63164
Q9WV80	3.33	5.65	6.130	Sorting nexin-1	4	0.98006	0.87865	1.01974	0.90591
Q9WV91	2.17	2.76	1.138	Prostaglandin F2 receptor negative regulator	1	0.86067	1.18853	1.16120	1.39296
Q9WV92	5.31	6.07	2.799	Band 4.1-like protein 3	3	0.76413	0.62484	1.30791	0.84234
Q9WVA4	11	11.07	37.690	Transgelin-2	8	0.68065	1.08210	1.46832	1.61967
Q9WVC8	12.9	13.11	10.830	Chloride anion exchanger	8	0.35372	0.24415	2.82545	0.63414
Q9WVP1	2.08	2.31	1.891	AP-1 complex subunit mu-2	1	0.72668	0.97606	1.37531	1.35052
Q9Z0F4	2	2.04	4.712	Calcium and integrin-binding protein 1	1	0.56772	0.60069	1.76038	1.06386
Q9Z0G9	2.94	2.94	5.479	Claudin-3	3	0.85898	0.44563	1.16349	0.53363

Q9Z0L8	2.22	2.35	3.470	Gamma-glutamyl hydrolase	1	0.86433	0.68151	1.15628	0.79144
Q9Z0S1	1.85	1.89	3.896	3'(2'),5'-bisphosphate nucleotidase 1	1	0.82236	0.75691	1.21529	0.92545
Q9Z0U1	16.2	17.59	6.427	Tight junction protein ZO-2	7	1.09455	1.25247	0.91308	1.15031
Q9Z0X1	5.94	6.46	4.575	Apoptosis-inducing factor 1, mitochondrial	3	1.14110	0.96687	0.87583	0.85705
Q9Z110	3.17	4.74	2.516	Delta-1-pyrroline-5-carboxylate synthase	3	0.93964	0.73480	1.06361	0.78743
Q9Z199	2.09	2.17	6.838	Transcription elongation factor SPT4-B	1	1.39198	2.11009	0.71798	1.52418
Q9Z1G3	3.69	3.91	4.712	V-type proton ATPase subunit C 1	2	0.68493	1.02102	1.45913	1.47805
Q9Z1G4	2.37	2.66	1.073	V-type proton ATPase 116 kDa subunit a isoform 1	1	0.97761	0.92085	1.02230	0.94716
Q9Z1N5	6.75	7.05	8.411	Spliceosome RNA helicase Ddx39b	5	1.27342	1.44209	0.78482	1.13194
Q9Z1P6	6.96	7.09	37.170	NADH dehydrogenase [ubiquinone] 1 alpha subcomplex subunit 7	4	1.18143	1.03314	0.84593	0.89088
Q9Z1Q5	4.26	4.85	12.450	Chloride intracellular channel protein 1	3	0.81102	1.05602	1.23229	1.30967
Q9Z1W8	19.1	32.8	15.750	Potassium-transporting ATPase alpha chain 2	21	0.42693	0.45321	2.34090	1.06149
Q9Z204	6.62	6.68	13.420	Heterogeneous nuclear ribonucleoproteins C1/C2	4	1.17458	1.06966	0.85087	0.91251
Q9Z261	2	2	7.583	Claudin-7	2	1.01674	0.57613	0.98295	0.56974
Q9Z2I0	15.2	16.81	10.300	LETM1 and EF-hand domain-containing protein 1, mitochondrial	8	1.04093	0.66456	0.96011	0.64683
Q9Z2I8	15.8	16.74	17.320	Succinyl-CoA ligase [GDP-forming] subunit beta, mitochondrial	9	0.92281	0.58459	1.08301	0.63633
Q9Z2I9	7.88	10.88	12.100	Succinyl-CoA ligase [ADP-forming] subunit beta, mitochondrial	7	1.02941	0.73627	0.97086	0.71859
Q9Z2M6	1.5	1.55	5.983	Ubiquitin-like protein 3	1	0.74182	1.04917	1.34725	1.41905
Q9Z2M7	8.67	8.75	19.010	Phosphomannomutase 2	4	0.93493	0.85198	1.06897	0.91600
Q9Z2Q6	4	4.07	5.149	Septin-5	2	1.04861	1.52963	0.95308	1.46702
Q9Z2U0	8.11	9.88	23.390	Proteasome subunit alpha type-7	7	0.91900	1.03946	1.08750	1.13854
Q9Z2U1	2.85	3.55	6.224	Proteasome subunit alpha type-5	2	0.69451	0.83711	1.43902	1.21243

Q9Z2W0	2.03	2.32	2.114	Aspartyl aminopeptidase	1	1.00080	1.31956	0.99861	1.32571
Q9Z2X1	3.96	4.44	5.301	Heterogeneous nuclear ribonucleoprotein F	2	0.83515	0.79416	1.19669	0.95612
RRRRRA2AAJ9	1.49	10.42	0.540	REVERSED Obscurin	4	0.78399	0.45526	1.27477	0.58387
RRRRRQ8BSP2	1.9	1.96	0.824	REVERSED Condensin-2 complex subunit H2	1	0.87023	0.91280	1.14844	1.05465
RRRRRQ8CGN5	1.31	1.4	1.161	REVERSED Perilipin-1	1	1.38956	1.31861	0.71923	0.95413
RRRRRQ9D9V7	2.05	5.5	2.906	REVERSED Protein DENND6B	4	0.64787	0.40248	1.54261	0.62464

Table S6. The proteins identified and quantified in the experiment replicate 3

Accession	Unused	Total	%Cov(95)	Name	Peptides (95%)	116:114	117:114	114:116	117:116
A2A863	20.23	21.18	6.216	Integrin beta-4	11	1.11716	1.68985	0.89445	1.51522
A2AQ25	2.09	6.14	0.771	Sickle tail protein	2	1.25741	1.12636	0.79468	0.89906
B1AXP6	2.72	2.72	15.690	Mitochondrial import receptor subunit TOM5 homolog	1	0.96624	1.21683	1.03416	1.24989
B2RX12	15.71	16.84	3.677	Canalicular multispecific organic anion transporter 2	6	0.93983	0.59277	1.06322	0.63938
B2RXS4	31.66	33.26	9.229	Plexin-B2	18	0.92235	0.82554	1.08337	0.88871
D3Z6P0	2.84	3.82	2.087	Protein disulfide-isomerase A2	1	1.60920	0.68198	0.62096	0.42133
E9Q557	10.14	19.85	1.457	Desmoplakin	5	1.24815	1.18536	0.80058	0.94510
F7BWT7	2.33	2.38	6.803	Tetraspanin-15	2	0.91375	0.97546	1.09357	1.07279
G5E8K5	4.25	7.5	1.173	Ankyrin-3	3	0.87332	0.88098	1.14419	1.01244
O08528	8.52	9.57	4.689	Hexokinase-2	5	0.99089	0.96983	1.00843	0.98315
O08553	4.32	5.41	4.545	Dihydropyrimidinase-related protein 2	2	0.91867	1.15038	1.08771	1.25720
O08585	2.72	3.03	2.979	Clathrin light chain A	1	0.93538	1.06186	1.06827	1.13752
O08600	2.02	2.07	3.401	Endonuclease G, mitochondrial	1	0.83793	0.35075	1.19251	0.42012
O08638	21.65	40.58	7.150	Myosin-11	18	1.26397	1.57998	0.79056	1.22438
O08663	2.69	2.9	1.883	Methionine aminopeptidase 2	1	1.13376	1.17531	0.88135	1.03950

O08709	14.39	17.25	32.140	Peroxiredoxin-6	8	0.63950	0.47702	1.56254	0.74990
O08749	7.41	7.78	7.859	Dihydrolipoyl dehydrogenase, mitochondrial	5	0.72563	0.46477	1.37707	0.64438
O08756	6.26	6.27	11.490	3-hydroxyacyl-CoA dehydrogenase type-2	4	0.90578	0.64735	1.10318	0.72618
O08795	2.1	2.24	2.303	Glucosidase 2 subunit beta	1	1.87908	1.22425	0.53177	0.65702
O08917	3.77	4.14	5.140	Flotillin-1	2	1.30166	1.70049	0.76767	1.31088
O08997	2	2	11.760	Copper transport protein ATOX1	1	1.08374	1.29739	0.92203	1.20153
O09061	2.22	2.27	3.750	Proteasome subunit beta type-1	1	1.17879	0.68224	0.84768	0.58088
O09111	2	2	6.623	NADH dehydrogenase [ubiquinone] 1 beta subcomplex subunit 11, mitochondrial	1	1.50496	1.40023	0.66396	0.93381
O09117	3.82	4.18	5.747	Synaptophysin-like protein 1	2	1.28168	1.36720	0.77964	1.06599
O09131	4.68	5.33	13.330	Glutathione S-transferase omega-1	3	0.74118	0.60928	1.34818	0.82224
O09159	1.31	2.15	0.790	Lysosomal alpha-mannosidase	1	1.09372	1.01996	0.91362	0.93597
O35054	1.96	2	5.714	Claudin-4	2	1.09086	1.19027	0.91602	1.09513
O35114	2.4	2.7	1.674	Lysosome membrane protein 2	1	0.96703	1.33095	1.03332	1.37554
O35126	1.4	1.4	0.596	Atrophin-1	1	1.30225	0.41936	0.76732	0.32321
O35129	13	13.15	21.400	Prohibitin-2	8	1.26276	1.31898	0.79131	1.04589
O35143	2.18	2.19	19.810	ATPase inhibitor, mitochondrial	2	1.47338	0.52123	0.67820	0.35506
O35381	2.02	2.13	3.239	Acidic leucine-rich nuclear phosphoprotein 32 family member A	1	0.73183	1.44482	1.36540	1.96413
O35566	1.63	1.94	1.976	CD151 antigen	1	1.06822	1.22410	0.93543	1.15287
O35598	4.14	4.57	2.937	Disintegrin and metalloproteinase domain-containing protein 10	2	1.05727	1.48965	0.94512	1.41215
O35609	2.07	2.11	4.585	Secretory carrier-associated membrane protein 3	1	0.95533	1.23877	1.04597	1.29270
O35639	14.72	17.78	28.480	Annexin A3	9	1.09540	1.30917	0.91222	1.19690
O35640	4.41	5.31	12.840	Annexin A8	5	1.35637	2.47914	0.73670	1.90225
O35643	22.4	25.35	11.980	AP-1 complex subunit beta-1	13	0.85223	1.00355	1.17250	1.18206

O35744	3.07	3.86	3.518	Chitinase-3-like protein 3	2	0.51148	1.35565	1.95363	2.66016
O35857	2.02	2.4	1.770	Mitochondrial import inner membrane translocase subunit TIM44	1	0.76946	0.68412	1.29863	0.89234
O35887	2.92	3.08	5.714	Calumenin	3	1.14998	1.08245	0.86892	0.94257
O35900	1.72	2.12	7.368	U6 snRNA-associated Sm-like protein LSm2	1	0.65630	0.69620	1.52254	1.06469
O54734	9.25	9.49	11.110	Dolichyl-diphosphooligosaccharide--protein glycosyltransferase 48 kDa subunit	7	1.27577	0.94169	0.78324	0.74541
O54950	3.04	3.17	6.364	5'-AMP-activated protein kinase subunit gamma-1	2	0.89137	0.74789	1.12102	0.84231
O55013	1.87	1.88	4.444	Trafficking protein particle complex subunit 3	1	1.10827	1.17419	0.90162	1.06336
O55022	8.95	9.02	15.900	Membrane-associated progesterone receptor component 1	4	1.33478	0.72402	0.74862	0.54442
O55111	12.22	13.39	5.971	Desmoglein-2	6	1.26591	1.20248	0.78935	0.94826
O55125	1.59	1.75	2.113	Protein NipSnap homolog 1	1	1.25113	0.91632	0.79867	0.73665
O55131	2.86	3.3	5.275	Septin-7	3	1.30550	1.76391	0.76541	1.35608
O55143	9.93	17.64	7.567	Sarcoplasmic/endoplasmic reticulum calcium ATPase 2	8	0.95118	1.21054	1.05053	1.27538
O55222	2.02	2.45	2.212	Integrin-linked protein kinase	1	2.64711	5.79891	0.37749	2.19869
O70251	9.08	9.2	19.560	Elongation factor 1-beta	7	1.05771	0.83474	0.94473	0.78969
O70274	5.35	5.95	13.170	Protein tyrosine phosphatase type IVA 2	2	0.87354	0.92557	1.14390	1.06491
O70400	15.44	16.27	22.630	PDZ and LIM domain protein 1	8	0.73039	0.85756	1.36809	1.17997
O70435	5.89	6.04	10.980	Proteasome subunit alpha type-3	3	1.26532	1.16886	0.78972	0.92392
O70439	6.95	6.95	14.940	Syntaxin-7	4	0.86396	0.85746	1.15658	0.98676
O70456	1.95	8.3	14.920	14-3-3 protein sigma	6	0.81972	0.86480	1.21900	1.06090
O70475	18.49	19.06	21.300	UDP-glucose 6-dehydrogenase	11	0.83613	0.48724	1.19508	0.59142
O70492	3.94	4.37	14.200	Sorting nexin-3	2	0.99503	1.12651	1.00424	1.13779
O70493	2.1	2.15	5.455	Sorting nexin-12	1	0.98222	1.40884	1.01733	1.43960
O70570	10.77	11.06	8.301	Polymeric immunoglobulin receptor	6	1.11301	0.88402	0.89779	0.79790
O88312	15.79	15.89	33.140	Anterior gradient protein 2 homolog	14	1.17196	0.18743	0.85263	0.17872

O88329	9.54	11.65	5.561	Unconventional myosin-Ia	6	0.63435	0.75423	1.57523	1.19255
O88342	3.39	3.73	3.135	WD repeat-containing protein 1	2	0.75203	1.20177	1.32872	1.60856
O88343	2.05	2.65	0.649	Electrogenic sodium bicarbonate cotransporter 1	1	0.86956	0.53703	1.14914	0.61986
O88384	2.92	3.02	9.914	Vesicle transport through interaction with t-SNAREs homolog 1B	2	1.25134	1.75111	0.79854	1.40451
O88456	2.01	2.01	4.833	Calpain small subunit 1	1	1.11224	1.35914	0.89841	1.22783
O88487	4	4.24	2.778	Cytoplasmic dynein 1 intermediate chain 2	2	0.87122	0.93970	1.14695	1.07944
O88569	9.12	9.12	15.860	Heterogeneous nuclear ribonucleoproteins A2/B1	5	0.66518	0.35408	1.50221	0.53425
O88643	2.02	3.1	3.119	Serine/threonine-protein kinase PAK 1	2	0.95981	1.24749	1.04109	1.30449
O88685	3.34	4.17	1.584	26S protease regulatory subunit 6A	1	1.06517	0.75535	0.93811	0.73278
O88712	2.09	2.24	2.268	C-terminal-binding protein 1	1	1.11215	0.87424	0.89848	0.78896
O88844	13.3	13.44	16.430	Isocitrate dehydrogenase [NADP] cytoplasmic	7	0.97500	1.22293	1.02487	1.25719
O88952	7.06	8.5	20.300	Protein lin-7 homolog C	6	1.01383	1.27111	0.98561	1.26634
O88983	2.19	3.03	3.390	Syntaxin-8	1	0.86257	1.40247	1.15845	1.63188
O89001	16.27	17.61	5.301	Carboxypeptidase D	8	0.85512	0.65665	1.16855	0.77028
O89053	6.66	6.92	7.375	Coronin-1A	3	1.37554	2.76942	0.72644	2.19963
O89079	5.02	7.15	13.310	Coatomer subunit epsilon	3	0.96600	0.86016	1.03441	0.89369
P00329	5.07	5.44	7.467	Alcohol dehydrogenase 1	4	0.74207	0.63510	1.34655	0.86114
P00397	2	2	1.556	Cytochrome c oxidase subunit 1	1	1.10380	1.19604	0.90527	1.08754
P00405	3.56	6.28	10.570	Cytochrome c oxidase subunit 2	3	1.05867	0.62813	0.94387	0.59236
P00920	4.32	5.81	6.923	Carbonic anhydrase 2	2	0.39265	0.38895	2.54486	0.99421
P01027	2.03	5.12	0.962	Complement C3	2	1.31313	2.30392	0.76096	1.76096
P01592	2.81	2.81	6.918	Immunoglobulin J chain	1	1.27838	0.97930	0.78165	0.76725
P01837	5.37	5.37	25.470	Ig kappa chain C region	3	1.39771	1.20920	0.71491	0.86823
P01878	2.05	2.28	4.360	Ig alpha chain C region	1	1.31315	1.01249	0.76095	0.77058
P01899	10.59	10.69	14.920	H-2 class I histocompatibility antigen, D-B alpha chain	8	0.81559	0.95370	1.22518	1.13997

P01901	2.72	10.57	11.380	H-2 class I histocompatibility antigen, K-B alpha chain	7	0.91525	0.97152	1.09177	1.06565
P01902	2.04	4.86	5.435	H-2 class I histocompatibility antigen, K-D alpha chain	3	0.71411	0.24428	1.39928	0.34333
P01942	9.4	9.6	42.250	Hemoglobin subunit alpha	10	0.72525	0.49855	1.37779	0.72382
P02088	13.17	13.17	57.140	Hemoglobin subunit beta-1	23	0.69553	0.44072	1.43666	0.63940
P02301	2.44	2.88	10.290	Histone H3.3C	2	0.89729	1.84983	1.11362	2.06967
P02535	3.15	5.75	8.070	Keratin, type I cytoskeletal 10	8	0.43654	0.74229	2.28901	1.70113
P03930	3	3.01	28.360	ATP synthase protein 8	3	1.12687	1.12745	0.88674	1.00452
P04441	2.93	2.93	8.602	H-2 class II histocompatibility antigen gamma chain	2	1.60095	1.49203	0.62416	0.93538
P04919	2	5.23	1.615	Band 3 anion transport protein	3	1.05489	1.74455	0.94725	1.65983
P05064	24.23	24.72	33.790	Fructose-bisphosphate aldolase A	16	0.92760	0.90796	1.07724	0.98804
P05202	11.83	11.92	17.440	Aspartate aminotransferase, mitochondrial	8	1.20256	0.89396	0.83093	0.74784
P05555	1.72	2.38	0.694	Integrin alpha-M	2	1.28872	3.43003	0.77538	2.68475
P05784	28.7	34.48	35.460	Keratin, type I cytoskeletal 18	41	1.44736	0.97059	0.69039	0.67483
P06151	15.43	17.62	23.490	L-lactate dehydrogenase A chain	15	0.70455	0.74743	1.41827	1.03909
P06745	3.08	4.31	2.867	Glucose-6-phosphate isomerase	2	0.66224	1.12167	1.50888	1.70332
P06800	3.14	3.97	0.852	Receptor-type tyrosine-protein phosphatase C	1	1.07169	2.97911	0.93240	2.78967
P07309	3.18	3.42	10.880	Transthyretin	2	0.93355	1.19264	1.07037	1.28134
P07356	40.84	42.64	54.570	Annexin A2	45	1.11384	2.29422	0.89712	2.02954
P07724	24.98	25.76	17.110	Serum albumin	13	1.03187	1.20922	0.96838	1.15752
P07758	5.94	7.25	7.022	Alpha-1-antitrypsin 1-1	4	1.12979	1.14269	0.88445	1.01152
P07901	6.33	20.25	12.280	Heat shock protein HSP 90-alpha	11	1.03788	1.22109	0.96277	1.18269
P08003	21.78	22.7	19.120	Protein disulfide-isomerase A4	14	1.18791	1.10450	0.84117	0.93969
P08071	5.49	5.85	4.526	Lactotransferrin	3	1.57235	3.06981	0.63551	2.13702
P08113	24.58	32.3	19.580	Endoplasmin	18	1.45803	1.03122	0.68534	0.71178
P08207	9.29	9.29	34.020	Protein S100-A10	7	0.98489	1.38790	1.01458	1.40352
P08228	2	2.08	7.143	Superoxide dismutase [Cu-Zn]	1	1.58262	0.69309	0.63139	0.43955

P08249	28.3	29.25	54.730	Malate dehydrogenase, mitochondrial	23	1.10808	0.49893	0.90178	0.46911
P08752	12.01	13.78	19.440	Guanine nucleotide-binding protein G(i) subunit alpha-2	11	0.89349	0.97059	1.11836	1.09335
P09055	22.56	24.01	13.780	Integrin beta-1	15	0.99872	1.34291	1.00053	1.34227
P09103	41	41.2	35.760	Protein disulfide-isomerase	24	1.26380	0.89519	0.79067	0.71614
P09405	14.6	15.4	11.880	Nucleolin	9	1.27235	1.29904	0.78535	1.03383
P09411	12.57	15.44	13.190	Phosphoglycerate kinase 1	7	0.83178	0.90091	1.20133	1.09031
P09671	6.18	6.44	13.510	Superoxide dismutase [Mn], mitochondrial	4	0.88009	0.63097	1.13539	0.72342
P09803	7.07	7.89	3.959	Cadherin-1	3	1.11067	1.33244	0.89968	1.24787
P10107	29.13	31.58	42.200	Annexin A1	21	1.49610	4.59508	0.66790	3.38190
P10126	15.32	17.8	22.290	Elongation factor 1-alpha 1	14	1.11084	1.60541	0.89954	1.44445
P10404	4.04	4.75	3.276	MLV-related proviral Env polyprotein	2	0.98887	2.59704	1.01049	2.63906
P10639	6.17	6.63	29.520	Thioredoxin	7	0.78673	0.69520	1.27013	0.88617
P10649	9.82	9.93	22.480	Glutathione S-transferase Mu 1	5	0.92820	0.72992	1.07654	0.79326
P10852	4.51	4.74	3.802	4F2 cell-surface antigen heavy chain	2	1.05878	1.47429	0.94377	1.39393
P11352	4.49	4.66	13.430	Glutathione peroxidase 1	3	0.90880	0.94482	1.09951	1.04125
P11438	8.05	8.09	11.820	Lysosome-associated membrane glycoprotein 1	6	0.88438	1.25188	1.12988	1.39660
P11499	31.24	31.81	18.920	Heat shock protein HSP 90-beta	19	1.13926	1.57341	0.87710	1.39448
P11672	1.71	2.24	3.500	Neutrophil gelatinase-associated lipocalin	1	1.41294	3.04374	0.70721	2.23151
P11679	71.46	71.47	63.470	Keratin, type II cytoskeletal 8	119	1.78690	0.44697	0.55921	0.26965
P11688	2.06	2.49	1.330	Integrin alpha-5	1	1.38406	1.57056	0.72197	1.12290
P11835	2.63	3.23	1.297	Integrin beta-2	1	1.22607	2.33756	0.81500	1.91170
P11983	13.56	16.32	15.110	T-complex protein 1 subunit alpha	7	0.97622	1.20785	1.02358	1.24280
P12367	4.12	4.43	4.239	cAMP-dependent protein kinase type II-alpha regulatory subunit	2	0.91539	0.72355	1.09160	0.79328
P12787	15.3	15.67	53.420	Cytochrome c oxidase subunit 5A, mitochondrial	13	1.38294	0.83635	0.72255	0.60455
P13020	4.73	7.44	4.615	Gelsolin	4	0.76234	0.95170	1.31077	1.25650

P13634	17.91	18.28	37.160	Carbonic anhydrase 1	20	0.65301	0.19585	1.53022	0.29291
P13808	9.04	10.92	4.042	Anion exchange protein 2	5	0.91955	0.55158	1.08667	0.59934
P14069	5.49	5.55	16.850	Protein S100-A6	3	0.87246	1.09452	1.14532	1.25889
P14094	12.9	14.32	21.380	Sodium/potassium-transporting ATPase subunit beta-1	19	0.72106	0.50189	1.38579	0.69308
P14131	2.28	2.66	6.849	40S ribosomal protein S16	1	0.73085	1.28412	1.36723	1.76104
P14152	6.4	7.3	8.084	Malate dehydrogenase, cytoplasmic	4	0.74185	0.78960	1.34696	1.06881
P14206	10.5	11.12	22.030	40S ribosomal protein SA	8	1.14832	1.09382	0.87018	1.05395
P14211	16.69	17.09	17.790	Calreticulin	12	1.53847	1.23723	0.64950	0.80699
P14733	4.84	5.89	3.912	Lamin-B1	2	1.08317	1.40764	0.92252	1.30473
P14824	45.51	46.63	25.850	Annexin A6	24	1.03376	3.05878	0.96661	3.02419
P14869	22.47	22.69	39.430	60S acidic ribosomal protein P0	13	1.22993	1.20259	0.81244	0.98241
P15105	1.63	1.65	2.145	Glutamine synthetase	1	1.07380	1.60167	0.93057	1.49706
P15379	5.66	5.79	4.370	CD44 antigen	3	0.90858	1.60750	1.09978	1.77668
P15532	8.38	9.6	36.180	Nucleoside diphosphate kinase A	7	0.89571	0.99752	1.11559	1.14044
P15535	2	2.11	2.506	Beta-1,4-galactosyltransferase 1	1	1.30352	1.33147	0.76657	1.02518
P16045	2.27	2.36	5.926	Galectin-1	1	0.99738	4.21940	1.00187	4.28799
P16110	15.39	15.39	29.170	Galectin-3	17	0.80996	0.86565	1.23369	0.96882
P16546	72.98	73.49	16.550	Spectrin alpha chain, non-erythrocytic 1	44	1.11264	1.15337	0.89809	1.04055
P16858	18.66	20.09	25.830	Glyceraldehyde-3-phosphate dehydrogenase	12	0.95336	1.42928	1.04813	1.48721
P17047	4.77	4.82	4.096	Lysosome-associated membrane glycoprotein 2	3	1.54032	4.65352	0.64872	4.08791
P17182	20.92	22.23	27.880	Alpha-enolase	14	0.96954	1.01323	1.03064	1.06387
P17426	6.88	8.25	3.071	AP-2 complex subunit alpha-1	3	1.32264	3.58325	0.75549	2.71910
P17427	1.51	7.39	2.985	AP-2 complex subunit alpha-2	3	1.07590	1.83758	0.92875	1.71420
P17563	21.46	21.58	22.460	Selenium-binding protein 1	13	0.63125	0.47014	1.58295	0.69598
P17665	4	4	28.570	Cytochrome c oxidase subunit 7C, mitochondrial	3	1.45140	0.73703	0.68847	0.50392
P17710	1.96	6.46	3.080	Hexokinase-1	3	1.21752	1.15463	0.82072	0.94789

P17742	10.35	12.33	32.930	Peptidyl-prolyl cis-trans isomerase A	6	0.78872	1.47395	1.26692	1.83730
P17751	8.93	9.12	18.060	Triosephosphate isomerase	5	0.78767	0.80445	1.26860	1.02831
P17809	1.57	1.68	2.033	Solute carrier family 2, facilitated glucose transporter member 1	1	1.02375	0.80473	0.97606	0.78894
P18242	7.16	7.73	8.780	Cathepsin D	4	1.03905	1.53516	0.96169	1.49275
P18572	14.21	14.96	17.220	Basigin	12	0.79665	0.45857	1.25431	0.56799
P18760	13.65	16.23	49.400	Cofilin-1	11	0.82769	1.50496	1.20727	1.83341
P19001	62.44	62.44	73.700	Keratin, type I cytoskeletal 19	87	1.70139	0.52498	0.58731	0.32223
P19096	6.95	12.63	1.597	Fatty acid synthase	5	0.83739	1.17867	1.19329	1.41614
P19157	4.2	4.68	13.810	Glutathione S-transferase P 1	7	0.69894	0.88286	1.42965	1.26776
P19253	2.58	2.67	3.448	60S ribosomal protein L13a	1	0.98516	1.28457	1.01430	1.31536
P19324	2.68	2.8	2.638	Serpin H1	1	1.02782	2.43732	0.97219	2.38133
P19467	12.34	12.59	15.530	Mucin-13	11	0.67348	0.89271	1.48370	1.32864
P19536	10.61	10.85	30.470	Cytochrome c oxidase subunit 5B, mitochondrial	13	1.39084	0.74849	0.71845	0.57519
P19639	1.66	3.82	9.633	Glutathione S-transferase Mu 3	2	0.70667	0.44451	1.41402	0.63133
P19783	11.69	11.85	27.810	Cytochrome c oxidase subunit 4 isoform 1, mitochondrial	7	1.26113	0.79275	0.79234	0.63281
P20029	45.47	47.74	37.560	78 kDa glucose-regulated protein	39	1.39414	0.94370	0.71675	0.67245
P20060	2.03	4.51	2.985	Beta-hexosaminidase subunit beta	2	1.43702	1.11225	0.69536	0.77684
P20108	5.04	5.48	11.670	Thioredoxin-dependent peroxide reductase, mitochondrial	3	0.53480	0.55143	1.86843	1.03486
P20152	11.02	18.6	19.100	Vimentin	15	1.84865	1.67081	0.54053	0.91105
P21107	2.33	8.52	12.630	Tropomyosin alpha-3 chain	7	0.85181	1.03333	1.17308	1.21623
P21278	17.03	17.58	20.060	Guanine nucleotide-binding protein subunit alpha-11	9	0.53116	0.54953	1.88124	1.03837
P21279	2.1	14.07	17.270	Guanine nucleotide-binding protein G(q) subunit alpha	8	0.82065	1.02116	1.21762	1.23334
P21447	42.02	42.45	16.140	Multidrug resistance protein 1A	21	0.57161	0.42054	1.74811	0.73147
P22892	4.35	4.48	2.068	AP-1 complex subunit gamma-1	2	0.83817	1.01290	1.19217	1.21549
P23116	1.48	1.8	0.819	Eukaryotic translation initiation factor 3 subunit A	1	0.83729	1.05146	1.19342	1.24898

P23506	2	2.31	4.846	Protein-L-isoaspartate(D-aspartate) O-methyltransferase	1	1.18430	1.38362	0.84374	1.17257
P24270	4.39	4.44	4.744	Catalase	3	1.14525	1.02558	0.87251	0.89675
P24369	20.43	20.49	37.960	Peptidyl-prolyl cis-trans isomerase B	15	1.16010	1.15901	0.86134	1.00215
P24527	3.33	3.85	2.619	Leukotriene A-4 hydrolase	2	0.50465	0.72769	1.98007	1.42918
P24668	1.44	1.52	2.878	Cation-dependent mannose-6-phosphate receptor	1	1.04462	1.34983	0.95656	1.28906
P25444	3.4	3.54	4.437	40S ribosomal protein S2	2	0.83322	0.92662	1.19925	1.12188
P26039	10.92	13.83	1.928	Talin-1	5	0.95691	1.21495	1.04425	1.30514
P26040	26	29	17.750	Ezrin	23	0.65699	0.91415	1.52095	1.38362
P26041	4.28	12.76	6.759	Moesin	8	1.10130	3.29123	0.90733	3.01348
P26043	2	10.03	5.317	Radixin	7	0.77321	1.55079	1.29232	2.01299
P26150	2.38	2.56	5.630	3 beta-hydroxysteroid dehydrogenase/Delta 5-->4-isomerase type 3	2	1.55725	0.89297	0.64167	0.57703
P26231	59.09	59.93	31.130	Catenin alpha-1	50	1.09938	1.13063	0.90892	1.02760
P26369	2.28	2.36	2.105	Splicing factor U2AF 65 kDa subunit	1	0.87349	0.81469	1.14397	0.92110
P26443	16.21	16.74	15.770	Glutamate dehydrogenase 1, mitochondrial	8	1.07204	1.11295	0.93210	1.04041
P27546	2.56	2.78	0.800	Microtubule-associated protein 4	1	1.22308	1.20404	0.81699	0.97061
P27601	2.76	5.93	5.040	Guanine nucleotide-binding protein subunit alpha-13	2	0.57454	0.75972	1.73921	1.31038
P27659	3.42	3.9	4.715	60S ribosomal protein L3	2	0.85101	1.14488	1.17419	1.35429
P27773	50.03	50.4	49.700	Protein disulfide-isomerase A3	41	1.26427	0.77532	0.79037	0.61683
P28063	2.05	3.98	4.348	Proteasome subunit beta type-8	2	0.88088	1.15279	1.13436	1.32105
P28667	2.06	2.1	7.500	MARCKS-related protein	1	1.49142	2.66560	0.67000	1.81140
P28825	3.85	5.14	3.213	Meprin A subunit alpha	3	0.69048	0.50630	1.44717	0.73588
P28867	1.57	3.8	1.335	Protein kinase C delta type	1	0.69362	0.80238	1.44062	1.16392
P29341	2.14	2.99	1.572	Polyadenylate-binding protein 1	1	0.95770	0.96155	1.04338	1.00000
P29416	2.03	4.06	2.273	Beta-hexosaminidase subunit alpha	2	1.37291	1.35614	0.72783	0.99141
P29452	2.09	3.37	3.980	Caspase-1	2	0.81473	0.65318	1.22647	0.81045

P29595	1.69	1.83	13.580	NEDD8	1	0.86858	1.05121	1.15044	1.21194
P30275	26.28	26.39	29.900	Creatine kinase U-type, mitochondrial	13	1.01551	0.46609	0.98398	0.46555
P30416	2.01	2.12	2.183	Peptidyl-prolyl cis-trans isomerase FKBP4	1	0.80833	1.32570	1.23619	1.64607
P30681	2.81	2.81	6.190	High mobility group protein B2	1	1.30152	4.30547	0.76775	3.30439
P30999	26.65	27.12	13.650	Catenin delta-1	16	0.91494	1.03560	1.09214	1.13144
P31001	4.25	10.99	13.860	Desmin	10	1.34623	1.88630	0.74225	1.40705
P31428	3.94	4.36	6.829	Dipeptidase 1	4	0.48233	0.33840	2.07170	0.68753
P31725	6.44	6.46	30.090	Protein S100-A9	7	2.78372	6.61609	0.35896	2.53390
P31809	7.66	8.29	9.981	Carcinoembryonic antigen-related cell adhesion molecule 1	8	0.68300	0.31639	1.46302	0.46893
P32020	11.09	11.3	9.872	Non-specific lipid-transfer protein	8	1.25413	0.98343	0.79676	0.79626
P32883	3.7	3.96	16.400	GTPase KRas	3	0.81354	0.96740	1.22827	1.18990
P34022	1.85	2.12	5.419	Ran-specific GTPase-activating protein	1	0.72418	1.02118	1.37983	1.41528
P35276	4.75	8.36	19.180	Ras-related protein Rab-3D	7	1.03715	0.63996	0.96345	0.61866
P35278	12.24	12.29	26.850	Ras-related protein Rab-5C	8	1.03282	0.93845	0.96749	0.91083
P35279	13.11	15.42	37.500	Ras-related protein Rab-6A	13	0.95723	0.72204	1.04389	0.75833
P35282	2.18	2.38	4.955	Ras-related protein Rab-21	1	1.11290	0.97456	0.89787	0.87543
P35285	2.75	2.76	5.670	Ras-related protein Rab-22A	1	1.02276	1.53923	0.97701	1.51035
P35293	2.52	5.34	14.080	Ras-related protein Rab-18	3	1.08545	0.83247	0.92058	0.77099
P35486	10.54	10.98	15.130	Pyruvate dehydrogenase E1 component subunit alpha, somatic form, mitochondrial	6	1.03735	0.80710	0.96327	0.79154
P35564	14.24	14.5	13.200	Calnexin	7	1.53571	1.20440	0.65067	0.77132
P35700	20.7	22.54	45.230	Peroxiredoxin-1	19	0.88955	1.31834	1.12331	1.48123
P35979	6.11	6.61	28.480	60S ribosomal protein L12	5	1.12189	1.15253	0.89067	1.03357
P35980	2.02	2.27	6.915	60S ribosomal protein L18	1	0.61927	1.03987	1.61359	1.68919
P36536	2.18	2.23	5.556	GTP-binding protein SAR1a	1	1.07471	1.18647	0.92978	1.11123
P37040	2.46	2.66	2.065	NADPH--cytochrome P450 reductase	1	1.33546	0.76422	0.74824	0.57728

P37804	15.77	16.07	40.800	Transgelin	13	1.21319	1.55247	0.82365	1.28400
P38060	4.59	4.59	5.846	Hydroxymethylglutaryl-CoA lyase, mitochondrial	2	0.80962	0.50598	1.23421	0.62385
P38647	20.03	24.35	15.910	Stress-70 protein, mitochondrial	14	0.87761	0.61763	1.13859	0.71171
P39054	9.76	11.14	4.598	Dynamin-2	4	0.96170	1.07375	1.03904	1.33886
P39447	2.04	2.61	0.516	Tight junction protein ZO-1	1	1.11786	1.36425	0.89389	1.22489
P40124	3.59	3.72	8.228	Adenylyl cyclase-associated protein 1	3	1.14366	1.17158	0.87372	1.04140
P40142	14.85	15.16	10.430	Transketolase	8	0.93343	1.27526	1.07051	1.37036
P40240	2.34	2.34	3.097	CD9 antigen	1	0.87537	1.37708	1.14151	1.57357
P41105	2	2	5.109	60S ribosomal protein L28	1	0.68682	0.87210	1.45488	1.27442
P41216	2.34	3.46	1.288	Long-chain-fatty-acid--CoA ligase 1	1	1.06811	0.75598	0.93553	0.71649
P41731	4.09	4.19	7.563	CD63 antigen	3	1.05799	1.44397	0.94448	1.36983
P42125	4.11	4.45	9.689	Enoyl-CoA delta isomerase 1, mitochondrial	2	0.92745	0.78969	1.07741	0.84904
P42669	2	2.04	2.804	Transcriptional activator protein Pur-alpha	1	1.02493	1.10785	0.97494	1.08487
P42932	19.32	20.95	18.610	T-complex protein 1 subunit theta	9	0.83061	0.98396	1.20303	1.18688
P43274	3.25	3.41	5.023	Histone H1.4	1	0.90153	1.16245	1.10838	1.29770
P43276	2	2.21	4.933	Histone H1.5	1	0.87707	0.95851	1.13930	1.09686
P43406	9.51	10.83	4.310	Integrin alpha-V	4	1.06270	1.54682	0.94029	1.46432
P45376	2.26	2.35	4.430	Aldose reductase	1	0.79210	0.53921	1.26151	0.68323
P45952	9.94	10.83	11.640	Medium-chain specific acyl-CoA dehydrogenase, mitochondrial	4	0.99400	0.75685	1.00528	0.76304
P46460	11.28	12.38	8.602	Vesicle-fusing ATPase	6	1.01388	1.25451	0.98557	1.24378
P46467	7.24	7.64	8.108	Vacuolar protein sorting-associated protein 4B	4	0.87503	1.05758	1.14195	1.20775
P46471	7.62	10.86	9.007	26S protease regulatory subunit 7	5	0.92079	0.76406	1.08521	0.83476
P46978	2.81	2.87	1.560	Dolichyl-diphosphooligosaccharide--protein glycosyltransferase subunit STT3A	1	1.22802	1.08412	0.81370	0.88265
P47738	22.2	22.6	25.630	Aldehyde dehydrogenase, mitochondrial	16	0.90779	0.54735	1.10074	0.60581

P47753	4.1	4.26	8.741	F-actin-capping protein subunit alpha-1	3	0.98095	0.94983	1.01865	0.96961
P47757	4.14	4.65	6.859	F-actin-capping protein subunit beta	3	0.85207	0.99128	1.17272	1.17027
P47856	14.59	16.22	13.920	Glutamine--fructose-6-phosphate aminotransferase [isomerizing] 1	9	0.93349	0.65238	1.07044	0.70453
P47962	3.12	3.16	5.051	60S ribosomal protein L5	2	1.48574	2.98548	0.67255	2.03690
P47964	1.4	1.4	8.571	60S ribosomal protein L36	1	0.90094	1.15980	1.10911	1.29203
P48036	25.23	25.63	36.360	Annexin A5	15	1.22548	2.61988	0.81539	2.21379
P48428	1.48	1.48	6.481	Tubulin-specific chaperone A	1	0.92104	1.19875	1.08491	1.30629
P48453	1.74	2.79	2.286	Serine/threonine-protein phosphatase 2B catalytic subunit beta isoform	1	1.01531	0.91230	0.98417	0.90183
P48678	8.86	9.56	5.714	Prelamin-A/C	4	1.08028	1.41536	0.92498	1.31447
P48758	7.54	7.65	13.720	Carbonyl reductase [NADPH] 1	3	0.81184	0.56894	1.23084	0.70867
P48771	1.88	2.03	27.710	Cytochrome c oxidase subunit 7A2, mitochondrial	4	1.42343	0.80009	0.70200	0.56415
P49312	2	4.69	5.937	Heterogeneous nuclear ribonucleoprotein A1	2	0.67998	0.73609	1.46951	1.08648
P49817	3.31	3.31	8.427	Caveolin-1	2	2.11112	3.47577	0.47332	1.81902
P50516	4.98	5.24	5.835	V-type proton ATPase catalytic subunit A	6	0.84702	0.91244	1.17972	1.08103
P50518	2.25	2.4	5.310	V-type proton ATPase subunit E 1	1	0.87222	0.82912	1.14563	0.95491
P50544	16.75	17.5	15.090	Very long-chain specific acyl-CoA dehydrogenase, mitochondrial	9	1.22332	0.85650	0.81683	0.70381
P51150	18.3	18.3	44.440	Ras-related protein Rab-7a	14	0.90208	0.95185	1.10772	1.07423
P51174	2	3.2	3.023	Long-chain specific acyl-CoA dehydrogenase, mitochondrial	1	1.03657	0.76607	0.96399	0.74175
P51410	2.07	2.42	5.208	60S ribosomal protein L9	1	1.06054	1.23254	0.94220	1.16643
P51432	3.27	4.58	1.621	1-phosphatidylinositol 4,5-bisphosphate phosphodiesterase beta-3	2	0.76718	0.90193	1.30248	1.18359
P51437	5.87	6.11	17.920	Cathelin-related antimicrobial peptide	5	1.05792	2.86460	0.94453	2.71768
P51658	4.43	4.48	6.299	Estradiol 17-beta-dehydrogenase 2	2	1.64403	0.61442	0.60780	0.38016

P51660	10.08	10.46	7.075	Peroxisomal multifunctional enzyme type 2	4	1.32343	1.04794	0.75504	0.79839
P51661	7.39	7.76	10.360	Corticosteroid 11-beta-dehydrogenase isozyme 2	4	1.33282	0.93826	0.74972	0.71266
P51791	3.17	3.49	2.078	H(+)/Cl(-) exchange transporter 3	2	1.22036	1.66360	0.81881	1.36823
P51855	4.05	5.27	4.219	Glutathione synthetase	3	0.81281	0.87915	1.22937	1.08231
P51859	1.8	2.07	3.797	Hepatoma-derived growth factor	1	0.87403	1.09536	1.14325	1.26183
P51863	3.46	3.65	5.128	V-type proton ATPase subunit d 1	2	1.14827	1.35983	0.87022	1.19408
P51881	22.45	22.63	34.900	ADP/ATP translocase 2	20	1.20963	1.24330	0.82607	1.03477
P52196	7.04	7.19	17.850	Thiosulfate sulfurtransferase	5	0.82707	0.62100	1.20817	0.76403
P52480	25.71	27.27	32.960	Pyruvate kinase PKM	16	0.90278	1.19236	1.10685	1.33694
P52503	4.01	4.01	22.410	NADH dehydrogenase [ubiquinone] iron-sulfur protein 6, mitochondrial	2	1.00114	0.59854	0.99811	0.60056
P52795	4.86	5.8	8.986	Ephrin-B1	2	0.99795	0.79544	1.00130	0.79750
P52825	2.7	3.13	1.216	Carnitine O-palmitoyltransferase 2, mitochondrial	1	1.04134	0.97550	0.95958	0.94388
P52840	4.42	4.58	8.591	Sulfotransferase 1A1	3	0.76951	0.27823	1.29854	0.36232
P53395	5.19	5.69	3.112	Lipoamide acyltransferase component of branched-chain alpha-keto acid dehydrogenase complex, mitochondrial	2	1.06276	0.74242	0.94023	0.70294
P53702	2	2.02	4.779	Cytochrome c-type heme lyase	1	1.10937	0.90801	0.90073	0.82149
P53986	4.94	5.1	4.462	Monocarboxylate transporter 1	4	0.85051	0.65520	1.17488	0.77072
P53994	14.85	15.26	33.490	Ras-related protein Rab-2A	8	0.98552	0.67859	1.01393	0.69759
P54071	8.29	10.58	12.390	Isocitrate dehydrogenase [NADP], mitochondrial	6	1.10355	0.82102	0.90548	0.74637
P54116	1.92	2.4	4.930	Erythrocyte band 7 integral membrane protein	1	0.78498	1.64699	1.27296	2.12224
P54775	2	2.11	2.153	26S protease regulatory subunit 6B	1	0.87390	0.69676	1.14342	0.80021
P54869	25.79	26.8	23.230	Hydroxymethylglutaryl-CoA synthase, mitochondrial	23	1.19169	0.38309	0.83851	0.32484
P55012	25.17	25.8	11.290	Solute carrier family 12 member 2	16	0.94505	1.14959	1.05735	1.28586
P55050	1.38	1.45	6.818	Fatty acid-binding protein, intestinal	1	0.60834	0.20791	1.64258	0.33648
P55088	2.6	4.41	2.477	Aquaporin-4	1	0.66952	0.36856	1.49248	0.55289

P55264	1.82	2.37	2.216	Adenosine kinase	1	0.58923	0.45438	1.69585	0.77397
P55292	1.31	2.31	0.887	Desmocollin-2	1	1.04567	1.28014	0.95560	1.22872
P55302	2.02	2.4	1.944	Alpha-2-macroglobulin receptor-associated protein	1	1.20184	1.18238	0.83143	0.98704
P56382	1.76	1.79	13.460	ATP synthase subunit epsilon, mitochondrial	2	0.99171	0.78678	1.00760	0.79166
P56383	2	2.32	4.795	ATP synthase F(0) complex subunit C2, mitochondrial	1	1.01295	0.89487	0.98647	0.88666
P56391	7.25	7.29	40.700	Cytochrome c oxidase subunit 6B1	6	1.23229	0.57196	0.81088	0.46638
P56395	7.88	7.98	45.520	Cytochrome b5	5	1.49986	0.81221	0.66622	0.54434
P56480	40.73	42.35	47.450	ATP synthase subunit beta, mitochondrial	45	1.14215	0.64370	0.87488	0.55739
P56528	8.13	8.33	15.790	ADP-ribosyl cyclase 1	7	0.97282	0.88301	1.02716	0.91374
P56677	4.53	4.59	2.456	Suppressor of tumorigenicity 14 protein homolog	2	0.91130	0.94988	1.09650	1.04534
P57716	9.49	9.97	7.203	Nicastrin	6	0.99043	1.21942	1.00890	1.24411
P57759	4.48	4.73	8.397	Endoplasmic reticulum resident protein 29	3	1.09075	1.02127	0.91611	0.93747
P57776	3.91	4.08	8.541	Elongation factor 1-delta	4	1.28486	0.94871	0.77770	0.75163
P57780	39.54	40.03	25.660	Alpha-actinin-4	29	0.79197	0.81065	1.26172	1.02672
P57784	2.14	2.24	2.353	U2 small nuclear ribonucleoprotein A'	1	1.31547	1.31562	0.75961	0.99993
P58021	10.39	10.6	7.553	Transmembrane 9 superfamily member 2	6	1.60185	0.93822	0.62381	0.57656
P58252	29.14	30.07	17.720	Elongation factor 2	19	1.17213	1.47098	0.85250	1.26921
P58281	7.01	9.7	3.958	Dynamin-like 120 kDa protein, mitochondrial	4	1.21882	1.12523	0.81984	0.92706
P58771	9.79	10.36	17.250	Tropomyosin alpha-1 chain	8	0.80259	1.15820	1.24503	1.45596
P59242	4.73	7.69	2.771	Cingulin	4	1.16227	0.91352	0.85973	0.78880
P59999	4.86	4.86	11.310	Actin-related protein 2/3 complex subunit 4	3	0.94550	1.08484	1.05684	1.15401
P60335	8.44	8.71	13.760	Poly(rC)-binding protein 1	6	1.03141	1.42997	0.96881	1.39680
P60766	6.9	6.9	24.080	Cell division control protein 42 homolog	4	0.89867	1.01331	1.11192	1.13303
P60843	6.62	8.84	8.621	Eukaryotic initiation factor 4A-I	6	0.99734	1.32062	1.00190	1.34650
P61022	6.57	6.82	20.510	Calcineurin B homologous protein 1	3	0.71004	0.58380	1.40730	0.82223
P61027	2.93	9.34	20.500	Ras-related protein Rab-10	5	0.79529	0.72934	1.25644	0.91171

P61082	1.85	1.85	6.011	NEDD8-conjugating enzyme Ubc12	1	0.93459	0.97505	1.06918	1.04711
P61089	5.49	5.75	21.050	Ubiquitin-conjugating enzyme E2 N	4	0.93889	1.14994	1.06428	1.24623
P61161	10.46	11.39	20.560	Actin-related protein 2	9	0.98946	0.96384	1.00989	0.97920
P61211	2.65	2.65	6.077	ADP-ribosylation factor-like protein 1	1	1.18372	1.17817	0.84415	1.00029
P61620	2.12	2.68	1.891	Protein transport protein Sec61 subunit alpha isoform 1	1	1.21706	1.23721	0.82103	1.01472
P61804	4	4	19.470	Dolichyl-diphosphooligosaccharide--protein glycosyltransferase subunit DAD1	2	1.34895	0.93573	0.74076	0.69661
P61924	2	2.04	6.215	Coatomer subunit zeta-1	1	0.80316	0.68076	1.24414	0.85071
P61961	2	2.35	17.650	Ubiquitin-fold modifier 1	1	0.99262	1.16182	1.00668	1.17476
P61979	15.75	16.1	17.490	Heterogeneous nuclear ribonucleoprotein K	8	1.22714	0.91366	0.81429	0.74689
P61982	5.4	10.15	18.220	14-3-3 protein gamma	7	1.07962	1.21879	0.92555	1.13304
P62071	4.68	4.74	9.804	Ras-related protein R-Ras2	2	0.94998	1.04675	1.05186	1.10676
P62137	3.41	3.48	5.455	Serine/threonine-protein phosphatase PP1-alpha catalytic subunit	2	1.08045	1.21134	0.92484	1.13315
P62196	4.36	6.29	5.172	26S protease regulatory subunit 8	3	1.08705	0.88750	0.91922	0.82034
P62204	10.65	10.83	38.260	Calmodulin	10	0.80186	0.66394	1.24616	0.66224
P62242	1.46	1.51	4.327	40S ribosomal protein S8	1	1.16948	1.60981	0.85444	1.38157
P62245	3.06	3.2	12.310	40S ribosomal protein S15a	2	1.01445	1.15304	0.98501	1.13212
P62259	8	12.29	23.920	14-3-3 protein epsilon	9	0.80253	1.03896	1.24512	1.30268
P62264	2	2.04	8.609	40S ribosomal protein S14	1	1.02995	1.09993	0.97019	1.07186
P62267	2	2.04	7.692	40S ribosomal protein S23	1	1.14587	2.58443	0.87204	2.26370
P62281	5.34	5.63	16.460	40S ribosomal protein S11	3	0.79114	1.14456	1.26304	1.44950
P62311	2	2.04	11.760	U6 snRNA-associated Sm-like protein LSm3	1	0.71431	0.61812	1.39890	0.86852
P62320	1.58	1.65	7.143	Small nuclear ribonucleoprotein Sm D3	1	2.13743	2.61732	0.46750	1.22901
P62331	5.66	7.41	23.430	ADP-ribosylation factor 6	6	0.69116	0.99576	1.44575	1.44325
P62492	14.19	14.19	31.940	Ras-related protein Rab-11A	8	0.94196	0.77752	1.06081	0.82852

P62702	2.34	2.38	3.422	40S ribosomal protein S4, X isoform	1	0.84605	1.73407	1.18107	2.03805
P62746	3.12	5.26	18.370	Rho-related GTP-binding protein RhoB	5	0.71614	0.92977	1.39533	1.29689
P62748	4.09	4.45	10.880	Hippocalcin-like protein 1	3	0.92007	0.76393	1.08606	0.83593
P62751	2.81	3.22	5.128	60S ribosomal protein L23a	1	0.93687	1.45640	1.06658	1.56184
P62754	2.72	2.87	3.213	40S ribosomal protein S6	1	0.87560	1.11469	1.14121	1.29130
P62806	8.2	8.27	38.830	Histone H4	6	1.23807	2.72803	0.80710	2.18555
P62814	4.78	5.35	5.675	V-type proton ATPase subunit B, brain isoform	2	1.00013	0.77741	0.99911	0.78697
P62821	17.49	17.68	38.050	Ras-related protein Rab-1A	10	0.81537	0.75191	1.22551	0.92309
P62830	3.89	3.92	16.430	60S ribosomal protein L23	2	1.16265	1.43997	0.85945	1.25708
P62838	2	2.01	7.483	Ubiquitin-conjugating enzyme E2 D2	1	0.99984	1.35921	0.99940	1.36441
P62855	2	2	7.826	40S ribosomal protein S26	1	0.80008	1.23965	1.24893	1.55508
P62858	4.08	4.1	30.430	40S ribosomal protein S28	2	1.02188	0.89811	0.97785	0.88188
P62862	2	2	16.950	40S ribosomal protein S30	1	0.76310	0.90713	1.30946	1.19311
P62869	2.72	3.19	13.560	Transcription elongation factor B polypeptide 2	2	0.85376	0.85421	1.17041	1.00698
P62874	3.49	14.79	25.590	Guanine nucleotide-binding protein G(I)/G(S)/G(T) subunit beta-1	9	0.82863	0.96940	1.20590	1.16677
P62880	14.33	14.42	23.530	Guanine nucleotide-binding protein G(I)/G(S)/G(T) subunit beta-2	9	1.03470	1.03558	0.96574	1.00884
P62889	1.42	1.51	5.217	60S ribosomal protein L30	1	1.17912	1.34621	0.84745	1.14590
P62897	8.06	8.07	43.810	Cytochrome c, somatic	6	1.29853	0.62542	0.76952	0.48384
P62908	14.34	14.43	29.630	40S ribosomal protein S3	9	1.03478	1.22227	0.96566	1.18511
P62962	14.27	14.3	60.000	Profilin-1	16	0.70990	1.59732	1.40758	2.23796
P62984	8.49	8.66	31.250	Ubiquitin-60S ribosomal protein L40	7	1.23070	1.81279	0.81193	1.46342
P63001	1.3	4.57	17.190	Ras-related C3 botulinum toxin substrate 1	4	0.76648	1.14711	1.30367	1.50207
P63017	32.63	39.64	32.970	Heat shock cognate 71 kDa protein	33	0.97756	1.10014	1.02218	1.12285
P63028	2.18	2.2	7.558	Translationally-controlled tumor protein	1	0.83379	1.02996	1.19843	1.24381

P63038	27.7	30.17	27.750	60 kDa heat shock protein, mitochondrial	20	0.90298	0.51716	1.10661	0.57640
P63044	2.03	2.07	14.660	Vesicle-associated membrane protein 2	4	0.81217	1.04535	1.23034	1.29745
P63073	2	2.04	3.226	Eukaryotic translation initiation factor 4E	1	0.87304	0.86941	1.14455	0.99949
P63101	19.66	19.86	35.510	14-3-3 protein zeta/delta	17	0.85494	1.02821	1.16878	1.21801
P63168	2.06	2.47	7.865	Dynein light chain 1, cytoplasmic	1	0.76298	0.92747	1.30966	1.22005
P63213	2	2	22.540	Guanine nucleotide-binding protein G(I)/G(S)/G(O) subunit gamma-2	1	1.16507	2.52203	0.85767	2.17264
P63242	4.3	4.33	14.940	Eukaryotic translation initiation factor 5A-1	3	0.88095	1.15015	1.13428	1.31379
P63276	2	2.17	8.148	40S ribosomal protein S17	1	0.79814	0.90892	1.25197	1.14297
P63323	4	4.02	13.640	40S ribosomal protein S12	2	1.02703	0.97115	0.97294	0.95767
P67778	18.03	18.11	32.720	Prohibitin	12	1.18592	0.90752	0.84259	0.76499
P67984	2.75	2.86	8.594	60S ribosomal protein L22	1	1.27155	1.51427	0.78585	1.20373
P68037	1.62	2.16	4.545	Ubiquitin-conjugating enzyme E2 L3	1	0.87382	1.02093	1.14353	1.16674
P68040	6.64	7.06	9.779	Guanine nucleotide-binding protein subunit beta-2-like 1	3	1.37329	1.24073	0.72763	0.94538
P68368	4.96	16.77	20.980	Tubulin alpha-4A chain	12	1.13707	1.08120	0.87879	0.96739
P68372	20.64	21	24.490	Tubulin beta-4B chain	20	0.98715	0.82905	1.01225	0.84875
P68373	19.57	19.78	23.390	Tubulin alpha-1C chain	14	1.26976	1.70646	0.78695	1.31569
P68510	2.01	9.02	17.480	14-3-3 protein eta	7	0.73648	1.19020	1.35679	1.62199
P70124	3.78	4.85	10.130	Serpin B5	4	0.81635	1.02755	1.22404	1.26326
P70296	4.03	4.29	14.440	Phosphatidylethanolamine-binding protein 1	2	0.84989	0.77451	1.17573	0.92845
P70302	4.54	5.18	2.920	Stromal interaction molecule 1	2	1.31984	0.98008	0.75709	0.74086
P70333	1.41	2.62	1.336	Heterogeneous nuclear ribonucleoprotein H2	1	0.61480	0.66929	1.62531	1.08831
P70349	2	2.15	11.110	Histidine triad nucleotide-binding protein 1	2	0.69957	0.74694	1.42836	1.07163
P70404	2.01	2.12	2.036	Isocitrate dehydrogenase [NAD] subunit gamma 1, mitochondrial	1	1.20301	0.88215	0.83062	0.73597
P70441	13.49	14.53	24.510	Na(+)/H(+) exchange regulatory cofactor NHE-RF1	9	0.51308	0.43382	1.94756	0.84615

P70452	2	2.12	3.691	Syntaxin-4	1	0.82262	0.67127	1.21470	0.81900
P70460	6.76	7.1	8.533	Vasodilator-stimulated phosphoprotein	3	0.99242	1.06871	1.00688	1.08695
P70695	2	2.12	3.540	Fructose-1,6-bisphosphatase isozyme 2	1	0.81098	0.57673	1.23214	0.71376
P80313	4.42	5.6	4.963	T-complex protein 1 subunit eta	3	1.01142	1.29618	0.98796	1.28605
P80314	11.24	12.23	13.640	T-complex protein 1 subunit beta	6	0.94268	1.01385	1.06000	1.06656
P80315	2.46	5.38	5.937	T-complex protein 1 subunit delta	3	0.90006	1.15601	1.11019	1.28850
P80316	14.7	16.45	9.797	T-complex protein 1 subunit epsilon	7	0.93004	1.13294	1.07441	1.22300
P80317	2.67	3.85	3.202	T-complex protein 1 subunit zeta	3	1.02112	1.20321	0.97857	1.18708
P80318	8.74	13.28	6.972	T-complex protein 1 subunit gamma	4	0.98919	1.29820	1.01016	1.32172
P83870	2	2.04	7.273	PHD finger-like domain-containing protein 5A	1	0.99987	1.17249	0.99937	1.17693
P83940	2	2.01	8.036	Transcription elongation factor B polypeptide 1	1	1.01793	0.75495	0.98165	0.74437
P84078	12.02	13.2	35.360	ADP-ribosylation factor 1	12	0.79221	0.87795	1.26135	1.11286
P84089	2	2	6.731	Enhancer of rudimentary homolog	1	1.07018	0.99372	0.93371	0.93195
P84091	2.04	2.09	1.609	AP-2 complex subunit mu	1	0.89107	2.95264	1.12140	3.32573
P84096	2.76	4.95	14.660	Rho-related GTP-binding protein RhoG	3	0.89506	1.15555	1.11640	1.30710
P97300	1.52	1.52	1.511	Neuroplastin	1	0.74801	0.74236	1.33586	0.99687
P97315	6.01	6.62	21.760	Cysteine and glycine-rich protein 1	4	1.11451	1.44883	0.89657	1.36020
P97360	1.84	1.93	1.031	Transcription factor ETV6	1	0.48236	0.98106	2.07155	2.04131
P97371	11.51	12.13	23.290	Proteasome activator complex subunit 1	7	0.83682	0.89813	1.19410	1.08062
P97372	7.21	7.21	11.300	Proteasome activator complex subunit 2	3	0.80339	0.71469	1.24378	0.88549
P97384	17.41	23.28	24.060	Annexin A11	18	0.99664	1.03667	1.00261	1.03822
P97429	23.59	28.81	49.220	Annexin A4	24	1.01048	1.49240	0.98888	1.48613
P97447	3.19	3.21	8.214	Four and a half LIM domains protein 1	4	0.97328	0.89680	1.02667	0.92480
P97450	6.9	7.03	35.190	ATP synthase-coupling factor 6, mitochondrial	6	1.22433	0.82540	0.81616	0.69066
P97792	6.41	6.46	9.041	Coxsackievirus and adenovirus receptor homolog	4	0.93867	0.96603	1.06453	1.03761
P97805	8.45	8.58	17.490	Protein FAM3D	9	1.07611	0.68326	0.92857	0.63750

P97807	11.32	12.14	9.270	Fumarate hydratase, mitochondrial	7	0.76895	0.57184	1.29950	0.74996
P97823	2.07	2.13	4.783	Acyl-protein thioesterase 1	1	0.78870	0.85203	1.26695	1.08451
P97855	2.49	2.75	2.366	Ras GTPase-activating protein-binding protein 1	1	1.36801	1.38378	0.73043	1.01615
P97864	2	2.5	3.300	Caspase-7	1	1.43944	1.17604	0.69419	0.82001
P97930	2	2.76	5.189	Thymidylate kinase	1	0.83708	0.93397	1.19373	1.11984
P99024	6	20.98	24.550	Tubulin beta-5 chain	18	1.22988	1.77910	0.81247	1.46617
P99027	8.21	8.21	76.520	60S acidic ribosomal protein P2	8	0.83138	0.63569	1.20192	0.84591
P99028	6.03	6.03	43.820	Cytochrome b-c1 complex subunit 6, mitochondrial	6	1.03726	0.67883	0.96334	0.66542
P99029	6.89	6.98	26.190	Peroxiredoxin-5, mitochondrial	4	0.76496	0.89859	1.30627	1.18047
Q00623	2.12	2.92	4.167	Apolipoprotein A-I	1	1.39018	1.27618	0.71879	0.92135
Q01279	1.44	1.95	0.992	Epidermal growth factor receptor	1	1.47260	1.35404	0.67856	0.92844
Q01339	2.11	2.24	3.188	Beta-2-glycoprotein 1	1	1.39518	1.61836	0.71621	1.16192
Q01853	26.13	27.13	13.770	Transitional endoplasmic reticulum ATPase	14	1.09789	1.06658	0.91015	0.97967
Q02053	5.52	7.29	2.741	Ubiquitin-like modifier-activating enzyme 1	3	1.05188	1.04560	0.94996	1.00029
Q02248	24.15	24.23	20.100	Catenin beta-1	21	1.07785	1.08000	0.92707	1.00785
Q02257	13.43	20.26	13.960	Junction plakoglobin	13	1.05770	1.01148	0.94473	0.96046
Q02819	9.16	9.88	12.640	Nucleobindin-1	7	1.10202	0.79120	0.90674	0.71884
Q03265	39.4	43.43	35.260	ATP synthase subunit alpha, mitochondrial	34	1.31465	0.82502	0.76008	0.64069
Q04447	2.45	7.12	6.824	Creatine kinase B-type	2	0.86294	1.10640	1.15796	1.28572
Q05144	3.05	5.35	17.190	Ras-related C3 botulinum toxin substrate 2	3	0.93138	1.09092	1.07287	1.17559
Q06138	3.8	5.52	2.933	Calcium-binding protein 39	2	0.76502	0.91257	1.30617	1.20551
Q06185	4.56	4.8	50.700	ATP synthase subunit e, mitochondrial	4	1.10646	0.99426	0.90310	0.89994
Q07076	19.64	20.4	21.170	Annexin A7	11	1.03225	1.81147	0.96803	1.77732
Q07113	4.64	6.08	0.765	Cation-independent mannose-6-phosphate receptor	2	1.09572	0.98781	0.91195	0.90000
Q07417	8.62	8.75	11.170	Short-chain specific acyl-CoA dehydrogenase, mitochondrial	5	1.02699	0.47335	0.97298	0.46161
Q07797	2.8	3.28	1.733	Galectin-3-binding protein	1	1.16686	1.43911	0.85635	1.23721

Q08091	1.48	1.52	3.704	Calponin-1	1	1.18110	1.06267	0.84603	0.90223
Q08189	6.92	7.11	3.896	Protein-glutamine gamma-glutamyltransferase E	4	0.55322	0.45417	1.80623	0.81838
Q08509	9.07	9.8	6.212	Epidermal growth factor receptor kinase substrate 8	4	0.62874	0.75511	1.58928	1.20921
Q09199	8.58	9.06	9.608	Beta-1,4 N-acetylgalactosaminyltransferase 2	5	0.95085	0.54714	1.05089	0.58189
Q09200	2.13	2.43	2.064	Beta-1,4 N-acetylgalactosaminyltransferase 1	1	0.91654	0.80033	1.09023	0.74128
Q10470	2.05	2.69	2.788	Beta-1,4-mannosyl-glycoprotein 4-beta-N-acetylglucosaminyltransferase	1	0.93909	0.27105	1.06405	0.28969
Q148W0	4.26	5.01	1.918	Probable phospholipid-transporting ATPase IC	2	0.84841	0.91524	1.17779	1.08439
Q2KHK6	5.09	7.82	7.500	Gasdermin-C2	6	0.92213	0.76291	1.08362	0.83241
Q3THE2	10.8	10.97	27.910	Myosin regulatory light chain 12B	8	1.05799	1.33165	0.94447	1.26899
Q3THS6	1.44	1.67	1.772	S-adenosylmethionine synthase isoform type-2	1	0.71104	1.11124	1.40533	1.56856
Q3TMQ6	2.04	2.04	6.944	Angiogenin-4	1	0.83670	0.41706	1.19427	0.50668
Q3TTY5	2.02	8.45	5.092	Keratin, type II cytoskeletal 2 epidermal	6	0.37006	0.65356	2.70024	1.77259
Q3TWI9	2	2.74	1.082	Transmembrane protein 63B	1	0.98198	0.85272	1.01758	0.87155
Q3U1J4	2.14	4.34	0.877	DNA damage-binding protein 1	1	1.20475	1.08956	0.82942	0.90779
Q3UDR8	3.72	3.72	5.476	Protein YIPF3	2	1.06943	0.67363	0.93437	0.63057
Q3ULJ0	2.07	2.36	2.279	Glycerol-3-phosphate dehydrogenase 1-like protein	1	0.91993	0.71668	1.08621	0.78222
Q3UMR5	1.59	2.47	3.143	Calcium uniporter protein, mitochondrial	2	1.25493	0.81067	0.79625	0.66600
Q3UQ44	5.29	12.93	2.984	Ras GTPase-activating-like protein IQGAP2	5	0.93624	0.86247	1.06729	0.92784
Q3USF0	4.08	4.14	5.371	UDP-GlcNAc:betaGal beta-1,3-N-acetylglucosaminyltransferase 6	2	0.95419	0.70344	1.04722	0.73709
Q3UTJ2	2.22	5.15	1.356	Sorbin and SH3 domain-containing protein 2	2	1.19852	1.86308	0.83373	1.58377
Q3UW53	5.27	7.09	3.348	Protein Niban	4	1.01195	1.01577	0.98744	1.01016
Q3UZZ6	2.16	3.28	4.746	Sulfotransferase 1 family member D1	2	0.69595	0.64675	1.43580	0.94079
Q3V0K9	11.9	14.57	10.630	Plastin-1	8	0.60716	0.53671	1.64578	0.88480
Q3V3R1	5.55	7.97	4.913	Monofunctional C1-tetrahydrofolate synthase, mitochondrial	5	1.21852	0.96080	0.82005	0.79574

Q3V3R4	1.73	2.73	1.272	Integrin alpha-1	2	0.91802	1.62194	1.08847	1.81025
Q45VN2	2.38	2.38	11.580	Alpha-defensin 20	1	1.17357	1.91238	0.85146	1.69591
Q501J6	2.88	13.43	11.690	Probable ATP-dependent RNA helicase DDX17	7	1.31319	1.23625	0.76093	0.94777
Q505F5	2.06	2.38	2.065	Leucine-rich repeat-containing protein 47	1	1.02574	1.00197	0.97416	0.99203
Q5FWI3	1.55	4.21	0.868	Transmembrane protein 2	1	0.97957	1.19188	1.02009	1.21633
Q5FWK3	2.09	2.24	2.278	Rho GTPase-activating protein 1	1	1.23192	1.17315	0.81113	0.95578
Q5JCS9	4.54	4.65	4.032	UDP-GlcNAc:betaGal beta-1,3-N-acetylglucosaminyltransferase 3	2	1.21028	0.93706	0.82563	0.77378
Q5SRX1	2.03	3.23	1.775	TOM1-like protein 2	2	0.73803	0.84636	1.35393	1.15098
Q5XJY5	4.18	4.48	3.914	Coatomer subunit delta	2	0.99192	0.94397	1.00738	0.95581
Q60597	3.86	4.49	1.760	2-oxoglutarate dehydrogenase, mitochondrial	2	0.99513	0.72897	1.00414	0.73538
Q60598	8.11	8.18	7.326	Src substrate cortactin	5	1.07185	0.83446	0.93226	0.77997
Q60604	10.51	12.48	8.252	Adseverin	7	0.55592	0.46588	1.79746	0.83914
Q60605	11.49	11.57	40.400	Myosin light polypeptide 6	10	1.07030	1.03559	0.93361	0.98200
Q60634	4.27	4.64	5.140	Flotillin-2	2	1.12505	2.08036	0.88818	1.87248
Q60668	3.95	4.29	6.197	Heterogeneous nuclear ribonucleoprotein D0	2	0.42204	0.27575	2.36765	0.65463
Q60766	3.81	4.08	6.357	Immunity-related GTPase family M protein 1	2	1.55415	1.58544	0.64295	1.03220
Q60770	2.28	2.92	1.689	Syntaxin-binding protein 3	1	0.83143	1.09410	1.20183	1.32172
Q60854	11.5	11.88	15.610	Serpin B6	5	0.74897	0.75059	1.33415	1.00910
Q60864	4.84	5.13	4.052	Stress-induced-phosphoprotein 1	2	0.89289	0.96546	1.11911	1.08912
Q60870	3.7	3.85	9.189	Receptor expression-enhancing protein 5	2	1.20994	1.16478	0.82586	0.96746
Q60902	3.71	4.56	2.315	Epidermal growth factor receptor substrate 15-like 1	2	0.87079	1.24553	1.14751	1.43653
Q60930	15.86	16.25	30.850	Voltage-dependent anion-selective channel protein 2	9	1.42982	1.14454	0.69886	0.79178
Q60931	5.51	8.09	12.010	Voltage-dependent anion-selective channel protein 3	5	1.32335	1.11623	0.75508	0.84561
Q60932	16.8	17.54	32.770	Voltage-dependent anion-selective channel protein 1	14	1.48735	0.99421	0.67183	0.67275
Q61081	4.8	5.22	6.860	Hsp90 co-chaperone Cdc37	2	1.17565	1.21611	0.84995	1.03821

Q61112	1.82	2.15	1.939	45 kDa calcium-binding protein	1	1.51833	0.75194	0.65812	0.49705
Q61165	1.66	2.24	0.854	Sodium/hydrogen exchanger 1	1	0.92604	0.78638	1.07905	0.85411
Q61171	7.95	8.7	26.260	Peroxiredoxin-2	6	0.73174	1.30960	1.36558	1.78220
Q61176	1.54	1.68	2.477	Arginase-1	1	0.92614	2.28582	1.07894	2.47717
Q61187	2.19	2.49	3.069	Tumor susceptibility gene 101 protein	1	0.86517	1.50545	1.15497	1.74644
Q61205	2.69	3.84	3.879	Platelet-activating factor acetylhydrolase IB subunit gamma	2	0.78883	0.99934	1.26674	1.27635
Q61206	2	3.18	3.930	Platelet-activating factor acetylhydrolase IB subunit beta	2	0.63167	0.68859	1.58192	1.09411
Q61207	5.83	6.47	5.566	Sulfated glycoprotein 1	5	0.89348	1.04469	1.11838	1.17264
Q61233	4.61	7.9	4.785	Plastin-2	3	1.01976	1.53124	0.97988	1.51306
Q61316	3.23	5.22	3.092	Heat shock 70 kDa protein 4	2	0.80278	1.15417	1.24473	1.44235
Q61335	7.51	7.77	9.796	B-cell receptor-associated protein 31	4	1.51734	1.10358	0.65855	0.72749
Q61420	2	2.31	3.571	CMP-sialic acid transporter	1	1.31737	0.70768	0.75851	0.53915
Q61425	14.89	15.92	23.250	Hydroxyacyl-coenzyme A dehydrogenase, mitochondrial	8	1.07085	0.50743	0.93313	0.50982
Q61503	4.28	4.99	3.472	5'-nucleotidase	2	0.66507	1.28414	1.50247	1.93512
Q61526	2	5.05	0.747	Receptor tyrosine-protein kinase erbB-3	2	0.78487	0.57180	1.27313	0.73119
Q61543	25.32	27.19	9.362	Golgi apparatus protein 1	13	1.22249	1.17752	0.81738	0.96845
Q61598	9.11	9.38	14.830	Rab GDP dissociation inhibitor beta	7	0.75591	0.84347	1.32191	1.12063
Q61599	1.33	2.65	5.500	Rho GDP-dissociation inhibitor 2	2	0.71439	1.49063	1.39873	2.09420
Q61656	16.38	16.53	12.050	Probable ATP-dependent RNA helicase DDX5	7	1.22536	1.31502	0.81547	1.07635
Q61696	3.54	12.91	10.920	Heat shock 70 kDa protein 1A	14	0.78283	0.97309	1.27645	1.24521
Q61735	5.6	7.58	12.870	Leukocyte surface antigen CD47	4	1.15837	1.72203	0.86263	1.49722
Q61739	21.48	24.44	10.080	Integrin alpha-6	13	1.00020	1.38369	0.99904	1.37485
Q61792	2.1	3.19	5.323	LIM and SH3 domain protein 1	2	0.77167	1.05623	1.29491	1.38294
Q61847	1.62	1.81	1.420	Meprin A subunit beta	1	0.83543	0.75275	1.19609	0.90299
Q61897	7.69	8.16	12.620	Keratin, type I cuticular Ha3-II	7	3.36926	1.93921	0.29658	0.62962
Q61937	4.03	4.68	5.479	Nucleophosmin	2	1.70371	1.43426	0.58651	0.84432

Q61941	10.32	12.09	4.972	NAD(P) transhydrogenase, mitochondrial	5	1.14229	1.34884	0.87478	1.18419
Q61990	2.45	6.81	10.500	Poly(rC)-binding protein 2	3	0.91238	1.21855	1.09521	1.34356
Q62086	1.88	2.55	2.542	Serum paraoxonase/arylesterase 2	2	1.37923	0.82931	0.72450	0.60333
Q62087	6.42	6.52	11.300	Serum paraoxonase/lactonase 3	3	1.49747	0.90475	0.66729	0.62358
Q62167	2.21	5.08	3.323	ATP-dependent RNA helicase DDX3X	2	1.26091	1.10526	0.79248	0.88932
Q62186	6	6	19.770	Translocon-associated protein subunit delta	4	1.49113	0.98657	0.67013	0.66197
Q62189	1.92	2.08	2.787	U1 small nuclear ribonucleoprotein A	1	0.97460	1.18505	1.02528	1.22039
Q62261	46.52	50.53	9.099	Spectrin beta chain, non-erythrocytic 1	22	1.10447	1.09773	0.90473	0.99356
Q62273	4.16	4.89	2.436	Sulfate transporter	2	0.86958	0.60006	1.14911	0.70252
Q62313	4.03	4.35	5.099	Trans-Golgi network integral membrane protein 1	2	0.89821	0.68439	1.11248	0.76330
Q62351	4.14	4.92	2.752	Transferrin receptor protein 1	2	1.28032	1.30623	0.78046	1.02556
Q62393	2.65	3.91	11.160	Tumor protein D52	2	0.83885	0.65853	1.19121	0.78482
Q62425	6.08	6.08	30.490	NADH dehydrogenase [ubiquinone] 1 alpha subcomplex subunit 4	4	1.23091	1.12640	0.81179	0.90760
Q62433	2.05	2.22	4.061	Protein NDRG1	2	1.04890	0.56961	0.95266	0.54322
Q62465	8.76	8.76	10.840	Synaptic vesicle membrane protein VAT-1 homolog	4	1.00854	1.05142	0.99079	1.04742
Q62468	48.2	48.66	28.300	Villin-1	35	0.49910	0.70382	2.00207	1.39965
Q62469	18.23	21.1	10.950	Integrin alpha-2	13	0.94368	0.86765	1.05888	0.92320
Q62470	9.07	9.79	3.609	Integrin alpha-3	6	0.83289	0.97045	1.19973	1.17015
Q63844	1.89	2.3	6.316	Mitogen-activated protein kinase 3	2	1.02590	1.03346	0.97402	1.01122
Q64133	11.05	12.74	11.980	Amine oxidase [flavin-containing] A	6	1.08437	0.63396	0.92150	0.59600
Q64433	7.71	7.78	41.180	10 kDa heat shock protein, mitochondrial	5	0.72886	0.83822	1.37097	1.15889
Q64435	5	5.32	4.896	UDP-glucuronosyltransferase 1-6	4	1.35526	1.71393	0.73731	1.35132
Q64444	7.49	7.49	17.700	Carbonic anhydrase 4	11	0.42891	0.19848	2.32970	0.44700
Q64471	2	2.42	4.167	Glutathione S-transferase theta-1	1	0.99703	0.84232	1.00222	0.84793
Q64478	8.09	8.16	29.370	Histone H2B type 1-H	7	1.48842	4.89148	0.67134	3.26370

Q64518	20.82	22.71	11.750	Sarcoplasmic/endoplasmic reticulum calcium ATPase 3	10	1.21952	0.84543	0.81938	0.69227
Q64521	16.48	16.88	11.970	Glycerol-3-phosphate dehydrogenase, mitochondrial	9	1.09344	1.04338	0.91385	0.94571
Q64727	23.01	24	9.850	Vinculin	12	0.99804	1.14768	1.00120	1.15171
Q64735	1.83	1.91	2.070	Complement component receptor 1-like protein	2	1.13660	1.35839	0.87915	1.20696
Q66JS6	2.03	2.11	3.042	Eukaryotic translation initiation factor 3 subunit J-B	1	0.66841	0.67721	1.49496	1.01688
Q68FD5	33.18	37.1	10.330	Clathrin heavy chain 1	23	0.94139	0.94127	1.06145	1.00331
Q69ZF7	18.21	19.43	10.770	Metal transporter CNNM4	12	0.89656	0.67449	1.11452	0.76221
Q69ZN7	2.27	7.18	0.781	Myoferlin	2	1.05556	2.29229	0.94665	2.17927
Q6GQT9	12.39	14.08	4.695	Nodal modulator 1	5	1.27767	1.03469	0.78208	0.81853
Q6GSS7	6.79	6.85	26.920	Histone H2A type 2-A	4	1.28076	2.37001	0.78020	1.84357
Q6IRU2	4.39	6.68	10.080	Tropomyosin alpha-4 chain	4	0.97078	1.25678	1.02932	1.29927
Q6IRU5	3.68	3.74	8.297	Clathrin light chain B	3	0.89577	0.96980	1.11551	1.08661
Q6NZJ6	1.76	2.93	0.687	Eukaryotic translation initiation factor 4 gamma 1	1	1.16193	1.39725	0.85998	1.21257
Q6P069	2.4	2.4	9.596	Sorcin	2	0.75233	0.95624	1.32820	1.26965
Q6P5E4	9.24	11.12	2.515	UDP-glucose:glycoprotein glucosyltransferase 1	4	0.97885	0.92200	1.02083	0.94881
Q6PB66	7.19	12.67	2.586	Leucine-rich PPR motif-containing protein, mitochondrial	5	0.97087	0.83838	1.02923	0.86785
Q6PDQ2	1.59	2.65	0.470	Chromodomain-helicase-DNA-binding protein 4	1	1.10513	1.18258	0.90418	1.07400
Q6PHU5	4.4	5.92	2.303	Sortilin	2	0.99317	1.07883	1.00612	1.08262
Q6Q473	10.42	11.33	4.870	Calcium-activated chloride channel regulator 4	5	0.81561	0.65449	1.22515	0.80414
Q6R0H7	2	5.44	1.853	Guanine nucleotide-binding protein G(s) subunit alpha isoforms XLas	2	0.81709	1.18235	1.22294	1.45234
Q6URW6	35.4	54.12	13.200	Myosin-14	31	1.28105	1.00448	0.78002	0.79045
Q6X893	4.4	4.43	3.216	Choline transporter-like protein 1	2	0.94316	0.71136	1.05946	0.75676
Q6ZQ38	6.76	11.86	3.577	Cullin-associated NEDD8-dissociated protein 1	4	1.11170	1.16628	0.89885	1.05313
Q6ZQI3	3.76	4.17	5.155	Malectin	2	1.06667	1.00604	0.93679	0.94271
Q6ZQM8	1.54	5.03	4.520	UDP-glucuronosyltransferase 1-7C	4	1.80602	0.79998	0.55329	0.44987

Q6ZWX6	4.07	5.19	5.397	Eukaryotic translation initiation factor 2 subunit 1	2	0.67014	0.69935	1.49109	1.04828
Q76MZ3	7.4	7.7	4.584	Serine/threonine-protein phosphatase 2A 65 kDa regulatory subunit A alpha isoform	3	0.91248	0.94963	1.09508	1.04075
Q78KK3	1.52	4.37	3.448	Solute carrier family 22 member 18	3	1.44950	0.88312	0.68937	0.61149
Q78PY7	4.73	8.12	2.747	Staphylococcal nuclease domain-containing protein 1	3	1.16230	1.30857	0.85971	1.12218
Q7TMF3	3.7	5.16	31.720	NADH dehydrogenase [ubiquinone] 1 alpha subcomplex subunit 12	5	1.09068	0.93981	0.91616	0.86483
Q7TMK9	2.08	3.57	2.247	Heterogeneous nuclear ribonucleoprotein Q	2	1.39058	1.65843	0.71858	1.19726
Q7TN37	1.67	2.64	0.495	Transient receptor potential cation channel subfamily M member 4	1	0.98351	0.71970	1.01599	0.73210
Q7TNS2	1.55	1.55	10.530	Mitochondrial inner membrane organizing system protein 1	1	0.93494	1.00552	1.06878	1.07943
Q7TPR4	6.48	23.82	12.110	Alpha-actinin-1	14	1.06123	1.68464	0.94159	1.59266
Q7TST0	2.02	2.45	2.161	Butyrophilin-like protein 1	1	0.79740	0.37465	1.25313	0.47156
Q7TSV6	2	2.3	1.733	Putative aspartate aminotransferase, cytoplasmic 2	2	0.62839	0.54333	1.59016	0.86780
Q7TT45	2.03	4.17	2.004	Ras-related GTP-binding protein D	2	0.62065	0.90370	1.60998	1.46139
Q80SW1	6.04	6.3	6.415	Putative adenosylhomocysteinase 2	4	1.10092	1.05632	1.03759	1.10640
Q80TH2	7.29	7.84	1.854	Protein LAP2	3	0.91570	0.80317	1.09124	0.88202
Q80U72	5.79	6.23	2.233	Protein scribble homolog	3	1.02652	1.25798	0.97343	1.23266
Q80UG5	7.64	8.38	4.803	Septin-9	4	1.13855	1.78624	0.87765	1.61657
Q80V26	8.75	10.05	9.551	Inositol monophosphatase 3	4	0.95308	0.59175	1.04844	0.61999
Q80VA0	4.74	5.48	3.653	N-acetylgalactosaminyltransferase 7	2	1.22322	0.91663	0.81689	0.75390
Q80W54	2.69	2.88	1.684	CAAX prenyl protease 1 homolog	1	1.07069	1.10483	0.93327	1.03547
Q80X90	98.98	98.98	22.750	Filamin-B	54	1.09048	1.13271	0.91634	1.04512
Q80XN0	10.46	10.66	17.490	D-beta-hydroxybutyrate dehydrogenase, mitochondrial	6	1.11202	0.66815	0.89858	0.59541
Q80Y14	1.38	1.46	3.947	Glutaredoxin-related protein 5, mitochondrial	1	0.57898	0.48485	1.72587	0.86356
Q80YN3	1.95	2.3	1.106	Breast carcinoma-amplified sequence 1 homolog	1	0.86424	0.71581	1.15621	0.83360

Q80Z19	54.29	55.71	7.985	Mucin-2 (Fragments)	27	0.64778	0.34196	1.54257	0.52563
Q810Q5	6	6	19.280	Normal mucosa of esophagus-specific gene 1 protein	4	1.28563	0.62053	0.77724	0.48715
Q811D0	5.32	6.04	3.425	Disks large homolog 1	3	0.92103	0.84806	1.08491	0.92670
Q8BFR5	20.56	22.11	23.010	Elongation factor Tu, mitochondrial	13	1.09267	0.73320	0.91449	0.67933
Q8BFW7	2.42	2.51	1.468	Lipoma-preferred partner homolog	1	0.98673	1.40635	1.01269	1.43076
Q8BFZ9	8.57	8.72	16.470	Erlin-2	8	1.43706	1.07025	0.69534	0.74737
Q8BG05	7.3	8.03	12.660	Heterogeneous nuclear ribonucleoprotein A3	4	0.92107	0.39712	1.08488	0.43452
Q8BGC4	3.47	5.22	5.836	Zinc-binding alcohol dehydrogenase domain-containing protein 2	2	1.24784	0.78232	0.80078	0.63796
Q8BGD8	2	2	13.920	Cytochrome c oxidase assembly factor 6 homolog	1	1.19899	0.93912	0.83341	0.78613
Q8BGH2	2.12	2.8	2.559	Sorting and assembly machinery component 50 homolog	1	1.17455	0.94964	0.85074	0.81488
Q8BH24	6.24	7.04	6.221	Transmembrane 9 superfamily member 4	5	1.19059	0.97482	0.83929	0.82459
Q8BH79	2.04	4.23	2.428	Anoctamin-10	2	1.31649	1.31864	0.75902	1.00530
Q8BH95	18.6	19.66	37.590	Enoyl-CoA hydratase, mitochondrial	15	1.09436	0.56856	0.91308	0.52701
Q8BHG3	2.83	2.89	3.683	Cell cycle control protein 50B	2	0.94187	0.99629	1.06091	1.06249
Q8BHL4	2.71	2.8	3.652	Retinoic acid-induced protein 3	1	0.48118	0.30964	2.07665	0.64555
Q8BHN3	2.09	2.31	0.953	Neutral alpha-glucosidase AB	1	1.29359	1.30990	0.77246	1.00575
Q8BHY3	1.53	2.96	0.938	Anoctamin-1	1	1.06887	1.55979	0.93486	1.46463
Q8BI08	2	2	6.286	Protein MAL2	1	1.02089	0.88600	0.97880	0.87105
Q8BI84	2.21	4.44	0.725	Melanoma inhibitory activity protein 3	2	1.72920	1.31452	0.57786	0.76298
Q8BIJ6	1.49	2.85	0.988	Isoleucine--tRNA ligase, mitochondrial	1	0.90122	0.66107	1.10876	0.73621
Q8BJ64	4.73	5.13	4.027	Choline dehydrogenase, mitochondrial	3	1.07577	1.05149	0.92887	0.98232
Q8BK08	2	2	4.211	Transmembrane protein 11, mitochondrial	1	1.65608	1.39988	0.60338	0.84840
Q8BK30	2.6	2.65	16.350	NADH dehydrogenase [ubiquinone] flavoprotein 3, mitochondrial	2	0.88022	1.59639	1.13522	1.92955
Q8BK48	8.45	9.19	9.660	Pyrethroid hydrolase Ces2e	5	1.13748	0.47873	0.87848	0.43780

Q8BK64	3.31	3.83	6.213	Activator of 90 kDa heat shock protein ATPase homolog 1	2	1.00112	1.08882	0.99813	1.10289
Q8BKX1	2.64	2.93	2.804	Brain-specific angiogenesis inhibitor 1-associated protein 2	2	0.79801	1.32544	1.25216	1.66751
Q8BLF1	3.29	3.45	4.412	Neutral cholesterol ester hydrolase 1	2	1.09642	0.90236	0.91137	0.82448
Q8BM96	1.9	3.21	2.038	Probable G-protein coupled receptor 128	2	0.71987	0.58846	1.38810	0.82045
Q8BMD8	13.92	15.39	16.000	Calcium-binding mitochondrial carrier protein SCaMC-1	9	1.24986	0.96673	0.79949	0.76938
Q8BMF4	11.67	12.66	11.680	Dihydrolipoyllysine-residue acetyltransferase component of pyruvate dehydrogenase complex, mitochondrial	6	0.83391	0.59502	1.19827	0.72549
Q8BMK4	25.66	27.16	21.220	Cytoskeleton-associated protein 4	14	1.24753	1.19436	0.80098	0.96062
Q8BMP6	2.08	2.08	1.143	Golgi resident protein GCP60	1	1.04980	0.84119	0.95184	0.80422
Q8BMS1	17.28	18.4	8.650	Trifunctional enzyme subunit alpha, mitochondrial	10	1.21356	1.15115	0.82340	0.94719
Q8BN82	2	2.13	1.616	Sialin	1	1.21074	1.38875	0.82532	1.15123
Q8BP67	2.02	2.03	6.369	60S ribosomal protein L24	1	1.02957	1.56220	0.97054	1.52288
Q8BR63	4.14	4.17	13.040	Protein FAM177A1	3	1.00141	1.03101	0.99784	1.03474
Q8BRF7	2.96	4.74	3.756	Sec1 family domain-containing protein 1	3	0.85501	0.77742	1.16869	0.91581
Q8BT60	7.22	8.18	7.692	Copine-3	5	1.01975	1.35888	0.97989	1.33442
Q8BTM8	20.71	25.84	5.213	Filamin-A	12	1.16775	1.90311	0.85570	1.64141
Q8BTY2	5.01	5.67	2.805	Sodium bicarbonate cotransporter 3	3	0.96555	1.02411	1.03489	1.06451
Q8BTZ7	1.75	1.8	3.056	Mannose-1-phosphate guanyltransferase beta	1	0.85995	0.86005	1.16198	1.00518
Q8BU31	2.98	6.56	17.490	Ras-related protein Rap-2c	4	0.88675	1.04032	1.12686	1.18192
Q8BWM0	1.85	1.95	2.344	Prostaglandin E synthase 2	1	1.16598	0.95703	0.85700	0.82381
Q8BWT1	9.2	10.04	16.120	3-ketoacyl-CoA thiolase, mitochondrial	5	0.98984	0.83187	1.00950	0.90466
Q8BXK9	4.03	4.5	9.562	Chloride intracellular channel protein 5	2	0.56616	0.51714	1.76494	0.91660
Q8BXN9	7.37	10.36	8.468	Transmembrane protein 87A	5	1.06951	0.95916	0.93430	0.89715
Q8BY89	1.68	3.26	1.841	Choline transporter-like protein 2	2	1.05766	1.59668	0.94477	1.53593
Q8C0Z1	9.64	9.68	6.847	Protein ITFG3	5	0.78993	0.55957	1.26497	0.70858
Q8C102	4.93	6.49	2.258	Polypeptide N-acetylgalactosaminyltransferase 5	2	1.17730	0.73121	0.84876	0.62468

Q8C129	1.65	2.46	1.171	Leucyl-cystinyl aminopeptidase	1	1.22252	1.52010	0.81736	1.25632
Q8C165	2.08	2.27	2.584	Probable carboxypeptidase PM20D1	1	0.95611	0.66422	1.04511	0.69726
Q8C196	2.24	3.46	0.733	Carbamoyl-phosphate synthase [ammonia], mitochondrial	1	1.50262	1.09311	0.66500	0.73281
Q8C1B1	1.46	2.55	0.890	Calmodulin-regulated spectrin-associated protein 2	1	1.07499	0.66275	0.92954	0.61878
Q8C407	2	2.06	4.472	Protein YIPF4	1	1.11782	1.21477	0.89392	1.09071
Q8C522	2.04	2.43	1.996	Endonuclease domain-containing 1 protein	1	2.15115	2.03703	0.46452	0.95042
Q8CAQ8	25.8	27.87	17.970	Mitochondrial inner membrane protein	13	1.10864	0.93153	0.90132	0.83983
Q8CBW3	2	2.27	1.871	Abl interactor 1	1	0.69315	0.79291	1.44159	1.14811
Q8CC88	2.95	3.85	1.102	von Willebrand factor A domain-containing protein 8	2	1.15874	0.85061	0.86236	0.73677
Q8CCS6	2.05	3.52	2.318	Polyadenylate-binding protein 2	1	0.80034	0.73481	1.24853	0.92149
Q8CGK3	10.19	13.21	5.374	Lon protease homolog, mitochondrial	7	0.92371	0.62770	1.08177	0.69946
Q8CI51	1.8	1.82	1.523	PDZ and LIM domain protein 5	1	0.71987	1.15202	1.38809	1.60618
Q8CI85	2.28	2.28	2.542	Carbonic anhydrase 12	1	1.00311	0.55715	0.99614	0.57980
Q8JZQ2	5.07	5.85	2.494	AFG3-like protein 2	2	1.10392	1.08802	0.90518	0.98751
Q8K021	2.71	4.63	4.734	Secretory carrier-associated membrane protein 1	2	0.88065	0.83283	1.13467	0.94907
Q8K0C5	14.93	14.93	40.720	Zymogen granule membrane protein 16	15	0.68307	0.32437	1.51689	0.48470
Q8K0J2	8.33	8.49	10.080	UDP-GlcNAc:betaGal beta-1,3-N-acetylglucosaminyltransferase 7	5	0.90119	0.98160	1.10881	1.09339
Q8K135	3.75	4.91	1.622	Dyslexia-associated protein KIAA0319-like protein	2	0.90500	0.89130	1.10413	0.98955
Q8K1M6	2.26	4.06	2.830	Dynamin-1-like protein	3	0.90934	0.77522	1.09887	0.85697
Q8K2B3	18.53	18.63	14.310	Succinate dehydrogenase [ubiquinone] flavoprotein subunit, mitochondrial	9	1.23679	0.77795	0.80793	0.62972
Q8K353	2	2	9.615	Cysteine-rich and transmembrane domain-containing protein 1	2	0.80154	0.66454	1.24665	0.83212
Q8K3J1	5.18	5.18	12.740	NADH dehydrogenase [ubiquinone] iron-sulfur protein 8, mitochondrial	4	1.15530	0.82659	0.86492	0.71813
Q8K419	27.09	27.85	40.490	Galectin-4	45	1.19467	1.64138	0.83641	1.37331

Q8K4L4	2.23	3.06	1.704	Protein POF1B	1	1.15502	0.97410	0.86513	0.84925
Q8QZR3	1.8	5.89	3.763	Pyrethroid hydrolase Ces2a	3	1.11754	0.45240	0.89415	0.40630
Q8QZT1	10.01	10.65	10.850	Acetyl-CoA acetyltransferase, mitochondrial	5	1.02701	0.66184	0.97297	0.65043
Q8R059	4.67	4.81	8.069	UDP-glucose 4-epimerase	2	0.85044	0.97025	1.17497	1.14551
Q8R081	6.12	6.25	4.778	Heterogeneous nuclear ribonucleoprotein L	3	1.08241	0.69717	0.95974	0.67233
Q8R088	1.66	1.75	5.263	Golgi phosphoprotein 3-like	1	0.76115	0.60760	1.31281	0.79400
Q8R0W0	4.04	9.36	2.871	Epiplakin	5	1.04344	0.92019	0.95764	0.89368
Q8R143	2.01	2.28	5.172	Pituitary tumor-transforming gene 1 protein-interacting protein	1	0.95210	1.15640	1.04951	1.21902
Q8R2S8	11.89	11.98	10.530	CD177 antigen	9	1.07411	1.67507	0.93030	1.59718
Q8R3B1	6.06	6.7	5.688	1-phosphatidylinositol 4,5-bisphosphate phosphodiesterase delta-1	3	0.77036	0.60180	1.29710	0.78503
Q8R3G9	10	10.13	19.570	Tetraspanin-8	9	0.77046	0.57752	1.29694	0.75191
Q8R4D1	1.35	2.5	1.042	Sodium/hydrogen exchanger 8	1	0.89048	0.77125	1.12215	0.88438
Q8R4G6	1.57	3.76	1.081	Alpha-1,6-mannosylglycoprotein 6-beta-N-acetylglucosaminyltransferase A	1	0.84987	0.93007	1.17577	1.10044
Q8R4N0	2.88	3	3.550	Citrate lyase subunit beta-like protein, mitochondrial	1	0.76460	0.45538	1.30688	0.59497
Q8VBT0	1.85	1.85	3.237	Thioredoxin-related transmembrane protein 1	1	1.00742	1.41843	0.99188	1.41315
Q8VCA5	2.09	4.52	3.908	Transmembrane protease serine 4	2	1.16140	1.75416	0.86038	1.51591
Q8VCC2	5.67	5.86	4.956	Liver carboxylesterase 1	3	1.19081	0.56612	0.83913	0.49254
Q8VCF1	8.04	8.05	13.150	Soluble calcium-activated nucleotidase 1	4	1.07723	0.88301	0.92760	0.82111
Q8VCR7	2.25	2.25	5.238	Alpha/beta hydrolase domain-containing protein 14B	1	0.82816	0.68600	1.20659	0.83120
Q8VCS3	1.63	1.91	4.890	Glycosaminoglycan xylosylkinase	2	0.92654	0.95474	1.07846	1.02049
Q8VCT4	2.02	3.83	3.717	Carboxylesterase 1D	2	0.91722	0.65783	1.08942	0.71838
Q8VCW8	8.58	10.61	6.667	Acyl-CoA synthetase family member 2, mitochondrial	4	0.85374	0.58001	1.17043	0.68208
Q8VDD5	63.87	66.26	16.280	Myosin-9	38	1.21667	1.65252	0.82129	1.38510
Q8VDJ3	2.15	3.49	0.710	Vigilin	1	1.09219	1.26047	0.91490	1.16318

Q8VDM4	4.95	5.11	1.872	26S proteasome non-ATPase regulatory subunit 2	2	0.88106	0.80521	1.13413	0.91525
Q8VDN2	89.85	89.85	41.540	Sodium/potassium-transporting ATPase subunit alpha-1	112	0.75774	0.43280	1.31872	0.56349
Q8VDW0	3.52	3.67	3.981	ATP-dependent RNA helicase DDX39A	4	1.38671	1.18302	0.72059	0.86013
Q8VED5	2	12.51	7.533	Keratin, type II cytoskeletal 79	11	0.46789	0.76919	2.13562	1.64997
Q8VEK3	2.83	3.35	1.250	Heterogeneous nuclear ribonucleoprotein U	1	1.07639	1.09670	0.92833	1.02594
Q8VEM8	3.2	3.42	5.042	Phosphate carrier protein, mitochondrial	2	1.33751	1.74124	0.74709	1.30662
Q8VHF2	8.9	9.45	7.702	Cadherin-related family member 5	8	0.48503	0.36972	2.06017	0.76412
Q8VIJ6	6.58	7.09	4.578	Splicing factor, proline- and glutamine-rich	3	1.16629	1.23254	0.85677	1.05893
Q91V41	9.15	12.17	30.700	Ras-related protein Rab-14	8	0.95507	0.65364	1.04625	0.69333
Q91V61	2.04	2.13	4.050	Sideroflexin-3	1	0.93163	0.98907	1.07258	1.06189
Q91V76	2.01	2.11	3.810	Ester hydrolase C11orf54 homolog	1	0.56068	0.21570	1.78221	0.38612
Q91V92	2.15	2.92	0.733	ATP-citrate synthase	1	0.73809	1.01905	1.35383	1.38572
Q91VA1	3.56	5.31	3.253	Choline transporter-like protein 4	3	0.52103	0.39489	1.91781	0.76067
Q91VD9	20.97	22.8	14.720	NADH-ubiquinone oxidoreductase 75 kDa subunit, mitochondrial	11	1.24254	0.82323	0.80419	0.66576
Q91VM9	3.38	3.42	7.879	Inorganic pyrophosphatase 2, mitochondrial	2	0.73739	0.64286	1.35511	0.87581
Q91VR2	12.63	13.1	20.470	ATP synthase subunit gamma, mitochondrial	7	1.28122	1.23698	0.77992	0.96925
Q91VR8	2	2.02	14.670	Protein BRICK1	1	1.18042	1.63079	0.84652	1.38660
Q91VS7	2.02	2.06	8.387	Microsomal glutathione S-transferase 1	1	1.39121	1.10196	0.71826	0.79499
Q91W90	7.58	7.97	15.830	Thioredoxin domain-containing protein 5	7	0.88484	0.63323	1.12929	0.71801
Q91WD5	8.56	9.37	9.287	NADH dehydrogenase [ubiquinone] iron-sulfur protein 2, mitochondrial	5	1.02950	1.00552	0.97061	0.98243
Q91WG0	5.92	8.71	9.804	Acylcarnitine hydrolase	5	1.32563	0.37942	0.75379	0.29844
Q91WG8	4.16	4.39	3.047	Bifunctional UDP-N-acetylglucosamine 2-epimerase/N-acetylmannosamine kinase	2	1.11354	0.94440	0.89736	0.85288

Q91WL0	3.73	4.4	3.000	Epidermal growth factor receptor kinase substrate 8-like protein 3	2	0.63438	0.92162	1.57515	1.45643
Q91WQ3	2.28	3.46	1.705	Tyrosine--tRNA ligase, cytoplasmic	1	0.73592	0.71071	1.35782	0.97086
Q91WQ9	3.24	3.35	14.380	Calmodulin-like protein 4	2	0.57405	0.51324	1.74070	0.89736
Q91WT9	2	2.04	1.783	Cystathionine beta-synthase	1	0.87312	0.38371	1.14445	0.44108
Q91X52	2.05	2.23	3.279	L-xylulose reductase	1	0.92656	1.00979	1.07844	1.07838
Q91XA2	13.75	14.63	22.390	Golgi membrane protein 1	15	1.02620	0.71312	0.97374	0.69510
Q91Y74	3.79	4.51	7.207	CMP-N-acetylneuraminate-beta-galactosamide-alpha-2,3-sialyltransferase 4	3	0.85069	0.65251	1.17463	0.77109
Q91Y97	1.77	4.81	3.846	Fructose-bisphosphate aldolase B	2	0.87128	0.65345	1.14687	0.75306
Q91YD6	1.85	2.16	1.164	Villin-like protein	1	0.79523	1.53247	1.25655	1.93413
Q91YQ5	29.9	30.78	28.780	Dolichyl-diphosphooligosaccharide--protein glycosyltransferase subunit 1	18	1.57222	1.06913	0.63556	0.66696
Q91YT0	6.26	10.74	9.052	NADH dehydrogenase [ubiquinone] flavoprotein 1, mitochondrial	5	1.12009	1.03339	0.89211	0.92719
Q91YT8	2	2.56	1.493	Transmembrane protein 63A	1	0.62019	0.60963	1.61120	0.98659
Q91YW3	4.85	6.78	3.770	DnaJ homolog subfamily C member 3	2	1.12171	0.85719	0.89082	0.74735
Q91ZA3	1.32	1.5	1.243	Propionyl-CoA carboxylase alpha chain, mitochondrial	1	0.96805	0.88121	1.03223	0.91363
Q91ZJ5	2.93	3.24	3.346	UTP--glucose-1-phosphate uridylyltransferase	2	0.67975	0.63418	1.47001	0.93642
Q91ZN5	2.69	3.25	4.408	Adenosine 3'-phospho 5'-phosphosulfate transporter 1	2	1.08135	0.78156	0.92407	0.72541
Q921F2	2	2.43	2.657	TAR DNA-binding protein 43	1	1.16062	1.08413	0.86096	0.93752
Q921H8	6.27	6.36	8.726	3-ketoacyl-CoA thiolase A, peroxisomal	4	1.25228	0.74955	0.88041	0.66787
Q921I1	4.43	5.13	2.869	Serotransferrin	2	0.69082	0.98016	1.44646	1.39076
Q921X9	8.24	8.38	6.963	Protein disulfide-isomerase A5	5	1.04242	0.90584	0.95858	0.87290
Q922B2	6.02	6.47	6.387	Aspartate--tRNA ligase, cytoplasmic	3	0.95372	1.02867	1.04773	1.08257
Q922D8	2.15	2.69	1.925	C-1-tetrahydrofolate synthase, cytoplasmic	2	0.95727	1.04728	1.04384	1.10443

Q922Q1	2.62	2.88	4.734	MOSC domain-containing protein 2, mitochondrial	2	1.22224	1.32709	0.81755	1.08979
Q922Q8	4.33	4.82	7.166	Leucine-rich repeat-containing protein 59	3	1.29204	1.47232	0.77338	1.13960
Q922R8	18.17	18.68	20.680	Protein disulfide-isomerase A6	10	1.26500	0.82998	0.78992	0.65359
Q922U2	2.87	13.94	8.621	Keratin, type II cytoskeletal 5	16	0.45932	0.64914	2.17547	1.41898
Q93092	3.98	4.59	6.231	Transaldolase	2	0.86774	0.97190	1.15155	1.12644
Q99J99	6.35	6.47	11.780	3-mercaptopyruvate sulfurtransferase	3	0.82189	0.44982	1.21578	0.55089
Q99JB2	2.08	3.12	3.966	Stomatin-like protein 2, mitochondrial	1	1.18561	1.20336	0.84281	1.01870
Q99JG3	3.88	7.15	7.886	Annexin A13	4	0.64754	0.74149	1.54315	1.14903
Q99JI6	6.81	7.14	13.040	Ras-related protein Rap-1b	3	0.79625	1.18374	1.25493	1.51199
Q99JR1	2.03	2.24	4.037	Sideroflexin-1	2	0.99721	0.80485	1.00204	0.81396
Q99JW5	15.97	17.14	25.080	Epithelial cell adhesion molecule	12	1.15490	0.99144	0.86522	0.77932
Q99JX3	3.43	3.71	4.213	Golgi reassembly-stacking protein 2	2	1.08161	0.92788	0.92385	0.86375
Q99JY0	18.79	19.09	18.320	Trifunctional enzyme subunit beta, mitochondrial	11	1.24203	0.98123	0.80453	0.78932
Q99JY9	7.45	7.83	15.310	Actin-related protein 3	7	0.99055	0.94754	1.00877	0.96018
Q99K01	6.52	9.42	6.480	Pyridoxal-dependent decarboxylase domain-containing protein 1	5	1.01865	0.79051	0.98095	0.78017
Q99K23	2.16	2.36	1.735	Ufm1-specific protease 2	1	1.38924	0.91961	0.71927	0.66438
Q99K30	4.07	4.8	3.292	Epidermal growth factor receptor kinase substrate 8-like protein 2	2	0.64376	0.80674	1.55220	1.25856
Q99KF1	5.22	6.04	15.740	Transmembrane emp24 domain-containing protein 9	7	1.27342	0.85342	0.78469	0.67479
Q99KI0	33.96	36.64	22.050	Aconitate hydratase, mitochondrial	22	0.97358	0.77472	1.02636	0.80744
Q99KJ8	4.04	4.09	6.219	Dynactin subunit 2	2	1.05116	1.15123	0.95061	1.10167
Q99KN9	6	6.12	5.705	Clathrin interactor 1	3	0.94390	0.82801	1.05863	0.87943
Q99KQ4	1.38	1.87	1.426	Nicotinamide phosphoribosyltransferase	1	0.86107	1.33252	1.16047	1.55320
Q99KV1	5.64	5.68	9.218	DnaJ homolog subfamily B member 11	4	1.35674	0.93347	0.73650	0.69720
Q99L04	3.6	3.65	6.070	Dehydrogenase/reductase SDR family member 1	3	1.09974	1.11442	0.90861	1.02270

Q99L47	3.01	3.06	4.582	Hsc70-interacting protein	2	0.67278	0.99123	1.48525	1.48022
Q99LB2	11.2	11.79	18.280	Dehydrogenase/reductase SDR family member 4	6	1.23564	1.18632	0.80869	0.96218
Q99LC3	2.15	2.24	7.324	NADH dehydrogenase [ubiquinone] 1 alpha subcomplex subunit 10, mitochondrial	2	1.25989	1.34152	0.79312	1.06399
Q99LC5	15.39	16.4	29.430	Electron transfer flavoprotein subunit alpha, mitochondrial	10	0.98243	0.47575	1.01712	0.48900
Q99LM2	2.04	2.68	2.783	CDK5 regulatory subunit-associated protein 3	1	0.64324	0.47137	1.55344	0.73548
Q99LP6	3.57	4.93	9.677	GrpE protein homolog 1, mitochondrial	3	0.85349	0.73131	1.17077	0.86342
Q99LR1	1.73	1.81	2.010	Monoacylglycerol lipase ABHD12	1	1.57684	2.27504	0.63370	1.44808
Q99LX0	4.15	4.6	11.110	Protein DJ-1	2	0.75399	1.21667	1.32527	1.62013
Q99M74	2	3.71	4.651	Keratin, type II cuticular Hb2	4	2.95955	0.88773	0.33763	0.30105
Q99P72	4.75	5.76	2.754	Reticulon-4	2	3.66679	5.06606	0.27251	1.72836
Q99PL5	16.42	24.11	5.109	Ribosome-binding protein 1	10	1.18068	1.10526	0.84633	0.93603
Q99PT1	4.26	5.43	17.160	Rho GDP-dissociation inhibitor 1	3	0.76330	0.74414	1.30912	0.97959
Q9CPP6	2.18	2.87	13.790	NADH dehydrogenase [ubiquinone] 1 alpha subcomplex subunit 5	2	1.24955	0.74131	0.79968	0.60095
Q9CPQ1	6.85	6.92	27.630	Cytochrome c oxidase subunit 6C	4	1.04720	0.70801	0.95420	0.68568
Q9CPQ8	4.07	4.23	31.070	ATP synthase subunit g, mitochondrial	3	1.01337	1.03165	0.98606	1.01604
Q9CPT4	3.12	3.17	10.840	UPF0556 protein C19orf10 homolog	2	1.17263	0.88551	0.85214	0.76431
Q9CPW4	2.9	3.29	9.934	Actin-related protein 2/3 complex subunit 5	2	1.31883	2.51018	0.75768	1.91032
Q9CPY7	4.19	7.08	4.624	Cytosol aminopeptidase	4	0.91241	0.82613	1.09517	0.91911
Q9CQ10	2.01	2.6	3.571	Charged multivesicular body protein 3	1	0.92240	1.07638	1.08331	1.17121
Q9CQ22	2	2.07	8.075	Ragulator complex protein LAMTOR1	2	0.88644	1.05402	1.12726	1.19341
Q9CQ54	2.07	2.07	7.500	NADH dehydrogenase [ubiquinone] 1 subunit C2	1	0.94328	1.17096	1.05933	1.23816
Q9CQ60	2	2.46	6.226	6-phosphogluconolactonase	1	0.76958	0.65548	1.29843	0.85486
Q9CQ62	6.9	7.28	8.657	2,4-dienoyl-CoA reductase, mitochondrial	4	1.12683	0.83849	0.88677	0.74792

Q9CQ75	2.96	3.32	16.160	NADH dehydrogenase [ubiquinone] 1 alpha subcomplex subunit 2	2	0.89917	0.64082	1.11130	0.71342
Q9CQA3	16.02	17.34	23.400	Succinate dehydrogenase [ubiquinone] iron-sulfur subunit, mitochondrial	7	1.08176	0.82671	0.92372	0.75617
Q9CQC6	2.12	5.76	3.580	Basic leucine zipper and W2 domain-containing protein 1	2	1.17305	1.26381	0.85183	1.08127
Q9CQC7	2.01	2.03	8.527	NADH dehydrogenase [ubiquinone] 1 beta subcomplex subunit 4	1	1.21887	1.01187	0.81981	0.83321
Q9CQD1	2	8.2	20.470	Ras-related protein Rab-5A	5	1.07324	0.77476	0.93105	0.72454
Q9CQE8	2.1	2.25	2.869	UPF0568 protein C14orf166 homolog	1	0.76752	0.66191	1.30191	0.86197
Q9CQF9	2.02	3.51	3.366	Prenylcysteine oxidase	2	1.00297	0.88075	0.99629	0.88077
Q9CQH3	3.87	3.92	10.050	NADH dehydrogenase [ubiquinone] 1 beta subcomplex subunit 5, mitochondrial	2	1.23002	1.05068	0.81238	0.85733
Q9CQI3	2.77	2.77	4.930	Glia maturation factor beta	1	0.92971	1.00426	1.07479	1.08442
Q9CQJ8	2.16	2.42	5.028	NADH dehydrogenase [ubiquinone] 1 beta subcomplex subunit 9	1	1.34607	0.99322	0.74234	0.74125
Q9CQM5	3.24	3.24	15.450	Thioredoxin domain-containing protein 17	2	0.71736	0.69582	1.39295	0.96902
Q9CQN1	3.9	6.8	4.391	Heat shock protein 75 kDa, mitochondrial	4	1.24800	0.80683	0.80067	0.65794
Q9CQQ7	8.6	9.05	16.800	ATP synthase F(0) complex subunit B1, mitochondrial	6	0.99924	0.96013	1.00000	0.97159
Q9CQR2	2.09	2.79	8.434	40S ribosomal protein S21	1	1.16076	1.40943	0.86085	1.23537
Q9CQS8	1.77	1.81	10.420	Protein transport protein Sec61 subunit beta	1	1.42247	1.10316	0.70247	0.77836
Q9CQU0	2.04	2.04	5.294	Thioredoxin domain-containing protein 12	1	1.14804	0.88235	0.87039	0.77139
Q9CQV8	2.32	16.62	28.050	14-3-3 protein beta/alpha	11	0.76371	0.99588	1.30841	1.31081
Q9CQW2	1.67	1.79	4.839	ADP-ribosylation factor-like protein 8B	2	0.97780	1.40965	1.02193	1.43639
Q9CQX2	6	6.93	21.230	Cytochrome b5 type B	3	1.45444	0.80751	0.68703	0.55912
Q9CQX5	1.57	1.57	3.557	Claudin domain-containing protein 1	1	0.89343	0.85232	1.11844	0.95748

Q9CQZ5	4.05	5.47	22.900	NADH dehydrogenase [ubiquinone] 1 alpha subcomplex subunit 6	4	1.10480	0.91868	0.90446	0.83404
Q9CR21	5.58	5.64	18.590	Acyl carrier protein, mitochondrial	6	0.98471	0.89577	1.01476	0.90622
Q9CR26	1.36	1.57	2.589	Vacuolar protein sorting-associated protein VTA1 homolog	1	0.97526	1.21303	1.02459	1.24836
Q9CR57	2.62	2.7	5.991	60S ribosomal protein L14	1	0.78263	1.25209	1.27677	1.60160
Q9CR61	4	4.05	16.790	NADH dehydrogenase [ubiquinone] 1 beta subcomplex subunit 7	2	1.06410	1.00495	0.93905	0.94754
Q9CR67	2	2.06	4.858	Transmembrane protein 33	1	1.46974	2.31036	0.67988	1.57771
Q9CR68	12.39	12.65	22.260	Cytochrome b-c1 complex subunit Rieske, mitochondrial	10	1.12794	0.73299	0.88590	0.65267
Q9CRB9	6.93	7.04	14.100	Coiled-coil-helix-coiled-coil-helix domain-containing protein 3, mitochondrial	4	1.17392	0.90928	0.85121	0.79654
Q9CVB6	14.07	15.21	21.330	Actin-related protein 2/3 complex subunit 2	10	1.04200	1.21524	0.95896	1.17198
Q9CWJ9	2.15	2.53	1.689	Bifunctional purine biosynthesis protein PURH	1	0.60333	0.69661	1.65622	1.14262
Q9CWS0	2.82	2.97	2.807	N(G),N(G)-dimethylarginine dimethylaminohydrolase 1	1	0.88367	1.05562	1.13079	1.19696
Q9CWZ3	2.55	2.56	6.322	RNA-binding protein 8A	1	1.23247	1.22751	0.81077	1.00064
Q9CWZ7	1.93	2.51	3.205	Gamma-soluble NSF attachment protein	2	0.96149	0.96450	1.03927	0.99826
Q9CX00	4.19	4.6	4.696	IST1 homolog	2	0.91360	1.16051	1.09374	1.27053
Q9CXI5	8.05	8.48	24.020	Mesencephalic astrocyte-derived neurotrophic factor	5	1.18747	1.15480	0.84149	0.97216
Q9CXW3	1.49	1.62	3.057	Calcyclin-binding protein	1	1.13214	1.31431	0.88262	1.16516
Q9CXW4	4.11	5.38	15.730	60S ribosomal protein L11	3	1.06720	1.40308	0.93632	1.31755
Q9CXZ1	6.38	6.47	16.570	NADH dehydrogenase [ubiquinone] iron-sulfur protein 4, mitochondrial	3	1.20986	0.68665	0.82592	0.57190
Q9CY50	4.01	4.02	6.643	Translocon-associated protein subunit alpha	3	1.75438	1.13268	0.56957	0.65559
Q9CYN2	5.15	5.34	9.735	Signal peptidase complex subunit 2	3	1.51225	1.30407	0.66077	0.86854
Q9CYR0	2.05	2.05	5.263	Single-stranded DNA-binding protein, mitochondrial	1	1.22097	1.37161	0.81840	1.12749

Q9CYW4	2	2.08	3.187	Haloacid dehalogenase-like hydrolase domain-containing protein 3	1	0.54009	0.44673	1.85013	0.83016
Q9CYZ2	6.93	7.09	21.820	Tumor protein D54	4	1.13101	1.01097	0.88350	0.89772
Q9CZ13	19.15	20.83	18.750	Cytochrome b-c1 complex subunit 1, mitochondrial	10	1.33211	0.72030	0.75012	0.54624
Q9CZ30	3.5	4.04	5.051	Obg-like ATPase 1	2	1.13521	1.09370	0.88023	0.97239
Q9CZ42	2.18	2.18	2.624	ATP-dependent (S)-NAD(P)H-hydrate dehydratase	1	0.84798	0.79585	1.17838	0.94383
Q9CZB0	2.01	2.05	7.692	Succinate dehydrogenase cytochrome b560 subunit, mitochondrial	1	1.26877	0.97035	0.78757	0.76760
Q9CZD3	2	2.13	1.509	Glycine--tRNA ligase	1	1.15015	1.20089	0.86879	1.04793
Q9CZG9	2.85	4.78	17.860	PDZ domain-containing protein 11	3	1.22592	0.65261	0.81510	0.53429
Q9CZS1	15.33	18.9	15.610	Aldehyde dehydrogenase X, mitochondrial	9	0.77070	0.36559	1.29655	0.47307
Q9CZU6	15.56	16.47	15.730	Citrate synthase, mitochondrial	10	1.02035	0.90228	0.97931	0.88327
Q9CZX8	3.78	4.76	13.100	40S ribosomal protein S19	2	0.87601	1.21148	1.14067	1.39307
Q9CZY3	3.58	3.77	11.560	Ubiquitin-conjugating enzyme E2 variant 1	2	0.93217	1.21801	1.07195	1.30181
Q9D051	5.74	8.29	8.914	Pyruvate dehydrogenase E1 component subunit beta, mitochondrial	4	0.91299	0.48205	1.09448	0.53209
Q9D0E1	5.31	5.89	2.469	Heterogeneous nuclear ribonucleoprotein M	3	1.05955	0.86144	0.94308	0.81165
Q9D0F3	1.91	2.5	1.934	Protein ERGIC-53	1	1.43254	1.12045	0.69753	0.78899
Q9D0I9	2.83	3.91	1.212	Arginine--tRNA ligase, cytoplasmic	1	1.06192	0.98079	0.94098	0.92695
Q9D0J8	2	2.04	10.890	Parathymosin	1	1.14612	1.69590	0.87185	1.48511
Q9D0K2	5.5	5.66	3.462	Succinyl-CoA:3-ketoacid coenzyme A transferase 1, mitochondrial	2	0.98037	0.70667	1.01926	0.72581
Q9D0M3	4.99	5.07	8.923	Cytochrome c1, heme protein, mitochondrial	2	1.20187	1.03049	0.83141	0.86049
Q9D154	4.78	7.22	11.610	Leukocyte elastase inhibitor A	4	0.84689	0.58012	1.17989	0.67767
Q9D1A2	2	2.08	2.316	Cytosolic non-specific dipeptidase	1	0.70289	0.85502	1.42163	1.22090
Q9D1D4	8	10.83	17.810	Transmembrane emp24 domain-containing protein 10	7	1.63750	1.02369	0.61022	0.65062

Q9D1G1	2	11.97	31.340	Ras-related protein Rab-1B	7	1.06317	0.86617	0.93987	0.81769
Q9D1G5	2	2.65	4.603	Leucine-rich repeat-containing protein 57	1	0.73918	1.04135	1.35184	1.41396
Q9D1M7	2.03	2.07	9.950	Peptidyl-prolyl cis-trans isomerase FKBP11	2	1.35539	0.81644	0.73724	0.60457
Q9D1Q6	7.46	8.13	8.621	Endoplasmic reticulum resident protein 44	4	1.06649	0.72833	0.93694	0.68620
Q9D1R9	1.96	2	5.983	60S ribosomal protein L34	1	1.12781	1.01607	0.88601	0.90423
Q9D279	15.52	15.9	12.810	Mitotic interactor and substrate of PLK1	9	0.73586	0.61046	1.35793	0.83456
Q9D2G2	5.95	7.7	5.507	Dihydrolipoyllysine-residue succinyltransferase component of 2-oxoglutarate dehydrogenase complex, mitochondrial	3	0.97949	0.69122	1.02017	0.71071
Q9D2Q8	9.74	9.74	48.080	Protein S100-A14	10	1.64220	3.28264	0.60848	2.00010
Q9D306	6.42	6.68	6.485	Alpha-1,3-mannosyl-glycoprotein 4-beta-N-acetylglucosaminyltransferase C	5	1.10028	0.67423	0.90817	0.61897
Q9D309	6.25	6.29	17.450	Protein FAM3B	5	1.34317	0.78505	0.74394	0.58794
Q9D312	40.78	43.8	50.120	Keratin, type I cytoskeletal 20	43	1.26903	0.55697	0.78741	0.44603
Q9D3D9	7.5	7.51	26.790	ATP synthase subunit delta, mitochondrial	7	1.19358	0.93061	0.83718	0.78315
Q9D3P8	2	2.14	6.803	Plasminogen receptor (KT)	1	1.00098	0.72330	0.99827	0.72524
Q9D6J6	6.74	7.18	16.940	NADH dehydrogenase [ubiquinone] flavoprotein 2, mitochondrial	6	0.99703	0.78502	1.00222	0.79169
Q9D6R2	15.02	15.25	19.950	Isocitrate dehydrogenase [NAD] subunit alpha, mitochondrial	8	0.83783	0.58350	1.19266	0.70031
Q9D6Y7	2.12	2.13	3.863	Mitochondrial peptide methionine sulfoxide reductase	1	0.70098	0.76616	1.42550	1.09767
Q9D7A8	2	2.09	4.255	Armadillo repeat-containing protein 1	1	0.95370	1.17195	1.04776	1.23335
Q9D7L8	1.75	1.86	3.065	Transmembrane and immunoglobulin domain-containing protein 1	1	0.66380	0.51893	1.50535	0.78463
Q9D7N9	6.69	7.17	9.880	Adipocyte plasma membrane-associated protein	4	1.14069	0.91253	0.87600	0.80470
Q9D7S0	3.26	4.02	5.882	Ly6/PLAUR domain-containing protein 8	4	0.62171	0.40278	1.60726	0.65135
Q9D7T1	2.46	2.64	4.348	Rab15 effector protein	2	0.92273	0.87637	1.08292	0.94853
Q9D7Z6	29.61	30.53	19.610	Calcium-activated chloride channel regulator 1	19	0.62244	0.40829	1.60538	0.64212

Q9D816	23.13	23.97	22.860	Cytochrome P450 2C55	14	1.80151	0.40819	0.55467	0.25466
Q9D819	7.95	8.34	18.690	Inorganic pyrophosphatase	5	0.83342	0.68310	1.19896	0.84065
Q9D855	11.05	11.05	46.850	Cytochrome b-c1 complex subunit 7	7	1.15471	0.67724	0.86536	0.59202
Q9D869	2.38	2.42	9.184	Calcineurin B homologous protein 2	2	0.61062	0.42355	1.63643	0.69589
Q9D880	2	2.08	3.116	Mitochondrial import inner membrane translocase subunit TIM50	1	1.02593	1.14126	0.97399	1.11649
Q9D898	2.02	2.05	7.843	Actin-related protein 2/3 complex subunit 5-like protein	1	0.96046	0.99687	1.04039	1.04288
Q9D8B3	5.91	7.29	13.390	Charged multivesicular body protein 4b	4	1.13367	1.48878	0.88142	1.31826
Q9D8E6	2.05	2.49	5.012	60S ribosomal protein L4	3	1.10460	1.42867	0.90462	1.29812
Q9D8N0	6.01	6.16	6.636	Elongation factor 1-gamma	5	1.21006	0.99151	0.82578	0.81814
Q9D8T2	2.7	2.93	2.053	Gasdermin-D	1	1.12335	1.04576	0.88952	0.92802
Q9D8V0	5.56	7.29	7.672	Minor histocompatibility antigen H13	5	1.32341	1.47187	0.75505	1.11672
Q9D8W7	2.48	2.49	11.040	OCIA domain-containing protein 2	2	1.19608	2.19100	0.83543	1.89121
Q9D8Y0	4.69	5.04	10.420	EF-hand domain-containing protein D2	3	0.75549	1.07875	1.32265	1.43641
Q9DAS9	4.17	4.17	37.500	Guanine nucleotide-binding protein G(I)/G(S)/G(O) subunit gamma-12	4	0.77691	0.72220	1.28618	0.93231
Q9DB05	9.06	9.07	17.970	Alpha-soluble NSF attachment protein	6	1.11157	1.03437	0.89895	0.93463
Q9DB20	10.23	10.47	24.880	ATP synthase subunit O, mitochondrial	8	1.20439	1.23565	0.82967	1.03785
Q9DB34	4.04	4.77	12.160	Charged multivesicular body protein 2a	2	1.06768	1.11944	0.93590	1.05477
Q9DB77	27.94	27.94	38.410	Cytochrome b-c1 complex subunit 2, mitochondrial	22	1.73130	1.20632	0.57716	0.69165
Q9DBG3	6.58	25.26	10.460	AP-2 complex subunit beta	12	1.01148	1.42927	0.98790	1.43343
Q9DBG5	1.56	1.6	1.831	Perilipin-3	1	1.06048	0.99213	0.94226	0.93719
Q9DBG6	10.06	10.14	9.984	Dolichyl-diphosphooligosaccharide--protein glycosyltransferase subunit 2	8	1.57727	1.38150	0.63353	0.87909
Q9DBG7	2.07	2.92	1.415	Signal recognition particle receptor subunit alpha	1	1.56001	2.97729	0.64054	1.91550
Q9DBG9	2.85	3.85	22.580	Tax1-binding protein 3	3	1.01890	1.54660	0.98071	1.52515

Q9DBH5	5.07	5.24	8.939	Vesicular integral-membrane protein VIP36	3	1.08186	0.92331	0.92364	0.85687
Q9DBJ1	2.12	3.12	6.299	Phosphoglycerate mutase 1	3	0.79918	1.24795	1.25033	1.57185
Q9DBJ3	4.13	4.58	3.696	Brain-specific angiogenesis inhibitor 1-associated protein 2-like protein 1	2	0.68656	0.85909	1.45543	1.25911
Q9DBM2	2.05	2.36	2.089	Peroxisomal bifunctional enzyme	1	2.23257	1.29305	0.72773	0.94516
Q9DBS1	2	2.28	2.500	Transmembrane protein 43	1	1.33419	1.63280	0.74895	1.22829
Q9DBU0	1.46	2.16	1.980	Transmembrane 9 superfamily member 1	1	1.02019	0.79565	0.97946	0.78169
Q9DBX6	2.09	2.57	3.992	Cytochrome P450 2S1	2	1.05915	0.92694	0.94344	0.87591
Q9DC23	4.42	4.79	2.648	DnaJ homolog subfamily C member 10	3	1.34318	1.19971	0.74394	0.89708
Q9DC29	4.08	7.25	3.444	ATP-binding cassette sub-family B member 6, mitochondrial	3	1.10575	1.05737	0.90368	0.95793
Q9DC51	4.07	13.71	20.060	Guanine nucleotide-binding protein G(k) subunit alpha	9	0.91982	0.81587	1.08634	0.88627
Q9DCD0	3.61	3.95	6.211	6-phosphogluconate dehydrogenase, decarboxylating	3	0.75720	1.15294	1.31966	1.52937
Q9DCH4	4.51	4.6	8.587	Eukaryotic translation initiation factor 3 subunit F	3	0.84576	0.68657	1.18148	0.81541
Q9DCM0	3.89	4.26	7.087	Persulfide dioxygenase ETHE1, mitochondrial	2	0.64649	0.19680	1.54564	0.31288
Q9DCS3	4.41	4.84	6.166	Trans-2-enoyl-CoA reductase, mitochondrial	2	0.68101	0.47351	1.46730	0.69753
Q9DCS9	1.89	2.2	3.977	NADH dehydrogenase [ubiquinone] 1 beta subcomplex subunit 10	1	0.92387	0.75742	1.08158	0.81852
Q9DCT2	9.9	9.9	19.390	NADH dehydrogenase [ubiquinone] iron-sulfur protein 3, mitochondrial	6	0.99130	0.65660	1.00802	0.66297
Q9DCV7	33.6	43.45	47.700	Keratin, type II cytoskeletal 7	42	1.53712	1.34786	0.65007	0.86909
Q9DCW4	12.99	13.2	25.490	Electron transfer flavoprotein subunit beta	7	0.91837	0.47084	1.08806	0.51722
Q9DCX2	10.89	10.97	32.300	ATP synthase subunit d, mitochondrial	6	1.01170	0.78571	0.98769	0.77308
Q9DD20	4.35	4.44	7.787	Methyltransferase-like protein 7B	4	1.68293	0.70953	0.59375	0.43143
Q9EPB4	1.89	1.89	7.254	Apoptosis-associated speck-like protein containing a CARD	2	1.06681	0.78316	0.93666	0.73680
Q9EPK2	6.42	6.42	7.493	Protein XRP2	3	0.78032	0.90206	1.28056	1.15773
Q9EPS2	2.01	2.01	9.184	Peptide YY	1	0.85948	0.71755	1.16261	0.83792

Q9EQ06	4.06	4.46	5.369	Estradiol 17-beta-dehydrogenase 11	2	1.05606	0.55346	0.94620	0.53183
Q9EQH2	1.87	2.65	1.398	Endoplasmic reticulum aminopeptidase 1	1	1.05786	0.80988	0.94459	0.76771
Q9EQI8	1.97	2.36	7.067	39S ribosomal protein L46, mitochondrial	2	1.06324	0.92177	0.93981	0.87012
Q9EQK5	10.13	13.44	8.943	Major vault protein	8	1.04446	0.94275	0.95671	0.90010
Q9ERE2	7.27	9.71	9.563	Keratin, type II cuticular Hb1	9	2.58514	1.00619	0.38653	0.38918
Q9ERE7	2.07	2.07	3.571	LDLR chaperone MESD	1	1.00923	0.70156	0.99011	0.69769
Q9ERG0	20.37	20.83	10.090	LIM domain and actin-binding protein 1	8	0.94840	1.05576	1.05361	1.11137
Q9ERI2	2.86	5.5	18.100	Ras-related protein Rab-27A	5	0.82656	0.63955	1.20891	0.77630
Q9ERS2	6	6.05	21.530	NADH dehydrogenase [ubiquinone] 1 alpha subcomplex subunit 13	4	1.14740	0.84033	0.87088	0.73429
Q9ES64	2.04	2.63	2.857	Harmonin	2	0.66742	0.35305	1.49718	0.53092
Q9ES97	3.96	5.03	1.971	Reticulon-3	3	1.88363	1.77193	0.53049	0.94260
Q9ESP1	4	4.52	9.502	Stromal cell-derived factor 2-like protein 1	2	1.14252	1.07399	0.87460	0.94491
Q9ET30	3.42	4.59	2.385	Transmembrane 9 superfamily member 3	3	1.30463	0.91834	0.76592	0.70745
Q9JHC0	5	5.06	16.320	Glutathione peroxidase 2	3	0.88491	1.01914	1.12920	1.15422
Q9JHJ0	7.02	7.99	10.510	Tropomodulin-3	4	1.02268	1.01521	0.97708	0.99721
Q9JHK0	1.55	2.18	2.632	Prolactin-2A1	1	1.16048	1.93405	0.86106	1.67270
Q9JHS3	1.49	1.51	7.200	Ragulator complex protein LAMTOR2	1	0.93688	1.63940	1.06657	1.75627
Q9JHU4	6.14	16.39	1.055	Cytoplasmic dynein 1 heavy chain 1	5	0.82656	0.94263	1.20891	1.14056
Q9JI18	1.38	2.67	0.217	Low-density lipoprotein receptor-related protein 1B	1	0.99235	0.67611	1.00694	0.69780
Q9JI67	4.56	8.11	7.143	Beta-1,3-galactosyltransferase 5	4	0.77544	0.47129	1.28862	0.61739
Q9JIF7	7.15	9.09	4.092	Coatomer subunit beta	4	0.98546	0.82544	1.01399	0.84533
Q9JII6	5.42	5.77	8.308	Alcohol dehydrogenase [NADP(+)]	3	0.81925	0.88442	1.21970	1.08456
Q9JIP7	1.5	1.86	0.987	Solute carrier family 15 member 1	1	0.38644	0.31173	2.58574	0.80962
Q9JIW9	2.08	2.14	5.340	Ras-related protein Ral-B	1	1.12345	1.31363	0.88944	1.17528
Q9JJ00	3.39	3.87	3.049	Phospholipid scramblase 1	1	0.76829	0.81444	1.30061	1.06416

Q9JJ06	3.37	3.63	6.612	Glycoprotein-N-acetylgalactosamine 3-beta-galactosyltransferase 1	4	0.98291	0.70935	1.01662	0.72287
Q9JJF9	2	2.27	1.530	Signal peptide peptidase-like 2A	1	1.13599	1.00715	0.87963	0.88984
Q9JJI8	2.25	2.34	28.570	60S ribosomal protein L38	2	0.98079	1.35911	1.01881	1.39111
Q9JJX6	4.52	6.18	6.701	P2X purinoceptor 4	3	0.76187	0.82516	1.31156	1.08631
Q9JJY3	2.73	3.52	2.901	Sphingomyelin phosphodiesterase 3	2	1.03953	1.05844	0.96124	1.01998
Q9JJZ4	2	2.04	2.201	Ubiquitin-conjugating enzyme E2 J1	1	1.50341	1.16097	0.66465	0.77506
Q9JKA5	11.05	11.57	22.880	Cell surface A33 antigen	12	0.92736	0.73255	1.07752	0.79550
Q9JKF1	14.17	17.84	4.587	Ras GTPase-activating-like protein IQGAP1	8	0.81984	1.26067	1.21883	1.55084
Q9JKR6	15.26	17.5	6.406	Hypoxia up-regulated protein 1	7	0.97782	0.77888	1.02191	0.79769
Q9JL27	3.49	3.81	5.764	Galactoside 2-alpha-L-fucosyltransferase 2	3	1.29570	0.70554	0.77120	0.55395
Q9JLQ0	7.26	7.72	6.436	CD2-associated protein	4	0.85415	0.94239	1.16987	1.10773
Q9JLZ6	1.92	2.42	1.616	Hypermethylated in cancer 2 protein	2	1.30043	1.67054	0.76839	1.28931
Q9JM76	3.15	3.47	6.180	Actin-related protein 2/3 complex subunit 3	1	0.95394	1.01688	1.04749	1.06967
Q9JM95	7.18	7.2	11.410	Alpha-N-acetylgalactosaminide alpha-2,6-sialyltransferase 6	4	1.42465	1.00536	0.70139	0.70494
Q9JMA9	2.07	2.53	1.097	Sodium- and chloride-dependent neutral and basic amino acid transporter B(0+)	1	0.55135	0.43357	1.81236	0.78989
Q9JMG2	2.71	3.27	3.165	C1GALT1-specific chaperone 1	1	1.32457	1.01575	0.75439	0.76882
Q9JMH9	2.28	4.61	0.781	Unconventional myosin-XVIIIa	2	1.26510	1.15984	0.78985	0.92113
Q9NYQ2	15.04	16.89	20.960	Hydroxyacid oxidase 2	8	1.36626	0.60689	0.73137	0.46640
Q9QUI0	6.17	6.28	18.650	Transforming protein RhoA	5	0.63451	0.93474	1.57482	1.47652
Q9QXS1	70.07	70.7	5.564	Plectin	29	1.23328	1.09107	0.81023	0.88453
Q9QXX4	5.2	7.1	5.621	Calcium-binding mitochondrial carrier protein Aralar2	4	1.10002	1.08708	0.90838	1.00193
Q9QY23	2.12	2.21	1.004	Plakophilin-3	1	1.27990	1.25547	0.78072	0.99247
Q9QY76	2.03	2.13	3.292	Vesicle-associated membrane protein-associated protein B	1	1.09283	1.04114	0.91436	0.94079
Q9QYA2	2	2.04	3.047	Mitochondrial import receptor subunit TOM40 homolog	1	1.08472	1.07665	0.92120	0.99620

Q9QYB1	2.01	2.91	4.348	Chloride intracellular channel protein 4	1	0.71626	1.38304	1.39508	1.93798
Q9QYJ0	4.27	5.63	6.553	DnaJ homolog subfamily A member 2	3	0.84300	1.07123	1.18535	1.26930
Q9QZD9	2.07	2.21	3.077	Eukaryotic translation initiation factor 3 subunit I	1	0.90981	0.78823	1.09830	0.86954
Q9QZE5	2.07	2.56	1.030	Coatomer subunit gamma-1	1	0.96738	1.03595	1.03294	1.07370
Q9R092	2	2.12	2.839	17-beta-hydroxysteroid dehydrogenase type 6	1	1.56298	2.52621	0.63932	1.62220
Q9R0A1	3.92	4.21	2.753	Chloride channel protein 2	2	1.06895	0.68672	0.93479	0.65082
Q9R0H0	4.88	4.88	4.841	Peroxisomal acyl-coenzyme A oxidase 1	3	1.43260	0.89153	0.69751	0.62751
Q9R0K7	3.07	3.87	2.170	Plasma membrane calcium-transporting ATPase 2	3	0.98492	0.71946	1.01454	0.73306
Q9R0P3	1.98	2.46	4.255	S-formylglutathione hydrolase	1	1.09175	1.14084	0.91527	1.04880
Q9R0P5	16.58	16.76	50.910	Destrin	12	0.67948	0.64575	1.47060	0.94994
Q9R0Q3	4.16	4.16	10.450	Transmembrane emp24 domain-containing protein 2	3	1.39488	0.74084	0.71636	0.53046
Q9R100	26.25	26.71	17.530	Cadherin-17	20	0.91543	0.68471	1.09156	0.74666
Q9R112	10.2	10.76	12.670	Sulfide:quinone oxidoreductase, mitochondrial	6	1.29535	0.81689	0.77141	0.63910
Q9R1J0	2.18	2.46	3.591	Sterol-4-alpha-carboxylate 3-dehydrogenase, decarboxylating	1	1.44333	0.92494	0.69232	0.64319
Q9R1P4	2.34	2.46	3.422	Proteasome subunit alpha type-1	1	0.96586	1.22728	1.03456	1.27339
Q9R1Q7	1.64	1.64	7.895	Proteolipid protein 2	1	0.99767	0.80108	1.00158	0.80590
Q9WTL2	2.32	4.2	9.859	Ras-related protein Rab-25	2	1.07927	0.81816	0.92585	0.76084
Q9WTM5	1.73	1.84	2.592	RuvB-like 2	1	0.87342	0.98493	1.14405	1.13384
Q9WTP6	4.96	6.22	11.300	Adenylate kinase 2, mitochondrial	3	0.88407	0.67547	1.13028	0.76799
Q9WTP7	11.28	11.57	33.040	GTP:AMP phosphotransferase AK3, mitochondrial	8	0.97465	0.55725	1.02523	0.57442
Q9WTX5	1.85	2.15	4.908	S-phase kinase-associated protein 1	1	0.81701	1.01548	1.22305	1.24748
Q9WU01	2.17	2.35	4.298	KH domain-containing, RNA-binding, signal transduction-associated protein 2	2	0.92743	1.03815	1.07743	1.12943
Q9WU78	17.32	20.21	8.170	Programmed cell death 6-interacting protein	11	0.93070	1.05997	1.07365	1.14104
Q9WUR2	4.05	4.26	5.371	Enoyl-CoA delta isomerase 2, mitochondrial	2	0.83842	0.58937	1.19182	0.70064
Q9WUZ9	2.52	3.06	2.342	Ectonucleoside triphosphate diphosphohydrolase 5	1	0.96038	0.76816	1.04047	0.80779

Q9WV02	6.31	6.31	8.696	RNA-binding motif protein, X chromosome	3	1.14545	1.18094	0.87236	1.03131
Q9WV54	4.15	4.44	5.076	Acid ceramidase	2	1.05513	1.13385	0.94703	1.08692
Q9WV55	2.15	2.15	3.213	Vesicle-associated membrane protein-associated protein A	1	1.62646	1.00895	0.61437	0.62066
Q9WV80	3.34	5.78	6.130	Sorting nexin-1	4	1.02886	0.85257	0.97122	0.83278
Q9WV91	2.12	2.67	1.138	Prostaglandin F2 receptor negative regulator	1	0.85896	1.19582	1.16332	1.40406
Q9WV92	4.18	4.82	1.615	Band 4.1-like protein 3	2	0.78485	0.72273	1.27316	0.92494
Q9WVA4	8.05	8.12	25.130	Transgelin-2	8	0.78013	1.10278	1.28086	1.47490
Q9WVC8	10.29	10.55	7.794	Chloride anion exchanger	6	0.39338	0.32195	2.54014	0.67896
Q9WVP1	2.08	2.3	1.891	AP-1 complex subunit mu-2	1	0.73216	0.96806	1.36480	1.32705
Q9Z0F4	2.09	2.13	4.712	Calcium and integrin-binding protein 1	1	0.57200	0.59577	1.74693	1.04537
Q9Z0F8	2.02	2.21	0.967	Disintegrin and metalloproteinase domain-containing protein 17	1	0.90812	1.46116	1.10035	1.61489
Q9Z0G9	5.03	5.03	11.870	Claudin-3	5	0.87099	0.44487	1.14725	0.52637
Q9Z0L8	2.26	2.36	3.470	Gamma-glutamyl hydrolase	1	0.87085	0.67592	1.14744	0.77769
Q9Z0P4	2	2.16	3.394	Paralemmin-1	1	0.92014	1.60086	1.08597	1.74617
Q9Z0S1	1.74	1.79	3.896	3'(2'),5'-bisphosphate nucleotidase 1	1	0.82856	0.75071	1.20600	0.90936
Q9Z0U1	9.67	11.12	3.770	Tight junction protein ZO-2	4	1.09135	1.21190	0.91560	1.11401
Q9Z0X1	5.89	6.46	5.392	Apoptosis-inducing factor 1, mitochondrial	3	1.16392	1.07278	0.85852	0.92989
Q9Z110	3.28	4.95	2.516	Delta-1-pyrroline-5-carboxylate synthase	3	0.94672	0.72878	1.05548	0.77374
Q9Z127	2.06	2.19	2.734	Large neutral amino acids transporter small subunit 1	1	1.24707	2.21130	0.80127	1.78122
Q9Z1G3	1.59	1.75	2.618	V-type proton ATPase subunit C 1	1	0.65332	1.00111	1.52948	1.51499
Q9Z1G4	4.58	4.94	2.384	V-type proton ATPase 116 kDa subunit a isoform 1	2	0.98498	0.91330	1.01448	0.93070
Q9Z1P6	4.97	5.05	30.090	NADH dehydrogenase [ubiquinone] 1 alpha subcomplex subunit 7	3	1.05993	1.07545	0.94274	1.01934
Q9Z1Q5	4.32	4.93	12.450	Chloride intracellular channel protein 1	3	0.81713	1.04737	1.22287	1.28691
Q9Z1W8	21.03	33.45	16.810	Potassium-transporting ATPase alpha chain 2	23	0.44152	0.46368	2.26320	1.04817

Q9Z204	6.91	7.05	13.420	Heterogeneous nuclear ribonucleoproteins C1/C2	4	1.18343	1.06089	0.84436	0.89665
Q9Z261	2	2	7.583	Claudin-7	4	1.07535	0.70581	0.92922	0.65876
Q9Z2I0	10.65	13.2	7.453	LETM1 and EF-hand domain-containing protein 1, mitochondrial	6	1.05735	0.67478	0.94504	0.64567
Q9Z2I8	15.77	17.33	17.320	Succinyl-CoA ligase [GDP-forming] subunit beta, mitochondrial	9	0.90903	0.57000	1.09924	0.62894
Q9Z2I9	7.96	11.07	11.020	Succinyl-CoA ligase [ADP-forming] subunit beta, mitochondrial	6	1.03716	0.73023	0.96344	0.70610
Q9Z2M6	1.48	1.52	5.983	Ubiquitin-like protein 3	1	0.74321	1.05180	1.34450	1.41868
Q9Z2M7	4.5	4.56	10.330	Phosphomannomutase 2	3	0.96177	0.86260	1.03897	0.89974
Q9Z2Q6	3.32	3.39	5.149	Septin-5	2	0.89637	1.25908	1.11477	1.40794
Q9Z2U0	8.22	10.43	17.740	Proteasome subunit alpha type-7	6	0.91105	1.03331	1.09680	1.14047
Q9Z2U1	2.84	3.55	6.224	Proteasome subunit alpha type-5	2	0.69974	0.83025	1.42802	1.19136
Q9Z2W0	2.03	2.35	2.114	Aspartyl aminopeptidase	1	1.06148	1.37116	0.94136	1.29375
Q9Z2X1	2.6	3.03	2.892	Heterogeneous nuclear ribonucleoprotein F	1	0.67022	0.72964	1.49093	1.09266
RRRRRA2AAJ9	1.74	10.3	0.540	REVERSED Obscurin	4	0.85602	0.95481	1.16731	1.19390

Table S7. The differentially expressed proteins between the cancer and normal membrane fractions enriched from the colon mucosa

Accession	Name	Average (adjacent to normal)	Average (cancer to normal)	Average (cancer to adjacent)	Pcancer to normal	Pcancer to adjacent	_LogPcancer to normal	_LogPcancer to adjacent	cancer to normal	cancer to adjacent
B2RX12	Canalicular multispecific organic anion transporter 2	1.008	0.547	0.610	1.47E-03	2.57E-03	2.834	2.589	down	down
O08749	Dihydrolipoyl dehydrogenase, mitochondrial	1.048	0.471	0.647	2.64E-02	9.25E-02	1.579	1.034	down	non

O08756	3-hydroxyacyl-CoA dehydrogenase type-2	1.009	0.556	0.639	2.23E-03	6.36E-03	2.653	2.196	down	non
O35143	ATPase inhibitor, mitochondrial	1.053	0.491	0.363	3.70E-02	1.57E-02	1.432	1.804	down	down
O70475	UDP-glucose 6-dehydrogenase	1.014	0.476	0.568	2.03E-03	5.77E-03	2.692	2.239	down	down
O88312	Anterior gradient protein 2 homolog	1.007	0.184	0.174	1.90E-05	1.78E-05	4.722	4.750	down	down
O88343	Electrogenic sodium bicarbonate cotransporter 1	1.015	0.499	0.587	3.06E-03	7.90E-03	2.514	2.102	down	down
O89001	Carboxypeptidase D	1.014	0.633	0.755	1.24E-02	5.61E-02	1.906	1.251	down	non
P00405	Cytochrome c oxidase subunit 2	1.001	0.626	0.592	2.43E-05	1.38E-05	4.614	4.859	down	down
P01902	H-2 class I histocompatibility antigen, K-D alpha chain	1.061	0.224	0.319	8.88E-03	1.55E-02	2.052	1.810	down	down
P02088	Hemoglobin subunit beta-1	1.050	0.433	0.599	2.18E-02	6.96E-02	1.661	1.157	down	non
P08249	Malate dehydrogenase, mitochondrial	1.002	0.451	0.432	1.03E-05	6.96E-06	4.987	5.157	down	down
P13808	Anion exchange protein 2	1.007	0.566	0.637	7.19E-04	2.16E-03	3.143	2.666	down	non
P18572	Basigin	1.018	0.464	0.554	3.31E-03	8.18E-03	2.481	2.087	down	down
P29452	Caspase-1	1.020	0.637	0.783	2.28E-02	1.18E-01	1.642	0.927	down	non
P30275	Creatine kinase U-type, mitochondrial	1.003	0.417	0.446	1.54E-05	1.47E-05	4.811	4.833	down	down
P38060	Hydroxymethylglutaryl-CoA lyase, mitochondrial	1.015	0.498	0.584	3.01E-03	8.81E-03	2.522	2.055	down	down
P38647	Stress-70 protein, mitochondrial	1.007	0.588	0.671	1.23E-03	4.27E-03	2.910	2.370	down	non

P47738	Aldehyde dehydrogenase, mitochondrial	1.007	0.496	0.560	4.92E-04	1.05E-03	3.308	2.980	down	down
P48758	Carbonyl reductase [NADPH] 1	1.020	0.537	0.661	8.53E-03	3.57E-02	2.069	1.447	down	non
P53986	Monocarboxylate transporter 1	1.027	0.567	0.680	5.48E-03	2.25E-02	2.261	1.648	down	non
P54869	Hydroxymethylglutaryl-CoA synthase, mitochondrial	1.011	0.323	0.276	2.65E-04	1.65E-04	3.576	3.782	down	down
P55088	Aquaporin-4	1.070	0.279	0.412	1.66E-02	3.77E-02	1.780	1.424	down	down
P56391	Cytochrome c oxidase subunit 6B1	1.020	0.572	0.471	1.16E-02	4.21E-03	1.937	2.375	down	down
P56480	ATP synthase subunit beta, mitochondrial	1.007	0.606	0.540	1.41E-03	5.54E-04	2.850	3.257	down	down
P62897	Cytochrome c, somatic	1.036	0.611	0.476	4.85E-02	1.64E-02	1.314	1.785	down	down
P63038	60 kDa heat shock protein, mitochondrial	1.013	0.491	0.570	2.16E-03	4.69E-03	2.666	2.329	down	down
P97807	Fumarate hydratase, mitochondrial	1.047	0.509	0.690	3.56E-02	1.27E-01	1.448	0.896	down	non
Q07417	Short-chain specific acyl-CoA dehydrogenase, mitochondrial	1.001	0.461	0.451	7.48E-06	3.24E-06	5.126	5.489	down	down
Q09199	Beta-1,4 N-acetylgalactosaminyltransferase 2	1.003	0.514	0.576	3.16E-05	7.55E-05	4.501	4.122	down	down
Q10470	Beta-1,4-mannosyl-glycoprotein 4-beta-N-acetylglucosaminyltransferase	1.006	0.384	0.436	2.16E-04	6.49E-04	3.666	3.188	down	down
Q3TMQ6	Angiogenin-4	1.037	0.343	0.440	7.73E-03	1.56E-02	2.112	1.806	down	down

Q61425	Hydroxyacyl-coenzyme A dehydrogenase, mitochondrial	1.006	0.530	0.507	2.92E-04	2.32E-04	3.534	3.635	down	down
Q62273	Sulfate transporter	1.028	0.512	0.653	1.44E-02	4.95E-02	1.842	1.305	down	non
Q62433	Protein NDRG1	1.008	0.583	0.520	1.41E-03	6.37E-04	2.851	3.196	down	down
Q64133	Amine oxidase [flavin-containing] A	1.005	0.580	0.543	6.08E-04	3.92E-04	3.216	3.407	down	down
Q7TST0	Butyrophilin-like protein 1	1.018	0.320	0.392	1.13E-03	2.69E-03	2.945	2.571	down	down
Q80V26	Inositol monophosphatase 3	1.005	0.532	0.563	3.14E-04	4.28E-04	3.503	3.368	down	down
Q810Q5	Normal mucosa of esophagus-specific gene 1 protein	1.019	0.561	0.466	1.04E-02	3.91E-03	1.983	2.408	down	down
Q8BG05	Heterogeneous nuclear ribonucleoprotein A3	1.003	0.382	0.414	1.20E-05	1.74E-05	4.922	4.759	down	down
Q8BH95	Enoyl-CoA hydratase, mitochondrial	1.004	0.516	0.479	2.34E-04	1.72E-04	3.632	3.764	down	down
Q8BK48	Pyrethroid hydrolase Ces2e	1.019	0.443	0.385	3.55E-03	2.40E-03	2.450	2.620	down	down
Q8BMF4	Dihydrolipoyllysine-residue acetyltransferase component of pyruvate dehydrogenase complex, mitochondrial	1.017	0.536	0.652	6.89E-03	2.54E-02	2.162	1.596	down	non
Q8C0Z1	Protein ITFG3	1.020	0.514	0.630	7.52E-03	2.80E-02	2.124	1.553	down	non
Q8CI85	Carbonic anhydrase 12	1.000	0.507	0.519	3.69E-08	1.24E-07	7.433	6.908	down	down
Q8QZR3	Pyrethroid hydrolase Ces2a	1.004	0.468	0.429	6.40E-05	4.27E-05	4.194	4.370	down	down
Q8QZT1	Acetyl-CoA acetyltransferase, mitochondrial	1.000	0.626	0.619	1.45E-05	3.97E-06	4.840	5.401	down	down
Q8R3G9	Tetraspanin-8	1.046	0.553	0.750	4.55E-02	1.87E-01	1.342	0.727	down	non
Q8VCC2	Liver carboxylesterase 1	1.028	0.537	0.449	1.79E-02	8.91E-03	1.746	2.050	down	down

Q8VCW8	Acyl-CoA synthetase family member 2, mitochondrial	1.021	0.488	0.589	8.62E-03	2.30E-02	2.065	1.639	down	non
Q8VDN2	Sodium/potassium-transporting ATPase subunit alpha-1	1.049	0.408	0.552	1.80E-02	4.87E-02	1.744	1.313	down	down
Q91W90	Thioredoxin domain-containing protein 5	1.011	0.573	0.669	3.27E-03	1.10E-02	2.486	1.958	down	non
Q91WG0	Acylcarnitine hydrolase	1.045	0.354	0.274	1.08E-02	6.32E-03	1.966	2.199	down	down
Q91Y97	Fructose-bisphosphate aldolase B	1.008	0.632	0.722	3.29E-03	1.36E-02	2.482	1.866	down	non
Q99J99	3-mercaptopyruvate sulfurtransferase	1.016	0.331	0.405	1.01E-03	2.26E-03	2.998	2.647	down	down
Q99LC5	Electron transfer flavoprotein subunit alpha, mitochondrial	1.000	0.462	0.469	1.97E-08	2.58E-08	7.705	7.588	down	down
Q9CZS1	Aldehyde dehydrogenase X, mitochondrial	1.039	0.333	0.443	6.63E-03	1.47E-02	2.178	1.833	down	down
Q9D051	Pyruvate dehydrogenase E1 component subunit beta, mitochondrial	1.006	0.470	0.530	2.05E-04	4.16E-04	3.687	3.381	down	down
Q9D154	Leukocyte elastase inhibitor A	1.022	0.601	0.738	1.97E-02	8.73E-02	1.705	1.059	down	non
Q9D312	Keratin, type I cytoskeletal 20	1.044	0.497	0.388	2.93E-02	1.34E-02	1.533	1.873	down	down
Q9D6R2	Isocitrate dehydrogenase [NAD] subunit alpha, mitochondrial	1.010	0.548	0.643	1.88E-03	6.68E-03	2.725	2.175	down	non
Q9DCS3	Trans-2-enoyl-CoA reductase, mitochondrial	1.060	0.473	0.672	3.84E-02	1.37E-01	1.416	0.865	down	non

Q9DCW4	Electron transfer flavoprotein subunit beta	1.001	0.467	0.497	2.36E-06	4.44E-06	5.628	5.352	down	down
Q9JI67	Beta-1,3-galactosyltransferase 5	1.031	0.432	0.560	8.34E-03	2.54E-02	2.079	1.595	down	down
Q9WTP7	GTP:AMP phosphotransferase AK3, mitochondrial	1.016	0.481	0.556	3.33E-03	6.77E-03	2.477	2.169	down	down
Q9Z0G9	Claudin-3	1.009	0.416	0.491	3.23E-04	8.05E-04	3.491	3.094	down	down
Q9Z2I8	Succinyl-CoA ligase [GDP-forming] subunit beta, mitochondrial	1.002	0.535	0.568	6.27E-05	2.50E-04	4.203	3.602	down	down
A2A863	Integrin beta-4	1.009	1.617	1.402	2.88E-04	4.44E-03	3.541	2.353	up	non
O08638	Myosin-11	1.039	1.713	1.264	1.31E-02	2.66E-01	1.881	0.575	up	non
O08917	Flotillin-1	1.044	2.641	1.975	5.06E-03	1.89E-02	2.295	1.723	up	non
O35640	Annexin A8	1.031	2.562	2.066	4.39E-05	7.69E-04	4.358	3.114	up	up
P02301	Histone H3.3C	1.006	2.176	2.206	1.20E-03	1.15E-05	2.923	4.940	up	up
P06800	Receptor-type tyrosine-protein phosphatase C	1.011	3.311	2.912	1.72E-06	5.29E-06	5.764	5.277	up	up
P07356	Annexin A2	1.005	2.381	2.134	3.30E-07	2.09E-06	6.482	5.680	up	up
P10126	Elongation factor 1-alpha 1	1.009	1.728	1.508	2.96E-04	1.23E-03	3.528	2.909	up	non
P10404	MLV-related proviral Env polyprotein	1.007	2.777	2.575	6.43E-07	9.82E-07	6.191	6.008	up	up
P11499	Heat shock protein HSP 90-beta	1.012	1.649	1.424	5.66E-04	4.98E-03	3.247	2.303	up	non
P11672	Neutrophil gelatinase-associated lipocalin	1.040	2.854	2.272	1.01E-04	3.32E-04	3.997	3.478	up	up
P11688	Integrin alpha-5	1.045	1.718	1.291	1.37E-02	2.79E-01	1.862	0.554	up	non

P14824	Annexin A6	1.009	3.941	3.577	5.48E-04	4.31E-05	3.261	4.366	up	up
P15105	Glutamine synthetase	1.006	1.590	1.442	1.30E-04	1.71E-03	3.886	2.767	up	non
P16045	Galectin-1	1.007	5.038	5.593	1.44E-04	1.35E-03	3.843	2.870	up	up
P17427	AP-2 complex subunit alpha-2	1.002	1.932	1.915	9.57E-06	2.66E-04	5.019	3.576	up	up
P18242	Cathepsin D	1.000	1.774	1.769	8.28E-04	1.92E-03	3.082	2.717	up	non
P18760	Cofilin-1	1.017	1.519	1.838	4.54E-03	3.14E-04	2.343	3.502	up	up
P19324	Serpin H1	1.039	2.963	2.580	1.68E-04	1.16E-04	3.774	3.936	up	up
P26041	Moesin	1.010	3.929	3.641	2.19E-04	3.34E-04	3.659	3.476	up	up
P28667	MARCKS-related protein	1.058	3.834	2.947	9.87E-03	4.34E-02	2.006	1.363	up	up
P37804	Transgelin	1.019	1.611	1.330	3.48E-03	5.02E-02	2.458	1.299	up	non
P43406	Integrin alpha-V	1.002	1.683	1.603	3.67E-05	2.52E-04	4.435	3.598	up	non
P48036	Annexin A5	1.020	2.789	2.331	5.42E-06	3.06E-05	5.266	4.514	up	up
P54116	Erythrocyte band 7 integral membrane protein	1.028	1.854	2.386	5.00E-03	5.21E-04	2.301	3.283	up	up
P62267	40S ribosomal protein S23	1.007	2.562	2.426	2.32E-07	7.26E-06	6.635	5.139	up	up
P62702	40S ribosomal protein S4, X isoform	1.015	1.770	2.098	3.10E-04	3.49E-05	3.509	4.458	up	up
P62806	Histone H4	1.038	3.030	2.312	1.80E-04	3.17E-04	3.744	3.498	up	up
P67984	60S ribosomal protein L22	1.028	1.615	1.286	1.01E-02	1.57E-01	1.996	0.803	up	non
Q01339	Beta-2-glycoprotein 1	1.052	1.585	1.160	4.20E-02	6.27E-01	1.377	0.203	up	non
Q07076	Annexin A7	1.005	1.917	1.800	5.49E-06	7.88E-06	5.260	5.104	up	up
Q60634	Flotillin-2	1.009	2.286	2.020	8.68E-06	2.17E-05	5.061	4.663	up	up
Q61233	Plastin-2	1.000	1.776	1.788	3.89E-05	7.74E-05	4.410	4.111	up	up
Q61735	Leukocyte surface antigen CD47	1.013	2.012	1.717	2.29E-03	4.70E-03	2.640	2.328	up	non
Q69ZN7	Myoferlin	1.005	2.270	2.116	3.91E-07	3.86E-06	6.408	5.414	up	up

Q80UG5	Septin-9	1.014	1.970	1.713	1.95E-03	1.27E-03	2.709	2.895	up	up
Q8BP67	60S ribosomal protein L24	1.000	1.596	1.560	6.93E-08	7.38E-08	7.159	7.132	up	up
Q8K419	Galectin-4	1.008	1.628	1.427	1.87E-04	2.11E-03	3.728	2.675	up	non
Q8R2S8	CD177 antigen	1.006	1.955	1.789	8.49E-05	8.21E-05	4.071	4.086	up	up
Q8VDD5	Myosin-9	1.045	1.984	1.498	6.47E-03	6.42E-02	2.189	1.193	up	non
Q8VEM8	Phosphate carrier protein, mitochondrial	1.044	1.595	1.201	3.10E-02	4.71E-01	1.508	0.327	up	non
Q91VR8	Protein BRICK1	1.009	1.982	1.833	5.04E-03	2.76E-02	2.297	1.559	up	non
Q9DBG9	Tax1-binding protein 3	1.000	1.563	1.595	2.29E-07	8.69E-07	6.640	6.061	up	up
Q9JHS3	Ragulator complex protein LAMTOR2	1.026	1.947	1.762	7.84E-03	2.04E-03	2.106	2.691	up	up
Q9JLZ6	Hypermethylated in cancer 2 protein	1.015	3.107	2.726	3.52E-03	9.67E-03	2.453	2.015	up	non

Table S8. The differential proteins between the cancer and adjacent membrane fractions enriched from the colon mucosa

Accession	Name	Average (adjacent to normal)	Average (cancer to normal)	Average (cancer to adjacent)	Pcancer to normal	Pcancer to adjacent	_Log Pcancer to normal	_Log Pcancer to adjacent	cancer to normal	cancer to adjacent
B2RX12	Canalicular multispecific organic anion transporter 2	1.008	0.547	0.610	1.47E-03	2.57E-03	2.83	2.59	down	down
O35143	ATPase inhibitor, mitochondrial	1.053	0.491	0.363	3.70E-02	1.57E-02	1.43	1.80	down	down
O70475	UDP-glucose 6-dehydrogenase	1.014	0.476	0.568	2.03E-03	5.77E-03	2.69	2.24	down	down
O88312	Anterior gradient protein 2 homolog	1.007	0.184	0.174	1.90E-05	1.78E-05	4.72	4.75	down	down

O88343	Electrogenic sodium bicarbonate cotransporter 1	1.015	0.499	0.587	3.06E-03	7.90E-03	2.51	2.10	down	down
P00405	Cytochrome c oxidase subunit 2	1.001	0.626	0.592	2.43E-05	1.38E-05	4.61	4.86	down	down
P01902	H-2 class I histocompatibility antigen, K-D alpha chain	1.061	0.224	0.319	8.88E-03	1.55E-02	2.05	1.81	down	down
P08249	Malate dehydrogenase, mitochondrial	1.002	0.451	0.432	1.03E-05	6.96E-06	4.99	5.16	down	down
P18572	Basigin	1.018	0.464	0.554	3.31E-03	8.18E-03	2.48	2.09	down	down
P19536	Cytochrome c oxidase subunit 5B, mitochondrial	1.042	0.695	0.535	1.20E-01	3.53E-02	0.92	1.45	non	down
P19783	Cytochrome c oxidase subunit 4 isoform 1, mitochondrial	1.020	0.746	0.612	7.81E-02	1.74E-02	1.11	1.76	non	down
P27773	Protein disulfide-isomerase A3	1.037	0.758	0.584	1.67E-01	4.07E-02	0.78	1.39	non	down
P30275	Creatine kinase U-type, mitochondrial	1.003	0.417	0.446	1.54E-05	1.47E-05	4.81	4.83	down	down
P35276	Ras-related protein Rab-3D	1.003	0.679	0.638	6.23E-04	1.81E-04	3.21	3.74	non	down
P38060	Hydroxymethylglutaryl-CoA lyase, mitochondrial	1.015	0.498	0.584	3.01E-03	8.81E-03	2.52	2.06	down	down
P47738	Aldehyde dehydrogenase, mitochondrial	1.007	0.496	0.560	4.92E-04	1.05E-03	3.31	2.98	down	down
P52503	NADH dehydrogenase [ubiquinone] iron-sulfur protein 6, mitochondrial	1.024	0.635	0.537	3.41E-02	1.31E-02	1.47	1.88	non	down
P54869	Hydroxymethylglutaryl-CoA synthase, mitochondrial	1.011	0.323	0.276	2.65E-04	1.65E-04	3.58	3.78	down	down
P55088	Aquaporin-4	1.070	0.279	0.412	1.66E-02	3.77E-02	1.78	1.42	down	down
P56391	Cytochrome c oxidase subunit 6B1	1.020	0.572	0.471	1.16E-02	4.21E-03	1.94	2.38	down	down

P56480	ATP synthase subunit beta, mitochondrial	1.007	0.606	0.540	1.41E-03	5.54E-04	2.85	3.26	down	down
P62897	Cytochrome c, somatic	1.036	0.611	0.476	4.85E-02	1.64E-02	1.31	1.79	down	down
P63038	60 kDa heat shock protein, mitochondrial	1.013	0.491	0.570	2.16E-03	4.69E-03	2.67	2.33	down	down
P97805	Protein FAM3D	1.006	0.659	0.602	2.66E-03	1.28E-03	2.57	2.89	non	down
P99028	Cytochrome b-c1 complex subunit 6, mitochondrial	1.022	0.703	0.592	5.74E-02	1.90E-02	1.24	1.72	non	down
Q07417	Short-chain specific acyl-CoA dehydrogenase, mitochondrial	1.001	0.461	0.451	7.48E-06	3.24E-06	5.13	5.49	down	down
Q09199	Beta-1,4 N-acetylgalactosaminyltransferase 2	1.003	0.514	0.576	3.16E-05	7.55E-05	4.50	4.12	down	down
Q10470	Beta-1,4-mannosyl-glycoprotein 4-beta-N-acetylglucosaminyltransferase	1.006	0.384	0.436	2.16E-04	6.49E-04	3.67	3.19	down	down
Q3TMQ6	Angiogenin-4	1.037	0.343	0.440	7.73E-03	1.56E-02	2.11	1.81	down	down
Q61420	CMP-sialic acid transporter	1.036	0.642	0.497	6.92E-02	2.05E-02	1.16	1.69	non	down
Q61425	Hydroxyacyl-coenzyme A dehydrogenase, mitochondrial	1.006	0.530	0.507	2.92E-04	2.32E-04	3.53	3.64	down	down
Q62433	Protein NDRG1	1.008	0.583	0.520	1.41E-03	6.37E-04	2.85	3.20	down	down
Q64133	Amine oxidase [flavin-containing] A	1.005	0.580	0.543	6.08E-04	3.92E-04	3.22	3.41	down	down
Q7TST0	Butyrophilin-like protein 1	1.018	0.320	0.392	1.13E-03	2.69E-03	2.95	2.57	down	down
Q80V26	Inositol monophosphatase 3	1.005	0.532	0.563	3.14E-04	4.28E-04	3.50	3.37	down	down
Q80XN0	D-beta-hydroxybutyrate dehydrogenase, mitochondrial	1.022	0.663	0.546	3.77E-02	1.16E-02	1.42	1.94	non	down

Q810Q5	Normal mucosa of esophagus-specific gene 1 protein	1.019	0.561	0.466	1.04E-02	3.91E-03	1.98	2.41	down	down
Q8BG05	Heterogeneous nuclear ribonucleoprotein A3	1.003	0.382	0.414	1.20E-05	1.74E-05	4.92	4.76	down	down
Q8BH95	Enoyl-CoA hydratase, mitochondrial	1.004	0.516	0.479	2.34E-04	1.72E-04	3.63	3.76	down	down
Q8BK48	Pyrethroid hydrolase Ces2e	1.019	0.443	0.385	3.55E-03	2.40E-03	2.45	2.62	down	down
Q8C102	Polypeptide N-acetylgalactosaminyltransferase 5	1.011	0.589	0.508	1.73E-02	5.21E-03	1.76	2.28	non	down
Q8CI85	Carbonic anhydrase 12	1.000	0.507	0.519	3.69E-08	1.24E-07	7.43	6.91	down	down
Q8K2B3	Succinate dehydrogenase [ubiquinone] flavoprotein subunit, mitochondrial	1.025	0.761	0.610	1.19E-01	2.74E-02	0.92	1.56	non	down
Q8QZR3	Pyrethroid hydrolase Ces2a	1.004	0.468	0.429	6.40E-05	4.27E-05	4.19	4.37	down	down
Q8QZT1	Acetyl-CoA acetyltransferase, mitochondrial	1.000	0.626	0.619	1.45E-05	3.97E-06	4.84	5.40	down	down
Q8VCC2	Liver carboxylesterase 1	1.028	0.537	0.449	1.79E-02	8.91E-03	1.75	2.05	down	down
Q8VDN2	Sodium/potassium-transporting ATPase subunit alpha-1	1.049	0.408	0.552	1.80E-02	4.87E-02	1.74	1.31	down	down
Q91VD9	NADH-ubiquinone oxidoreductase 75 kDa subunit, mitochondrial	1.026	0.781	0.625	1.50E-01	3.28E-02	0.82	1.48	non	down
Q91WG0	Acylcarnitine hydrolase	1.045	0.354	0.274	1.08E-02	6.32E-03	1.97	2.20	down	down
Q922R8	Protein disulfide-isomerase A6	1.037	0.763	0.584	1.79E-01	4.28E-02	0.75	1.37	non	down
Q99J99	3-mercaptopyruvate sulfurtransferase	1.016	0.331	0.405	1.01E-03	2.26E-03	3.00	2.65	down	down
Q99LC5	Electron transfer flavoprotein subunit alpha, mitochondrial	1.000	0.462	0.469	1.97E-08	2.58E-08	7.70	7.59	down	down

Q9CPP6	NADH dehydrogenase [ubiquinone] 1 alpha subcomplex subunit 5	1.026	0.711	0.575	7.68E-02	2.07E-02	1.11	1.68	non	down
Q9CR68	Cytochrome b-c1 complex subunit Rieske, mitochondrial	1.006	0.693	0.621	4.96E-03	1.69E-03	2.30	2.77	non	down
Q9CZ13	Cytochrome b-c1 complex subunit 1, mitochondrial	1.033	0.684	0.529	8.05E-02	2.14E-02	1.09	1.67	non	down
Q9CZS1	Aldehyde dehydrogenase X, mitochondrial	1.039	0.333	0.443	6.63E-03	1.47E-02	2.18	1.83	down	down
Q9D051	Pyruvate dehydrogenase E1 component subunit beta, mitochondrial	1.006	0.470	0.530	2.05E-04	4.16E-04	3.69	3.38	down	down
Q9D306	Alpha-1,3-mannosyl-glycoprotein 4-beta-N-acetylglucosaminyltransferase C	1.022	0.659	0.563	3.66E-02	1.48E-02	1.44	1.83	non	down
Q9D309	Protein FAM3B	1.044	0.737	0.552	1.66E-01	4.21E-02	0.78	1.38	non	down
Q9D312	Keratin, type I cytoskeletal 20	1.044	0.497	0.388	2.93E-02	1.34E-02	1.53	1.87	down	down
Q9D855	Cytochrome b-c1 complex subunit 7	1.011	0.645	0.551	7.34E-03	2.36E-03	2.13	2.63	non	down
Q9DCW4	Electron transfer flavoprotein subunit beta	1.001	0.467	0.497	2.36E-06	4.44E-06	5.63	5.35	down	down
Q9JI67	Beta-1,3-galactosyltransferase 5	1.031	0.432	0.560	8.34E-03	2.54E-02	2.08	1.60	down	down
Q9NYQ2	Hydroxyacid oxidase 2	1.034	0.638	0.534	5.83E-02	2.58E-02	1.23	1.59	non	down
Q9R0A1	Chloride channel protein 2	1.012	0.610	0.544	6.65E-03	3.56E-03	2.18	2.45	non	down
Q9WTP7	GTP:AMP phosphotransferase AK3, mitochondrial	1.016	0.481	0.556	3.33E-03	6.77E-03	2.48	2.17	down	down
Q9Z0G9	Claudin-3	1.009	0.416	0.491	3.23E-04	8.05E-04	3.49	3.09	down	down
Q9Z261	Claudin-7	1.001	0.627	0.608	2.73E-05	7.41E-06	4.56	5.13	non	down

Q9Z2I0	LETM1 and EF-hand domain-containing protein 1, mitochondrial	1.001	0.677	0.647	4.92E-05	2.55E-05	4.31	4.59	non	down
Q9Z2I8	Succinyl-CoA ligase [GDP-forming] subunit beta, mitochondrial	1.002	0.535	0.568	6.27E-05	2.50E-04	4.20	3.60	down	down
O35640	Annexin A8	1.031	2.562	2.066	4.39E-05	7.69E-04	4.36	3.11	up	up
O88983	Syntaxin-8	1.008	1.764	1.871	1.86E-02	1.44E-03	1.73	2.84	non	up
P02301	Histone H3.3C	1.006	2.176	2.206	1.20E-03	1.15E-05	2.92	4.94	up	up
P06800	Receptor-type tyrosine-protein phosphatase C	1.011	3.311	2.912	1.72E-06	5.29E-06	5.76	5.28	up	up
P07356	Annexin A2	1.005	2.381	2.134	3.30E-07	2.09E-06	6.48	5.68	up	up
P10404	MLV-related proviral Env polyprotein	1.007	2.777	2.575	6.43E-07	9.82E-07	6.19	6.01	up	up
P11672	Neutrophil gelatinase-associated lipocalin	1.040	2.854	2.272	1.01E-04	3.32E-04	4.00	3.48	up	up
P14131	40S ribosomal protein S16	1.044	1.466	1.957	8.61E-02	3.73E-03	1.07	2.43	non	up
P14824	Annexin A6	1.009	3.941	3.577	5.48E-04	4.31E-05	3.26	4.37	up	up
P16045	Galectin-1	1.007	5.038	5.593	1.44E-04	1.35E-03	3.84	2.87	up	up
P17427	AP-2 complex subunit alpha-2	1.002	1.932	1.915	9.57E-06	2.66E-04	5.02	3.58	up	up
P17742	Peptidyl-prolyl cis-trans isomerase A	1.038	1.619	2.100	1.83E-02	1.49E-03	1.74	2.83	non	up
P18760	Cofilin-1	1.017	1.519	1.838	4.54E-03	3.14E-04	2.34	3.50	up	up
P19324	Serpin H1	1.039	2.963	2.580	1.68E-04	1.16E-04	3.77	3.94	up	up
P26041	Moesin	1.010	3.929	3.641	2.19E-04	3.34E-04	3.66	3.48	up	up
P28667	MARCKS-related protein	1.058	3.834	2.947	9.87E-03	4.34E-02	2.01	1.36	up	up
P30416	Peptidyl-prolyl cis-trans isomerase FKBP4	1.018	1.385	1.669	2.53E-02	1.24E-03	1.60	2.91	non	up

P40240	CD9 antigen	1.028	1.374	1.701	6.72E-02	6.55E-03	1.17	2.18	non	up
P48036	Annexin A5	1.020	2.789	2.331	5.42E-06	3.06E-05	5.27	4.51	up	up
P54116	Erythrocyte band 7 integral membrane protein	1.028	1.854	2.386	5.00E-03	5.21E-04	2.30	3.28	up	up
P62267	40S ribosomal protein S23	1.007	2.562	2.426	2.32E-07	7.26E-06	6.63	5.14	up	up
P62702	40S ribosomal protein S4, X isoform	1.015	1.770	2.098	3.10E-04	3.49E-05	3.51	4.46	up	up
P62806	Histone H4	1.038	3.030	2.312	1.80E-04	3.17E-04	3.74	3.50	up	up
P68510	14-3-3 protein eta	1.039	1.244	1.643	3.09E-01	1.41E-02	0.51	1.85	non	up
Q07076	Annexin A7	1.005	1.917	1.800	5.49E-06	7.88E-06	5.26	5.10	up	up
Q60634	Flotillin-2	1.009	2.286	2.020	8.68E-06	2.17E-05	5.06	4.66	up	up
Q61171	Peroxiredoxin-2	1.056	1.326	1.847	2.64E-01	9.75E-03	0.58	2.01	non	up
Q61233	Plastin-2	1.000	1.776	1.788	3.89E-05	7.74E-05	4.41	4.11	up	up
Q69ZN7	Myoferlin	1.005	2.270	2.116	3.91E-07	3.86E-06	6.41	5.41	up	up
Q7TPR4	Alpha-actinin-1	1.003	1.564	1.634	8.17E-05	2.34E-05	4.09	4.63	non	up
Q80UG5	Septin-9	1.014	1.970	1.713	1.95E-03	1.27E-03	2.71	2.90	up	up
Q8BP67	60S ribosomal protein L24	1.000	1.596	1.560	6.93E-08	7.38E-08	7.16	7.13	up	up
Q8R2S8	CD177 antigen	1.006	1.955	1.789	8.49E-05	8.21E-05	4.07	4.09	up	up
Q99JI6	Ras-related protein Rap-1b	1.018	1.311	1.566	9.28E-02	3.98E-03	1.03	2.40	non	up
Q9CR57	60S ribosomal protein L14	1.035	1.400	1.840	1.10E-01	9.81E-03	0.96	2.01	non	up
Q9DBG9	Tax1-binding protein 3	1.000	1.563	1.595	2.29E-07	8.69E-07	6.64	6.06	up	up
Q9DCD0	6-phosphogluconate dehydrogenase, decarboxylating	1.055	1.177	1.646	5.99E-01	3.57E-02	0.22	1.45	non	up
Q9JHS3	Ragulator complex protein LAMTOR2	1.026	1.947	1.762	7.84E-03	2.04E-03	2.11	2.69	up	up

Table S9. The common differential proteins between the cancer and normal, as well as cancer and adjacent membrane fraction enriched from colon mucosa

Accession	Gene	Ratio (Adj/Nor)	Ratio (Can/Nor)	Ratio (Can/Adj)
B2RX12	Abcc3	1.01	0.55	0.61
O35143	Atpif1	1.05	0.49	0.36
O70475	Ugdh	1.01	0.48	0.57
O88312	Agr2	1.01	0.18	0.17
O88343	Slc4a4	1.01	0.50	0.59
P00405	mtCo2	1.00	0.63	0.59
P01902	H2-K1	1.06	0.22	0.32
P08249	Mdh2	1.00	0.45	0.43
P18572	Bsg	1.02	0.46	0.55
P30275	Ckmt1	1.00	0.42	0.45
P38060	Hmgcl	1.02	0.50	0.58
P47738	Aldh2	1.01	0.50	0.56
P54869	Hmgcs2	1.01	0.32	0.28
P55088	Aqp4	1.07	0.28	0.41
P56391	Cox6b1	1.02	0.57	0.47
P56480	Atp5b	1.01	0.61	0.54
P62897	Cycs	1.04	0.61	0.48
P63038	Hspd1	1.01	0.49	0.57
Q07417	Acads	1.00	0.46	0.45
Q09199	B4galnt2	1.00	0.51	0.58
Q10470	Mgat3	1.01	0.38	0.44
Q3TMQ6	Ang4	1.04	0.34	0.44

Q61425	Hadh	1.01	0.53	0.51
Q62433	Ndrg1	1.01	0.58	0.52
Q64133	Maoa	1.01	0.58	0.54
Q7TST0	Btnl1	1.02	0.32	0.39
Q80V26	Impad1	1.01	0.53	0.56
Q810Q5	Nmes1	1.02	0.56	0.47
Q8BG05	Hnrnpa3	1.00	0.38	0.41
Q8BH95	Echs1	1.00	0.52	0.48
Q8BK48	Ces2e	1.02	0.44	0.38
Q8CI85	Ca12	1.00	0.51	0.52
Q8QZR3	Ces2a	1.00	0.47	0.43
Q8QZT1	Acat1	1.00	0.63	0.62
Q8VCC2	Ces1	1.03	0.54	0.45
Q8VDN2	Atp1a1	1.05	0.41	0.55
Q91WG0	Ces2c	1.05	0.35	0.27
Q99J99	Mpst	1.02	0.33	0.41
Q99LC5	Etfa	1.00	0.46	0.47
Q9CZS1	Aldh1b1	1.04	0.33	0.44
Q9D051	Pdhb	1.01	0.47	0.53
Q9D312	Krt20	1.04	0.50	0.39
Q9DCW4	Etfb	1.00	0.47	0.50
Q9JI67	B3galt5	1.03	0.43	0.56
Q9WTP7	Ak3	1.02	0.48	0.56
Q9Z0G9	Cldn3	1.01	0.42	0.49
Q9Z2I8	Suclg2	1.00	0.53	0.57
O35640	Anxa8	1.03	2.56	2.07

P02301	H3f3c	1.01	2.18	2.21
P06800	Ptprc	1.01	3.31	2.91
P07356	Anxa2	1.00	2.38	2.13
P10404	1 SV	1.01	2.78	2.57
P11672	Lcn2	1.04	2.85	2.27
P14824	Anxa6	1.01	3.94	3.58
P16045	Lgals1	1.01	5.04	5.59
P17427	Ap2a2	1.00	1.93	1.91
P18760	Cfl1	1.02	1.52	1.84
P19324	Serpinh1	1.04	2.96	2.58
P26041	Msn	1.01	3.93	3.64
P28667	Marcksl1	1.06	3.83	2.95
P48036	Anxa5	1.02	2.79	2.33
P54116	Stom	1.03	1.85	2.39
P62267	Rps23	1.01	2.56	2.43
P62702	Rps4x	1.01	1.77	2.10
P62806	Hist1h4a	1.04	3.03	2.31
Q07076	Anxa7	1.00	1.92	1.80
Q60634	Flot2	1.01	2.29	2.02
Q61233	Lcp1	1.00	1.78	1.79
Q69ZN7	Myof	1.00	2.27	2.12
Q80UG5	SEPTX9	1.01	1.97	1.71
Q8BP67	Rpl24	1.00	1.60	1.56
Q8R2S8	Cd177	1.01	1.95	1.79
Q9DBG9	Tax1bp3	1.00	1.56	1.60
Q9JHS3	Lamtor2	1.03	1.95	1.76

Table S10. The protein expression of 74 differential proteins detected in this study and reported by CPTAC database, and their KEGG pathways

*P1: Valine, leucine and isoleucine degradation; P2: Butanoate metabolism; P3: Metabolic pathways; P4: Fatty acid degradation; P5: Tryptophan metabolism P6: Pyruvate metabolism; P7: Lysine degradation; P8: Carbon metabolism; P9: Beta-Alanine metabolism; P10: Propanoate metabolism. #, protein regulation in tumor compared to adjacent normal colon tissues described in CPTAC database. Y, N and NA, represent $p < 0.05$, $p > 0.05$, and not found in CPTAC database.

**Accession**	**Gene**	**Name**	**%Cov (95)**	**Peptides (95%)**	**Ratio (Adj/Nor)**	**Ratio (Can/Nor)**	**Ratio (Can/Adj)**	P1*	P2*	P3*	P4*	P5*	P6*	P7*	P8*	P9*	P10*	Regulation in CPTAC#	P<0.05 in CPTAC #
B2RX12	Abcc3	Canalicular multispecific organic anion transporter 2	7.22	13	1.01	0.55	0.61											Down	Y
O35143	Atpif1	ATPase inhibitor, mitochondrial	7.55	1	1.05	0.49	0.36											Down	N
O35640	Anxa8	Annexin A8	12.84	4	1.03	2.56	2.07											NA	NA
O70475	Ugdh	UDP-glucose 6-dehydrogenase	21.50	10	1.01	0.48	0.57			Y								Down	Y
O88312	Agr2	Anterior gradient protein 2 homolog	33.14	15	1.01	0.18	0.17											Down	N
O88343	Slc4a4	Electrogenic sodium bicarbonate cotransporter 1	1.76	2	1.01	0.50	0.59											Down	Y
P00405	mtCo2	Cytochrome c oxidase subunit 2	7.49	2	1.00	0.63	0.59			Y								NA	NA

P01902	H2-K1	H-2 class I histocompatibility antigen, K-D alpha chain	5.43	2	1.06	0.22	0.32				NA	NA
P02301	H3f3c	Histone H3.3C	19.12	4	1.01	2.18	2.21				NA	NA
P06800	Ptprc	Receptor-type tyrosine-protein phosphatase C	2.17	3	1.01	3.31	2.91				Up	N
P07356	Anxa2	Annexin A2	52.51	42	1.00	2.38	2.13				Down	Y
P08249	Mdh2	Malate dehydrogenase, mitochondrial	54.73	23	1.00	0.45	0.43	Y	Y	Y	Up	Y
P10404	1 SV	MLV-related proviral Env polyprotein	5.30	3	1.01	2.78	2.57				NA	NA
P11672	Lcn2	Neutrophil gelatinase-associated lipocalin	7.00	2	1.04	2.85	2.27				Up	Y
P14824	Anxa6	Annexin A6	30.91	23	1.01	3.94	3.58				Down	Y
P16045	Lgals1	Galectin-1	17.78	3	1.01	5.04	5.59				Down	Y
P17427	Ap2a2	AP-2 complex subunit alpha-2	4.26	4	1.00	1.93	1.91				Down	Y
P18572	Bsg	Basigin	17.22	10	1.02	0.46	0.55				Down	Y
P18760	Cfl1	Cofilin-1	50.00	11	1.02	1.52	1.84				Down	Y
P19324	Serpinh1	Serpin H1	6.00	2	1.04	2.96	2.58				Up	Y
P26041	Msn	Moesin	13.52	10	1.01	3.93	3.64				Down	Y
P28667	Marcksl1	MARCKS-related protein	7.50	1	1.06	3.83	2.95				Up	Y
P30275	Ckmt1	Creatine kinase U-type, mitochondrial	30.86	14	1.00	0.42	0.45	Y			NA	NA

P38060	Hmgcl	Hydroxymethylglutaryl-CoA lyase, mitochondrial	5.85	2	1.02	0.50	0.58	Y	Y	Y							NA	NA
P47738	Aldh2	Aldehyde dehydrogenase, mitochondrial	27.36	17	1.01	0.50	0.56	Y		Y	Y	Y	Y	Y		Y	Down	Y
P48036	Anxa5	Annexin A5	34.17	14	1.02	2.79	2.33										Down	Y
P54116	Stom	Erythrocyte band 7 integral membrane protein	4.93	1	1.03	1.85	2.39										Down	Y
P54869	Hmgcs 2	Hydroxymethylglutaryl-CoA synthase, mitochondrial	27.17	20	1.01	0.32	0.28	Y	Y	Y							Down	Y
P55088	Aqp4	Aquaporin-4	3.10	1	1.07	0.28	0.41										NA	NA
P56391	Cox6b1	Cytochrome c oxidase subunit 6B1	53.49	7	1.02	0.57	0.47			Y							Down	Y
P56480	Atp5b	ATP synthase subunit beta, mitochondrial	49.53	38	1.01	0.61	0.54			Y							Down	Y
P62267	Rps23	40S ribosomal protein S23	13.29	2	1.01	2.56	2.43										Down	Y
P62702	Rps4x	40S ribosomal protein S4, X isoform	6.84	2	1.01	1.77	2.10										Up	Y
P62806	Hist1h4 a	Histone H4	50.49	6	1.04	3.03	2.31										Up	Y
P62897	Cycs	Cytochrome c, somatic	23.81	4	1.04	0.61	0.48										Down	N
P63038	Hspd1	60 kDa heat shock protein, mitochondrial	31.76	21	1.01	0.49	0.57										Up	Y
Q07076	Anxa7	Annexin A7	19.22	11	1.00	1.92	1.80										Down	Y

Q07417	Acads	Short-chain specific acyl-CoA dehydrogenase, mitochondrial	19.42	7	1.00	0.46	0.45	Y	Y	Y	Y				Y	Y	Y	Down	Y
Q09199	B4galnt2	Beta-1,4 N-acetylgalactosaminyltransferase 2	15.88	8	1.00	0.51	0.58											Down	Y
Q10470	Mgat3	Beta-1,4-mannosyl-glycoprotein 4-beta-N-acetylglucosaminyltransferase	1.86	1	1.01	0.38	0.44			Y								NA	NA
Q3TMQ6	Ang4	Angiogenin-4	6.94	1	1.04	0.34	0.44											NA	NA
Q60634	Flot2	Flotillin-2	17.99	7	1.01	2.29	2.02											Down	Y
Q61233	Lcp1	Plastin-2	5.58	3	1.00	1.78	1.79											Up	Y
Q61425	Hadh	Hydroxyacyl-coenzyme A dehydrogenase, mitochondrial	21.34	7	1.01	0.53	0.51	Y	Y	Y	Y	Y		Y				Down	Y
Q62433	Ndrg1	Protein NDRG1	12.94	4	1.01	0.58	0.52											Down	Y
Q64133	Maoa	Amine oxidase [flavin-containing] A	13.12	8	1.01	0.58	0.54			Y		Y						Down	Y
Q69ZN7	Myof	Myoferlin	0.59	2	1.00	2.27	2.12											Up	N
Q7TST0	Btnl1	Butyrophilin-like protein 1	2.16	1	1.02	0.32	0.39											NA	
Q80UG5	SEPTX9	Septin-9	4.63	4	1.01	1.97	1.71											NA	

Q80V26	Impad1	Inositol monophosphatase 3	11.52	4	1.01	0.53	0.56			Y								Up	Y
Q810Q5	Nmes1	Normal mucosa of esophagus-specific gene 1 protein	18.07	2	1.02	0.56	0.47											NA	NA
Q8BG05	Hnrnpa3	Heterogeneous nuclear ribonucleoprotein A3	16.89	6	1.00	0.38	0.41											Up	Y
Q8BH95	Echs1	Enoyl-CoA hydratase, mitochondrial	35.52	12	1.00	0.52	0.48	Y	Y	Y	Y	Y		Y	Y	Y	Y	Down	Y
Q8BK48	Ces2e	Pyrethroid hydrolase Ces2e	4.65	3	1.02	0.44	0.38											NA	NA
Q8BP67	Rpl24	60S ribosomal protein L24	14.65	2	1.00	1.60	1.56											Up	N
Q8CI85	Ca12	Carbonic anhydrase 12	2.54	1	1.00	0.51	0.52			Y								Down	Y
Q8QZR3	Ces2a	Pyrethroid hydrolase Ces2a	4.84	3	1.00	0.47	0.43											NA	NA
Q8QZT1	Acat1	Acetyl-CoA acetyltransferase, mitochondrial	17.45	9	1.00	0.63	0.62	Y	Y	Y	Y	Y	Y	Y	Y		Y	Down	Y
Q8R2S8	Cd177	CD177 antigen	11.51	9	1.01	1.95	1.79											Down	Y
Q8VCC2	Ces1	Liver carboxylesterase 1	3.54	2	1.03	0.54	0.45											Down	Y
Q8VDN2	Atp1a1	Sodium/potassium-transporting ATPase subunit alpha-1	40.57	89	1.05	0.41	0.55											Down	Y

Q91WG0	Ces2c	Acylcarnitine hydrolase	9.09	5	1.05	0.35	0.27										NA	NA
Q99J99	Mpst	3-mercaptopyruvate sulfurtransferase	14.48	4	1.02	0.33	0.41			Y							Down	Y
Q99LC5	Etfa	Electron transfer flavoprotein subunit alpha, mitochondrial	36.04	15	1.00	0.46	0.47										Down	Y
Q9CZS1	Aldh1b1	Aldehyde dehydrogenase X, mitochondrial	27.17	14	1.04	0.33	0.44	Y		Y	Y	Y	Y	Y		Y	Down	Y
Q9D051	Pdhb	Pyruvate dehydrogenase E1 component subunit beta, mitochondrial	19.22	6	1.01	0.47	0.53			Y			Y		Y		Down	Y
Q9D312	Krt20	Keratin, type I cytoskeletal 20	55.68	45	1.04	0.50	0.39										Down	Y
Q9DBG9	Tax1bp3	Tax1-binding protein 3	17.74	3	1.00	1.56	1.60										Down	Y
Q9DCW4	Etfb	Electron transfer flavoprotein subunit beta	19.61	6	1.00	0.47	0.50										Down	Y
Q9JHS3	Lamtor2	Ragulator complex protein LAMTOR2	7.20	1	1.03	1.95	1.76										Up	N
Q9JI67	B3galt5	Beta-1,3-galactosyltransferase 5	10.39	5	1.03	0.43	0.56			Y							Down	Y
Q9WTP7	Ak3	GTP:AMP phosphotransferase AK3, mitochondrial	48.90	11	1.02	0.48	0.56										Down	Y

Q9Z0G9	Cldn3	Claudin-3	5.48	2	1.01	0.42	0.49							Up	N
Q9Z2I8	Suclg2	Succinyl-CoA ligase [GDP-forming] subunit beta, mitochondrial	23.79	11	1.00	0.53	0.57		Y				Y	Down	Y

Table S11. The relationship between protein expressions and survival rate of patients with cancers analyzed by TCPA database

Gene	**Cancer name**	expression-survival rate	**Cancer name**	expression-survival rate	**Cancer name**	expression-survival rate	**Cancer name**	expression-survival rate	**Cancer name**	expression-survival rate	**Cancer name**	expression-survival rate	**Cancer name**	expression-survival rate	**Cancer name**	expression-survival rate	**Cancer name**	expression-survival rate
Suclg2*	**COAD**	lower-lower																
B4galnt2	**COAD**	lower-lower																
Serpinh1	**COAD**	lower-higher																
*Hmgcs2***	**COAD**	lower-lower	**KIRC**	lower-lower	**LIHC**	lower-lower												
Hadh	**KIRC**	lower-lower	**KIRR**	lower-lower	**LAML**	lower-higher	**PRAD**	lower-higher	**THYM**	lower-lower	**UCEC**	lower-lower						
ALDH2	**SKCM**	lower-lower	**HNSC**	lower-lower	**KICH**	lower-lower	**KIRC**	lower-lower	**KIRP**	lower-lower	**LGG**	lower-lower	**LIHC**	lower-lower	**LUSC**	lower-higher	**MESO**	lower-lower
Hmgcl	**GBM**	lower-higher	**KIRC**	lower-lower	**KIRP**	lower-lower	**LGG**	lower-higher										

Acat1	**KIRC**	lower-lower	**KIRP**	lower-lower	**LGG**	lower-lower	**LIHC**	lower-lower	**MESO**	lower-higher	**PRAD**	lower-higher						
Acads	**CESC**	lower-lower	**GBM**	lower-higher	**HNSC**	lower-lower	**KIRC**	lower-lower	**KIRP**	lower-lower	**LAML**	lower-higher	**LGG**	lower-higher	**LIHC**	lower-lower	**UCEC**	lower-lower
Echs1	**KIRC**	lower-lower	**KIRP**	lower-lower	**LAML**	lower-higher	**LGG**	lower-lower	**LIHC**	lower-lower	**UCEC**	lower-lower	**UVM**	lower-higher				
Aldh1b1	**ACC**	lower-higher	**HNSC**	lower-higher	**KIRC**	lower-lower	**MESO**	lower-higher	**THYM**	lower-higher								

**, enriched in a metabolic pathway; **, enriched in the pathway of valine, leucine and isoleucine degradation, and was related to the survival rate of patients with COAD; All the eight proteins enriched in the pathway of valine, leucine and isoleucine degradation were shown in iitalics.*

The abbreviation of each cancer was shown below

ACC	Adrenocortical carcinoma
BLCA	Bladder Urothelial Carcinoma
BRCA	Breast invasive carcinoma
CESC	Cervical squamous cell carcinoma and endocervical adenocarcinoma
CHOL	Cholangio carcinoma
COAD	Colon adenocarcinoma
DLBC	Lymphoid Neoplasm Diffuse Large B-cell Lymphoma
ESCA	Esophageal carcinoma
GBM	Glioblastoma multiforme
HNSC	Head and Neck squamous cell carcinoma
KICH	Kidney Chromophobe
KIRC	Kidney renal clear cell carcinoma
KIRP	Kidney renal papillary cell carcinoma
LAML	Acute Myeloid Leukemia

LGG	Brain Lower Grade Glioma
LIHC	Liver hepatocellular carcinoma
LUAD	Lung adenocarcinoma
LUSC	Lung squamous cell carcinoma
MESO	Mesothelioma
OV	Ovarian serous cystadenocarcinoma
PAAD	Pancreatic adenocarcinoma
PCPG	Pheochromocytoma and Paraganglioma
PRAD	Prostate adenocarcinoma
READ	Rectum adenocarcinoma
SARC	Sarcoma
SKCM	Skin Cutaneous Melanoma
STAD	Stomach adenocarcinoma
TGCT	Testicular Germ Cell Tumors
THCA	Thyroid carcinoma
THYM	Thymoma
UCEC	Uterine Corpus Endometrial Carcinoma
UCS	Uterine Carcinosarcoma
UVM	Uveal Melanoma

Table S12. The raw data of leucine, isoleucine and valine detected by UHPLC-MS/MS

Leucine

Sequence	Sample Name	Analyte Peak Area (counts)	Analyte Peak Height (cps)	Analyte Retention Time (min)	IS Peak Area (counts)	IS Peak Height (cps)	IS Retention Time (min)

1	Adj-1	5.76E+05	1.39E+05	1.01	2.67E+05	6.34E+04	0.992
2	Adj-2	9.54E+05	2.24E+05	1.01	3.15E+05	7.74E+04	0.987
3	Adj-3	6.68E+05	1.58E+05	1.01	2.94E+05	7.12E+04	0.99
4	Adj-4	1.48E+07	3.18E+06	1.01	2.78E+05	6.56E+04	0.989
5	Adj-5	1.21E+07	2.71E+06	1.01	2.69E+05	6.54E+04	0.987
6	Adj-6	1.89E+07	4.09E+06	1.01	2.53E+05	6.07E+04	0.99
7	Adj-7	3.67E+06	8.22E+05	1.01	2.82E+05	6.71E+04	0.983
8	Adj-1	3.28E+05	7.73E+04	1.01	1.60E+05	3.95E+04	0.99
9	Adj-2	6.62E+05	1.52E+05	1.01	2.28E+05	5.61E+04	0.989
10	Adj-3	5.23E+05	1.20E+05	1.01	2.28E+05	5.60E+04	0.989
11	Adj-4	9.17E+06	2.05E+06	1.01	1.66E+05	4.11E+04	0.988
12	Adj-5	8.91E+06	2.09E+06	1.01	1.92E+05	4.89E+04	0.991
13	Adj-6	1.53E+07	3.40E+06	1.01	2.14E+05	5.09E+04	0.989
14	Adj-7	3.22E+06	7.80E+05	1.01	2.48E+05	5.95E+04	0.99
15	Adj-1	3.89E+05	9.21E+04	1.01	1.91E+05	4.69E+04	0.99
16	Adj-2	6.29E+05	1.52E+05	1.01	2.18E+05	5.54E+04	0.993
17	Adj-3	4.33E+05	9.92E+04	1.01	1.95E+05	4.80E+04	0.989
18	Adj-4	1.26E+07	2.68E+06	1.01	2.48E+05	5.73E+04	0.99
19	Adj-5	1.07E+07	2.42E+06	1.01	2.41E+05	5.94E+04	0.99
20	Adj-6	1.12E+07	2.43E+06	1.01	1.49E+05	3.69E+04	0.989
21	Adj-7	2.24E+06	5.33E+05	1.01	1.73E+05	4.27E+04	0.99
22	Tum-1	9.79E+06	2.28E+06	1.01	3.01E+05	7.33E+04	0.991
23	Tum-2	3.69E+05	8.92E+04	1.01	2.77E+05	7.11E+04	0.99
24	Tum-3	1.89E+07	4.18E+06	1	2.74E+05	6.77E+04	0.984
25	Tum-4	1.89E+06	4.26E+05	1.01	2.95E+05	7.40E+04	0.989
26	Tum-5	2.39E+07	5.01E+06	1.01	2.66E+05	6.57E+04	0.989

27	Tum-6	1.92E+07	4.18E+06	1.01	2.69E+05	6.54E+04	0.99
28	Tum-7	1.90E+07	4.20E+06	1.01	2.78E+05	6.76E+04	0.989
29	Tum-1	7.40E+06	1.68E+06	1.01	2.26E+05	5.63E+04	0.987
30	Tum-2	3.41E+05	8.65E+04	1.01	2.65E+05	6.51E+04	0.992
31	Tum-3	1.63E+07	3.62E+06	1.01	2.39E+05	5.86E+04	0.986
32	Tum-4	1.69E+06	3.82E+05	1.01	2.71E+05	6.73E+04	0.989
33	Tum-5	2.45E+07	5.18E+06	1.01	2.83E+05	6.85E+04	0.99
34	Tum-6	1.89E+07	4.05E+06	1.01	2.70E+05	6.36E+04	0.99
35	Tum-7	1.31E+07	2.92E+06	1.01	1.86E+05	4.64E+04	0.988
36	Tum-1	7.89E+06	1.91E+06	1.01	2.40E+05	5.88E+04	0.991
37	Tum-2	3.35E+05	7.77E+04	1.01	2.66E+05	6.60E+04	0.989
38	Tum-3	1.75E+07	3.98E+06	1.01	2.63E+05	6.38E+04	0.991
39	Tum-4	1.51E+06	3.59E+05	1.01	2.50E+05	6.15E+04	0.99
40	Tum-5	1.98E+07	4.47E+06	1.01	2.28E+05	5.47E+04	0.992
41	Tum-6	1.51E+07	3.44E+06	1.01	2.07E+05	5.27E+04	0.989
42	Tum-7	1.17E+07	2.67E+06	1.02	1.69E+05	4.15E+04	0.991

Isoleucine

Sequence	Sample Name	Analyte Peak Area (counts)	Analyte Peak Height (cps)	Analyte Retention Time (min)	IS Peak Area (counts)	IS Peak Height (cps)	IS Retention Time (min)
1	Adj-1	4.55E+05	1.06E+05	1.01	2.67E+05	6.34E+04	0.992
2	Adj-2	7.34E+05	1.71E+05	1.01	3.15E+05	7.74E+04	0.987
3	Adj-3	5.44E+05	1.25E+05	1.01	2.94E+05	7.12E+04	0.99
4	Adj-4	1.17E+07	2.49E+06	1.01	2.78E+05	6.56E+04	0.989
5	Adj-5	9.55E+06	2.13E+06	1.01	2.69E+05	6.54E+04	0.987

6	Adj-6	1.50E+07	3.32E+06	1.01	2.53E+05	6.07E+04	0.99
7	Adj-7	2.84E+06	6.21E+05	1.01	2.82E+05	6.71E+04	0.983
8	Adj-1	2.45E+05	5.82E+04	1.01	1.60E+05	3.95E+04	0.99
9	Adj-2	5.16E+05	1.17E+05	1.01	2.28E+05	5.61E+04	0.989
10	Adj-3	3.98E+05	9.23E+04	1.01	2.28E+05	5.60E+04	0.989
11	Adj-4	6.98E+06	1.57E+06	1.01	1.66E+05	4.11E+04	0.988
12	Adj-5	6.74E+06	1.59E+06	1.01	1.92E+05	4.89E+04	0.991
13	Adj-6	1.18E+07	2.66E+06	1.01	2.14E+05	5.09E+04	0.989
14	Adj-7	2.45E+06	6.05E+05	1.01	2.48E+05	5.95E+04	0.99
15	Adj-1	2.93E+05	6.91E+04	1.01	1.91E+05	4.69E+04	0.99
16	Adj-2	4.83E+05	1.14E+05	1.01	2.18E+05	5.54E+04	0.993
17	Adj-3	3.29E+05	7.57E+04	1.01	1.95E+05	4.80E+04	0.989
18	Adj-4	9.82E+06	2.10E+06	1.01	2.48E+05	5.73E+04	0.99
19	Adj-5	8.34E+06	1.90E+06	1.01	2.41E+05	5.94E+04	0.99
20	Adj-6	8.49E+06	1.83E+06	1.01	1.49E+05	3.69E+04	0.989
21	Adj-7	1.71E+06	4.04E+05	1.01	1.73E+05	4.27E+04	0.99
22	Tum-1	7.84E+06	1.84E+06	1.01	3.01E+05	7.33E+04	0.991
23	Tum-2	2.84E+05	6.63E+04	1.01	2.77E+05	7.11E+04	0.99
24	Tum-3	1.52E+07	3.34E+06	1	2.74E+05	6.77E+04	0.984
25	Tum-4	1.50E+06	3.40E+05	1.01	2.95E+05	7.40E+04	0.989
26	Tum-5	1.91E+07	4.06E+06	1.01	2.66E+05	6.57E+04	0.989
27	Tum-6	1.54E+07	3.43E+06	1.01	2.69E+05	6.54E+04	0.99
28	Tum-7	1.51E+07	3.38E+06	1.01	2.78E+05	6.76E+04	0.989
29	Tum-1	5.76E+06	1.32E+06	1.01	2.26E+05	5.63E+04	0.987
30	Tum-2	2.67E+05	6.74E+04	1.01	2.65E+05	6.51E+04	0.992
31	Tum-3	1.28E+07	2.84E+06	1.01	2.39E+05	5.86E+04	0.986

32	Tum-4	1.29E+06	2.94E+05	1.01	2.71E+05	6.73E+04	0.989
33	Tum-5	1.97E+07	4.29E+06	1.01	2.83E+05	6.85E+04	0.99
34	Tum-6	1.49E+07	3.23E+06	1.01	2.70E+05	6.36E+04	0.99
35	Tum-7	1.00E+07	2.23E+06	1.01	1.86E+05	4.64E+04	0.988
36	Tum-1	6.06E+06	1.44E+06	1.01	2.40E+05	5.88E+04	0.991
37	Tum-2	2.66E+05	6.18E+04	1.01	2.66E+05	6.60E+04	0.989
38	Tum-3	1.38E+07	3.15E+06	1.01	2.63E+05	6.38E+04	0.991
39	Tum-4	1.16E+06	2.77E+05	1.01	2.50E+05	6.15E+04	0.99
40	Tum-5	1.56E+07	3.51E+06	1.01	2.28E+05	5.47E+04	0.992
41	Tum-6	1.19E+07	2.72E+06	1.01	2.07E+05	5.27E+04	0.989
42	Tum-7	8.91E+06	2.03E+06	1.01	1.69E+05	4.15E+04	0.991

Valine

Sequence	Sample Name	Analyte Peak Area (counts)	Analyte Peak Height (cps)	Analyte Retention Time (min)	IS Peak Area (counts)	IS Peak Height (cps)	IS Retention Time (min)
1	Adj-1	3.24E+05	4.12E+04	0.851	5.35E+05	6.59E+04	0.839
2	Adj-2	3.70E+05	5.03E+04	0.847	6.24E+05	8.26E+04	0.842
3	Adj-3	3.32E+05	4.51E+04	0.847	5.75E+05	7.39E+04	0.849
4	Adj-4	3.38E+06	4.08E+05	0.858	5.02E+05	6.09E+04	0.844
5	Adj-5	2.94E+06	3.51E+05	0.852	5.00E+05	6.23E+04	0.843
6	Adj-6	4.45E+06	5.32E+05	0.859	4.88E+05	6.04E+04	0.84
7	Adj-7	1.19E+06	1.64E+05	0.83	6.65E+05	9.34E+04	0.818
8	Adj-1	1.96E+05	2.74E+04	0.845	3.23E+05	4.10E+04	0.832
9	Adj-2	2.61E+05	3.38E+04	0.839	4.56E+05	5.67E+04	0.831
10	Adj-3	2.69E+05	3.32E+04	0.846	4.60E+05	5.62E+04	0.842

11	Adj-4	2.20E+06	2.75E+05	0.85	3.10E+05	3.94E+04	0.844
12	Adj-5	2.18E+06	2.66E+05	0.856	3.63E+05	4.55E+04	0.84
13	Adj-6	3.73E+06	4.58E+05	0.853	3.83E+05	4.85E+04	0.841
14	Adj-7	9.56E+05	1.30E+05	0.842	5.23E+05	7.10E+04	0.83
15	Adj-1	2.33E+05	3.25E+04	0.837	3.95E+05	5.07E+04	0.832
16	Adj-2	2.44E+05	3.31E+04	0.845	4.25E+05	5.29E+04	0.836
17	Adj-3	2.26E+05	3.03E+04	0.854	3.90E+05	5.04E+04	0.843
18	Adj-4	2.94E+06	3.60E+05	0.85	4.29E+05	5.31E+04	0.837
19	Adj-5	2.67E+06	3.35E+05	0.851	4.47E+05	5.65E+04	0.84
20	Adj-6	2.85E+06	3.38E+05	0.851	3.00E+05	3.67E+04	0.841
21	Adj-7	7.22E+05	9.80E+04	0.847	3.85E+05	5.14E+04	0.839
22	Tum-1	2.49E+06	3.17E+05	0.853	5.72E+05	7.36E+04	0.844
23	Tum-2	1.21E+05	1.69E+04	0.859	5.75E+05	6.99E+04	0.847
24	Tum-3	4.94E+06	5.69E+05	0.833	5.50E+05	6.70E+04	0.827
25	Tum-4	8.06E+05	1.10E+05	0.841	6.24E+05	8.20E+04	0.833
26	Tum-5	6.86E+06	8.39E+05	0.859	4.86E+05	6.02E+04	0.847
27	Tum-6	5.85E+06	7.06E+05	0.855	4.99E+05	6.13E+04	0.848
28	Tum-7	5.09E+06	6.22E+05	0.857	5.17E+05	6.32E+04	0.84
29	Tum-1	1.97E+06	2.45E+05	0.85	4.43E+05	5.68E+04	0.841
30	Tum-2	1.21E+05	1.68E+04	0.842	5.45E+05	6.82E+04	0.833
31	Tum-3	4.15E+06	5.07E+05	0.85	4.63E+05	5.78E+04	0.839
32	Tum-4	7.15E+05	9.75E+04	0.844	5.66E+05	7.55E+04	0.831
33	Tum-5	7.21E+06	8.85E+05	0.845	5.04E+05	6.43E+04	0.836
34	Tum-6	5.91E+06	7.25E+05	0.847	4.99E+05	6.28E+04	0.836
35	Tum-7	3.59E+06	4.32E+05	0.855	3.46E+05	4.27E+04	0.846
36	Tum-1	2.13E+06	2.79E+05	0.846	4.84E+05	6.48E+04	0.838

37	Tum-2	1.22E+05	1.79E+04	0.838	5.49E+05	7.24E+04	0.833
38	Tum-3	4.57E+06	5.59E+05	0.841	5.10E+05	6.31E+04	0.829
39	Tum-4	6.78E+05	8.66E+04	0.858	5.17E+05	6.59E+04	0.846
40	Tum-5	5.76E+06	6.79E+05	0.852	4.02E+05	4.84E+04	0.837
41	Tum-6	4.91E+06	6.14E+05	0.853	4.10E+05	5.29E+04	0.845
42	Tum-7	3.44E+06	4.24E+05	0.851	3.31E+05	4.24E+04	0.846
